# Controllable access to trifluoromethyl-containing indoles and indolines: palladium-catalyzed regioselective functionalization of unactivated alkenes with trifluoroacetimidoyl chlorides†

**DOI:** 10.1039/d2sc00546h

**Published:** 2022-03-03

**Authors:** Hefei Yang, Le-Cheng Wang, Yu Zhang, Dongling Zheng, Zhengkai Chen, Xiao-Feng Wu

**Affiliations:** Department of Chemistry, Key Laboratory of Surface & Interface Science of Polymer Materials of Zhejiang Province, Zhejiang Sci-Tech University Hangzhou 310018 People's Republic of China zkchen@zstu.edu.cn; Dalian National Laboratory for Clean Energy, Dalian Institute of Chemical Physics, Chinese Academy of Sciences Dalian 116023 Liaoning China xwu2020@dicp.ac.cn; Leibniz-Institut für Katalyse e. V. Albert-Einstein-Straβe 29a 18059 Rostock Germany xiao-feng.wu@catalysis.de

## Abstract

The synthesis of diverse products from the same starting materials is always attractive in organic chemistry. Here, a palladium-catalyzed substrate-controlled regioselective functionalization of unactivated alkenes with trifluoroacetimidoyl chlorides has been developed, which provides a direct but controllable access to a variety of structurally diverse trifluoromethyl-containing indoles and indolines. In more detail, with respect to γ,δ-alkenes, 1,1-geminal difunctionalization of unactivated alkenes with trifluoroacetimidoyl chloride enables the [4 + 1] annulation to produce indoles; as for β,γ-alkenes, a [3 + 2] heteroannulation with the hydrolysis product of trifluoroacetimidoyl chloride through 1,2-vicinal difunctionalization of alkenes occurs to deliver indoline products. The structure of alkene substrates differentiates the regioselectivity of the reaction.

## Abstract 2

A palladium-catalyzed dual functionalization of unactivated alkenes with trifluoroacetimidoyl chlorides toward the synthesis of structurally diverse trifluoromethyl-containing indoles and indolines has been developed.

## Graphical abstract

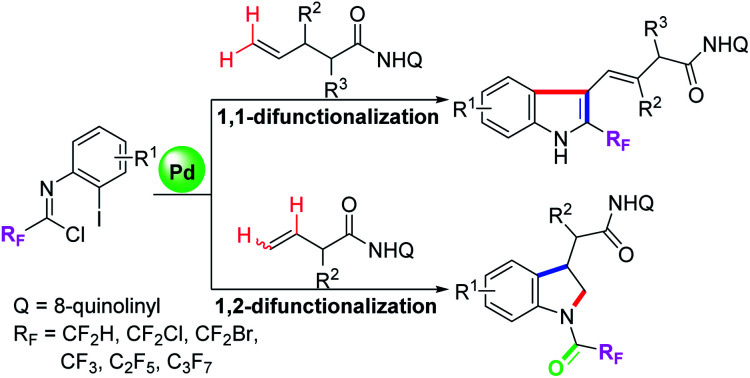

## Introduction

**Scheme 1:**
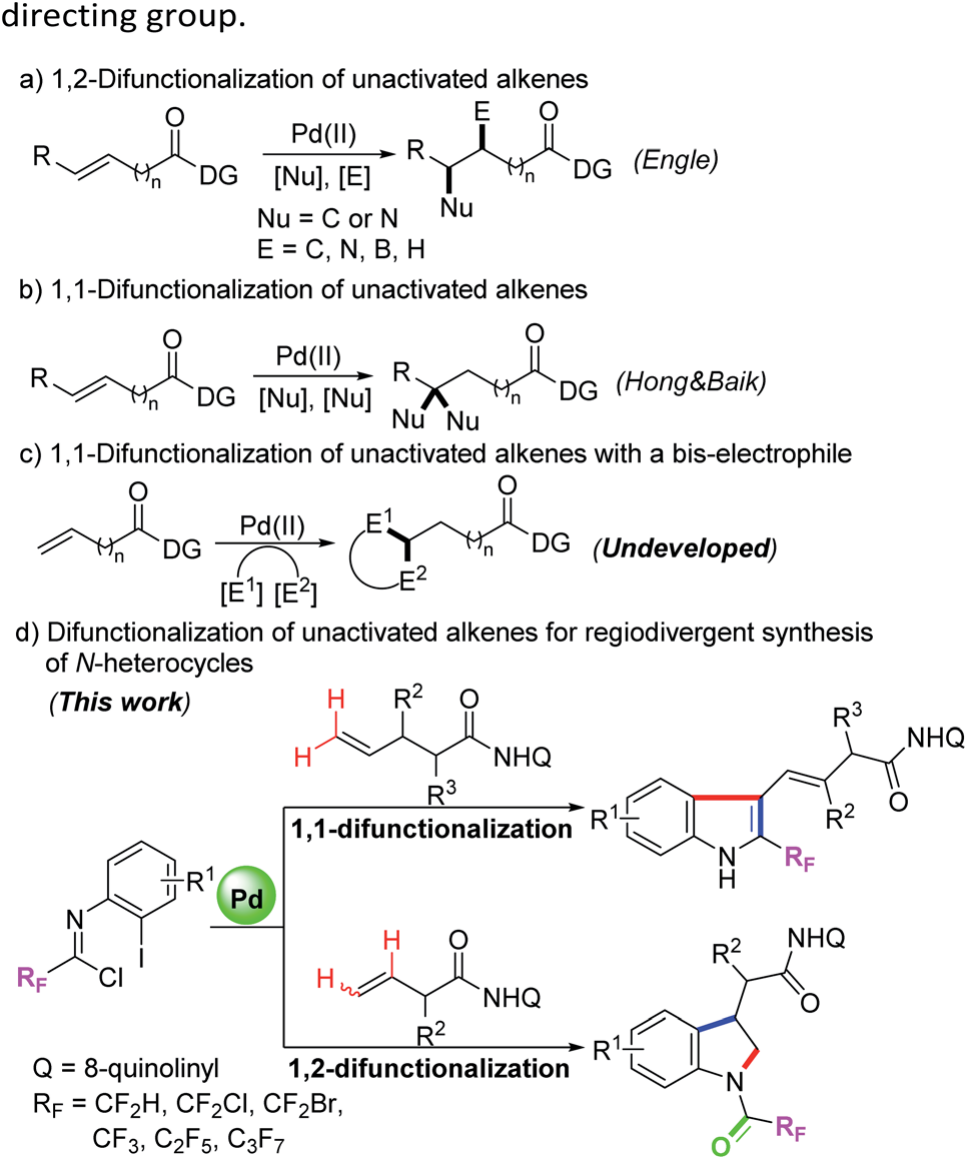
Palladium-catalyzed difunctionalization of unactivated alkenes.

Transition-metal-catalyzed difunctionalization of alkenes has emerged as a powerful synthetic strategy for the assembly of structurally complex molecules.^1^ The transformation can forge two new C–C or C–X bonds for installing two different components into the C–C double bonds, which has aroused tremendous interest from many research groups. For the more difficult difunctionalization of unactivated alkenes, in recent years, the Engle group and others have developed a series of transition-metal-catalyzed alkene 1,2-difunctionalization reactions with the assistance of the 8-aminoquinoline (AQ) auxiliary as a strongly coordinating bidentate directing group, including hydrofunctionalization,^2^ dicarbofunctionalization,^3^ carboamination,^4^ carboboration and aminoboration,^5^ and so on (Scheme 1a). In most cases of the above transformations, the nucleopalladated alkylpalladium(ii) species stabilized by a bidentate directing group were initially generated, and then protodepalladation or oxidative addition with electrophiles occurred to afford the difunctionalizated products.^2*a*,*b*^ Meanwhile, a competitive β-hydride elimination process was usually suppressed by the conformational rigidity of the directing group.

Compared with the rapid development of 1,2-vicinal difunctionalization protocols, reactions involving 1,1-geminal difunctionalization of unactivated alkenes have been rarely reported. To date, palladium-catalyzed 1,1-arylhalogenation, 1,1-diarylation and 1,1-arylborylation of alkenes have been explored by the use of prefunctionalized aryl sources.^6^ In 2019, Hong, Baik and their co-workers demonstrated a palladium(ii)-catalyzed site-selective 1,1-difunctionalization of unactivated alkenes with two nucleophiles, wherein a regioselective β-H elimination of the cationic palladacycle and subsequent migratory insertion were involved (Scheme 1b).^7^ In the 1,1-difunctionalization reactions, the key step lies in the β-H elimination from a less stable six-membered palladacycle to regenerate an olefin moiety, thereby enabling the following olefin insertion.^2*b*^ We surmised that the structural flexibility of the *in situ* formed palladacycle results in the regioselective difference of the 1,1-difunctionalization and 1,2-difunctionalization reactions. Pertinent to the present research, the utilization of difunctionalization of unactivated alkenes with two different electrophiles to produce structurally diverse heterocycles remains undeveloped and is of great significance (Scheme 1c).

Trifluoromethyl-substituted nitrogen-containing heterocycles as core skeletons widely exist in numerous bioactive and pharmaceutical molecules.^8^ Due to the unique properties of fluorine atoms, the physicochemical and pharmacological properties of the parent heterocyclic molecules could be obviously improved.^9^ Trifluoroacetimidoyl chlorides have been applied as a powerful and versatile fluorinated synthon for the assembly of trifluoromethyl-substituted N-heterocycles.^10^ Our group and others have developed a variety of synthetic methods for preparing various trifluoromethyl-containing N-heterocycles by the employment of trifluoroacetimidoyl chlorides as reactive partners.^11^ When the halogen atom was located at the *ortho* position of the aryl moiety of trifluoroacetimidoyl chloride, it will serve as a 4-atom reaction precursor with two electrophilic reactive sites, which can be adopted as useful building blocks to construct trifluoromethyl-substituted N-heterocycles. For instance, Zhu, Chen and co-workers demonstrated a palladium-catalyzed directed C-2 and C-3 dual C–H functionalization of *N*-(2-pyrimidyl)-indoles with trifluoroacetimidoyl chlorides for accessing fluorinated isocryptolepine analogues.^12^ Nevertheless, the more challenging task involving the combination of dual C–H functionalization of unactivated alkenes with trifluoroacetimidoyl chlorides is still elusive. Herein, we report our research finding of a palladium-catalyzed bidentate-directed 1,1-geminal and 1,2-vicinal difunctionalization of unactivated alkenes with trifluoroacetimidoyl chlorides for the regioselective synthesis of biologically important trifluoromethyl-containing indoles and indolines^13^ (Scheme 1d).

## Results and discussion

We commenced our studies by using trifluoroacetimidoyl chloride 1a and 4-pentenoic acid derivative 2a with an 8-aminoquinoline directing group as the model substrate (Table 1). The reaction was performed at 80 °C under N_2_ atmosphere in different solvents in the presence of Pd(OAc)_2_, PPh_3_ and Na_3_PO_4_. The results indicated that only THF could afford the 1,1-geminal difunctionalization product 3a with an alkenyl moiety at 3-position of indole in 37% yield (Table 1, entries 1–5). The exact structure of indole 3a was unambiguously confirmed by single X-ray diffraction analysis (CCDC: 2144265†).^14^ Other bidentate directing groups were also examined, including 2-methyl-8-quinolinyl, pyridyl, picolyl and 2-methylmercaptophenyl, and only trace of product 3a could be detected. The replacement of 8-quinolinyl with 1-naphthyl totally inhibited the reaction, highlighting the necessity of the 8-aminoquinoline directing group for the success of this reaction. Then, various bases were surveyed under the reaction system, which suggested the product 3a was obtained in 47% yield applying Na_2_CO_3_ as the base (Table 1, entries 6–9). The reaction conditions were further optimized by the use of a series of palladium catalysts, and Pd(hfac)_2_ could afford the best outcome (Table 1, entries 10–14). Furthermore, changing PPh_3_ with other phosphine ligands failed to give a better result (Table 1, entries 15–18). Elevating or lowering the reaction temperature exerted a negative effect on the reaction (Table 1, entries 19 and 20). Gratifyingly, the employment of a mixed solvent of THF and PhCF_3_ (v/v = 4/1) promoted the reaction to deliver 3a in 58% yield (Table 1, entry 21). Further optimization of the reaction conditions was implemented by the addition of diverse additives, such as TBAI, BQ, AgOAc or TEMPO, but the desired product 3a was not detected. The relatively lower yield of this reaction came from the inevitable residue of alkene 2a and the susceptibility of trifluoroacetimidoyl chloride 1a under the current reaction conditions.

**Table 1:** Optimization of reaction conditions^a^

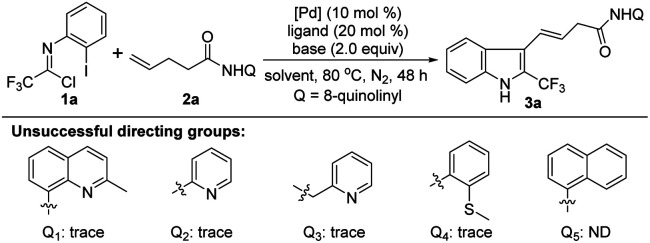					
Entry	[Pd] (mol%)	Ligand (mol%)	Base (equiv.)	Solvent (mL)	Yield^b^ (%)
1	Pd(OAc)_2_	PPh_3_	Na_3_PO_4_	MeCN	ND
2	Pd(OAc)_2_	PPh_3_	Na_3_PO_4_	1,4-Dioxane	Trace
3	Pd(OAc)_2_	PPh_3_	Na_3_PO_4_	Toluene	Trace
4	Pd(OAc)_2_	PPh_3_	Na_3_PO_4_	THF	37
5	Pd(OAc)_2_	PPh_3_	Na_3_PO_4_	HFIP	ND
6	Pd(OAc)_2_	PPh_3_	K_3_PO_4_	THF	38
7	Pd(OAc)_2_	PPh_3_	K_2_CO_3_	THF	Trace
8	Pd(OAc)_2_	PPh_3_	Na_2_CO_3_	THF	47
9	Pd(OAc)_2_	PPh_3_	Et_3_N	THF	13
10	Pd(dba)_2_	PPh_3_	Na_2_CO_3_	THF	40
11	Pd(PPh_3_)_4_	PPh_3_	Na_2_CO_3_	THF	35
12	Pd(PPh_3_)_2_Cl_2_	PPh_3_	Na_2_CO_3_	THF	40
13	Pd(TFA)_2_	PPh_3_	Na_2_CO_3_	THF	38
14	Pd(hfac)_2_	PPh_3_	Na_2_CO_3_	THF	51
15	Pd(hfac)_2_	P(4-F-Ph)_3_	Na_2_CO_3_	THF	48
16	Pd(hfac)_2_	P(4-OMe-Ph)_3_	Na_2_CO_3_	THF	23
17	Pd(hfac)_2_	dppf	Na_2_CO_3_	THF	32
18	Pd(hfac)_2_	Xantphos	Na_2_CO_3_	THF	ND
19	Pd(hfac)_2_	PPh_3_	Na_2_CO_3_	THF	42^c^
20	Pd(hfac)_2_	PPh_3_	Na_2_CO_3_	THF	23^d^
**21**	**Pd(hfac)**_**2**_	**PPh**_**3**_	**Na**_**2**_ **CO**_**3**_	**THF/PhCF**_**3**_ **(4/1)**	**58**
22	Pd(hfac)_2_	PPh_3_	Na_2_CO_3_	THF/PhCF_3_ (4/1)	ND^e^

a Reaction conditions: 1a (0.4 mmol), 2a (0.2 mmol), [Pd] (10 mol%), ligand (20 mol%), base (2.0 equiv.) in solvent (2.0 mL) at 80 °C under N_2_ atmosphere for 48 h.

b Isolated yields.

c 110 °C.

d 60 °C.

e The reaction was conducted with the addition of 2.0 equiv. of additive (TBAI, BQ, AgOAc or TEMPO). ND = no detection of the product. Pd(hfac)_2_ = palladium(ii) hexafluoroacetylacetonate.

With the establishment of the optimal reaction conditions, the scope and limitation of this 1,1-geminal difunctionalization reaction was explored by adopting a range of trifluoroacetimidoyl chlorides (Table 2). In general, the trifluoroacetimidoyl chlorides with electron-donating or electron-withdrawing groups in aryl moiety all could participate in this transformation to give rise to the corresponding 2-CF_3_-indole products 3b–m in low to moderate yields. In all cases, it was found that the γ,δ-alkene 2 remained and the excess trifluoroacetimidoyl chloride 1 was decomposed under the current reaction conditions. Extensive effort towards the screening of the reaction conditions was devoted to fully consume the alkene substrate 2 but without success. Therefore, another group of yield data was provided in the parentheses calculated based on the recovery of alkene 2. Trifluoroacetimidoyl chlorides bearing electron-donating groups 3b–g showed higher reactivity than that of substrates with electron-withdrawing groups 3h–m. The halogen substituents (F, Cl and Br) were also tolerated in this reaction (3h–m), but only lower efficiency was observed. Noteworthy is that other fluoroalkyl groups, including CF_2_H, C_2_F_5_ and C_3_F_7_, were also smoothly incorporated into the indole products 3n–p with moderate reactivity. Then, several substituted unactivated alkenes were evaluated to further explore the generality of the protocol. For instance, β-methyl- and phenyl-substituted 4-pentenoic amides reacted with 1a to deliver the indole products 3q–r in acceptable yields. α-Methyl- and cyclopropyl-substituted 4-pentenoic amides also acted as viable coupling partners (3s–t). Unfortunately, 1,2- and 1,1-disubstituted alkenes were not compatible with the reaction, presumably due to the remarkable steric factors. According to the structure of the obtained indole product 3, the presence of substituent at δ-position of 4-pentenoic amides will impede the formation of indole product.

**Table 2:** Substrate scope of 1,1-difunctionalization of γ,δ-alkenes^a^^,^^b^

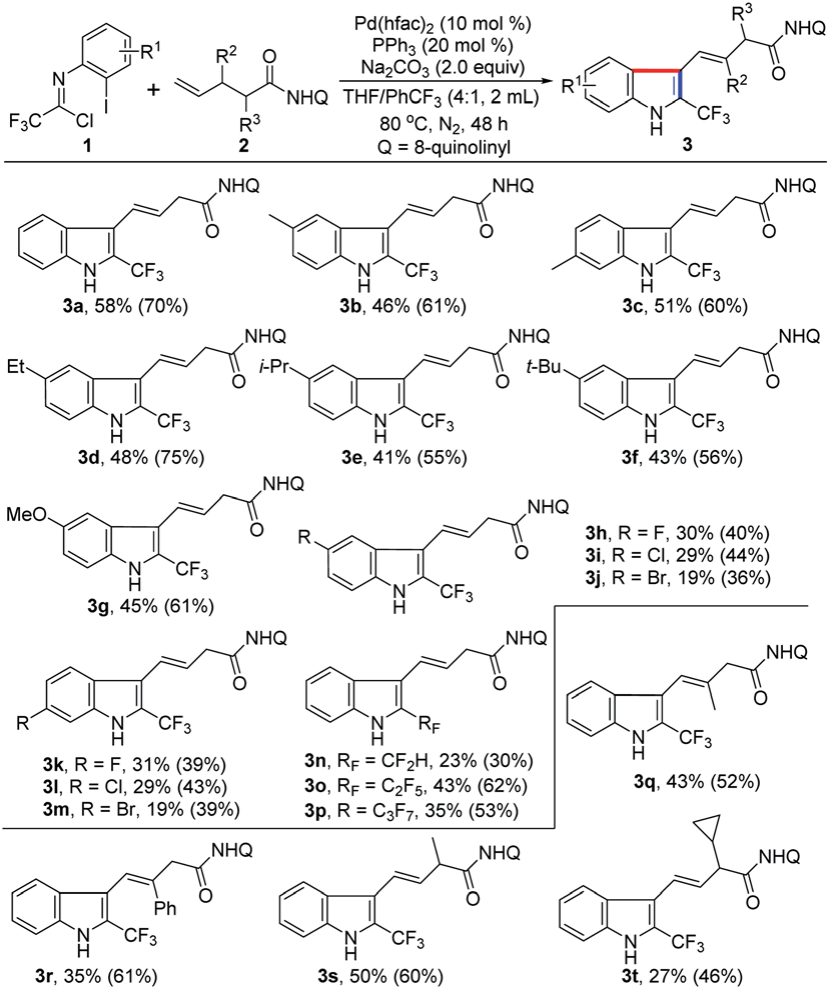

a Reaction conditions: 1 (0.4 mmol), 2 (0.2 mmol), Pd(hfac)_2_ (10 mol%), PPh_3_ (20 mol%), Na_2_CO_3_ (2.0 equiv.) in THF/PhCF_3_ (2.0 mL, v/v = 4/1) at 80 °C under N_2_ atmosphere for 48 h.

b Isolated yields and the yields in the parentheses were determined based on the recovery of alkene 2.

Interestingly, a unexpected *N*-trifluoroacetyl-substituted indoline product 4a was afforded in moderate yield when we used β,γ-alkenes as substrates to further evaluate the generality of this transformation (Table 3). We speculated that the hydrolysis of trifluoroacetimidoyl chlorides 1 occurs to give trifluoroacetamides, which undergo [3 + 2] heteroannulation with 3-butenoic acid derivatives in a 1,2-vicinal difunctionalization manner. An analogous process involving palladium-catalyzed [3 + 2] heteroannulation of non-conjugated alkenyl amides and *ortho*-iodoanilines/phenols for preparing 2,3-dihydrobenzofurans and indolines was disclosed by Engle and co-workers.^15^

**Table 3:** Substrate scope of 1,2-difunctionalization of β,γ-alkenes^a^^,^^b^

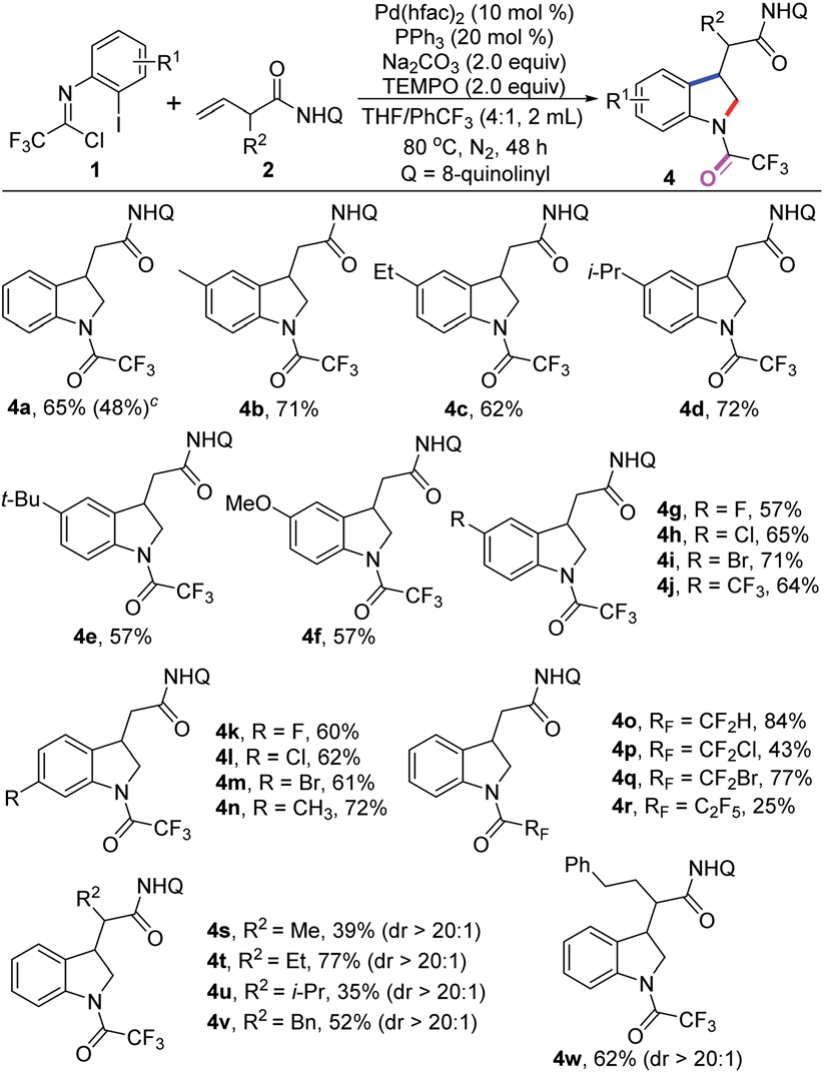

a Reaction conditions: 1 (0.4 mmol), 2 (0.2 mmol), Pd(hfac)_2_ (10 mol%), PPh_3_ (20 mol%), Na_2_CO_3_ (2.0 equiv.), TEMPO (2.0 equiv.) in THF/PhCF_3_ (2.0 mL, v/v = 4/1) at 80 °C under N_2_ atmosphere for 48 h.

b Isolated yields.

c Without the addition of TEMPO.

We next explored this protocol through slight modification of the reaction conditions by the addition of 2.0 equiv. of TEMPO, and the yield of product 4a was significantly increased to 65%. The scope of this 1,2-difunctionalization reaction was examined with diverse trifluoroacetimidoyl chlorides, and to our delight, the *N*-trifluoroacetyl-substituted indoline products 4a–n was delivered in moderate to good yields (Table 3).

A wide variety of trifluoroacetimidoyl chlorides were applicable to the reaction system, regardless of the electron properties of the aryl moiety. The halogen groups, even strong electron-withdrawing –CF_3_ group, could survive well in the reaction (4g–m). Apart from trifluoromethyl, other fluoroalkyl groups were also compatible with the reaction for producing the corresponding indoline products 4o–r in 25–84% yields. The structure of product 4l was also determined by single X-ray diffraction analysis (CCDC: 2144260†).^14^ In addition, β,γ-alkene substrates bearing different substituents at the α-position were suitable substrates under the optimal conditions to provide the desired products 4s–w in reasonable yields with excellent stereoselectivity. However, when internal alkenes were tested under the standard conditions, no desired reaction occurred. The yields of the 1,2-vicinal difunctionalization reaction are generally higher than that of 1,1-geminal difunctionalization reaction. It is well-known that chelation-stabilized five-membered palladacycle species which either generated from nucleopalladation of β,γ-alkenes or from C–H activation of the corresponding aliphatic chains could favorably react with carbon electrophiles, which was pioneered by Daugulis and co-workers.^16^

**Scheme 2:**
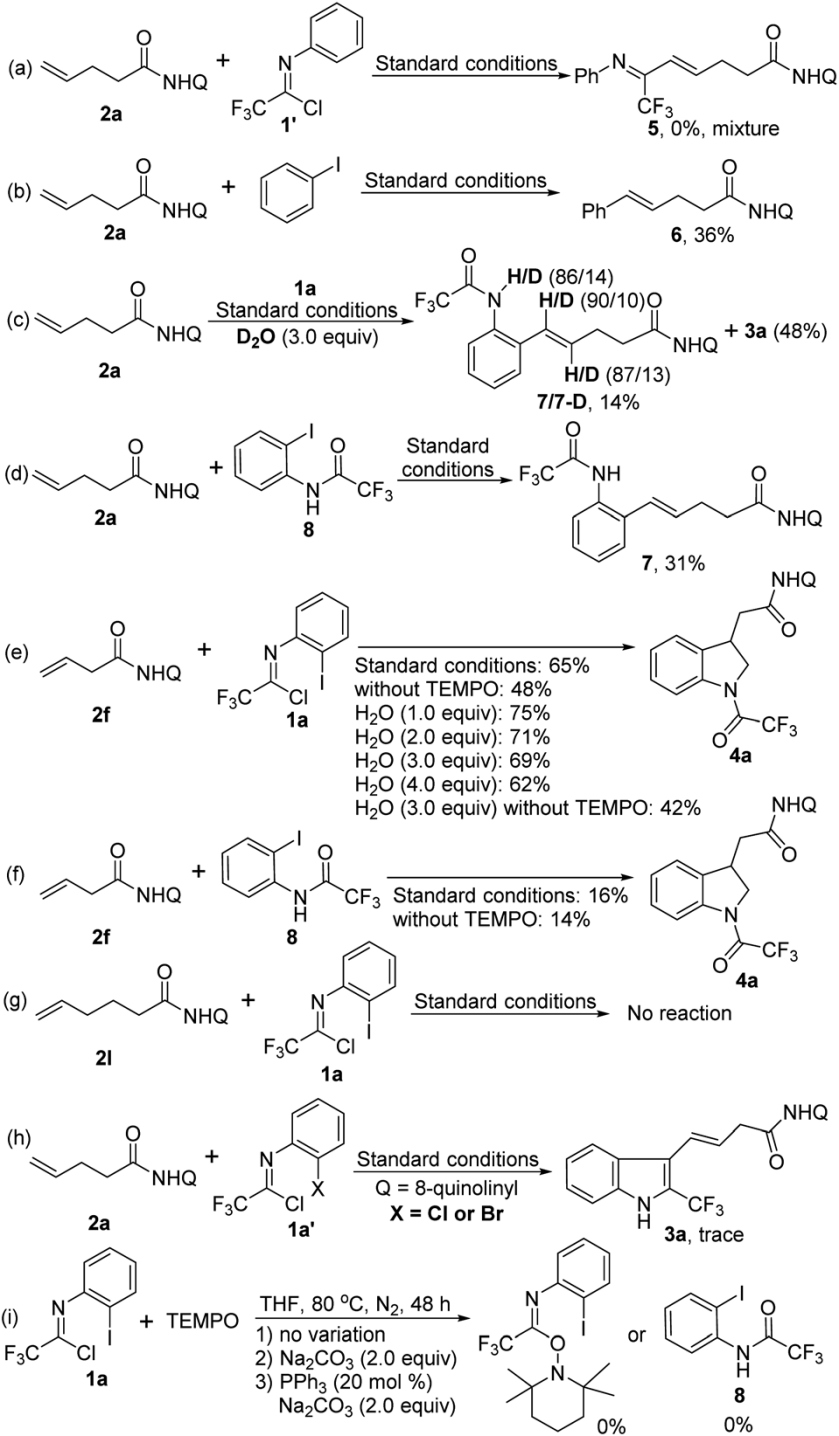
Control experiments.

In order to gain some deeper insight into the reaction pathway, a series of control experiments were performed as shown in Scheme 2. First, the reaction of alkene 2a with trifluoroacetimidoyl chloride 1′ without iodine substituent was carried out under standard conditions, but the coupling product 5 was not observed (Scheme 2a). The coupling reaction of 2a with iodobenzene proceeded smoothly to give the Heck-coupling product 6 in 36% yield (Scheme 2b). The above results indicated that the coupling reaction occurred initially between the alkene 2a and the C–I bond of trifluoroacetimidoyl chloride. When deuterated water as an additive was subjected into the reaction, a Heck-coupling product 7 with an amide moiety was obtained in 14% yield with the concomitant formation of the product 3a in 48% yield (Scheme 2c). The N–H bonds of amide moiety and the C(sp^2^)–H bonds of alkene moiety were both partially deuterated. The replacement of trifluoroacetimidoyl chloride 1a with its hydrolysis product 8 to react with alkene 2a under the standard conditions could afford the coupling product 7 in 31% yield without generation of the [3 + 2] heteroannulation indoline product (Scheme 2d). For the indoline synthesis using β,γ-alkenes, the addition of water into the reaction could give improved yields, whereas the reaction yield decreased in the absence of TEMPO or with higher loading of water (Scheme 2e). Combined with the yield data of standard conditions (65%) or without TEMPO (42%), it is evident that TEMPO greatly promotes the reaction, but the exact role of TEMPO in the reaction is still unclear. We also utilized the hydrolysis product 8 as the coupling partner in the optimal conditions or in the absence of TEMPO, the comparable poor yields were obtained (Scheme 2f), which revealed that trifluoroacetimidoyl chloride was possibly not rapidly transformed into amide 8 to participate in the reaction. Notably, the alkene 2l with the longer chain was unreactive in the reaction system, presumably due to the very unstable palladacycle intermediate (Scheme 2g). Only trace of the desired product could be detected when bromide or chloride analogues of trifluoroacetimidoyl chlorides were tested (Scheme 2h). Finally, reactions between trifluoroacetimidoyl chloride and TEMPO were also carried out to exclude the possible reaction pathway (Scheme 2i).

**Scheme 3:**
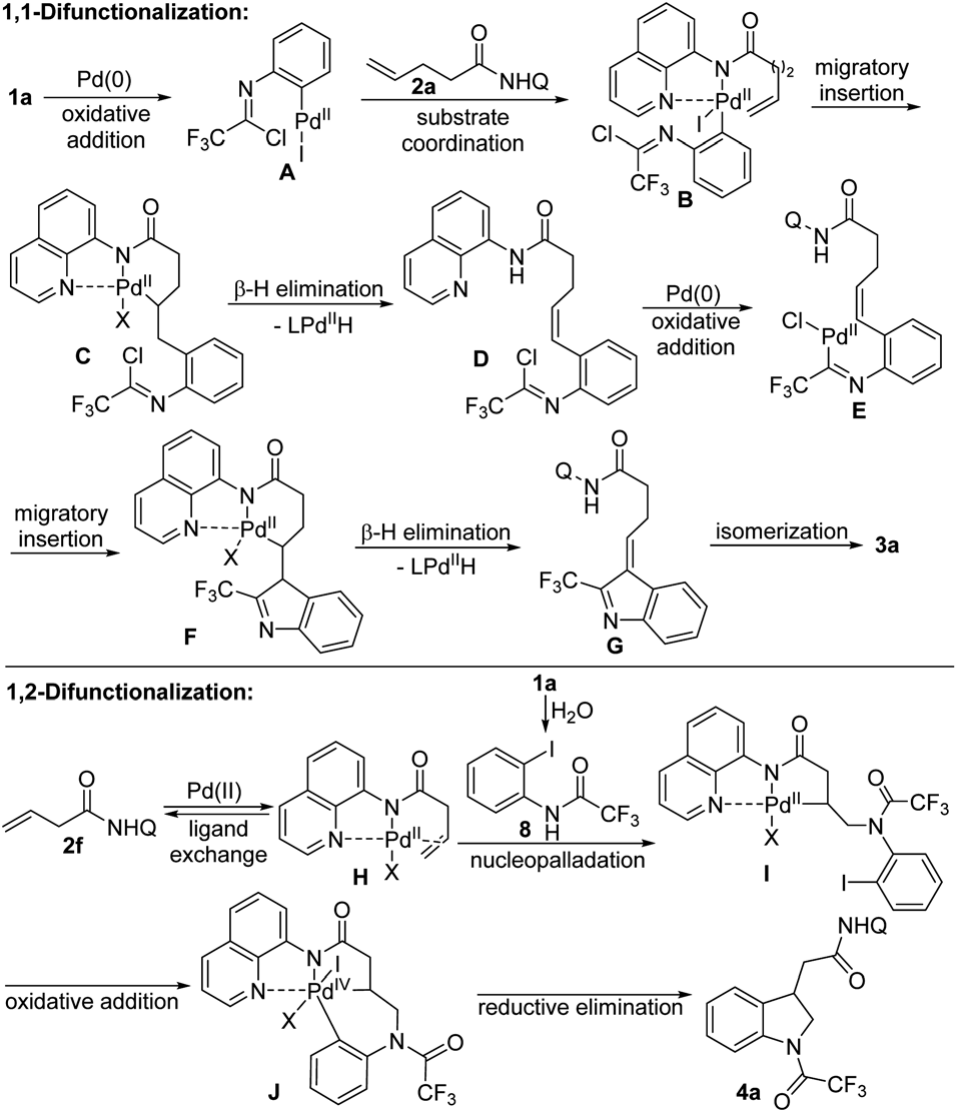
Plausible reaction mechanism.

Based on the mechanistic investigation and the precedent literature,^2*g*,12,15–17^ the plausible reaction mechanisms of two different difunctionalizations of unactivated alkenes were proposed as depicted in Scheme 3. For the 1,1-difunctionalization reaction, the oxidative addition of C–I bond of trifluoroacetimidoyl chloride 1a to Pd(0) generated imidoyl Pd(ii) intermediate A, which coordinated with the directing group in the alkene 2a to form complex B. Then, the 1,2-migratory insertion of B afforded the 6-membered palladacycle intermediate C, followed by the β-H elimination to deliver the alkene-tethered trifluoroacetimidoyl chloride D. Subsequently, the second sequence of oxidative addition of C–Cl bond of D, migratory insertion of E and β-H elimination of F occurred to give the 3*H*-indole G, which underwent the double bond isomerization process to furnish the desired 2-CF_3_-indole product 3a. For the 1,2-difunctionalization reaction, a similar Pd(ii)/Pd(iv) catalytic pathway based on the Engle's work was reasonable.^15^ Initially, the hydrolysis of trifluoroacetimidoyl chloride in the presence of trace amount of water in reaction system occurred to produce amide 8. The alkene coordination with Pd(ii) catalyst generated Pd(ii) species H, which underwent nucleopalladation with 8 to afford Pd(ii) complex I. Then, the oxidative addition of C–I bond of aryl moiety to Pd(ii) center delivered Pd(iv) intermediate J, followed by the reductive elimination step to give rise to the final indoline product 4a. Although the exact role of TEMPO in the 1,2-difunctionalization reaction is still unclear, we propose that TEMPO reoxidize Pd(0) that was formed from during the reaction to the catalytically active Pd(ii) form.

**Scheme 4:**
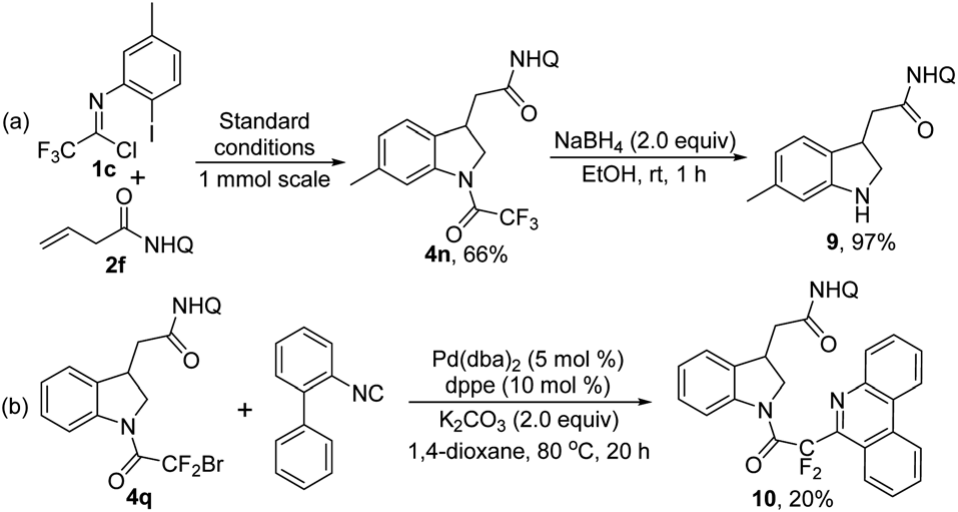
Scale-up reaction and synthetic transformations.

We also explored the practicability of the reaction by performing a scale-up reaction. The reaction could be easily reproducible at 1.0 mmol scale for product 4n with slightly decreased efficiency (66%) and the trifluoroacetyl group of 4n could be readily removed in the presence of NaBH_4_ (Scheme 4a). With regard to product 4q with a CF_2_Br moiety, a palladium-catalyzed radical annulation reaction with isocyanide^18^ was conducted for incorporating a phenanthridine scaffold into the indoline 4q to afford compound 10, albeit with low yield (Scheme 4b).

## Conclusions

In conclusion, we have developed a straightforward approach for the regioselective synthesis of structurally diverse trifluoromethyl-containing indoles and indolines *via* a palladium-catalyzed dual functionalization of unactivated alkenes with trifluoroacetimidoyl chlorides. The structure of alkene substrates controls the regioselectivity of the annulation reaction, which enables the divergence of 1,1-geminal and 1,2-vicinal difunctionalization of unactivated alkenes. Several control experiments have been conducted to elucidate the reaction mechanism. Further efforts towards the additional application of trifluoroacetimidoyl chlorides as versatile coupling partners in the C–H functionalization field should be pursued in future.

## Author contributions

XFW and ZC directed this project and prepared this manuscript. HY, LCW, YZ, and DZ performed all the experiments and prepared ESI.†

## Conflicts of interest

There are no conflicts to declare.

## Supplementary Material

SC-013-D2SC00546H-s001

SC-013-D2SC00546H-s002
