# Supplementary material for: Controllable access to trifluoromethyl-containing indoles and indolines: palladium-catalyzed regioselective functionalization of unactivated alkenes with trifluoroacetimidoyl chlorides
Source: Chem Sci. 2022 Mar 3;13(12):3526–32. doi: 10.1039/d2sc00546h (PMC8943892; doi:10.1039/d2sc00546h)
Supplement: SC-013-D2SC00546H-s001 [file SC-013-D2SC00546H-s001.pdf]

Electronic Supplementary Material (ESI) for Chemical Science.
This journal is © The Royal Society of Chemistry 2022

Supporting information

Controllable access to trifluoromethyl-containing indoles and indolines: Palladium-catalyzed regioselective functionalization of unactivated alkenes with trifluoroacetimidoyl chlorides

Hefei Yang,[a+] Le-Cheng Wang,[a+] Yu Zhang,[a] Dongling Zheng,[a] Zhengkai Chen,[a,*] Xiao-Feng Wu[b,c,*]

[a] Department of Chemistry, Key Laboratory of Surface & Interface Science of Polymer Materials of Zhejiang Province, Zhejiang Sci-Tech University, Hangzhou 310018, China

[b] Dalian National Laboratory for Clean Energy, Dalian Institute of Chemical Physics, Chinese Academy of Sciences, Dalian 116023, Liaoning, China

[c] Leibniz-Institut für Katalyse e. V., Albert-Einstein-Straβe 29a, 18059 Rostock, Germany

+ Contribute equally to this work.

E-mail: zkchen@zstu.edu.cn; xiao-feng.wu@catalysis.de

Contents

1 General Information 1
1.1 Preparation of Fluorinated Imidoyl Chlorides 1
1.2 Preparation of Alkenes 2
2 Experimental Procedures 3
2.1 Optimization of the Reaction Conditions 3
2.2 General Procedure for the Synthesis of Indole **3** and Indoline **4** 7
3 Control Experiments 8
4 Scale-up Reaction and Synthetic Transformations 12
5 The Crystal Structure of Product **3a** and **4l** 12
6 Characterization Data of the Corresponding Products 14
7 References 46
8 Copy of ^{1}H, ^{13}C and ^{19}F NMR Spectra of Products 46

1 General Information

Unless otherwise noted, all reactions were carried out under N_2 atmosphere. All reagents were from commercial sources and used as received without further purification. All solvents were dried by standard techniques and distilled prior to use. Column chromatography was performed on silica gel (200-300 meshes) using petroleum ether (bp. 60~90 °C) and ethyl acetate as eluent. ^{1}NMR spectra were recorded on a Bruker Avance operating at for ^{1}H NMR at 400 MHz, ^{13}C NMR at 100 MHz and ^{19}F NMR at 377 MHz and spectral data were reported in ppm relative to tetramethylsilane (TMS) as internal standard and $CDCl_3$ (^{1}H NMR δ 7.26, ^{13}C NMR δ 77.16) as solvent. All coupling constants (*J*) are reported in Hz. The following abbreviations were used to describe peak splitting patterns when appropriate: s = singlet, d = doublet, dd = double doublet, ddd = double doublet of doublets, t = triplet, dt = double triplet, q = quatriplet, m = multiplet, br = broad. Gas chromatography (GC) analyses were performed on a Shimadzu GC-2014C chromatograph equipped with a FID detector. Mass spectra (MS) were measured on spectrometer by direct inlet at 70 eV. Mass spectroscopy data of the products were collected on an HRMS-TOF instrument or Waters TOFMS GCT Premier using EI or ESI ionization. Melting points were measured with WRR digital point apparatus and not corrected.

1.1 Preparation of Fluorinated Imidoyl Chlorides[1]

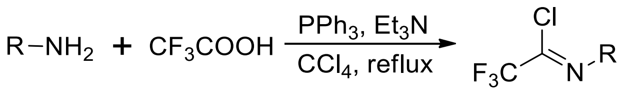

A 200 mL two-necked flask equipped with a septum cap, a condenser, and a Tefloncoated magnetic stir bar was charged with PPh_3 (34.5 g, 132 mmol), Et_3N (7.3 mL, 53 mmol), CCl_4 (21.1 mL, 220 mmol), and TFA (3.4 mL, 44 mmol) or other perfluoroalkanoic acids. After the solution was stirred for about 10 min (ice bath), amine (53 mmol) dissolved in CCl_4 (21.1 mL, 220 mmol) was added. The mixture was then refluxed under stirring (3 h). After the reaction was completed, residual solid Ph_3PO, PPh_3 and Et_3N-HC1 were washed with hexane several times. Then the hexane was filtered and concentrated under vacuum. The crude product was purified by column chromatography on silica gel or neutral alumina to afford the corresponding product.

1.2 Preparation of Alkenes 2 [2-3]

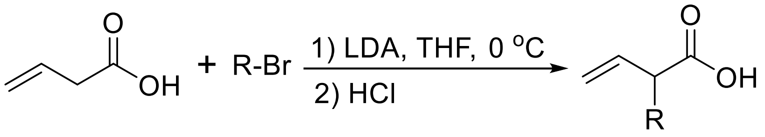

A solution of commercial LDA 2 M (11.8 mL, 23.5 mmol) in THF was cooled to ice-water temperature and a solution of 3-butenoic acid (1 mL, 11.77 mmol) in 10 mL of THF was added slowly over a period of 15 min. The resulting mixture was stirred at the same temperature for 45 min to obtain a deep yellow solution. A total of 1.1 equiv. (12.9 mmol) of the alkylating agent was added, where upon the reaction mixture immediately turned colorless. After 30 min at the same temperature and 1 h at room temperature, the pH of the solution was adjusted to 2.5 with 10% HCl. The organic phase was separated. The aqueous layer was saturated with solid NaCl and the mixture was extracted with ethyl acetate. The combined organic layers were dried over anhydrous Na_2SO_4 and filtered. Removal of solvents under reduced pressure followed by chromatography on silica gel (10-20% ethyl acetate/hexanes) produced the targeted molecules (28%-78% yield).

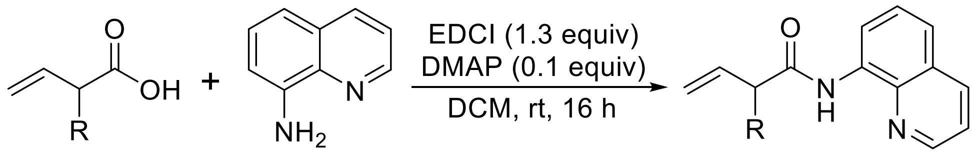

The acid (13 mmol) was charged into a 250 mL RB flask containing 100 mL DCM at room temperature. Quinolin-8-amine (10 mmol), EDCl (2.5 g, 13 mmol) and DMAP (122 mg, 1 mmol) were added successively, and the reaction was stirred at room temperature for 16 h. The brown solution was diluted with DCM (100 mL), washed with HCl (100 mL, 0.1 mol/L), sat. $NaHCO_3$ (100 mL×2) and brine (100 mL×1), and purified by column chromatography (20% EtOAc in petroleum ether) to afford the corresponding amide products.

2 Experimental Procedures

2.1 Optimization of the Reaction Conditions

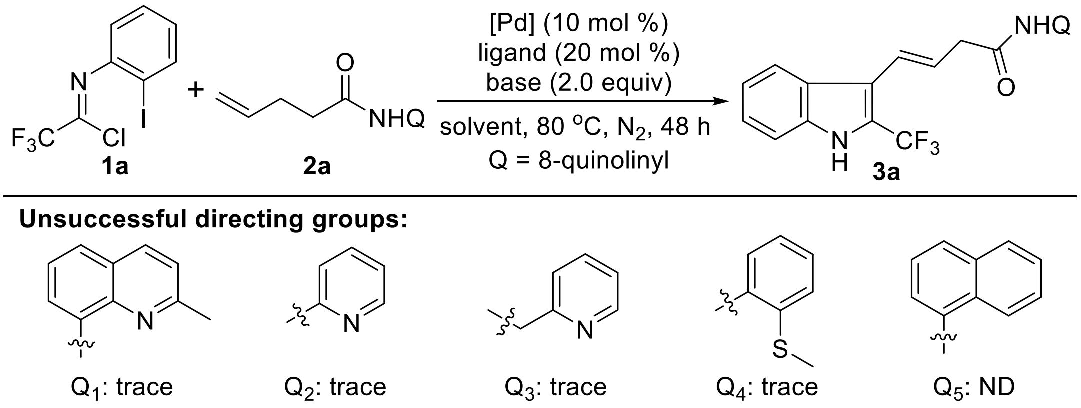

2.1.1 Screening of Solvent[a]

Entry	Solvent	Yield (%)[b]
1	1,4-dioxane	Trace
2	MeCN	N.D
3	DMF	Trace
4	DMSO	N.D
**5**	**THF**	**37%**
6	DCE	Trace
7	Toluene	Trace
8	2-MeTHF	34%
9	HFIP	N.D

[a]Reaction conditions: **2a** (0.2 mmol), **1a** (2.0 equiv.), $Pd(OAc)_2$ (10 mol%), PPh_3 (20 mol%), Na_3PO_4 (2.0 equiv.), solvent (2 mL), 80 °C, 48 h. [b]Isolated Yield.

2.1.2 Screening of Base[a]

Entry	Base	Yield (%)[b]
1	DIPEA	11%
2	Et_3N	13%

3	Cs_2CO_3	Trace
4	K_2CO_3	Trace
5	K_3PO_4	38%
6	Na_3PO_4	37%
**7**	**Na_2CO_3**	**47%**
8	*t*-BuOK	10%
9	/	N.D

[a]Reaction conditions: **2a** (0.2 mmol), **1a** (2.0 equiv), $Pd(OAc)_2$ (10 mol%), PPh_3 (20 mol%), base (2.0 equiv), THF (2 mL), 80 °C, 48 h. [b]Isolated Yield.

2.1.3 Screening of Temperature[a]

Entry	T/°C	Yield (%)[b]
1	130	37%
2	120	39%
3	110	42%
4	100	45%
5	90	44%
**6**	**80**	**47%**
7	60	23%

[a]Reaction conditions: **2a** (0.2 mmol), **1a** (2.0 equiv), $Pd(OAc)_2$ (10 mol%), PPh_3 (20 mol%), base (2.0 equiv), THF (2 mL), T, 48 h. [b]Isolated Yield.

2.1.4 Screening of Catalyst[a]

Entry	[Pd]	Yield (%)[b]
1	$Pd(OAc)_2$	47%
**2**	**$Pd(hfac)_2$**	**51%**

3	$Pd_2(dba)_3.CHCl_3$	42%
4	$Pd(dba)_2$	40%
5	$Pd(PPh_3)_4$	35%
6	$Pd(PPh_3)_2Cl_2$	40%
7	$PdCl_2$	36%
8	$Pd(TFA)_2$	38%
9	$Pd(acac)_2$	37%
10	$Pd_2(dba)_3$	35%
11	$Pd(MeCN)_2Cl_2$	41%

[a]Reaction conditions: **2a** (0.2 mmol), **1a** (2.0 equiv), [Pd] (10 mol%), PPh_3 (20 mol%), base (2.0 equiv), THF (2 mL), 80 ^{o}C, 48 h. [b]Isolated Yield.

2.1.5 Screening of Ligand[a]

Entry	Ligand	Yield (%)[b]
1	PCy_3	N.D
2	P(*p*-F-Ph)$_3$	48%
3	P(*p*-Me-Ph)$_3$	43%
4	DPPP	N.D
5	Xantphos	N.D
6	DPPF	32%
7	BINAP	N.D
8	Sphos	N.D
9	$HPPh_2$	N.D
10	PPh_2Py	N.D
11	P(*p*-CF_3-Ph)$_3$	28%
12	P(*m*-MeO-Ph)$_3$	26%
13	P(*p*-MeO-Ph)$_3$	23%
14	P(*o*-Me-Ph)$_3$	26%

15	P(*m*-Me-Ph)$_3$	26%
16	4-(Dimethylamino)PPh$_3$	24%
17	/	N.D

[a]Reaction conditions: **2a** (0.2 mmol), **1a** (2.0 equiv), Pd(OAc)$_2$ (10 mol%), ligand (20 mol%), base (2.0 equiv), THF (2 mL), 80 ^oC, 48 h. [b]Isolated Yield.

2.1.6 Screening of Additive[a]

Entry	Additive.	Yield (%)[b]
1	NaOAc	Trace
2	PivONa	Trace
3	PhCOONa	Trace
4	BQ	Trace
5	Ag_2CO_3	N.D
6	AgOAc	N.D
7	NaI	N.D
8	TBAI	N.D
9	PhI(OAc)$_2$	N.D
10	TEMPO	Trace
11	Cu(OAc)$_2$	N.D

[a]Reaction conditions: **2a** (0.2 mmol), **1a** (2.0 equiv), Pd(OAc)$_2$ (10 mol%), PPh$_3$ (20 mol%), base (2.0 equiv), additive (2.0 equiv), THF (2 mL), 80 ^oC, 48 h. [b]Isolated Yield.

2.1.7 Screening of amounts of ligand and solvent[a]

Entry	PPh$_3$ (mol%)	Solvent (2 mL)	Yield (%)[b]
1	30	THF (2 mL)	46%

2	10	THF (2 mL)	41%
3	20	THF:Dioxane=1:1	39%
**4**	**20**	**THF:CF_3Ph=4:1**	**58%**
5	20	THF:CF_3Ph=3:1	48%

[a]Reaction conditions: **2a** (0.2 mmol), **1a** (2.0 equiv), $Pd(OAc)_2$ (10 mol%), PPh_3 (20 mol%), base (2.0 equiv), additive (2.0 equiv), THF/CF_3Ph (1.6+0.4 mL), 80 °C, 48 h. [b]Isolated Yield.

2.2 General Procedure for the Synthesis of Indole 3 and Indoline 4

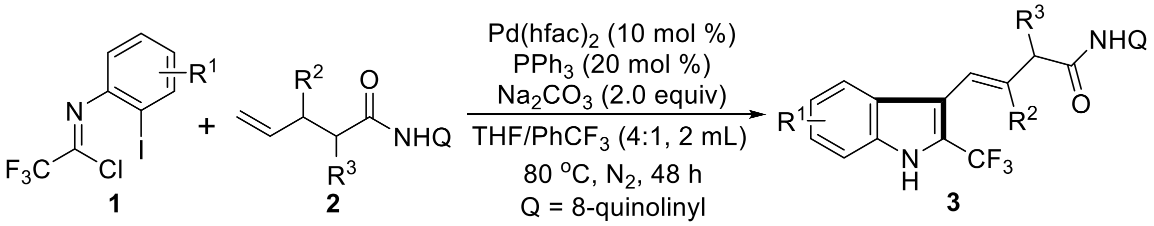

Under nitrogen atmosphere, **2** (0.2 mmol, 1.0 equiv), **1** (0.4 mmol, 2.0 equiv), $Pd(hfac)_2$ (10 mol%), PPh_3 (20 mol%, 10.5 mg), Na_2CO_3 (0.4 mmol, 2.0 equiv, 40.4 mg) and THF:CF_3Ph=4:1 (2 mL) (extra dry) were added to an oven-dried 15 mL reaction tube. Then the tube was sealed and the mixture was stirred at 80 °C (oil bath) for 48 h. After the reaction was completed, the mixture was slowly cooled to room temperature, and extracted with EtOAc for three times (3×10 mL). The extract was combined and concentrated under vacuum. The residue was purified by column chromatography on silica gel (petroleum ether/EtOAc) to yield the product **3**.

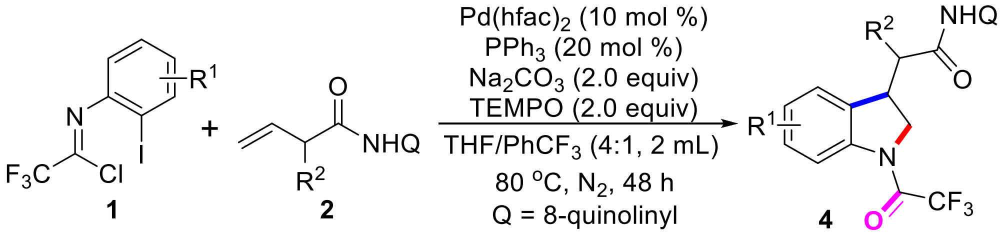

Under nitrogen atmosphere, **2** (0.2 mmol, 1.0 equiv), **1** (0.4 mmol, 2.0 equiv), $Pd(hfac)_2$ (10 mol%), PPh_3 (20 mol%, 10.5 mg), Na_2CO_3 (0.4 mmol, 2.0 equiv, 40.4 mg), TEMPO (0.4 mmol, 2.0 equiv, 62.5 mg) and THF:CF_3Ph=4:1 (2 mL) (extra dry) were added to an oven-dried 15 mL reaction tube. Then the tube was sealed and the mixture was stirred at 80 °C (oil bath) for 48 h. After the

reaction was completed, the mixture was slowly cooled to room temperature, and extracted with EtOAc for three times (3×10 mL). The extract was combined and concentrated under vacuum. The residue was purified by column chromatography on silica gel (petroleum ether/EtOAc) to yield the product **4**.

3 Control Experiments

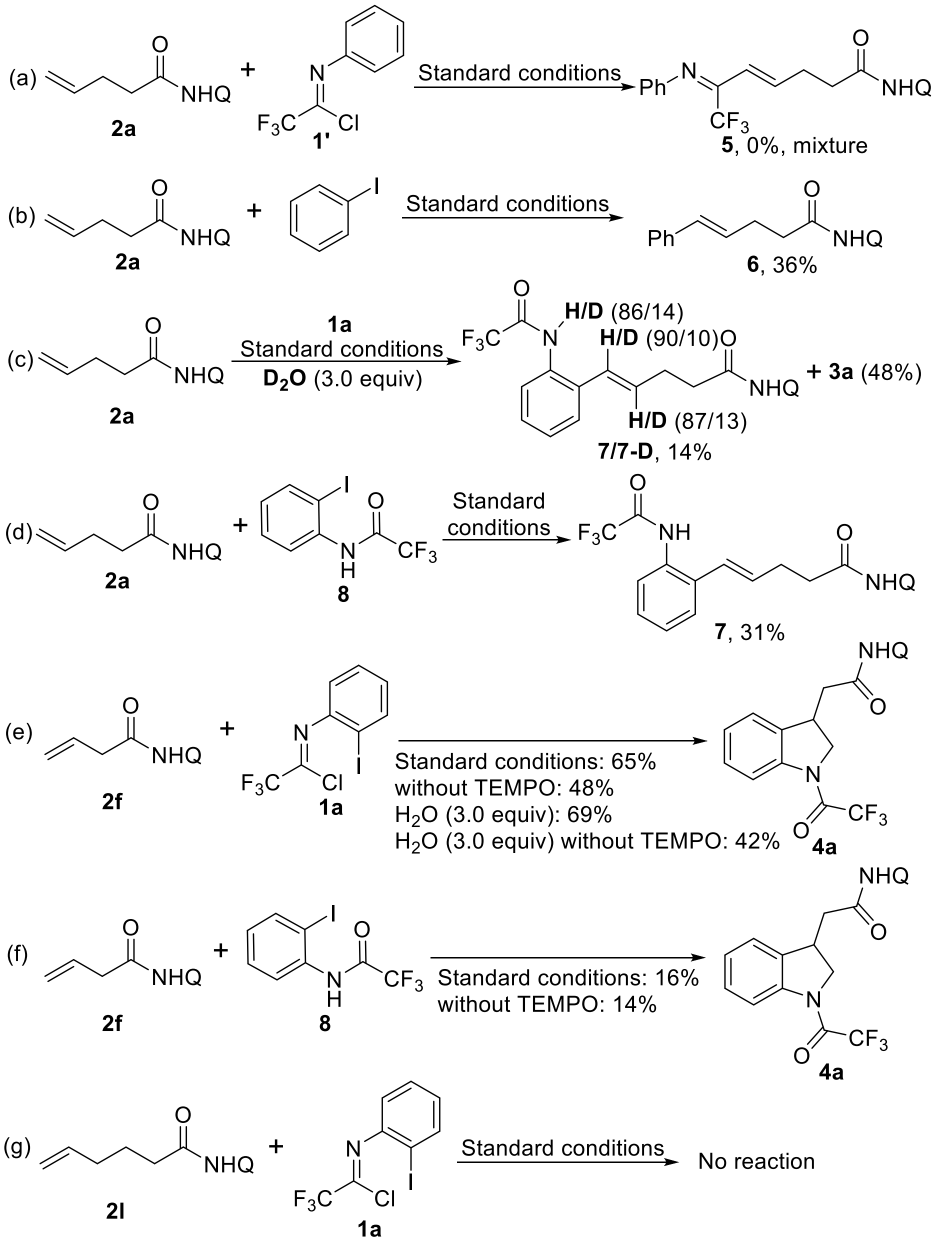

Eq a: Under nitrogen atmosphere, **2a** (0.2 mmol, 1.0 equiv), **1'** (0.4 mmol, 2.0 equiv), $Pd(hfac)_2$ (10 mol%), PPh_3 (20 mol%, 10.5 mg), Na_2CO_3 (0.4 mmol, 2.0 equiv, 40.4 mg) and THF:CF_3Ph=4:1 (2 mL) (extra dry) were added to an oven-dried 15 mL *In-Ex* tube. Then the tube was sealed and the

mixture was stirred at 80 °C (oil bath) for 48 h. The sample of the reaction was tested by ^{1}H NMR and the coupling product **5** was not detected by ^{1}H NMR.

Eq b: Under nitrogen atmosphere, **2a** (0.2 mmol, 1.0 equiv), iodobenzene (0.4 mmol, 2.0 equiv), $Pd(hfac)_2$ (10 mol%), PPh_3 (20 mol%, 10.5 mg), Na_2CO_3 (0.4 mmol, 2.0 equiv, 40.4 mg), and THF:CF_3Ph=4:1 (2 mL) (extra dry) were added to an oven-dried 15 mL *In-Ex* tube. Then the tube was sealed and the mixture was stirred at 80 °C (oil bath) for 48 h. After the reaction was completed, the mixture was slowly cooled to room temperature, and extracted with EtOAc for three times (3× 10 mL). The extract was combined and concentrated under vacuum. The residue was purified by column chromatography on silica gel (petroleum ether/EtOAc) to yield the product Heck-coupling **6** in 36% yield.

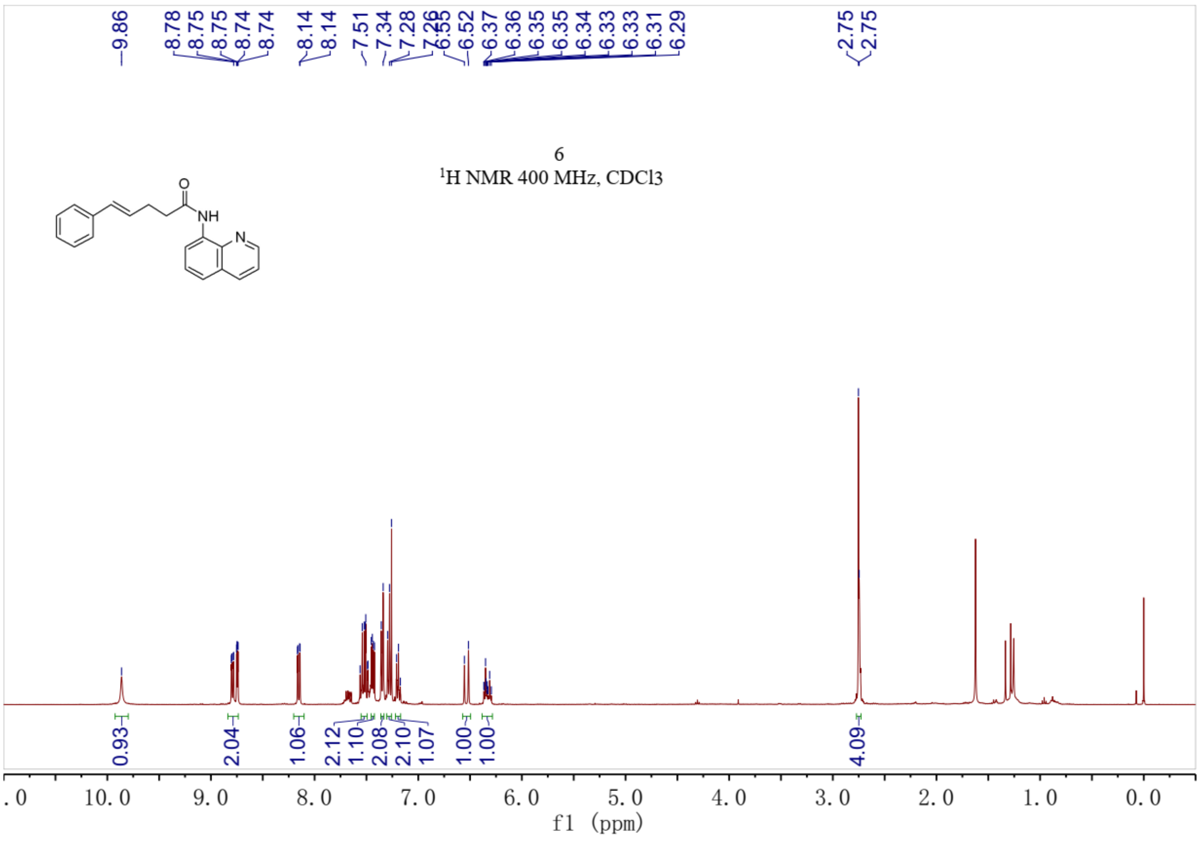

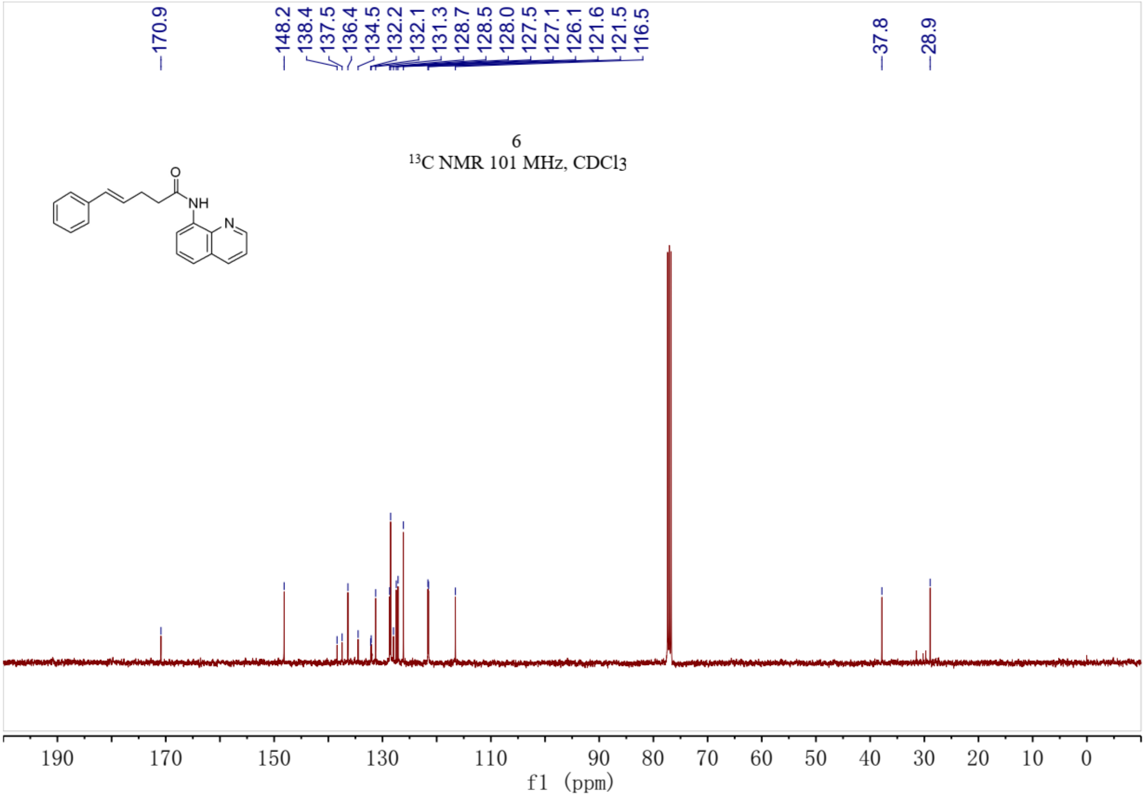

Eq c: Under nitrogen atmosphere, **2a** (0.2 mmol, 1.0 equiv), **1a** (0.4 mmol, 2.0 equiv), Pd(hfac)$_2$ (10 mol%), PPh_3 (20 mol%, 10.5 mg), Na_2CO_3 (0.4 mmol, 2.0 equiv, 40.4 mg), D_2O (0.6 mmol, 3.0 equiv, 12 mg) and THF:CF_3Ph=4:1 (2 mL) (extra dry) were added to an oven-dried 15 mL *In-Ex* tube. Then the tube was sealed and the mixture was stirred at 80 °C (oil bath) for 48 h. After the reaction was completed, the mixture was slowly cooled to room temperature, and extracted with EtOAc for three times (3×10 mL). The extract was combined and concentrated under vacuum. The residue was purified by column chromatography on silica gel (petroleum ether/EtOAc) to yield the product **3a** in 48% yield and the Heck-coupling product **7/7-D**. The sample of deuterated product **7/7-D** was tested by ^{1}H NMR.

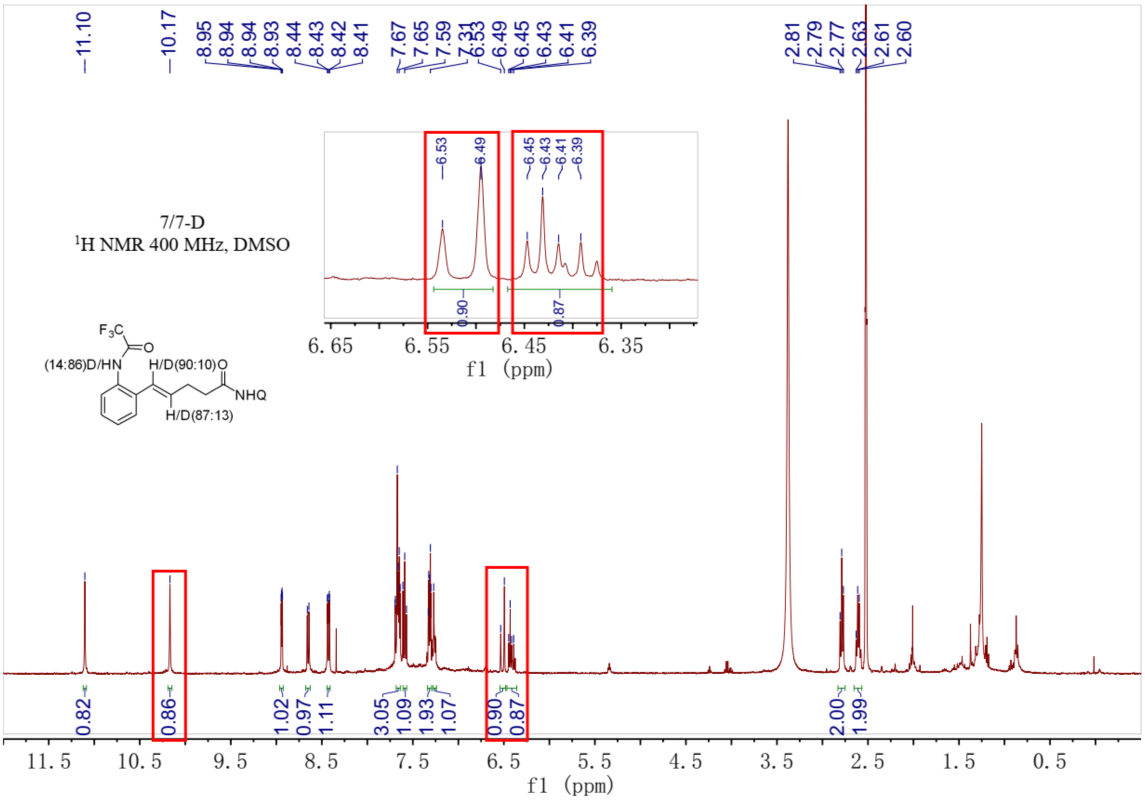

Eq d: Under Nitrogen atmosphere, **2a** (0.2 mmol, 1.0 equiv), **8** (0.4 mmol, 2.0 equiv), Pd(hfac)$_2$ (10 mol%), PPh_3 (20 mol%, 10.5 mg), Na_2CO_3 (0.4 mmol, 2.0 equiv, 40.4 mg) and THF:CF_3Ph=4:1 (2 mL) (extra dry) were added to an oven-dried 15 mL *In-Ex* tube. Then the tube was sealed and the mixture was stirred at 80 °C (oil bath) for 48 h. After the reaction was completed, the mixture was slowly cooled to room temperature, and extracted with EtOAc for three times (3×10 mL). The extract was combined and concentrated under vacuum. The residue was purified by column chromatography on silica gel (petroleum ether/EtOAc) to yield the coupling product **7** in 31% yield.

Eq e: Under Nitrogen atmosphere, **2f** (0.2 mmol, 1.0 equiv), **1a** (0.4 mmol, 2.0 equiv), Pd(hfac)$_2$ (10 mol%), PPh_3 (20 mol%, 10.5 mg), Na_2CO_3 (0.4 mmol, 2.0 equiv, 40.4 mg), H_2O (0.6 mmol, 3.0 equiv, 7.2 mg), TEMPO (0.4 mmol, 2 equiv, 62.5 mg) or without TEMPO and THF:CF_3Ph=4:1 (2 mL) (extra dry) were added to an oven-dried 15 mL *In-Ex* tube. Then the tube was sealed and the mixture was stirred at 80 °C (oil bath) for 48 h. After the reaction was completed, the mixture was slowly cooled to room temperature, and extracted with EtOAc for three times (3×10 mL). The extract was combined and concentrated under vacuum. The residue was purified by column

chromatography on silica gel (petroleum ether/EtOAc) to yield the product **4a** in 69% (with TEMPO) or 42% (without TEMPO) yield.

Eq f: Under nitrogen atmosphere, **2f** (0.2 mmol, 1.0 equiv), **8** (0.4 mmol, 2.0 equiv), Pd(hfac)$_2$ (10 mol%), PPh_3 (20 mol%, 10.5 mg), Na_2CO_3 (0.4 mmol, 2.0 equiv, 40.4 mg), TEMPO (0.4 mmol, 2 equiv, 62.5 mg) or without TEMPO and THF:CF_3Ph=4:1 (2 mL) (extra dry) were added to an oven-dried 15 mL *In-Ex* tube. Then the tube was sealed and the mixture was stirred at 80 °C (oil bath) for 48 h. After the reaction was completed, the mixture was slowly cooled to room temperature, and extracted with EtOAc for three times (3×10 mL). The extract was combined and concentrated under vacuum. The residue was purified by column chromatography on silica gel (petroleum ether/EtOAc) to yield the product **4a** in 16% (with TEMPO) or 14% (without TEMPO) yield.

Eq g: Under nitrogen atmosphere, alkene **2l** with the longer chain (0.2 mmol, 1.0 equiv), **1a** (0.4 mmol, 2.0 equiv), Pd(hfac)$_2$ (10 mol%), PPh_3 (20 mol%, 10.5 mg), Na_2CO_3 (0.4 mmol, 2.0 equiv, 40.4 mg) or with the addition of TEMPO (0.4 mmol, 2 equiv, 62.5 mg) and THF:CF_3Ph=4:1 (2 mL) (extra dry) were added to an oven-dried 15 mL *In-Ex* tube. Then the tube was sealed and the mixture was stirred at 80 °C (oil bath) for 48 h. After the reaction was completed, the mixture was slowly cooled to room temperature, and extracted with EtOAc for three times (3×10 mL). The extract was combined and concentrated under vacuum. No desired product was observed under the standard conditions.

4 Scale-up Reaction and Synthetic Transformations

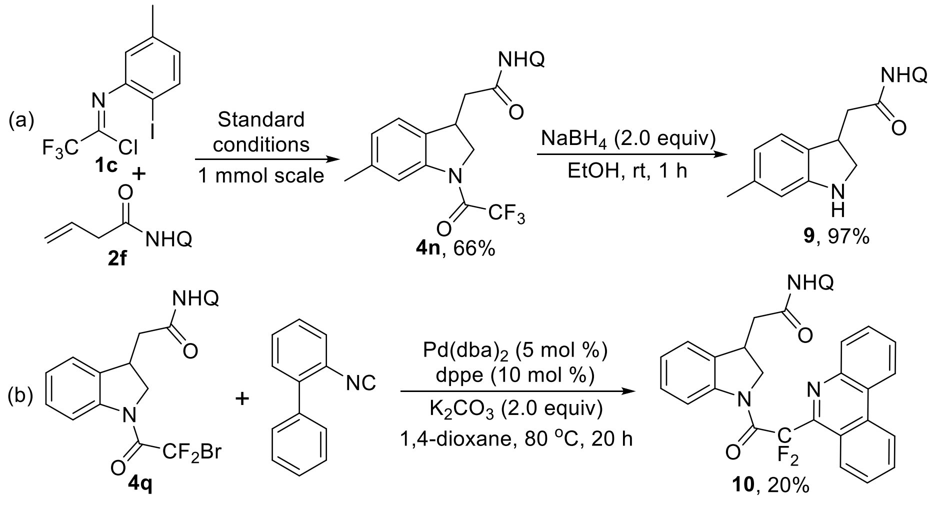

Eq a: Under nitrogen atmosphere, **2f** (1.0 mmol, 1.0 equiv), **1c** (2.0 mmol, 2.0 equiv), $Pd(hfac)_2$ (10 mol%), PPh_3 (20 mol%, 52.5 mg), Na_2CO_3 (2.0 mmol, 2.0 equiv, 202 mg), TEMPO (2.0 mmol, 2 equiv, 312.5 mg) and THF:CF_3Ph=4:1 (10 mL) (extra dry) were added to an oven-dried 50 mL *In-Ex* tube. Then the tube was sealed and the mixture was stirred at 80 °C (oil bath) for 48 h. After the reaction was completed, the mixture was slowly cooled to room temperature, and extracted with EtOAc for three times (3×20 mL). The extract was combined and concentrated under vacuum. The residue was purified by column chromatography on silica gel (petroleum ether/EtOAc) to yield the product **4n** in 66% yield.

Under nitrogen atmosphere, **4n** (0.2 mmol, 1.0 equiv), $NaBH_4$ (0.4 mmol, 2.0 equiv) and EtOH (4 mL) were added to an oven-dried 15 mL *In-Ex* tube. Then the tube was sealed and the mixture was stirred at room temperature (oil bath) for 1 h. After the reaction was completed, the mixture was slowly cooled to room temperature, and extracted with EtOAc for three times (3×10 mL). The extract was combined and concentrated under vacuum. The residue was purified by column chromatography on silica gel (petroleum ether/EtOAc) to yield the product **9** in 97% yield.

Eq b: Under nitrogen atmosphere, **4q** (0.1 mmol, 1.0 equiv), 2-isocyano-1,1'-biphenyl (0.15 mmol, 1.5 equiv), K_2CO_3 (0.2 mmol, 2.0 equiv), $Pd(PPh_3)_2Cl_2$ (5 mol%), dppe (10 mol%) and dioxane (1 mL) were added to an oven-dried 15 mL *In-Ex* tube. Then the tube was sealed and the mixture was stirred at 80 °C (oil bath) for 20 h. After the reaction was completed, the mixture was slowly cooled to room temperature, and extracted with EtOAc for three times (3×10 mL). The extract was combined and concentrated under vacuum. The residue was purified by column chromatography on silica gel (petroleum ether/EtOAc) to yield the product **10** in 20% yield.

5 The Crystal Structure of Product 3a and 4l

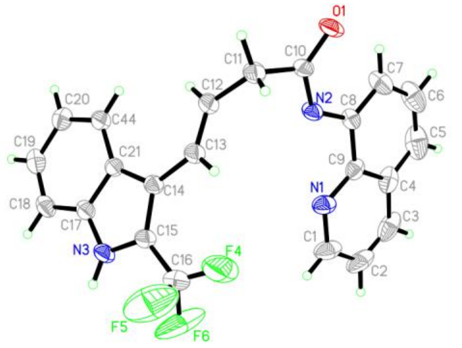

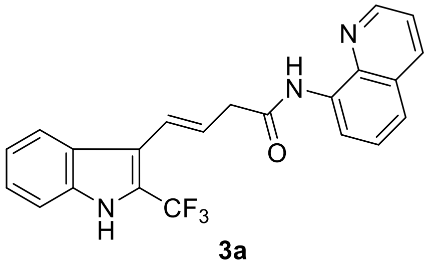

3a

CCDC number: 2144265

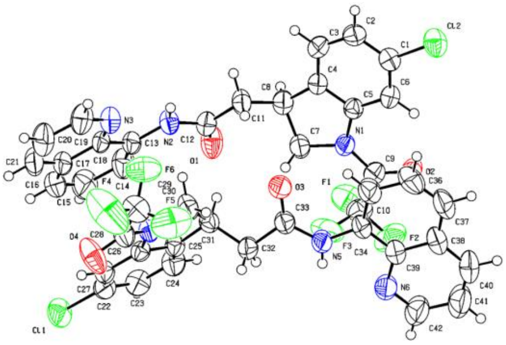

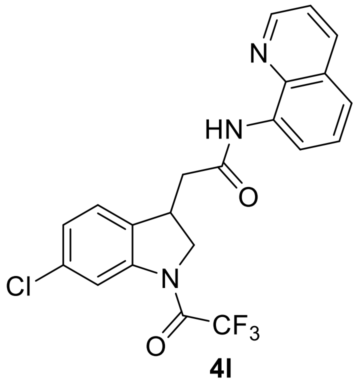

4l

CCDC number: 2144260

6 Characterization Data of the Corresponding Products

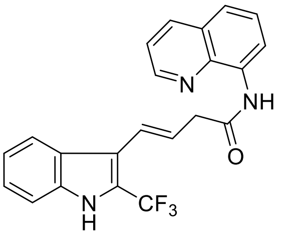

(*E*)-*N*-(quinolin-8-yl)-4-(2-(trifluoromethyl)-1*H*-indol-3-yl)but-3-enamide (**3a**)

Upon completion the mixture was concentrated and purified via flash column chromatography (petroleum ether / ethyl acetate = 5:1, Rf = 0.4) to give the titled product **3a** as a brown solid (45.8 mg, 58%).

^{1}H NMR (400 MHz, d$_6$-DMSO) δ 12.32 (s, 1H), 10.34 (s, 1H), 8.89 (d, *J* = 5.8 Hz, 1H), 8.69 (d, *J* = 8.7 Hz, 1H), 8.43 (d, *J* = 9.8 Hz, 1H), 8.07 (d, *J* = 8.2 Hz, 1H), 7.70 (d, *J* = 9.3 Hz, 1H), 7.67 – 7.65 (m, 1H), 7.63 (d, *J* = 8.2 Hz, 1H), 7.53 (d, *J* = 8.2 Hz, 1H), 7.37 (t, *J* = 7.5 Hz, 1H), 7.25 (t, *J* = 7.6 Hz, 1H), 6.93 (d, *J* = 17.5 Hz, 1H), 6.69 – 6.61 (m, 1H), 3.67 (d, *J* = 7.1 Hz, 2H).

^{13}C NMR (101 MHz, d$_6$-DMSO) δ 170.1, 149.3, 138.5, 137.1, 136.5, 134.9, 128.3, 127.5, 126.28, 125.1, 125.0, 123.7, 122.6, 122.5 (C-F, q, $J_{(C-F)}$ = 269.4 Hz), 122.4, 121.7, 121.6 (C-F, q, $J_{(C-F)}$ = 36.0 Hz), 121.6, 117.0, 114.5, 113.2, 42.2.

^{19}F NMR (377 MHz, d$_6$-DMSO) δ -56.1.

M.p. 112.3 - 113.7 ^{o}C

HRMS (ESI): [M+H]$^+$ calcd. for $C_{22}H_{17}F_3N_3O^+$ 396.1318, found 396.1320.

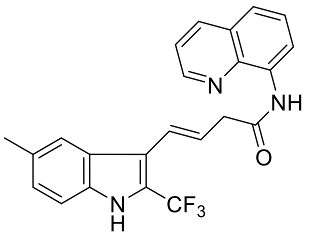

(*E*)-4-(5-methyl-2-(trifluoromethyl)-1*H*-indol-3-yl)-*N*-(quinolin-8-yl)but-3-enamide (**3b**)

Upon completion the mixture was concentrated and purified via flash column chromatography (petroleum ether / ethyl acetate = 5:1, Rf = 0.3) to give the titled product **3b** as a brown solid (37.6 mg, 46%).

^{1}H NMR (400 MHz, d$_6$-DMSO) δ 12.14 (s, 1H), 10.30 (s, 1H), 8.85 (d, *J* = 2.8 Hz, 1H), 8.67 (d, *J* = 7.3 Hz, 1H), 8.41 (d, *J* = 7.2 Hz, 1H), 7.81 (s, 1H), 7.68 (d, *J* = 7.7 Hz, 1H), 7.65 – 7.58 (m, 2H), 7.40 (d, *J* = 8.4 Hz, 1H), 7.18 (d, *J* = 8.4 Hz, 1H), 6.90 (d, *J* = 15.9 Hz, 1H), 6.68 – 6.54 (m, 1H), 3.65 (d, *J* = 7.0 Hz, 2H), 2.41 (s, 3H).

^{13}C NMR (101 MHz, d$_6$-DMSO) δ 170.1, 149.3, 138.6, 137.1, 134.9, 134.9, 130.4, 128.3, 127.5, 126.9, 125.8, 125.3, 124.0, 122.6, 122.4, 122.0 (C-F, q, $J_{(C-F)}$ = 283.9 Hz), 121.7 (C-F, q, $J_{(C-F)}$ = 36.5 Hz), 121.1, 117.0, 114.0, 112.9, 42.3, 21.8.

^{19}F NMR (377 MHz, d$_6$-DMSO) δ -55.9.

M.p. 181.4 - 182.7 ^{o}C

HRMS (ESI): [M+H]$^+$ calcd. for $C_{23}H_{19}F_3N_3O^+$ 410.1475, found 410.1474.

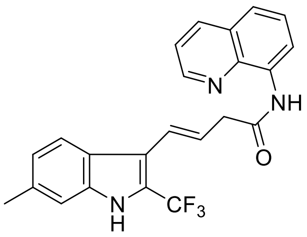

(E)-4-(6-methyl-2-(trifluoromethyl)-1H-indol-3-yl)-N-(quinolin-8-yl)but-3-enamide (**3c**)

Upon completion the mixture was concentrated and purified via flash column chromatography (petroleum ether / ethyl acetate = 5:1, Rf = 0.4) to give the titled product **3c** as a brown solid (41.6 mg, 51%).

^{1}H NMR (400 MHz, d$_6$-DMSO) δ 12.10 (s, 1H), 10.30 (s, 1H), 8.87 (d, J = 2.6 Hz, 1H), 8.67 (d, J = 8.7 Hz, 1H), 8.41 (d, J = 9.9 Hz, 1H), 7.93 (d, J = 8.3 Hz, 1H), 7.68 (d, J = 9.4 Hz, 1H), 7.65 – 7.58 (m, 2H), 7.29 (s, 1H), 7.06 (d, J = 9.3 Hz, 1H), 6.90 (d, J = 17.5 Hz, 1H), 6.66 – 6.55 (m, 1H), 3.64 (d, J = 7.0 Hz, 2H), 2.44 (s, 3H).

^{13}C NMR (101 MHz, d$_6$-DMSO) δ 170.1, 149.3, 138.6, 137.1, 137.0, 134.9, 134.6, 128.3, 127.5, 126.0, 123.9, 123.5, 123.0, 122.6, 122.4, 122.3 (C-F, q, $J_{(C-F)}$ = 280.5 Hz), 121.4, 121.0 (C-F, q, $J_{(C-F)}$ = 36.1 Hz), 117.0, 114.4, 112.7, 42.2, 21.8.

^{19}F NMR (377 MHz, d$_6$-DMSO) δ -55.8.

M.p. 98.9 - 99.7 ^{o}C

HRMS (ESI): $[M+H]^+$ calcd. for $C_{23}H_{19}F_3N_3O^+$ 410.1475, found 410.1477.

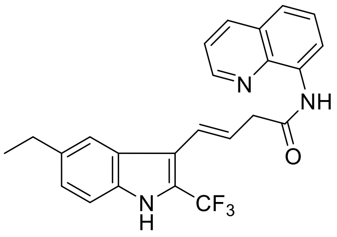

(E)-4-(5-ethyl-2-(trifluoromethyl)-1H-indol-3-yl)-N-(quinolin-8-yl)but-3-enamide (**3d**)

Upon completion the mixture was concentrated and purified via flash column chromatography (petroleum ether / ethyl acetate = 5:1, Rf = 0.3) to give the titled product **3d** as a white solid (40.5 mg, 48%).

^{1}H NMR (400 MHz, d$_6$-DMSO) δ 12.16 (s, 1H), 10.31 (s, 1H), 8.85 (d, J = 5.8 Hz, 1H), 8.68 (d, J = 8.7 Hz, 1H), 8.41 (d, J = 9.9 Hz, 1H), 7.82 (s, 1H), 7.68 (d, J = 9.4 Hz, 1H), 7.65 – 7.58 (m, 2H),

7.44 (d, *J* = 8.4 Hz, 1H), 7.22 (d, *J* = 9.4 Hz, 1H), 6.92 (d, *J* = 17.4 Hz, 1H), 6.68 – 6.57 (m, 1H), 3.66 (d, *J* = 7.0 Hz, 2H), 2.71 (q, *J* = 7.5 Hz, 2H), 1.21 (t, *J* = 7.6 Hz, 3H).

^{13}C NMR (101 MHz, d$_6$-DMSO) δ 170.1, 149.2, 138.6, 137.1, 135.1, 135.0, 128.3, 127.5, 125.9, 125.8, 125.3, 124.0, 122.6, 122.6 (C-F, q, $J_{(C-F)}$ = 269.4 Hz), 122.4, 121.7 (C-F, q, $J_{(C-F)}$ = 36.2 Hz), 119.9, 117.0, 114.1, 113.0, 42.3, 29.0, 16.9.

^{19}F NMR (377 MHz, d$_6$-DMSO) δ -55.9.

M.p. 148.5 - 149.6 ^{o}C

HRMS (ESI): [M+H]$^+$ calcd. for $C_{24}H_{21}F_3N_3O^+$ 424.1631, found 424.1631.

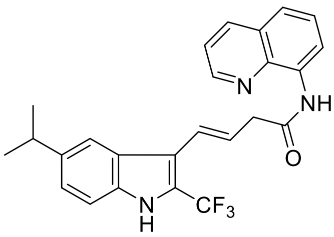

(*E*)-4-(5-isopropyl-2-(trifluoromethyl)-1*H*-indol-3-yl)-*N*-(quinolin-8-yl)but-3-enamide (**3e**)

Upon completion the mixture was concentrated and purified via flash column chromatography (petroleum ether / ethyl acetate = 5:1, Rf = 0.3) to give the titled product **3e** as a brown solid (35.4 mg, 41%).

^{1}H NMR (400 MHz, d$_6$-DMSO) δ 12.15 (s, 1H), 10.31 (s, 1H), 8.84 (d, *J* = 5.9 Hz, 1H), 8.68 (d, *J* = 8.7 Hz, 1H), 8.41 (d, *J* = 9.9 Hz, 1H), 7.81 (s, 1H), 7.68 (d, *J* = 9.5 Hz, 1H), 7.65 – 7.58 (m, 2H), 7.44 (d, *J* = 8.5 Hz, 1H), 7.27 (d, *J* = 9.7 Hz, 1H), 6.92 (d, *J* = 14.7 Hz, 1H), 6.68 – 6.56 (m, 1H), 3.67 (d, *J* = 7.0 Hz, 2H), 3.05 – 2.93 (m, 1H), 1.24 (d, *J* = 6.9 Hz, 6H).

^{13}C NMR (101 MHz, d$_6$-DMSO) δ 170.1, 149.3, 141.8, 138.6, 137.1, 135.2, 135.0, 128.3, 127.5, 126.0, 125.2, 124.3, 123.9, 122.6, 122.6 (C-F, q, $J_{(C-F)}$ = 268.8 Hz), 122.4, 121.7 (C-F, q, $J_{(C-F)}$ = 36.1 Hz), 118.3, 116.9, 114.3, 113.1, 42.3, 34.2, 24.9.

^{19}F NMR (377 MHz, d$_6$-DMSO) δ -56.0.

M.p. 126.6 - 128.0 ^{o}C

HRMS (ESI): [M+H]$^+$ calcd. for $C_{25}H_{23}F_3N_3O^+$ 438.1788, found 438.1790.

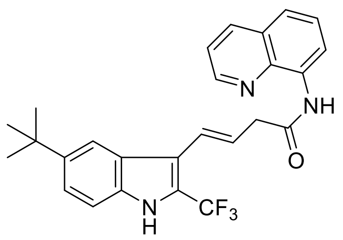

(E)-4-(5-(*tert*-butyl)-2-(trifluoromethyl)-1H-indol-3-yl)-N-(quinolin-8-yl)but-3-enamide (**3f**)

Upon completion the mixture was concentrated and purified via flash column chromatography (petroleum ether / ethyl acetate = 5:1, Rf = 0.4) to give the titled product **3f** as a brown oily liquid (38.3 mg, 43%).

^{1}H NMR (400 MHz, d$_6$-DMSO) δ 12.14 (s, 1H), 10.32 (s, 1H), 8.83 (d, J = 5.8 Hz, 1H), 8.69 (d, J = 8.7 Hz, 1H), 8.41 (d, J = 9.9 Hz, 1H), 7.91 (s, 1H), 7.65 – 7.59 (m, 2H), 7.45 (d, J = 1.0 Hz, 2H), 6.93 (dd, J = 16.1, 1.4 Hz, 1H), 6.67 – 6.53 (m, 1H), 3.68 (d, J = 7.0 Hz, 2H), 1.31 (s, 9H).

^{13}C NMR (101 MHz, d$_6$-DMSO) δ 170.1, 149.3, 143.9, 138.6, 137.1, 135.0, 134.7, 128.3, 127.5, 126.2, 125.0, 123.9, 123.6, 122.6, 122.6 (C-F, q, $J_{(C-F)}$ = 269.1 Hz), 122.3, 121.6 (C-F, q, $J_{(C-F)}$ = 36.2 Hz), 116.9, 116.8, 114.5, 112.7, 42.3, 34.9, 32.0.

^{19}F NMR (377 MHz, d$_6$-DMSO) δ -56.0.

HRMS (ESI): $[M+H]^+$ calcd. for $C_{26}H_{25}F_3N_3O^+$ 452.1944, found 452.1946.

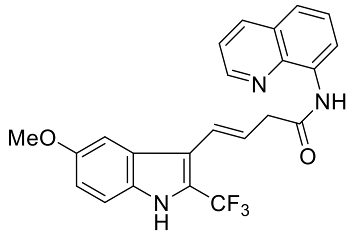

(E)-4-(5-methoxy-2-(trifluoromethyl)-1H-indol-3-yl)-N-(quinolin-8-yl)but-3-enamide (**3g**)

Upon completion the mixture was concentrated and purified via flash column chromatography (petroleum ether / ethyl acetate = 5:1, Rf = 0.4) to give the titled product **3g** as a white solid (38.0 mg, 45%).

^{1}H NMR (400 MHz, d$_6$-DMSO) δ 12.16 (s, 1H), 10.30 (s, 1H), 8.85 (d, J = 5.8 Hz, 1H), 8.68 (d, J = 8.5 Hz, 1H), 8.41 (d, J = 9.8 Hz, 1H), 7.68 (d, J = 9.3 Hz, 1H), 7.65 – 7.57 (m, 2H), 7.47 – 7.39 (m, 2H), 7.03 (d, J = 11.2 Hz, 1H), 6.91 (d, J = 17.3 Hz, 1H), 6.64 – 6.53 (m, 1H), 3.78 (s, 3H), 3.65 (d, J = 7.1 Hz, 2H).

^{13}C NMR (101 MHz, d$_6$-DMSO) δ 170.1, 155.2, 149.3, 138.6, 137.1, 134.9, 131.6, 128.3, 127.5, 125.7, 125.6, 124.0, 122.6, 122.5 (C-F, q, $J_{(C\text{-}F)}$ = 269.0 Hz), 122.4, 122.0 (C-F, q, $J_{(C\text{-}F)}$ = 36.3 Hz), 116.9, 115.9, 114.1, 102.8, 55.9, 42.3.

^{19}F NMR (377 MHz, d$_6$-DMSO) δ -56.1.

M.p. 175.2 - 177.1 ^{o}C

HRMS (ESI): $[M+H]^+$ calcd. for $C_{23}H_{19}F_3N_3O_2^+$ 426.1424, found 426.1424.

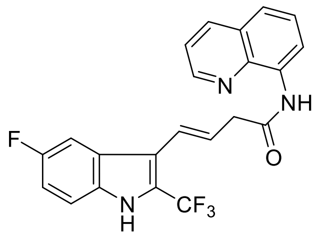

(*E*)-4-(5-fluoro-2-(trifluoromethyl)-1*H*-indol-3-yl)-*N*-(quinolin-8-yl)but-3-enamide (**3h**)

Upon completion the mixture was concentrated and purified via flash column chromatography (petroleum ether / ethyl acetate = 5:1, Rf = 0.4) to give the titled product **3h** as a white solid (24.3 mg, 30%).

^{1}H NMR (400 MHz, d$_6$-DMSO) δ 12.44 (s, 1H), 10.30 (s, 1H), 8.87 (d, *J* = 2.6 Hz, 1H), 8.68 (d, *J* = 6.6 Hz, 1H), 8.42 (d, *J* = 6.7 Hz, 1H), 7.81 (d, *J* = 12.5 Hz, 1H), 7.69 (d, *J* = 7.1 Hz, 1H), 7.67 – 7.58 (m, 2H), 7.57 – 7.51 (m, 1H), 7.25 (t, *J* = 7.9 Hz, 1H), 6.89 (d, *J* = 16.1 Hz, 1H), 6.65 – 6.54 (m, 1H), 3.64 (d, *J* = 7.1 Hz, 2H).

^{13}C NMR (101 MHz, d$_6$-DMSO) δ 170.0, 158.4 (C-F, d, $J_{(C\text{-}F)}$ = 234.5 Hz), 149.2, 138.6, 137.1, 134.9, 133.2, 128.3, 127.5, 126.6, 125.2 (C-F, d, $J_{(C\text{-}F)}$ = 10.2 Hz), 123.4, 122.9 (C-F, q, $J_{(C\text{-}F)}$ = 36.8 Hz), 122.6, 122.4, 122.2 (C-F, q, $J_{(C\text{-}F)}$ = 269.7 Hz), 117.0, 114.7, 114.6, 114.0 (C-F, d, $J_{(C\text{-}F)}$ = 26.6 Hz), 106.3 (C-F, d, $J_{(C\text{-}F)}$ = 24.0 Hz), 42.2.

^{19}F NMR (377 MHz, d$_6$-DMSO) δ -121.8, -56.5.

M.p. 184.3 - 186.2 ^{o}C

HRMS (ESI): $[M+H]^+$ calcd. for $C_{22}H_{16}F_4N_3O^+$ 414.1224, found 414.1222.

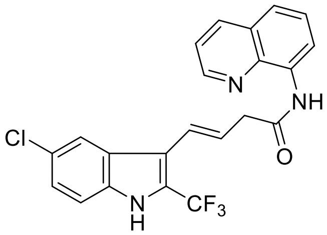

(E)-4-(5-chloro-2-(trifluoromethyl)-1H-indol-3-yl)-N-(quinolin-8-yl)but-3-enamide (**3i**)

Upon completion the mixture was concentrated and purified via flash column chromatography (petroleum ether / ethyl acetate = 5:1, Rf = 0.4) to give the titled product **3i** as a brown solid (24.2 mg, 29%).

^{1}H NMR (400 MHz, d$_6$-DMSO) δ 12.53 (s, 1H), 10.30 (s, 1H), 8.86 (d, J = 2.6 Hz, 1H), 8.67 (d, J = 6.5 Hz, 1H), 8.42 (d, J = 9.9 Hz, 1H), 8.05 (d, J = 1.7 Hz, 1H), 7.68 (d, J = 7.0 Hz, 1H), 7.65 – 7.58 (m, 2H), 7.54 (d, J = 8.7 Hz, 1H), 7.37 (d, J = 10.7 Hz, 1H), 6.88 (d, J = 16.2 Hz, 1H), 6.66 – 6.55 (m, 1H), 3.65 (d, J = 7.1 Hz, 2H).

^{13}C NMR (101 MHz, d$_6$-DMSO) δ 170.0, 149.3, 138.6, 137.1, 135.0, 134.9, 128.3, 127.5, 127.2, 126.2, 126.1, 125.4, 123.1, 123.0 (C-F, q, $J_{(C-F)}$ = 36.5 Hz), 122.6, 122.4, 121.8 (C-F, q, $J_{(C-F)}$ = 271.5 Hz), 120.8, 117.0, 115.0, 114.3, 42.1.

^{19}F NMR (377 MHz, d$_6$-DMSO) δ -56.5.

M.p. 191.2 - 192.6 ^{o}C

HRMS (ESI): $[M+H]^+$ calcd. for $C_{22}H_{16}ClF_3N_3O^+$ 430.0929, found 430.0928.

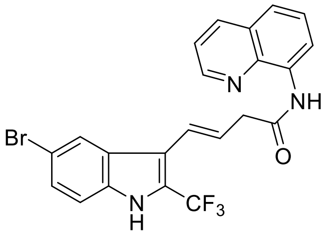

(E)-4-(5-bromo-2-(trifluoromethyl)-1H-indol-3-yl)-N-(quinolin-8-yl)but-3-enamide (**3j**)

Upon completion the mixture was concentrated and purified via flash column chromatography (petroleum ether / ethyl acetate = 5:1, Rf = 0.4) to give the titled product **3j** as a white solid (17.6 mg, 19%).

^{1}H NMR (400 MHz, d$_6$-DMSO) δ 12.53 (s, 1H), 10.30 (s, 1H), 8.86 (d, *J* = 5.8 Hz, 1H), 8.66 (d, *J* = 6.5 Hz, 1H), 8.41 (d, *J* = 9.9 Hz, 1H), 8.18 (s, 1H), 7.68 (d, *J* = 7.1 Hz, 1H), 7.65 – 7.57 (m, 2H), 7.48 (d, *J* = 1.0 Hz, 2H), 6.87 (d, *J* = 14.8 Hz, 1H), 6.65 – 6.52 (m, 1H), 3.64 (d, *J* = 7.0 Hz, 2H).

^{13}C NMR (101 MHz, d$_6$-DMSO) δ 170.0, 149.3, 138.6, 137.1, 135.2, 134.9, 128.3, 127.9, 127.5, 127.2, 126.8, 123.8, 123.2 (C-F, q, $J_{(C\text{-}F)}$ = 41.5 Hz), 123.1, 122.6, 122.4, 122.2 (C-F, q, $J_{(C\text{-}F)}$ = 269.4 Hz), 117.0, 115.3, 114.2, 42.1.

^{19}F NMR (377 MHz, d$_6$-DMSO) δ -56.5.

M.p. 146.3 - 148.1 ^{o}C

HRMS (ESI): $[M+H]^+$ calcd. for $C_{22}H_{16}BrF_3N_3O^+$ 474.0423, found 474.0421.

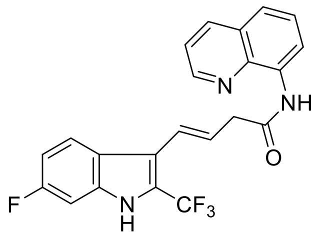

(*E*)-4-(6-fluoro-2-(trifluoromethyl)-1*H*-indol-3-yl)-*N*-(quinolin-8-yl)but-3-enamide (**3k**)

Upon completion the mixture was concentrated and purified via flash column chromatography (petroleum ether / ethyl acetate = 5:1, Rf = 0.4) to give the titled product **3k** as a brown solid (25.5 mg, 31%).

^{1}H NMR (400 MHz, d$_6$-DMSO) δ 12.41 (s, 1H), 10.30 (s, 1H), 8.88 (d, *J* = 5.8 Hz, 1H), 8.67 (d, *J* = 8.6 Hz, 1H), 8.42 (d, *J* = 9.9 Hz, 1H), 8.07 (dd, *J* = 8.9, 5.3 Hz, 1H), 7.69 (d, *J* = 9.4 Hz, 1H), 7.66 – 7.59 (m, 2H), 7.26 (d, *J* = 11.9 Hz, 1H), 7.12 (t, *J* = 9.3 Hz, 1H), 6.90 (d, *J* = 17.5 Hz, 1H), 6.67 – 6.58 (m, 1H), 3.66 (d, *J* = 7.0 Hz, 2H).

^{13}C NMR (101 MHz, d$_6$-DMSO) δ 170.0, 160.8 (C-F, d, $J_{(C\text{-}F)}$ = 239.4 Hz), 149.3, 138.6, 137.1, 136.7 (C-F, d, $J_{(C\text{-}F)}$ = 12.7 Hz), 134.9, 128.3, 127.5, 126.9, 123.4, 123.2 (C-F, q, $J_{(C\text{-}F)}$ = 42.6 Hz), 122.6, 122.4, 122.3 (C-F, q, $J_{(C\text{-}F)}$ = 269.1 Hz), 122.0, 117.0, 114.9, 110.6 (C-F, d, $J_{(C\text{-}F)}$ = 24.7 Hz), 98.9 (C-F, d, $J_{(C\text{-}F)}$ = 25.7 Hz), 42.1.

^{19}F NMR (377 MHz, d$_6$-DMSO) δ -117.0, -56.2.

M.p. 140.2 - 141.9 ^{o}C

HRMS (ESI): $[M+H]^+$ calcd. for $C_{22}H_{16}F_4N_3O^+$ 414.1224, found 414.1224.

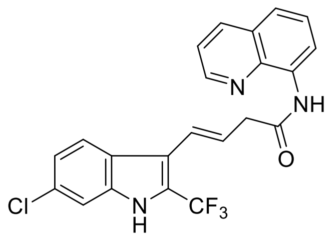

(*E*)-4-(6-chloro-2-(trifluoromethyl)-1*H*-indol-3-yl)-*N*-(quinolin-8-yl)but-3-enamide (**3l**)

Upon completion the mixture was concentrated and purified via flash column chromatography (petroleum ether / ethyl acetate = 5:1, Rf = 0.4) to give the titled product **3l** as a white solid (24.7 mg, 29%).

^{1}H NMR (400 MHz, d$_6$-DMSO) δ 12.47 (s, 1H), 10.29 (s, 1H), 8.87 (d, *J* = 5.9 Hz, 1H), 8.66 (d, *J* = 6.5 Hz, 1H), 8.41 (d, *J* = 6.7 Hz, 1H), 8.05 (d, *J* = 8.7 Hz, 1H), 7.68 (d, *J* = 7.0 Hz, 1H), 7.65 – 7.57 (m, 2H), 7.52 (d, *J* = 1.8 Hz, 1H), 7.25 (d, *J* = 10.6 Hz, 1H), 6.88 (d, *J* = 17.6 Hz, 1H), 6.67 – 6.54 (m, 1H), 3.65 (d, *J* = 7.0 Hz, 2H).

^{13}C NMR (101 MHz, d$_6$-DMSO) δ 169.9, 149.3, 138.6, 137.1, 136.9, 134.9, 129.8, 128.3, 127.5, 127.2, 123.9, 123.3, 123.2, 122.7, 122.4, 122.3 (C-F, q, $J_{(C\text{-}F)}$ = 269.3 Hz), 122.1 (C-F, q, $J_{(C\text{-}F)}$ = 36.5 Hz), 122.1, 117.0, 114.8, 112.7, 42.1.

^{19}F NMR (377 MHz, d$_6$-DMSO) δ -56.4.

M.p. 198.9 - 200.6 ^{o}C

HRMS (ESI): $[M+H]^+$ calcd. for $C_{22}H_{16}ClF_3N_3O^+$ 430.0929, found 430.0931.

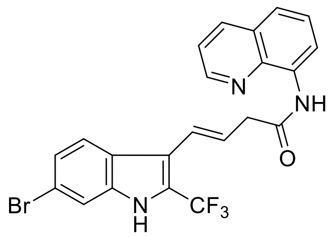

(*E*)-4-(6-bromo-2-(trifluoromethyl)-1*H*-indol-3-yl)-*N*-(quinolin-8-yl)but-3-enamide (**3m**)

Upon completion the mixture was concentrated and purified via flash column chromatography (petroleum ether / ethyl acetate = 5:1, Rf = 0.4) to give the titled product **3m** as a white solid (17.4 mg, 19%).

^{1}H NMR (400 MHz, d$_6$-DMSO) δ 12.47 (s, 1H), 10.29 (s, 1H), 8.87 (d, *J* = 5.8 Hz, 1H), 8.65 (d, *J* = 8.7 Hz, 1H), 8.41 (d, *J* = 9.9 Hz, 1H), 7.99 (d, *J* = 8.7 Hz, 1H), 7.68 (d, *J* = 7.2 Hz, 1H), 7.67 – 7.57 (m, 3H), 7.36 (d, *J* = 10.5 Hz, 1H), 6.88 (d, *J* = 16.1 Hz, 1H), 6.64 – 6.55 (m, 1H), 3.65 (d, *J* = 7.0 Hz, 2H).

^{13}C NMR (101 MHz, d$_6$-DMSO) δ 170.0, 149.3, 138.6, 137.3, 137.1, 134.9, 128.3, 127.5, 127.2, 124.6, 124.1, 123.6, 123.1, 122.7, 122.4, 122.3 (C-F, q, $J_{(C\text{-}F)}$ = 36.5 Hz), 122.2 (C-F, q, $J_{(C\text{-}F)}$ = 269.1 Hz), 117.9, 117.0, 115.7, 42.1.

^{19}F NMR (377 MHz, d$_6$-DMSO) δ -56.4.

M.p. 198.9 - 200.7 ^{o}C

HRMS (ESI): [M+H]$^+$ calcd. for $C_{22}H_{16}BrF_3N_3O^+$ 474.0423, found 474.0418.

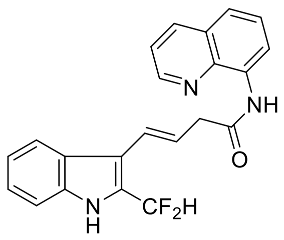

(*E*)-4-(2-(difluoromethyl)-1*H*-indol-3-yl)-*N*-(quinolin-8-yl)but-3-enamide (**3n**)

Upon completion the mixture was concentrated and purified via flash column chromatography (petroleum ether / ethyl acetate = 5:1, Rf = 0.2) to give the titled product **3n** as a brown solid (17.0 mg, 23%).

^{1}H NMR (400 MHz, d$_6$-DMSO) δ 11.91 (s, 1H), 10.30 (s, 1H), 8.86 (d, *J* = 5.9 Hz, 1H), 8.66 (d, *J* = 8.7 Hz, 1H), 8.42 (d, *J* = 9.9 Hz, 1H), 7.98 (d, *J* = 8.1 Hz, 1H), 7.68 (d, *J* = 8.3 Hz, 1H), 7.65 – 7.62 (m, 1H), 7.59 (d, *J* = 8.0 Hz, 1H), 7.47 (d, *J* = 9.8 Hz, 1H), 7.30 (dd, *J* = 15.3, 7.8 Hz, 1H), 7.17 (t, *J* = 7.2 Hz, 1H), 7.01 (d, *J* = 16.0 Hz, 1H), 6.55 – 6.44 (m, 1H), 3.60 (d, *J* = 7.1 Hz, 2H).

^{13}C NMR (101 MHz, d$_6$-DMSO) δ 170.2, 149.3, 138.6, 137.1, 136.8, 134.9, 128.3, 127.5, 127.5 (C-F, t, $J_{(C\text{-}F)}$ = 23.0 Hz), 125.2, 124.8, 124.3, 124.0, 122.7, 122.4, 121.3, 120.9, 116.9, 114.3 (C-F, t, $J_{(C\text{-}F)}$ = 246.6 Hz), 112.9, 110.8, 42.4.

^{19}F NMR (377 MHz, d$_6$-DMSO) δ -109.1.

M.p. 182.3 - 183.7 ^{o}C

HRMS (ESI): [M+H]$^+$ calcd. for $C_{22}H_{18}F_2N_3O^+$ 378.1412, found 378.1411.

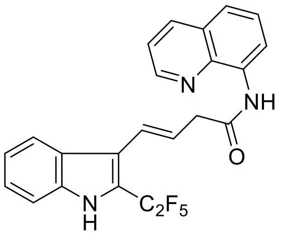

(*E*)-4-(2-(perfluoroethyl)-1*H*-indol-3-yl)-*N*-(quinolin-8-yl)but-3-enamide (**3o**)

Upon completion the mixture was concentrated and purified via flash column chromatography (petroleum ether / ethyl acetate = 5:1, Rf = 0.4) to give the titled product **3o** as a brown solid (38.1 mg, 43%).

^{1}H NMR (400 MHz, d$_6$-DMSO) δ 12.24 (s, 1H), 10.30 (s, 1H), 8.87 (d, *J* = 5.8 Hz, 1H), 8.68 (d, *J* = 8.6 Hz, 1H), 8.42 (d, *J* = 9.9 Hz, 1H), 8.09 (d, *J* = 8.2 Hz, 1H), 7.69 (d, *J* = 7.1 Hz, 1H), 7.66 – 7.59 (m, 2H), 7.54 (d, *J* = 8.3 Hz, 1H), 7.37 (t, *J* = 7.4 Hz, 1H), 7.24 (t, *J* = 7.6 Hz, 1H), 6.88 (d, *J* = 16.1 Hz, 1H), 6.70 – 6.60 (m, 1H), 3.65 (d, *J* = 7.0 Hz, 2H).

^{13}C NMR (101 MHz, d$_6$-DMSO) δ 170.1, 149.2, 138.6, 137.3, 137.1, 134.9, 128.3, 127.5, 126.6, 125.3, 125.2, 124.0, 122.6, 122.4, 121.7, 121.6, 119.4 (C-F, t, $J_{(C\text{-}F)}$ = 27.9 Hz), 117.0, 116.5, 113.2, 42.2.

^{19}F NMR (377 MHz, d$_6$-DMSO) δ -110.1, -83.8.

M.p. 96.4 - 98.4 ^{o}C

HRMS (ESI): [M+H]$^+$ calcd. for $C_{23}H_{17}F_5N_3O^+$ 446.1286, found 446.1286.

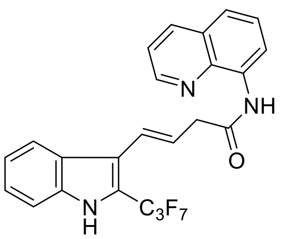

(E)-4-(2-(perfluoropropyl)-1H-indol-3-yl)-N-(quinolin-8-yl)but-3-enamide (**3p**)

Upon completion the mixture was concentrated and purified via flash column chromatography (petroleum ether / ethyl acetate = 5:1, Rf = 0.3) to give the titled product **3p** as a brown solid (34.5 mg, 35%).

^{1}H NMR (400 MHz, d$_6$-DMSO) δ 12.25 (s, 1H), 10.29 (s, 1H), 8.86 (d, J = 5.9 Hz, 1H), 8.68 (d, J = 8.7 Hz, 1H), 8.41 (d, J = 9.9 Hz, 1H), 8.09 (d, J = 8.2 Hz, 1H), 7.68 (d, J = 7.0 Hz, 1H), 7.65 – 7.58 (m, 2H), 7.54 (d, J = 8.3 Hz, 1H), 7.36 (t, J = 7.6 Hz, 1H), 7.23 (t, J = 8.0 Hz, 1H), 6.86 (d, J = 16.1 Hz, 1H), 6.72 – 6.58 (m, 1H), 3.65 (d, J = 7.0 Hz, 2H).

^{13}C NMR (101 MHz, d$_6$-DMSO) δ 170.1, 149.2, 138.6, 137.4, 137.1, 134.9, 128.3, 127.5, 126.8, 125.3, 125.2, 124.0, 122.6, 122.4, 121.8, 121.6, 119.3 (C-F, t, $J_{(C\text{-}F)}$ = 28.3 Hz), 117.0, 116.9, 113.2, 42.2.

^{19}F NMR (377 MHz, d$_6$-DMSO) δ -125.8, -107.0, -79.7.

M.p. 94.8 - 96.4 ^{o}C

HRMS (ESI): $[M+H]^+$ calcd. for $C_{24}H_{17}F_7N_3O^+$ 496.1254, found 496.1260.

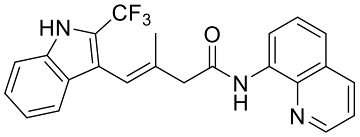

(E)-3-methyl-N-(quinolin-8-yl)-4-(2-(trifluoromethyl)-1H-indol-3-yl)but-3-enamide (**3q**)

Upon completion the mixture was concentrated and purified via flash column chromatography (petroleum ether / ethyl acetate = 5:1, Rf = 0.3) to give the titled product **3q** as a white solid (35.3 mg, 43%).

^{1}H NMR (400 MHz, d$_6$-DMSO) δ 12.24 (s, 1H), 9.94 (s, 1H), 8.87 (d, J = 5.8 Hz, 1H), 8.60 (d, J = 8.4 Hz, 1H), 8.40 (d, J = 9.9 Hz, 1H), 7.69 – 7.62 (m, 3H), 7.58 (t, J = 7.9 Hz, 1H), 7.49 (d, J = 8.3 Hz, 1H), 7.28 (t, J = 7.5 Hz, 1H), 7.10 (t, J = 7.2 Hz, 1H), 6.55 (s, 1H), 3.36 (s, 2H), 2.09 (s, 3H).

^{13}C NMR (101 MHz, d$_6$-DMSO) δ 169.2, 149.2, 138.4, 137.0, 136.2, 134.7, 128.2, 127.4, 126.4, 124.99, 122.6 (C-F, q, $J_{(C-F)}$ = 266.8 Hz), 122.6, 122.3, 122.1 (C-F, q, $J_{(C-F)}$ = 36.1 Hz), 121.4, 120.9, 118.4, 116.8, 114.0, 112.9, 42.5, 23.6.

^{19}F NMR (377 MHz, d$_6$-DMSO) δ -57.0.

M.p. 201.8 - 203.6 ^{o}C

HRMS (ESI): $[M+H]^+$ calcd. for $C_{23}H_{19}F_3N_3O^+$ 410.1475, found 410.1476.

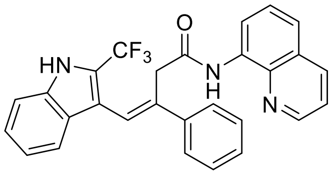

(*E*)-3-phenyl-*N*-(quinolin-8-yl)-4-(2-(trifluoromethyl)-1*H*-indol-3-yl)but-3-enamide (**3r**)

Upon completion the mixture was concentrated and purified via flash column chromatography (petroleum ether / ethyl acetate = 5:1, Rf = 0.3) to give the titled product **3r** as a brown solid (32.8 mg, 35%).

^{1}H NMR (400 MHz, d$_6$-DMSO) δ 12.18 (s, 1H), 10.41 (s, 1H), 8.82 (d, *J* = 5.7 Hz, 1H), 8.61 (d, *J* = 7.6 Hz, 1H), 8.41 (d, *J* = 9.7 Hz, 1H), 7.67 – 7.62 (m, 2H), 7.56 (t, *J* = 8.0 Hz, 1H), 7.39 (d, *J* = 8.2 Hz, 1H), 7.31 (d, *J* = 6.9 Hz, 2H), 7.16 (dd, *J* = 17.3, 7.9 Hz, 2H), 7.10 – 7.02 (m, 4H), 6.86 (t, *J* = 7.5 Hz, 1H), 4.03 (s, 2H).

^{13}C NMR (101 MHz, d$_6$-DMSO) δ 169.3, 149.2, 139.9, 139.2, 138.4, 137.1, 136.2, 134.7, 128.7, 128.5, 128.2, 127.7, 127.5, 125.3, 124.6, 122.7, 122.4 (C-F, q, $J_{(C-F)}$ = 269.5 Hz), 122.3, 122.0 (C-F, q, $J_{(C-F)}$ = 36.4 Hz), 121.6, 121.6, 120.4, 116.3, 114.5, 112.6, 47.9.

^{19}F NMR (377 MHz, d$_6$-DMSO) δ -57.1.

M.p. 219.4 - 220.6 ^{o}C

HRMS (ESI): $[M+H]^+$ calcd. for $C_{28}H_{21}F_3N_3O^+$ 472.1631, found 472.1634.

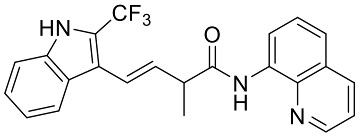

(*E*)-2-methyl-*N*-(quinolin-8-yl)-4-(2-(trifluoromethyl)-1*H*-indol-3-yl)but-3-enamide (**3s**)

Upon completion the mixture was concentrated and purified via flash column chromatography (petroleum ether / ethyl acetate = 5:1, Rf = 0.4) to give the titled product **3s** as a white solid (40.7 mg, 50%).

^{1}H NMR (400 MHz, d$_6$-DMSO) δ 12.30 (s, 1H), 10.34 (s, 1H), 8.84 (d, *J* = 2.6 Hz, 1H), 8.68 (d, *J* = 8.8 Hz, 1H), 8.41 (d, *J* = 8.3 Hz, 1H), 8.04 (d, *J* = 8.2 Hz, 1H), 7.68 (d, *J* = 9.5 Hz, 1H), 7.64 – 7.58 (m, 2H), 7.52 (d, *J* = 8.2 Hz, 1H), 7.35 (t, *J* = 7.5 Hz, 1H), 7.22 (t, *J* = 7.6 Hz, 1H), 6.98 (d, *J* = 16.2 Hz, 1H), 6.59 (dd, *J* = 16.1, 8.3 Hz, 1H), 3.87 (p, *J* = 7.0 Hz, 1H), 1.44 (d, *J* = 6.9 Hz, 3H).

^{13}C NMR (101 MHz, d$_6$-DMSO) δ 173.0, 149.3, 138.6, 137.1, 136.5, 134.9, 132.7, 128.3, 127.5, 125.1, 125.0, 122.6, 122.6 (C-F, q, $J_{(C\text{-}F)}$ = 269.3 Hz), 122.4, 121.8, 121.8 (C-F, q, $J_{(C\text{-}F)}$ = 36.3 Hz), 121.7, 121.6, 116.9, 114.3, 113.2, 45.7, 18.0.

^{19}F NMR (377 MHz, d$_6$-DMSO) δ -56.0.

M.p. 166.7 - 168.4 ^{o}C

HRMS (ESI): $[M+H]^+$ calcd. for $C_{23}H_{19}F_3N_3O^+$ 410.1475, found 410.1471.

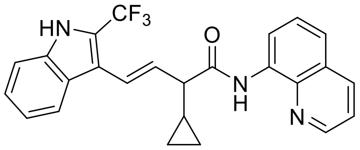

(*E*)-2-cyclopropyl-*N*-(quinolin-8-yl)-4-(2-(trifluoromethyl)-1*H*-indol-3-yl)but-3-enamide (**3t**)

Upon completion the mixture was concentrated and purified via flash column chromatography (petroleum ether / ethyl acetate = 5:1, Rf = 0.3) to give the titled product **3t** as a brown solid (23.1 mg, 27%).

^{1}H NMR (400 MHz, d$_6$-DMSO) δ 12.27 (s, 1H), 10.38 (s, 1H), 8.92 (d, *J* = 2.9 Hz, 1H), 8.71 (d, *J* = 7.5 Hz, 1H), 8.44 (d, *J* = 8.3 Hz, 1H), 8.00 (d, *J* = 8.2 Hz, 1H), 7.70 (d, *J* = 7.8 Hz, 1H), 7.64 (dt, *J* = 16.1, 6.1 Hz, 2H), 7.52 (d, *J* = 8.2 Hz, 1H), 7.36 (t, *J* = 7.6 Hz, 1H), 7.24 (t, *J* = 7.5 Hz, 1H), 6.93 (d,

J = 16.2 Hz, 1H), 6.62 (dd, *J* = 16.1, 8.1 Hz, 1H), 3.15 (t, *J* = 8.6 Hz, 1H), 1.43 – 1.33 (m, 1H), 0.67 (q, *J* = 5.5 Hz, 2H), 0.58 (dd, *J* = 8.6, 3.7 Hz, 1H), 0.37 (dd, *J* = 8.6, 3.6 Hz, 1H).

^{13}C NMR (101 MHz, d$_6$-DMSO) δ 172.4, 149.4, 138.6, 137.1, 136.5, 134.9, 130.9, 128.3, 127.5, 125.1, 125.0, 122.7, 122.6 (C-F, q, $J_{(C-F)}$ = 269.0 Hz), 122.4, 121.9, 121.9 (C-F, q, $J_{(C-F)}$ = 38.6 Hz), 121.6, 121.6, 116.9, 114.5, 113.2, 55.5, 14.3, 4.6, 4.1.

^{19}F NMR (377 MHz, d$_6$-DMSO) δ -56.1.

M.p. 156.8 - 158.4 ^{o}C

HRMS (ESI): $[M+H]^+$ calcd. for $C_{25}H_{21}F_3N_3O^+$ 436.1631, found 436.1633.

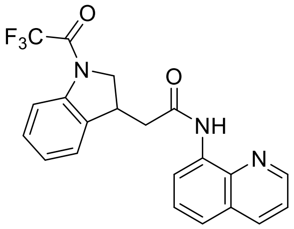

N-(quinolin-8-yl)-2-(1-(2,2,2-trifluoroacetyl)indolin-3-yl)acetamide (**4a**)

Upon completion the mixture was concentrated and purified via flash column chromatography (petroleum ether / ethyl acetate = 5:1, Rf = 0.4) to give the titled product **4a** as a white solid (51.5 mg, 65%).

^{1}H NMR (400 MHz, d$_6$-DMSO) δ 10.27 (s, 1H), 8.94 (d, *J* = 5.5 Hz, 1H), 8.67 (d, *J* = 7.5 Hz, 1H), 8.42 (d, *J* = 9.6 Hz, 1H), 8.10 (d, *J* = 8.0 Hz, 1H), 7.70 (d, *J* = 7.9 Hz, 1H), 7.67 – 7.58 (m, 2H), 7.51 (d, *J* = 7.4 Hz, 1H), 7.34 (t, *J* = 7.7 Hz, 1H), 7.24 (t, *J* = 7.4 Hz, 1H), 4.59 (t, *J* = 10.0 Hz, 1H), 4.25 – 4.13 (m, 1H), 4.07 – 3.96 (m, 1H), 3.32 (d, *J* = 4.9 Hz, 1H), 3.06 – 2.96 (m, 1H).

13C NMR (101 MHz, d$_6$-DMSO) δ 170.5, 153.7 (C-F, q, $J_{(C-F)}$ = 36.6 Hz), 149.3, 141.6, 138.7, 137.1, 135.9, 134.9, 128.4, 128.4, 127.4, 126.4, 125.2, 122.6, 117.6, 116.4 (C-F, q, $J_{(C-F)}$ = 288.3 Hz), 54.1, 54.1, 41.6, 37.5.

^{19}F NMR (377 MHz, d$_6$-DMSO) δ -71.3.

M.p. 96.8 - 98.4 ^{o}C

HRMS (ESI): $[M+H]^+$ calcd. for $C_{21}H_{17}F_3N_3O_2^+$ 400.1267, found 400.1270.

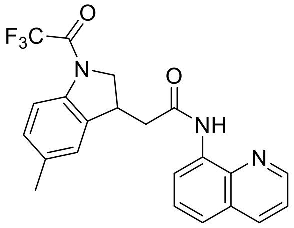

2-(5-methyl-1-(2,2,2-trifluoroacetyl)indolin-3-yl)-*N*-(quinolin-8-yl)acetamide (**4b**)

Upon completion the mixture was concentrated and purified via flash column chromatography (petroleum ether / ethyl acetate = 5:1, Rf = 0.4) to give the titled product **4b** as a white solid (58.8 mg, 71%).

^{1}H NMR (400 MHz, d$_6$-DMSO) δ 10.25 (s, 1H), 8.93 (d, *J* = 5.6 Hz, 1H), 8.65 (d, *J* = 7.5 Hz, 1H), 8.42 (d, *J* = 9.7 Hz, 1H), 7.96 (d, *J* = 8.2 Hz, 1H), 7.69 (d, *J* = 7.4 Hz, 1H), 7.67 – 7.57 (m, 2H), 7.32 (s, 1H), 7.12 (d, *J* = 8.2 Hz, 1H), 4.57 (t, *J* = 10.0 Hz, 1H), 4.21 – 4.13 (m, 1H), 4.01 – 3.91 (m, 1H), 3.36 – 3.27 (m, 1H), 3.02 – 2.92 (m, 1H), 2.29 (s, 3H).

^{13}C NMR (101 MHz, d$_6$-DMSO) δ 170.5, 153.3 (C-F, q, $J_{(C-F)}$ = 36.6 Hz), 149.3, 139.3, 138.7, 137.0, 135.9, 135.8, 134.9, 128.8, 128.3, 127.4, 125.6, 122.6, 117.5, 117.3, 116.4 (C-F, q, $J_{(C-F)}$ = 288.3 Hz), 54.2, 41.5, 37.4, 21.2.

^{19}F NMR (377 MHz, d$_6$-DMSO) δ -71.2.

M.p. 138.4 - 139.8 ^{o}C

HRMS (ESI): $[M+H]^+$ calcd. for $C_{22}H_{19}F_3N_3O_2^+$ 414.1424, found 414.1423.

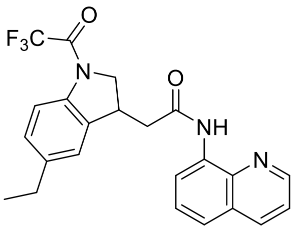

2-(5-ethyl-1-(2,2,2-trifluoroacetyl)indolin-3-yl)-*N*-(quinolin-8-yl)acetamide (**4c**)

Upon completion the mixture was concentrated and purified via flash column chromatography (petroleum ether / ethyl acetate = 5:1, Rf = 0.4) to give the titled product **4c** as a brown solid (52.6 mg, 62%).

^{1}H NMR (400 MHz, CDCl$_3$) δ 9.89 (s, 1H), 8.83 – 8.79 (m, 2H), 8.21 (d, *J* = 9.8 Hz, 1H), 8.16 (d, *J* = 8.3 Hz, 1H), 7.58 (q, *J* = 6.4 Hz, 2H), 7.52 – 7.48 (m, 1H), 7.20 – 7.15 (m, 2H), 4.66 (t, *J* = 9.6 Hz, 1H), 4.18 – 4.08 (m, 2H), 3.19 – 3.11 (m, 1H), 2.95 – 2.84 (m, 1H), 2.64 (q, *J* = 7.6 Hz, 2H), 1.21 (t, *J* = 7.6 Hz, 3H).

^{13}C NMR (101 MHz, CDCl$_3$) δ 168.9, 154.1 (C-F, q, $J_{(C-F)}$ = 37.4 Hz), 148.3, 142.6, 139.3, 138.3, 136.5, 134.3, 134.0, 128.0, 128.0, 127.4, 123.5, 122.0, 121.8, 117.9, 116.6, 116.2 (C-F, q, $J_{(C-F)}$ = 287.7 Hz), 54.3, 54.3, 42.9, 37.3, 28.6, 15.7.

^{19}F NMR (377 MHz, CDCl$_3$) δ -72.3.

M.p. 106.2 - 108.1 °C

HRMS (ESI): $[M+H]^+$ calcd. for $C_{23}H_{21}F_3N_3O_2^+$ 428.1580, found 428.1584.

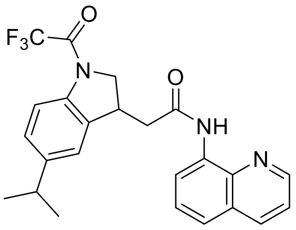

2-(5-isopropyl-1-(2,2,2-trifluoroacetyl)indolin-3-yl)-*N*-(quinolin-8-yl)acetamide (**4d**)

Upon completion the mixture was concentrated and purified via flash column chromatography (petroleum ether / ethyl acetate = 5:1, Rf = 0.3) to give the titled product **4d** as a brown solid (63.8 mg, 72%).

^{1}H NMR (400 MHz, CDCl$_3$) δ 9.85 (s, 1H), 8.79 – 8.75 (m, 2H), 8.18 – 8.12 (m, 2H), 7.54 (q, *J* = 6.5 Hz, 2H), 7.45 (d, *J* = 4.0 Hz, 1H), 7.19 – 7.14 (m, 2H), 4.68 – 4.57 (m, 1H), 4.14 – 4.05 (m, 2H), 3.15 – 3.06 (m, 1H), 2.90 – 2.83 (m, 2H), 1.20 – 1.16 (m, 6H).

^{13}C NMR (101 MHz, CDCl$_3$) δ 168.8, 154.1 (C-F, q, $J_{(C-F)}$ = 37.2 Hz), 148.3, 147.3, 139.4, 138.3, 136.4, 134.3, 128.0, 127.4, 126.7, 122.0, 122.0, 121.7, 121.6, 117.9, 116.7, 116.2 (C-F, q, $J_{(C-F)}$ = 288.3 Hz), 54.4, 43.0, 37.5, 33.9, 24.0.

^{19}F NMR (377 MHz, CDCl$_3$) δ -72.3.

M.p. 120.3 - 121.9 °C

HRMS (ESI): $[M+H]^+$ calcd. for $C_{24}H_{23}F_3N_3O_2^+$ 442.1737, found 442.1739.

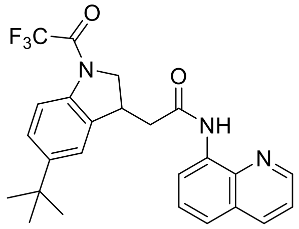

2-(5-(*tert*-butyl)-1-(2,2,2-trifluoroacetyl)indolin-3-yl)-*N*-(quinolin-8-yl)acetamide (**4e**)

Upon completion the mixture was concentrated and purified via flash column chromatography (petroleum ether / ethyl acetate = 5:1, Rf = 0.3) to give the titled product **4e** as a white solid (51.8 mg, 57%).

^{1}H NMR (400 MHz, CDCl$_3$) δ 9.85 (s, 1H), 8.84 – 8.73 (m, 2H), 8.24 – 8.07 (m, 2H), 7.60 – 7.54 (m, 2H), 7.49 – 7.44 (m, 1H), 7.33 (d, *J* = 8.2 Hz, 2H), 4.62 (t, *J* = 9.9 Hz, 1H), 4.16 – 4.08 (m, 2H), 3.10 (dd, *J* = 15.5, 4.9 Hz, 1H), 2.87 (dd, 1H), 1.24 (s, 9H).

^{13}C NMR (101 MHz, CDCl$_3$) δ 168.9, 154.1 (C-F, q, $J_{(C\text{-}F)}$ = 37.2 Hz), 149.6, 148.3, 139.0, 138.3, 136.5, 134.1, 133.9, 128.0, 127.4, 125.6, 122.0, 121.8, 121.0, 117.6, 116.7, 116.1 (C-F, q, $J_{(C\text{-}F)}$ = 287.5 Hz), 54.4, 54.3, 43.1, 37.6, 34.7, 31.4.

^{19}F NMR (377 MHz, CDCl$_3$) δ -72.3.

M.p. 106.4 - 107.8 ^{o}C

HRMS (ESI): $[M+H]^+$ calcd. for $C_{25}H_{25}F_3N_3O_2^+$ 456.1893, found 456.1897.

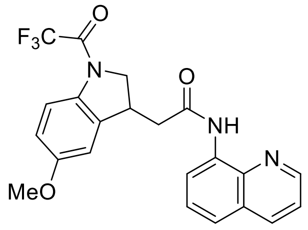

2-(5-methoxy-1-(2,2,2-trifluoroacetyl)indolin-3-yl)-*N*-(quinolin-8-yl)acetamide (**4f**)

Upon completion the mixture was concentrated and purified via flash column chromatography (petroleum ether / ethyl acetate = 5:1, Rf = 0.4) to give the titled product **4f** as a white solid (48.7 mg, 57%).

^{1}H NMR (400 MHz, CDCl$_3$) δ 9.85 (s, 1H), 8.77 (d, *J* = 3.9 Hz, 2H), 8.25 – 8.08 (m, 2H), 7.59 – 7.52 (m, 2H), 7.49 – 7.44 (m, 1H), 6.88 (d, *J* = 2.2 Hz, 1H), 6.82 (d, *J* = 8.9 Hz, 1H), 4.61 (t, *J* = 9.5 Hz, 1H), 4.14 – 4.05 (m, 2H), 3.76 (s, 3H), 3.12 – 3.03 (m, 1H), 2.91 – 2.82 (m, 1H).

^{13}C NMR (101 MHz, CDCl$_3$) δ 168.7, 158.1, 153.7 (C-F, q, $J_{(C\text{-}F)}$ = 37.6 Hz), 148.3, 138.3, 136.5, 135.9, 134.9, 134.0, 128.0, 127.4, 122.0, 121.8, 118.9, 116.7, 116.2 (C-F, q, $J_{(C\text{-}F)}$ = 287.7 Hz), 113.4, 110.1, 55.7, 54.4, 54.3, 42.8, 37.4.

^{19}F NMR (377 MHz, CDCl$_3$) δ -72.1.

M.p. 144.7 - 146.3 °C

HRMS (ESI): $[M+H]^+$ calcd. for $C_{22}H_{19}F_3N_3O_3^+$ 430.1373, found 430.1367.

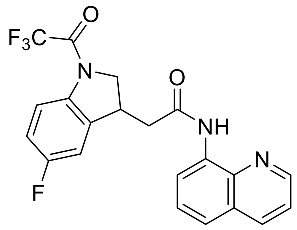

2-(5-fluoro-1-(2,2,2-trifluoroacetyl)indolin-3-yl)-*N*-(quinolin-8-yl)acetamide (**4g**)

Upon completion the mixture was concentrated and purified via flash column chromatography (petroleum ether / ethyl acetate = 5:1, Rf = 0.4) to give the titled product **4g** as a brown solid (47.3 mg, 57%).

^{1}H NMR (400 MHz, DMSO) δ 10.15 (s, 1H), 8.83 (d, *J* = 3.8 Hz, 1H), 8.54 (d, *J* = 7.5 Hz, 1H), 8.31 (d, *J* = 8.2 Hz, 1H), 8.03 – 7.91 (m, 1H), 7.61 – 7.47 (m, 3H), 7.30 (d, *J* = 8.4 Hz, 1H), 7.06 (t, *J* = 8.9 Hz, 1H), 4.51 (t, *J* = 10.0 Hz, 1H), 4.17 – 4.07 (m, 1H), 3.92 (d, *J* = 4.4 Hz, 1H), 3.23 (d, *J* = 4.4 Hz, 1H), 3.01 – 2.87 (m, 1H).

^{13}C NMR (101 MHz, DMSO) δ 170.4, 160.3 (C-F, d, $J_{(C\text{-}F)}$ = 242.4 Hz), 153.5 (C-F, q, $J_{(C\text{-}F)}$ = 36.8 Hz), 149.3, 138.7, 138.6, 138.0, 137.1, 134.8, 128.4, 127.4, 122.6, 118.7 (C-F, d, $J_{(C\text{-}F)}$ = 8.6 Hz), 117.6, 116.3 (C-F, q, $J_{(C\text{-}F)}$ = 288.2 Hz), 114.9, 114.7, 112.6 (C-F, d, $J_{(C\text{-}F)}$ = 24.5 Hz), 54.4, 41.2, 37.5.

^{19}F NMR (377 MHz, CDCl$_3$) δ -116.1, -71.3.

M.p. 158.6 - 160.4 °C

HRMS (ESI): $[M+H]^+$ calcd. for $C_{21}H_{16}F_4N_3O_2^+$ 418.1173, found 418.1169.

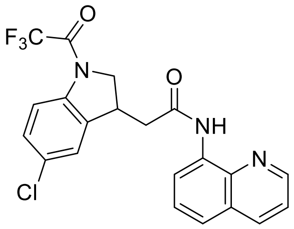

2-(5-chloro-1-(2,2,2-trifluoroacetyl)indolin-3-yl)-*N*-(quinolin-8-yl)acetamide (**4h**)

Upon completion the mixture was concentrated and purified via flash column chromatography (petroleum ether / ethyl acetate = 5:1, Rf = 0.4) to give the titled product **4h** as a brown solid (55.9 mg, 65%).

^{1}H NMR (400 MHz, CDCl$_3$) δ 9.86 (s, 1H), 8.81 – 8.70 (m, 2H), 8.17 (t, *J* = 8.7 Hz, 2H), 7.59 – 7.53 (m, 2H), 7.49 – 7.44 (m, 1H), 7.31 (s, 1H), 7.27 (d, *J* = 6.2 Hz, 1H), 4.65 (t, *J* = 9.6 Hz, 1H), 4.20 – 4.05 (m, 2H), 3.15 – 3.04 (m, 1H), 2.93 – 2.83 (m, 1H).

^{13}C NMR (101 MHz, CDCl$_3$) δ 168.3, 154.4 (C-F, q, $J_{(C-F)}$ = 38.0 Hz), 148.3, 140.1, 138.2, 136.5, 136.1, 133.9, 131.2, 128.6, 128.0, 127.4, 124.5, 122.1, 121.8, 119.0, 116.7,116.0 (C-F, q, $J_{(C-F)}$ = 287.8 Hz), 54.4, 42.5, 37.1.

^{19}F NMR (377 MHz, CDCl$_3$) δ -72.4.

M.p. 139.8 - 141.3 ^{o}C

HRMS (ESI): $[M+H]^+$ calcd. for $C_{21}H_{16}ClF_3N_3O_2^+$ 434.0878, found 434.0878.

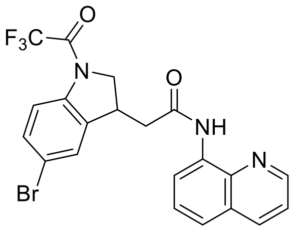

2-(5-bromo-1-(2,2,2-trifluoroacetyl)indolin-3-yl)-*N*-(quinolin-8-yl)acetamide (**4i**)

Upon completion the mixture was concentrated and purified via flash column chromatography (petroleum ether / ethyl acetate = 5:1, Rf = 0.3) to give the titled product **4i** as a brown solid (67.3 mg, 71%).

^{1}H NMR (400 MHz, CDCl$_3$) δ 9.86 (s, 1H), 8.78 (d, *J* = 5.5 Hz, 1H), 8.74 (d, *J* = 8.8 Hz, 1H), 8.17 (d, *J* = 9.6 Hz, 1H), 8.10 (d, *J* = 8.6 Hz, 1H), 7.59 – 7.52 (m, 2H), 7.49 – 7.44 (m, 2H), 7.41 (d, *J* = 8.6 Hz, 1H), 4.64 (t, *J* = 9.6 Hz, 1H), 4.20 – 4.03 (m, 2H), 3.16 – 3.03 (m, 1H), 2.96 – 2.81 (m, 1H).

^{13}C NMR (101 MHz, CDCl$_3$) δ 168.3, 154.4 (C-F, q, $J_{(C-F)}$ = 37.9 Hz), 148.3, 140.6, 138.2, 136.5, 133.9, 131.6, 128.0, 127.4, 127.3, 122.1, 121.8, 119.4, 118.8, 116.7,116.0 (C-F, q, $J_{(C-F)}$ = 287.8 Hz), 54.3, 54.3, 42.5, 37.1.

^{19}F NMR (377 MHz, CDCl$_3$) δ -72.4.

M.p. 142.9 - 144.8 ^{o}C

HRMS (ESI): [M+H]$^+$ calcd. for $C_{21}H_{16}BrF_3N_3O_2^+$ 478.0373, found 478.0373.

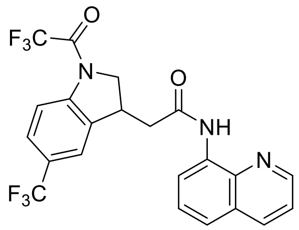

N-(quinolin-8-yl)-2-(1-(2,2,2-trifluoroacetyl)-5-(trifluoromethyl)indolin-3-yl)acetamide (**4j**)

Upon completion the mixture was concentrated and purified via flash column chromatography (petroleum ether / ethyl acetate = 5:1, Rf = 0.3) to give the titled product **4j** as a white solid (59.5 mg, 64%).

^{1}H NMR (400 MHz, CDCl$_3$) δ 9.88 (s, 1H), 8.77 (d, *J* = 2.6 Hz, 1H), 8.75 – 8.72 (m, 1H), 8.32 (d, *J* = 8.5 Hz, 1H), 8.18 (d, *J* = 6.8 Hz, 1H), 7.62 – 7.53 (m, 4H), 7.49 – 7.44 (m, 1H), 4.71 (t, *J* = 9.7 Hz, 1H), 4.25 – 4.12 (m, 2H), 3.20 – 3.13 (m, 1H), 2.96 – 2.87 (m, 1H).

^{13}C NMR (101 MHz, CDCl$_3$) δ 168.1, 154.8 (C-F, q, $J_{(C-F)}$ = 38.3 Hz), 148.3, 144.3, 138.3, 136.4, 135.0, 133.9, 128.2 (C-F, q, $J_{(C-F)}$ = 32.2 Hz), 128.0, 127.3, 126.2 (C-F, q, $J_{(C-F)}$ = 3.7 Hz), 123.9 (C-F, q, $J_{(C-F)}$ = 272.2 Hz), 122.1, 121.8, 121.3 (C-F, q, $J_{(C-F)}$ = 3.4 Hz), 118.0, 116.7, 115.9 (C-F, q, $J_{(C-F)}$ = 287.8 Hz), 54.5, 42.4, 37.1.

^{19}F NMR (377 MHz, CDCl$_3$) δ -72.6, -62.0.

M.p. 138.3 - 139.9 ^{o}C

HRMS (ESI): [M+H]$^+$ calcd. for $C_{22}H_{16}F_6N_3O_2^+$ 468.1141, found 468.1145.

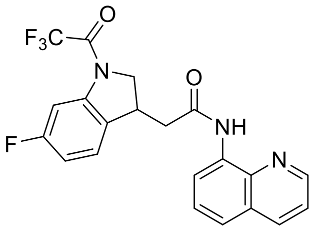

2-(6-fluoro-1-(2,2,2-trifluoroacetyl)indolin-3-yl)-*N*-(quinolin-8-yl)acetamide (**4k**)

Upon completion the mixture was concentrated and purified via flash column chromatography (petroleum ether / ethyl acetate = 5:1, Rf = 0.3) to give the titled product **4k** as a brown solid (49.7 mg, 60%).

^{1}H NMR (400 MHz, CDCl$_3$) δ 9.86 (s, 1H), 8.82 – 8.68 (m, 2H), 8.18 (d, *J* = 6.7 Hz, 1H), 7.98 (d, *J* = 12.4 Hz, 1H), 7.57 – 7.53 (m, 2H), 7.50 – 7.44 (m, 1H), 7.27 (s, 1H), 6.86 (t, *J* = 9.7 Hz, 1H), 4.71 – 4.61 (m, 1H), 4.20 – 4.13 (m, 1H), 4.12 – 4.05 (m, 1H), 3.10 – 3.03 (m, 1H), 2.92 – 2.83 (m, 1H).

^{13}C NMR (101 MHz, CDCl$_3$) δ 168.5, 162.6 (C-F, d, $J_{(C-F)}$ = 244.8 Hz), 154.5 (C-F, q, $J_{(C-F)}$ = 37.8 Hz), 148.3, 142.6 (C-F, d, $J_{(C-F)}$ = 12.5 Hz), 138.2, 136.5, 134.0, 129.6, 129.6, 128.0, 127.3, 124.9 (C-F, d, $J_{(C-F)}$ = 9.8 Hz), 122.1, 121.8, 116.7, 115.9 (C-F, q, $J_{(C-F)}$ = 287.8 Hz), 112.8 (C-F, d, $J_{(C-F)}$ = 23.0 Hz), 106.3 (C-F, d, $J_{(C-F)}$ = 29.1 Hz), 54.9, 42.9, 36.6.

^{19}F NMR (377 MHz, CDCl$_3$) δ -112.0, -72.5.

M.p. 112.3 - 113.9 ^{o}C

HRMS (ESI): $[M+H]^+$ calcd. for $C_{21}H_{16}F_4N_3O_2^+$ 418.1173, found 418.1176.

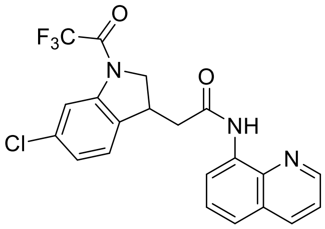

2-(6-chloro-1-(2,2,2-trifluoroacetyl)indolin-3-yl)-*N*-(quinolin-8-yl)acetamide (**4l**)

Upon completion the mixture was concentrated and purified via flash column chromatography (petroleum ether / ethyl acetate = 5:1, Rf = 0.4) to give the titled product **4l** as a white solid (53.4 mg, 62%).

^{1}H NMR (400 MHz, CDCl$_3$) δ 9.86 (s, 1H), 8.81 – 8.72 (m, 2H), 8.26 (d, *J* = 1.8 Hz, 1H), 8.18 (d, *J* = 6.7 Hz, 1H), 7.59 – 7.53 (m, 2H), 7.50 – 7.45 (m, 1H), 7.25 (d, *J* = 7.8 Hz, 1H), 7.14 (d, *J* = 10.0 Hz, 1H), 4.64 (t, *J* = 9.7 Hz, 1H), 4.18 – 4.05 (m, 2H), 3.11 – 3.02 (m, 1H), 2.93 – 2.84 (m, 1H).

^{13}C NMR (101 MHz, CDCl$_3$) δ 168.3, 154.5 (C-F, q, $J_{(C\text{-}F)}$ = 37.8 Hz), 148.3, 142.5, 138.3, 136.5, 134.3, 134.0, 132.7, 128.0, 127.3, 126.1, 124.9, 122.1, 121.8, 118.5, 116.7, 115.9 (C-F, q, $J_{(C\text{-}F)}$ = 287.9 Hz), 54.6, 42.7, 36.9.

^{19}F NMR (377 MHz, CDCl$_3$) δ -72.5.

M.p. 158.6 - 159.9 °C

HRMS (ESI): $[M+H]^+$ calcd. for $C_{21}H_{16}ClF_3N_3O_2^+$ 434.0878, found 434.0871.

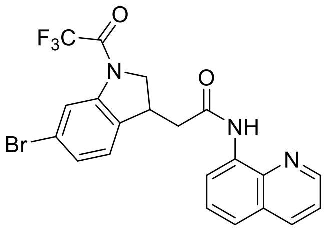

2-(6-bromo-1-(2,2,2-trifluoroacetyl)indolin-3-yl)-*N*-(quinolin-8-yl)acetamide (**4m**)

Upon completion the mixture was concentrated and purified via flash column chromatography (petroleum ether / ethyl acetate = 5:1, Rf = 0.3) to give the titled product **4m** as a white solid (58.1 mg, 61%).

^{1}H NMR (400 MHz, CDCl$_3$) δ 9.86 (s, 1H), 8.77 (d, *J* = 2.6 Hz, 1H), 8.76 – 8.73 (m, 1H), 8.18 (d, *J* = 9.9 Hz, 1H), 7.59 – 7.52 (m, 2H), 7.49 – 7.44 (m, 1H), 7.29 (d, *J* = 6.3 Hz, 1H), 7.19 (d, *J* = 8.1 Hz, 1H), 4.68 – 4.58 (m, 1H), 4.18 – 4.02 (m, 2H), 3.12 – 3.01 (m, 1H), 2.93 – 2.83 (m, 1H).

^{13}C NMR (101 MHz, CDCl$_3$) δ 168.4, 154.5 (C-F, q, $J_{(C\text{-}F)}$ = 37.7 Hz), 148.3, 142.6, 138.2, 136.5, 133.9, 133.3, 129.1, 128.0, 127.3, 125.4, 122.1, 122.0, 121.8, 121.2, 116.7, 115.9 (C-F, q, $J_{(C\text{-}F)}$ = 287.8 Hz), 54.5, 42.6, 36.9.

^{19}F NMR (377 MHz, CDCl$_3$) δ -72.5.

M.p. 181.9 - 183.6 °C

HRMS (ESI): $[M+H]^+$ calcd. for $C_{21}H_{16}BrF_3N_3O_2^+$ 478.0373, found 478.0374.

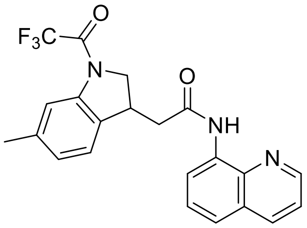

2-(6-methyl-1-(2,2,2-trifluoroacetyl)indolin-3-yl)-*N*-(quinolin-8-yl)acetamide (**4n**)

Upon completion the mixture was concentrated and purified via flash column chromatography (petroleum ether / ethyl acetate = 5:1, Rf = 0.4) to give the titled product **4n** as a white solid (59.6 mg, 72%).

^{1}H NMR (400 MHz, DMSO) δ 10.24 (s, 1H), 8.93 (d, *J* = 3.3 Hz, 1H), 8.66 (d, *J* = 7.6 Hz, 1H), 8.41 (d, *J* = 7.8 Hz, 1H), 7.93 (s, 1H), 7.69 (d, *J* = 8.2 Hz, 1H), 7.66 – 7.57 (m, 2H), 7.37 (d, *J* = 7.6 Hz, 1H), 7.04 (d, *J* = 7.7 Hz, 1H), 4.57 (t, *J* = 10.0 Hz, 1H), 4.21 – 4.13 (m, 1H), 4.01 – 3.89 (m, 1H), 3.32 – 3.24 (m, 1H), 3.02 – 2.91 (m, 1H), 2.33 (s, 3H).

^{13}C NMR (101 MHz, DMSO) δ 170.5, 153.6 (C-F, q, $J_{(C\text{-}F)}$ = 36.7 Hz), 149.3, 141.7, 138.7, 137.9, 137.0, 134.9, 133.0, 128.3, 127.4, 127.0, 124.8, 122.6, 118.1, 117.5, 116.4 (C-F, q, $J_{(C\text{-}F)}$ = 288.3 Hz), 54.4, 41.7, 37.1, 21.7.

^{19}F NMR (377 MHz, $CDCl_3$) δ -71.3.

M.p. 102.1 - 103.9 ^{o}C

HRMS (ESI): $[M+H]^+$ calcd. for $C_{22}H_{19}F_3N_3O_2^+$ 414.1424, found 414.1418.

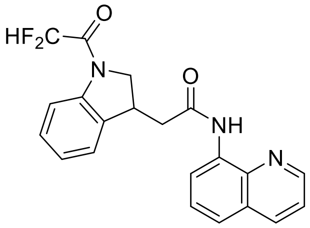

2-(1-(2,2-difluoroacetyl)indolin-3-yl)-*N*-(quinolin-8-yl)acetamide (**4o**)

Upon completion the mixture was concentrated and purified via flash column chromatography (petroleum ether / ethyl acetate = 5:1, Rf = 0.2) to give the titled product **4o** as a white solid (64.3 mg, 84%).

^{1}H NMR (400 MHz, DMSO) δ 10.28 (s, 1H), 8.94 (d, *J* = 2.8 Hz, 1H), 8.69 (d, *J* = 7.6 Hz, 1H), 8.43 (d, *J* = 8.3 Hz, 1H), 8.11 (d, *J* = 8.0 Hz, 1H), 7.70 (d, *J* = 8.1 Hz, 1H), 7.67 – 7.59 (m, 2H), 7.47 (d, *J* = 7.4 Hz, 1H), 7.30 (t, *J* = 7.7 Hz, 1H), 7.17 (t, *J* = 7.4 Hz, 1H), 6.77 (t, *J* = 52.8 Hz, 1H), 4.53 (t, *J* = 9.9 Hz, 1H), 4.12 – 4.05 (m, 1H), 4.04 – 3.95 (m, 1H), 3.34 – 3.25 (m, 1H), 3.05 – 2.94 (m, 1H).

^{13}C NMR (101 MHz, DMSO) δ 170.5, 160.0 (C-F, t, $J_{(C-F)}$ = 26.2 Hz), 149.3, 142.1, 138.7, 137.1, 135.4, 134.9, 128.4, 128.3, 127.4, 125.5, 125.0, 122.6, 122.6, 117.6, 117.0, 108.1 (C-F, t, $J_{(C-F)}$ = 243.2 Hz), 53.1, 42.0, 37.4.

^{19}F NMR (377 MHz, CDCl$_3$) δ -126.9.

M.p. 122.8 - 124.6 ^{o}C

HRMS (ESI): $[M+H]^+$ calcd. for $C_{21}H_{18}F_2N_3O_2^+$ 382.1362, found 382.1360.

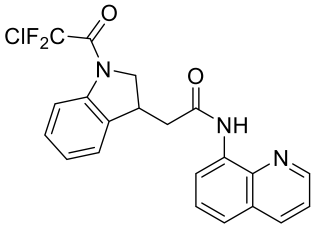

2-(1-(2-chloro-2,2-difluoroacetyl)indolin-3-yl)-*N*-(quinolin-8-yl)acetamide (**4p**)

Upon completion the mixture was concentrated and purified via flash column chromatography (petroleum ether / ethyl acetate = 5:1, Rf = 0.3) to give the titled product **4p** as a brown solid (53.3 mg, 64%).

^{1}H NMR (400 MHz, CDCl$_3$) δ 9.87 (s, 1H), 8.80 – 8.75 (m, 2H), 8.23 (d, *J* = 8.1 Hz, 1H), 8.18 (d, *J* = 9.9 Hz, 1H), 7.59 – 7.53 (m, 2H), 7.48 – 7.45 (m, 1H), 7.36 – 7.29 (m, 2H), 7.18 (t, *J* = 7.5 Hz, 1H), 4.67 – 4.59 (m, 1H), 4.25 – 4.18 (m, 1H), 4.17 – 4.07 (m, 1H), 3.12 – 3.04 (m, 1H), 2.91 – 2.82 (m, 1H).

^{13}C NMR (101 MHz, CDCl$_3$) δ 168.7, 156.3 (C-F, t, $J_{(C-F)}$ = 30.8 Hz), 148.3, 141.8, 138.3, 136.4, 134.2, 134.1, 128.6, 128.0, 127.4, 126.0, 124.2, 122.0, 121.8, 118.6 (C-F, t, $J_{(C-F)}$ = 300.4 Hz), 118.3, 116.7, 54.9, 42.9, 37.5.

^{19}F NMR (377 MHz, CDCl$_3$) δ -60.6.

M.p. 102.4 - 104.2 °C

HRMS (ESI): $[M+H]^+$ calcd. for $C_{21}H_{17}ClF_2N_3O_2^+$ 416.0972, found 416.0968.

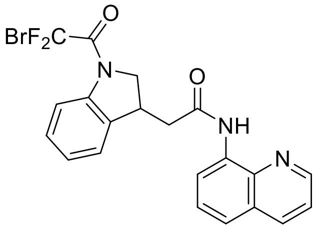

2-(1-(2-bromo-2,2-difluoroacetyl)indolin-3-yl)-*N*-(quinolin-8-yl)acetamide (**4q**)

Upon completion the mixture was concentrated and purified via flash column chromatography (petroleum ether / ethyl acetate = 5:1, Rf = 0.3) to give the titled product **4q** as a brown oily liquid (70.8 mg, 77%).

^{1}H NMR (400 MHz, $CDCl_3$) δ 9.87 (s, 1H), 8.83 – 8.72 (m, 2H), 8.23 (d, *J* = 8.1 Hz, 1H), 8.17 (d, *J* = 9.8 Hz, 1H), 7.60 – 7.52 (m, 2H), 7.45 (dd, *J* = 8.3, 4.2 Hz, 1H), 7.32 (dd, *J* = 16.1, 7.8 Hz, 2H), 7.17 (t, *J* = 7.2 Hz, 1H), 4.63 – 4.54 (m, 1H), 4.26 – 4.19 (m, 1H), 4.14 – 4.08 (m, 1H), 3.06 (dd, *J* = 15.6, 5.1 Hz, 1H), 2.86 (dd, *J* = 15.6, 9.5 Hz, 1H).

^{13}C NMR (101 MHz, $CDCl_3$) δ 168.8, 156.8 (C-F, t, $J_{(C-F)}$ = 27.8 Hz), 148.3, 141.8, 138.2, 136.5, 134.2, 134.0, 128.6, 128.0, 127.4, 126.1, 124.2, 122.0, 121.8, 118.4, 116.7, 110.8 (C-F, t, $J_{(C-F)}$ = 315.4 Hz), 55.2, 43.0, 37.6.

^{19}F NMR (377 MHz, $CDCl_3$) δ -57.1.

HRMS (ESI): $[M+H]^+$ calcd. for $C_{21}H_{17}BrF_2N_3O_2^+$ 460.0467, found 460.0475.

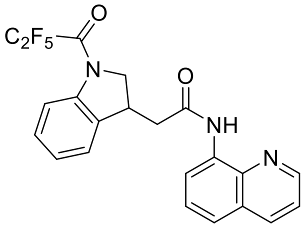

2-(1-(2,2,3,3,3-pentafluoropropanoyl)indolin-3-yl)-*N*-(quinolin-8-yl)acetamide (**4r**)

Upon completion the mixture was concentrated and purified via flash column chromatography (petroleum ether / ethyl acetate = 5:1, Rf = 0.3) to give the titled product **4q** as a brown solid (23.4 mg, 26%).

^{1}H NMR (400 MHz, CDCl$_3$) δ 9.86 (s, 1H), 8.80 – 8.75 (m, 2H), 8.24 (d, *J* = 8.1 Hz, 1H), 8.18 (d, *J* = 9.8 Hz, 1H), 7.56 (d, *J* = 6.8 Hz, 2H), 7.48 – 7.45 (m, 1H), 7.35 (d, *J* = 7.4 Hz, 1H), 7.31 (t, *J* = 7.8 Hz, 1H), 7.18 (t, *J* = 7.3 Hz, 1H), 4.72 – 4.62 (m, 1H), 4.24 – 4.10 (m, 2H), 3.13 – 3.06 (m, 1H), 2.92 – 2.83 (m, 1H).

^{13}C NMR (101 MHz, CDCl$_3$) δ 168.7, 155.5 (C-F, t, $J_{(C\text{-}F)}$ = 26.2 Hz), 148.3, 141.6, 138.3, 136.5, 134.2, 134.0, 128.6, 128.0, 127.4, 126.3, 124.2, 122.0, 121.8, 118.4, 116.7, 54.0, 42.9, 37.4.

^{19}F NMR (377 MHz, CDCl$_3$) δ -117.8, -81.8.

M.p. 136.4 - 137.8 °C

HRMS (ESI): $[M+H]^+$ calcd. for $C_{22}H_{17}F_5N_3O_2^+$ 450.1235, found 450.1299.

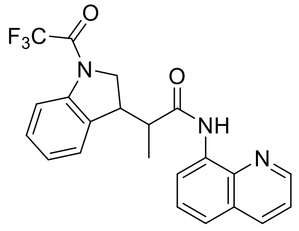

N-(quinolin-8-yl)-2-(1-(2,2,2-trifluoroacetyl)indolin-3-yl)propanamide (**4s**)

Upon completion the mixture was concentrated and purified via flash column chromatography (petroleum ether / ethyl acetate = 5:1, Rf = 0.4) to give the titled product **4s** as a white solid (32.4 mg, 39%).

^{1}H NMR (400 MHz, CDCl$_3$) δ 9.92 (s, 1H), 8.83 – 8.79 (m, 1H), 8.79 – 8.76 (m, 1H), 8.23 (d, *J* = 8.1 Hz, 1H), 8.20 – 8.16 (m, 1H), 7.59 – 7.54 (m, 2H), 7.49 – 7.45 (m, 1H), 7.33 – 7.27 (m, 2H), 7.15 – 7.10 (m, 1H), 4.48 – 4.35 (m, 2H), 4.09 – 4.00 (m, 1H), 3.05 – 2.95 (m, 1H), 1.29 (d, *J* = 7.0 Hz, 3H).

^{13}C NMR (101 MHz, CDCl$_3$) δ 172.8, 154.3 (C-F, q, $J_{(C\text{-}F)}$ = 38.5 Hz), 148.3, 141.9, 138.5, 136.4, 134.1, 133.3, 128.7, 128.0, 127.4, 126.1, 124.5, 122.0, 121.7, 118.0, 116.7, 116.1 (C-F, q, $J_{(C\text{-}F)}$ = 287.7 Hz), 50.3, 46.0, 42.9, 13.4.

^{19}F NMR (377 MHz, CDCl$_3$) δ -72.5.

M.p. 152.2 - 154.1 °C

HRMS (ESI): $[M+H]^+$ calcd. for $C_{22}H_{19}F_3N_3O_2^+$ 414.1424, found 414.1423.

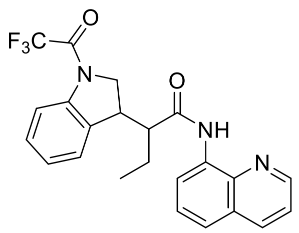

N-(quinolin-8-yl)-2-(1-(2,2,2-trifluoroacetyl)indolin-3-yl)butanamide (**4t**)

Upon completion the mixture was concentrated and purified via flash column chromatography (petroleum ether / ethyl acetate = 5:1, Rf = 0.4) to give the titled product **4t** as a brown solid (65.4 mg, 77%).

^{1}H NMR (400 MHz, CDCl$_3$) δ 9.90 (s, 1H), 8.89 (d, *J* = 8.9 Hz, 1H), 8.81 (d, *J* = 5.8 Hz, 1H), 8.25 (d, *J* = 8.1 Hz, 1H), 8.21 (d, *J* = 9.9 Hz, 1H), 7.63 – 7.57 (m, 2H), 7.51 – 7.47 (m, 1H), 7.36 (d, *J* = 7.6 Hz, 1H), 7.29 (s, 1H), 7.10 (t, *J* = 7.5 Hz, 1H), 4.55 (dd, *J* = 11.6, 4.1 Hz, 1H), 4.43 – 4.34 (m, 1H), 4.02 – 3.93 (m, 1H), 2.76 – 2.66 (m, 1H), 2.00 – 1.88 (m, 1H), 1.61 – 1.51 (m, 1H), 1.06 (t, *J* = 7.4 Hz, 3H).

^{13}C NMR (101 MHz, CDCl$_3$) δ 172.3, 154.2 (C-F, q, $J_{(C-F)}$ = 37.5 Hz), 148.3, 141.7, 138.4, 136.4, 133.9, 133.3, 128.7, 128.0, 127.4, 126.1, 124.7, 122.1, 121.8, 118.0, 116.8, 116.2 (C-F, q, $J_{(C-F)}$ = 288.0 Hz), 54.2, 50.7, 43.0, 22.2, 12.3.

^{19}F NMR (377 MHz, CDCl$_3$) δ -72.3.

M.p. 65.7 - 67.2 °C

HRMS (ESI): $[M+H]^+$ calcd. for $C_{23}H_{21}F_3N_3O_2^+$ 428.1580, found 428.1577.

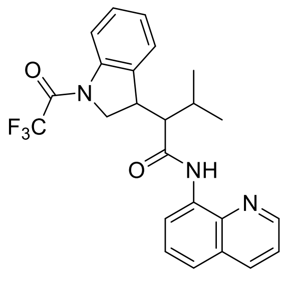

3-methyl-*N*-(quinolin-8-yl)-2-(1-(2,2,2-trifluoroacetyl)indolin-3-yl)butanamide (**4u**)

Upon completion the mixture was concentrated and purified via flash column chromatography (petroleum ether / ethyl acetate = 5:1, Rf = 0.3) to give the titled product **4u** as a yellow solid (31.2 mg, 35%).

^{1}H NMR (400 MHz, CDCl$_3$) δ 9.70 (s, 1H), 8.83 (d, *J* = 8.8 Hz, 1H), 8.71 (d, *J* = 5.6 Hz, 1H), 8.19 – 8.12 (m, 2H), 7.59 – 7.51 (m, 2H), 7.43 (dd, *J* = 8.3, 4.2 Hz, 1H), 7.33 (d, *J* = 7.6 Hz, 1H), 7.20 (t, *J* = 7.8 Hz, 1H), 7.01 (t, *J* = 7.5 Hz, 1H), 4.44 – 4.29 (m, 2H), 4.13 – 4.04 (m, 1H), 2.62 (dd, *J* = 9.1, 5.6 Hz, 1H), 2.26 – 2.15 (m, 1H), 1.19 – 1.11 (m, 6H).

^{13}C NMR (101 MHz, CDCl$_3$) δ 170.8, 154.1 (C-F, q, $J_{(C\text{-}F)}$ = 37.4 Hz), 148.3, 141.4, 138.4, 136.3, 133.9, 133.8, 128.5, 127.9, 127.4, 126.1, 125.2, 121.9, 121.7, 117.9, 116.5, 116.2 (C-F, q, $J_{(C\text{-}F)}$ = 288.0 Hz), 58.2, 51.1, 40.9, 28.9, 22.1, 18.3.

^{19}F NMR (377 MHz, CDCl$_3$) δ -72.2.

M.p. 110.6 - 111.8 ^{o}C

HRMS (ESI): $[M+H]^+$ calcd. for $C_{24}H_{23}F_3N_3O_2^+$ 442.1737, found 442.1737.

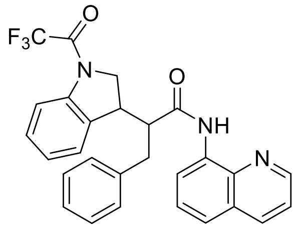

3-phenyl-*N*-(quinolin-8-yl)-2-(1-(2,2,2-trifluoroacetyl)indolin-3-yl)propanamide (**4v**)

Upon completion the mixture was concentrated and purified via flash column chromatography (petroleum ether / ethyl acetate = 5:1, Rf = 0.3) to give the titled product **4v** as a yellow solid (50.6 mg, 52%).

^{1}H NMR (400 MHz, CDCl$_3$) δ 9.44 (s, 1H), 8.74 (d, *J* = 7.2 Hz, 1H), 8.59 (d, *J* = 2.6 Hz, 1H), 8.24 (d, *J* = 8.1 Hz, 1H), 8.09 (d, *J* = 8.3 Hz, 1H), 7.54 – 7.46 (m, 2H), 7.40 – 7.35 (m, 2H), 7.29 (d, *J* = 7.7 Hz, 1H), 7.16 – 7.05 (m, 6H), 4.62 (dd, *J* = 11.7, 4.2 Hz, 1H), 4.46 – 4.37 (m, 1H), 4.08 – 4.00 (m, 1H), 3.17 – 3.03 (m, 2H), 2.74 (d, *J* = 12.8 Hz, 1H).

^{13}C NMR (101 MHz, CDCl$_3$) δ 171.6, 154.4 (C-F, q, $J_{(C-F)}$ = 37.4 Hz), 148.1, 142.0, 138.5, 138.3, 136.3, 133.8, 133.0, 128.9, 128.8, 128.8, 127.9, 127.3, 126.7, 126.2, 124.7, 122.0, 121.7, 118.2, 116.7, 116.2 (C-F, q, $J_{(C-F)}$ = 287.7 Hz), 54.7, 50.8, 43.2, 34.9.

^{19}F NMR (377 MHz, CDCl$_3$) δ -72.3.

M.p. 155.6 - 156.8 °C

HRMS (ESI): $[M+H]^+$ calcd. for $C_{28}H_{23}F_3N_3O_2^+$ 490.1737, found 490.1744.

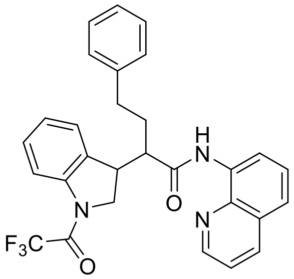

4-phenyl-*N*-(quinolin-8-yl)-2-(1-(2,2,2-trifluoroacetyl)indolin-3-yl)butanamide (**4w**)

Upon completion the mixture was concentrated and purified via flash column chromatography (petroleum ether / ethyl acetate = 5:1, Rf = 0.3) to give the titled product **4w** as a yellow solid (62.6 mg, 62%).

^{1}H NMR (400 MHz, CDCl$_3$) δ 9.83 (d, J = 11.4 Hz, 1H), 8.86 (d, J = 8.9 Hz, 1H), 8.79 (d, J = 2.6 Hz, 1H), 8.22 – 8.16 (m, 2H), 7.62 – 7.55 (m, 2H), 7.47 (dd, J = 8.3, 4.2 Hz, 1H), 7.27 – 7.21 (m, 4H), 7.19 – 7.10 (m, 4H), 7.02 (t, J = 7.5 Hz, 1H), 4.46 (dd, J = 11.5, 4.8 Hz, 1H), 4.38 – 4.29 (m, 1H), 3.96 – 3.87 (m, 1H), 2.88 – 2.80 (m, 1H), 2.75 – 2.67 (m, 1H), 2.58 (dt, J = 14.1, 8.3 Hz, 1H), 2.34 – 2.23 (m, 1H), 1.76 – 1.70 (m, 1H).

^{13}C NMR (101 MHz, CDCl$_3$) δ 172.1, 154.2 (C-F, q, $J_{(C-F)}$ = 37.4 Hz), 148.3, 141.7, 140.5, 138.4, 136.5, 133.9, 132.9, 128.7, 128.6, 128.6, 128.0, 127.4, 126.3, 126.0, 124.7, 122.2, 121.9, 118.0, 116.9, 116.1 (C-F, q, $J_{(C-F)}$ = 287.8 Hz), 50.9, 50.7, 43.1, 33.3, 30.3.

^{19}F NMR (377 MHz, CDCl$_3$) δ -72.4.

M.p. 65.4 - 66.7 °C

HRMS (ESI): $[M+H]^+$ calcd. for $C_{29}H_{25}F_3N_3O_2^+$ 504.1893, found 504.1898.

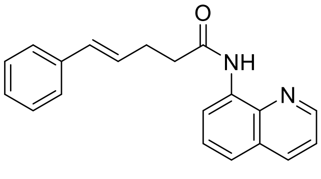

(E)-5-phenyl-N-(quinolin-8-yl)pent-4-enamide (**6**)

Upon completion the mixture was concentrated and purified via flash column chromatography (petroleum ether / ethyl acetate = 5:1, Rf = 0.5) to give the titled product **6** as a yellow oily liquid .

^{1}H NMR (400 MHz, CDCl$_3$) δ 9.86 (s, 1H), 8.84 – 8.74 (m, 2H), 8.15 (dd, J = 8.3, 1.6 Hz, 1H), 7.52 (dd, J = 9.1, 4.5 Hz, 2H), 7.45 – 7.42 (m, 1H), 7.35 (d, J = 7.2 Hz, 2H), 7.29 (d, J = 7.3 Hz, 2H), 7.19 (t, J = 7.2 Hz, 1H), 6.53 (d, J = 15.7 Hz, 1H), 6.38 – 6.28 (m, 1H), 2.75 (d, J = 2.8 Hz, 4H).

^{13}C NMR (101 MHz, CDCl$_3$) δ 170.9, 148.2, 138.4, 137.5, 136.4, 134.5, 132.2, 132.1, 131.3, 128.7, 128.5, 128.0, 127.5, 127.1, 126.1, 121.6, 121.5, 116.5, 37.8, 28.9.

HRMS (ESI): $[M+H]^+$ calcd. for $C_{20}H_{19}N_2O^+$ 303.1492, found 303.1490.

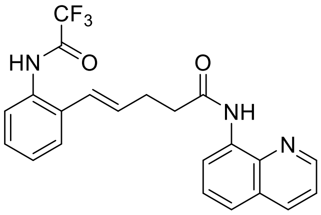

(E)-N-(quinolin-8-yl)-5-(2-(2,2,2-trifluoroacetamido)phenyl)pent-4-enamide (**7**)

Upon completion the mixture was concentrated and purified via flash column chromatography (petroleum ether / ethyl acetate = 5:1, Rf = 0.2) to give the titled product **7** as a yellow oily liquid (25.6 mg, 31%).

^{1}H NMR (400 MHz, CDCl$_3$) δ 9.84 (s, 1H), 8.78 – 8.72 (m, 2H), 8.22 (s, 1H), 8.15 (dd, J = 8.3, 1.6 Hz, 1H), 7.83 (d, J = 8.0 Hz, 1H), 7.53 (dd, J = 11.9, 5.1 Hz, 2H), 7.44 (dd, J = 8.3, 4.2 Hz, 1H), 7.37 (d, J = 7.7 Hz, 1H), 7.30 – 7.26 (m, 1H), 7.19 (t, J = 7.1 Hz, 1H), 6.55 (d, J = 15.7 Hz, 1H), 6.28 – 6.18 (m, 1H), 2.76 (d, J = 3.1 Hz, 4H).

^{13}C NMR (101 MHz, CDCl$_3$) δ 170.4, 155.1 (C-F, q, $J_{(C\text{-}F)}$ = 36.7 Hz), 148.2, 138.4, 136.4, 134.9, 134.3, 131.6, 130.7, 128.1, 128.0, 127.8, 127.4, 126.8, 125.6, 123.2, 121.6, 116.6, 115.9 (C-F, q, $J_{(C\text{-}F)}$ = 288.8 Hz), 37.1, 29.1.

^{19}F NMR (377 MHz, CDCl$_3$) δ -75.4.

HRMS (ESI): $[M+H]^+$ calcd. for $C_{22}H_{19}F_3N_3O_2^+$ 414.1424, found 414.1427.

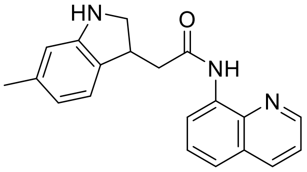

2-(6-methylindolin-3-yl)-*N*-(quinolin-8-yl)acetamide (**9**)

Upon completion the mixture was concentrated and purified via flash column chromatography (petroleum ether / ethyl acetate = 5:1, Rf = 0.5) to give the titled product **9** as a yellow oily liquid (61.5 mg, 97%).

^{1}H NMR (400 MHz, DMSO) δ 10.17 (s, 1H), 8.92 (dd, *J* = 4.1, 1.3 Hz, 1H), 8.69 (d, *J* = 7.5 Hz, 1H), 8.40 (dd, *J* = 8.2, 1.1 Hz, 1H), 7.68 (d, *J* = 8.1 Hz, 1H), 7.66 – 7.58 (m, 2H), 6.98 (d, *J* = 7.8 Hz, 1H), 6.35 (d, *J* = 6.5 Hz, 2H), 5.45 (s, 1H), 3.70 – 3.62 (m, 2H), 3.28 – 3.18 (m, 1H), 3.10 – 3.01 (m, 1H), 2.79 (dd, *J* = 15.2, 7.7 Hz, 1H), 2.17 (s, 3H).

^{13}C NMR (101 MHz, $CDCl_3$) δ 170.3, 151.5, 148.2, 138.3, 137.9, 136.4, 134.4, 128.7, 128.0, 127.4, 123.8, 121.7, 121.6, 119.6, 116.5, 110.6, 53.6, 42.9, 38.6, 21.6.

HRMS (ESI): $[M+H]^+$ calcd. for $C_{20}H_{20}N_3O^+$ 318.1601, found 318.1607.

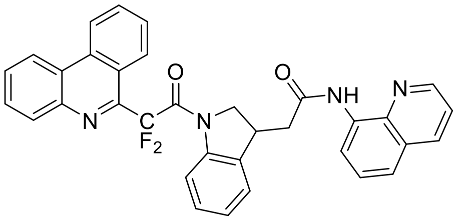

2-(1-(2,2-difluoro-2-(phenanthridin-6-yl)acetyl)indolin-3-yl)-*N*-(quinolin-8-yl)acetamide (**10**)

Upon completion the mixture was concentrated and purified via flash column chromatography (petroleum ether / ethyl acetate = 3:1, Rf = 0.3) to give the titled product **10** as a yellow oily liquid (11.2 mg, 20%).

^{1}H NMR (400 MHz, $CDCl_3$) δ 9.50 (s, 1H), 8.74 (dd, *J* = 4.2, 1.5 Hz, 1H), 8.69 (d, *J* = 8.2 Hz, 1H), 8.63 (d, *J* = 8.3 Hz, 1H), 8.48 – 8.36 (m, 3H), 8.15 (dd, *J* = 8.3, 1.5 Hz, 1H), 8.10 (d, *J* = 8.1 Hz, 1H), 7.92 (t, *J* = 7.6 Hz, 1H), 7.78 (t, *J* = 7.7 Hz, 1H), 7.52 – 7.45 (m, 3H), 7.45 – 7.39 (m, 2H), 7.37 (d, *J*

= 9.4 Hz, 1H), 7.31 (d, *J* = 7.2 Hz, 1H), 7.14 (t, *J* = 7.5 Hz, 1H), 4.42 – 4.34 (m, 1H), 4.01 (d, *J* = 11.6 Hz, 1H), 3.92 – 3.83 (m, 1H), 2.88 (dd, *J* = 15.8, 5.8 Hz, 1H), 2.68 (dd, *J* = 15.8, 8.9 Hz, 1H).

^{13}C NMR (101 MHz, $CDCl_3$) δ 168.8, 156.3 (C-F, t, $J_{(C-F)}$ = 36.6 Hz), 148.1, 142.6, 141.8, 138.2, 136.3, 134.5, 134.1, 134.0, 131.4, 130.7, 129.1, 128.7, 128.5, 128.0, 127.9, 127.3, 126.8, 125.2, 124.8, 124.2, 122.6, 122.5, 121.9, 121.7, 121.6, 118.5, 116.3, 115.2 (C-F, t, $J_{(C-F)}$ = 282.5 Hz), 55.1, 43.0, 37.2.

HRMS (ESI): $[M+H]^+$ calcd. for $C_{34}H_{25}F_3N_4O_2^+$ 559.1940, found 559.1948.

7 References

(1) K. Tamura, H. Mizukami, K. Maeda, H. Watanabe and K. Uneyama, *J. Org. Chem.* 1993, **58**, 32.

(2) S. Yang, L. Liu, Z. Zhou, Z. Huang and Y. Zhao, *Org. Lett.* 2021, **23**, 296.

(3) J.-B. Peng, F.-P. Wu, D. Li, H.-Q. Geng, X. Qi, J. Ying and X.-F. Wu, *ACS Catal.* 2019, **9**, 2977.

8 Copy of ^{1}H, ^{13}C and ^{19}F NMR Spectra of Products

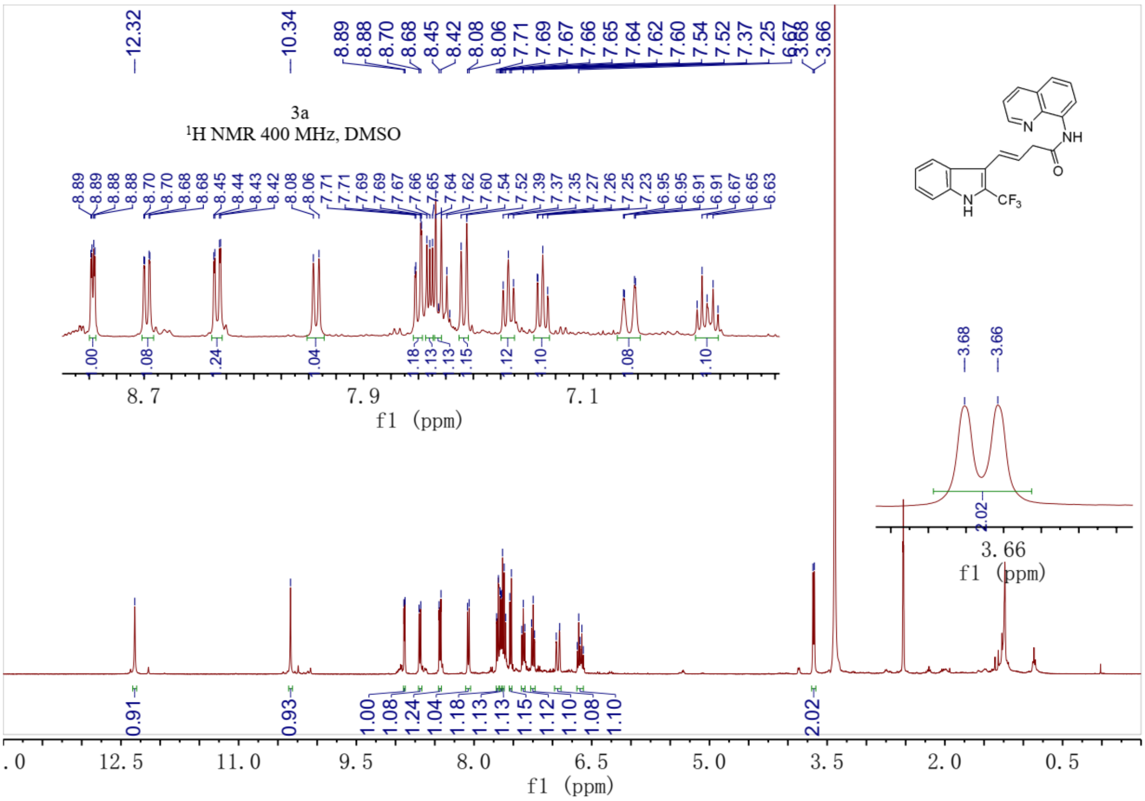

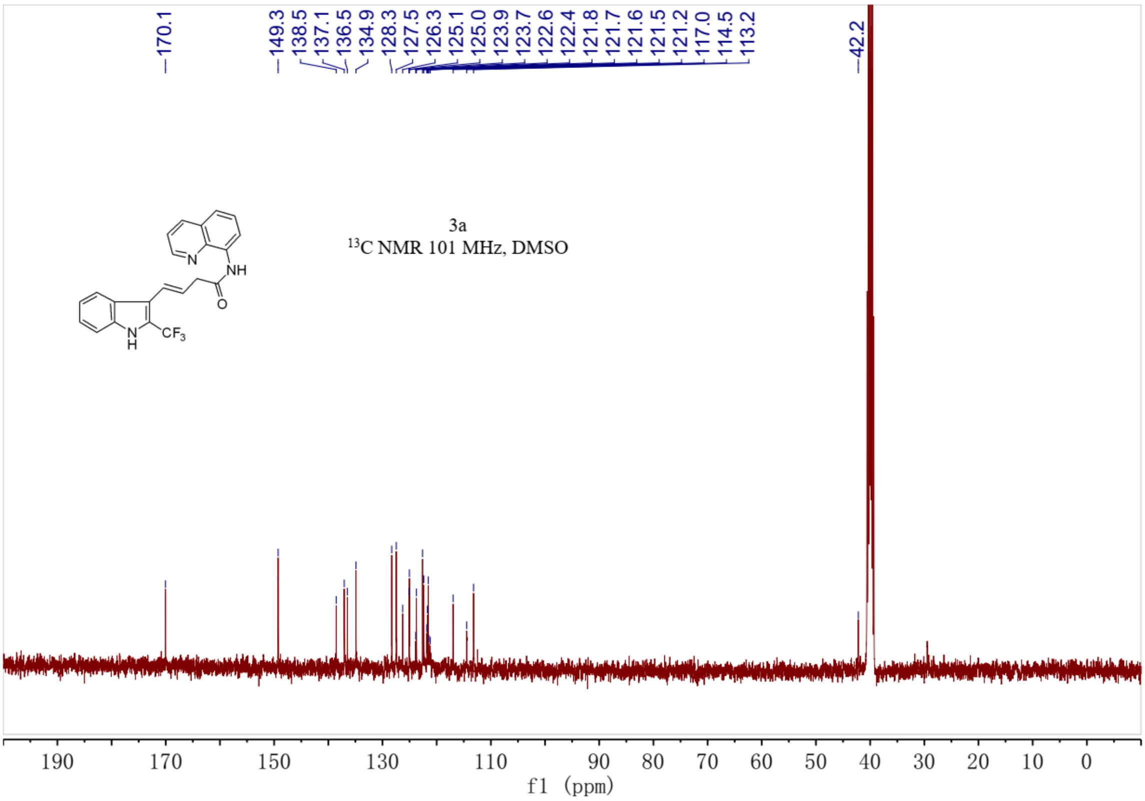

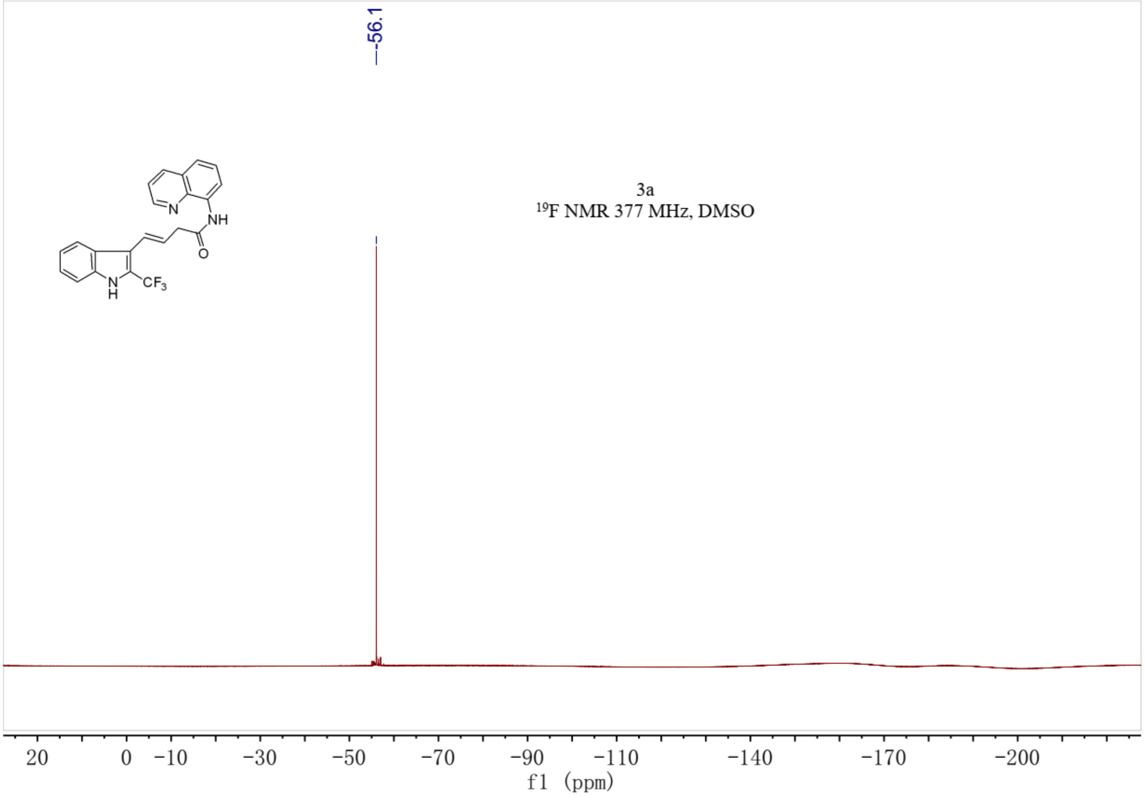

-56.1
3a
19F NMR 377 MHz, DMSO
NH
N
O
N
H
CF3
20
0
−10
−30
−50
−70
−90
−110
−140
−170
−200
f1 (ppm)

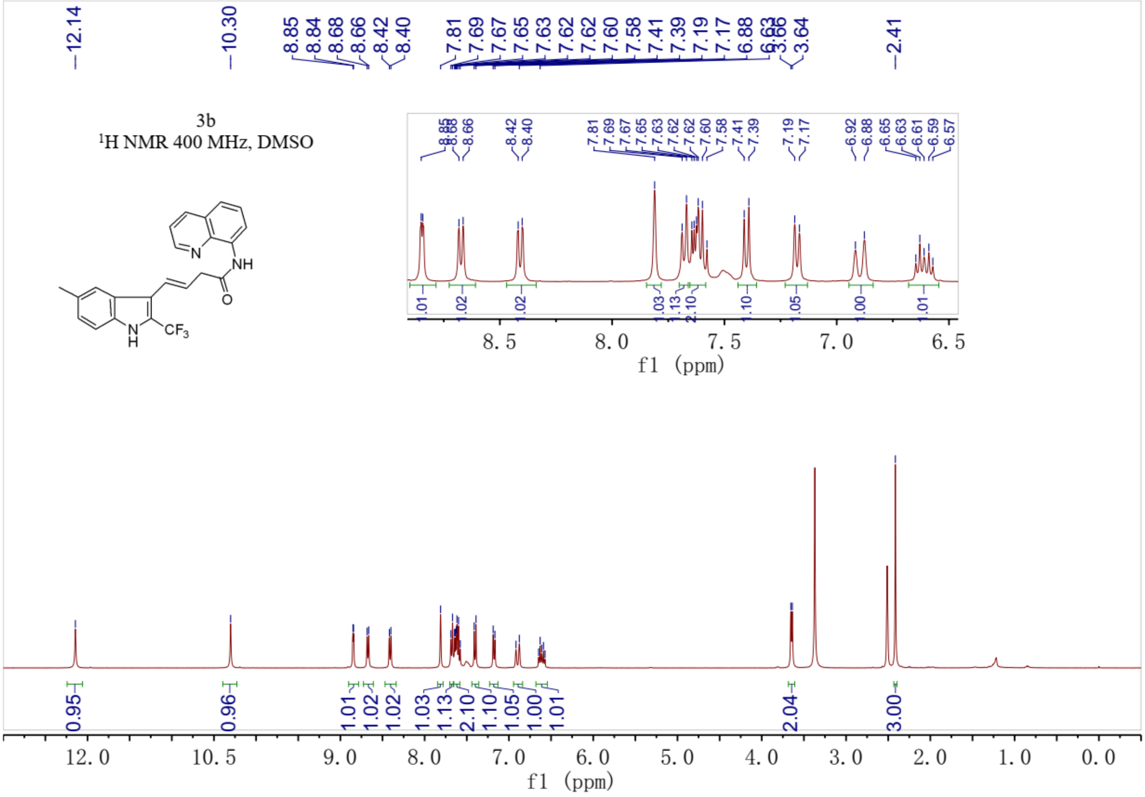

3b
1H NMR 400 MHz, DMSO
f1 (ppm)

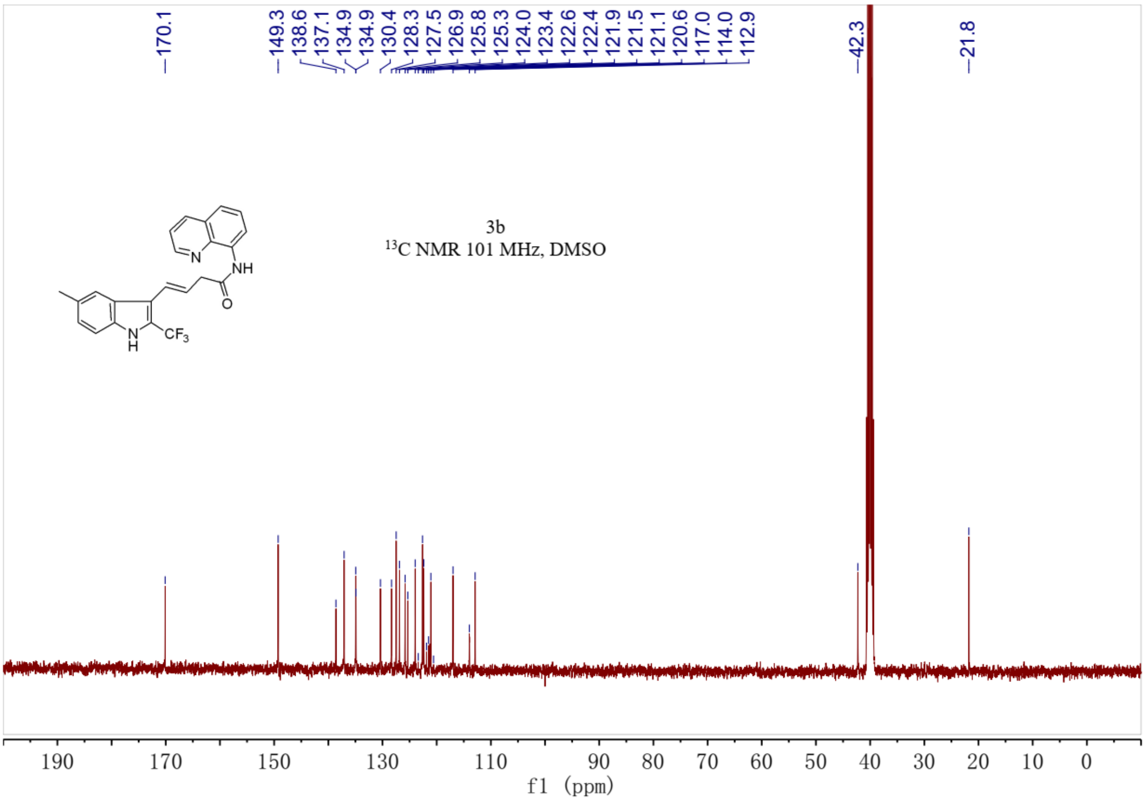

3b
13C NMR 101 MHz, DMSO
f1 (ppm)

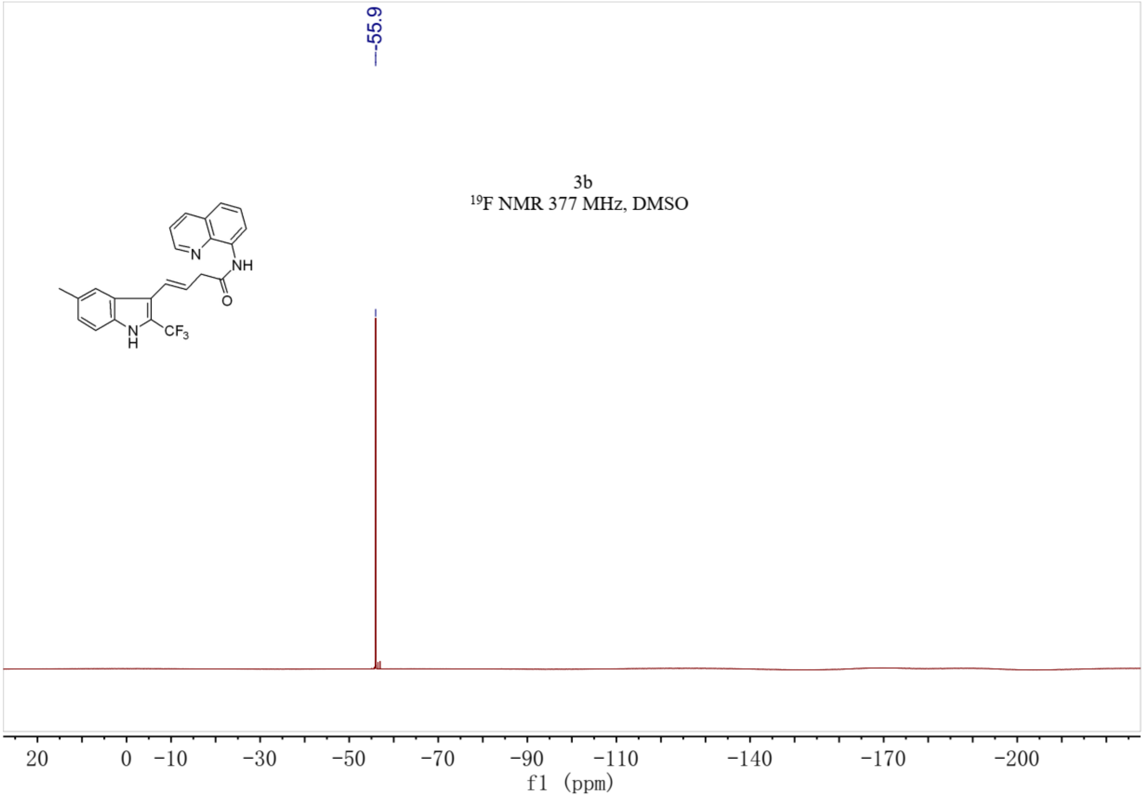
-55.9
3b
19F NMR 377 MHz, DMSO
NH
N
O
H
CF3
20
0
−10
−30
−50
−70
−90
−110
−140
−170
−200
f1 (ppm)

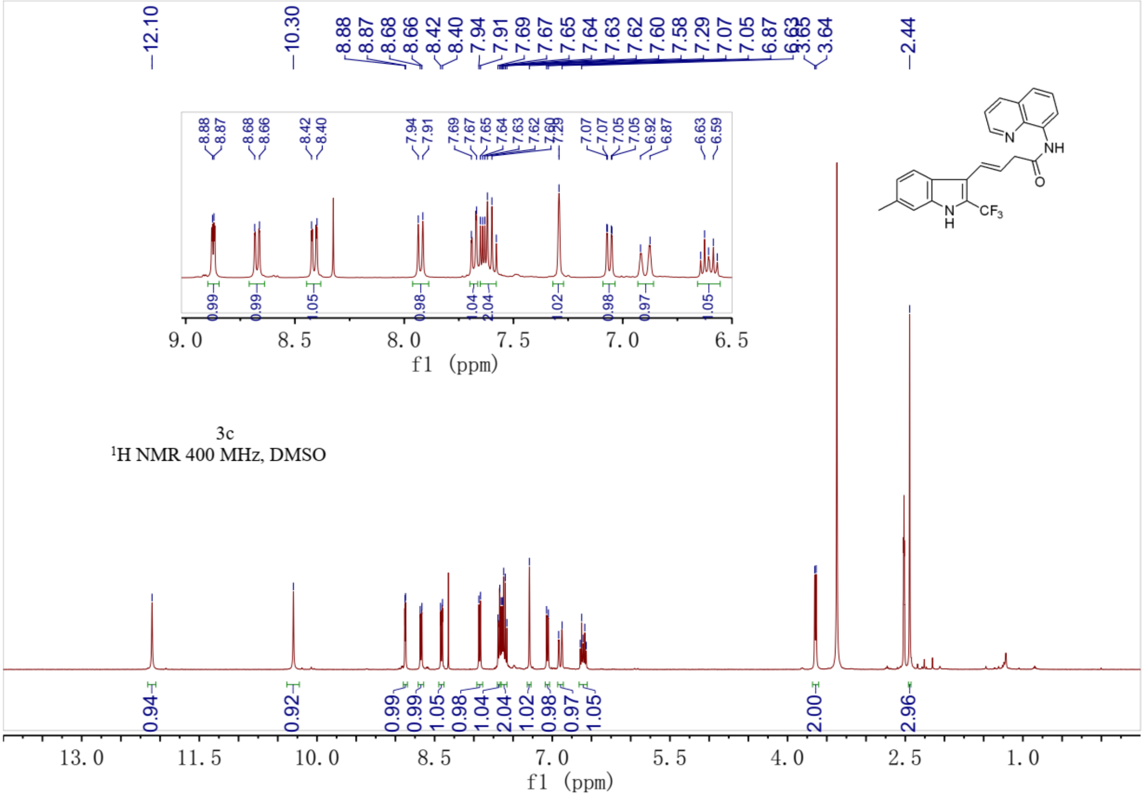
3c
^{1}H NMR 400 MHz, DMSO
f1 (ppm)

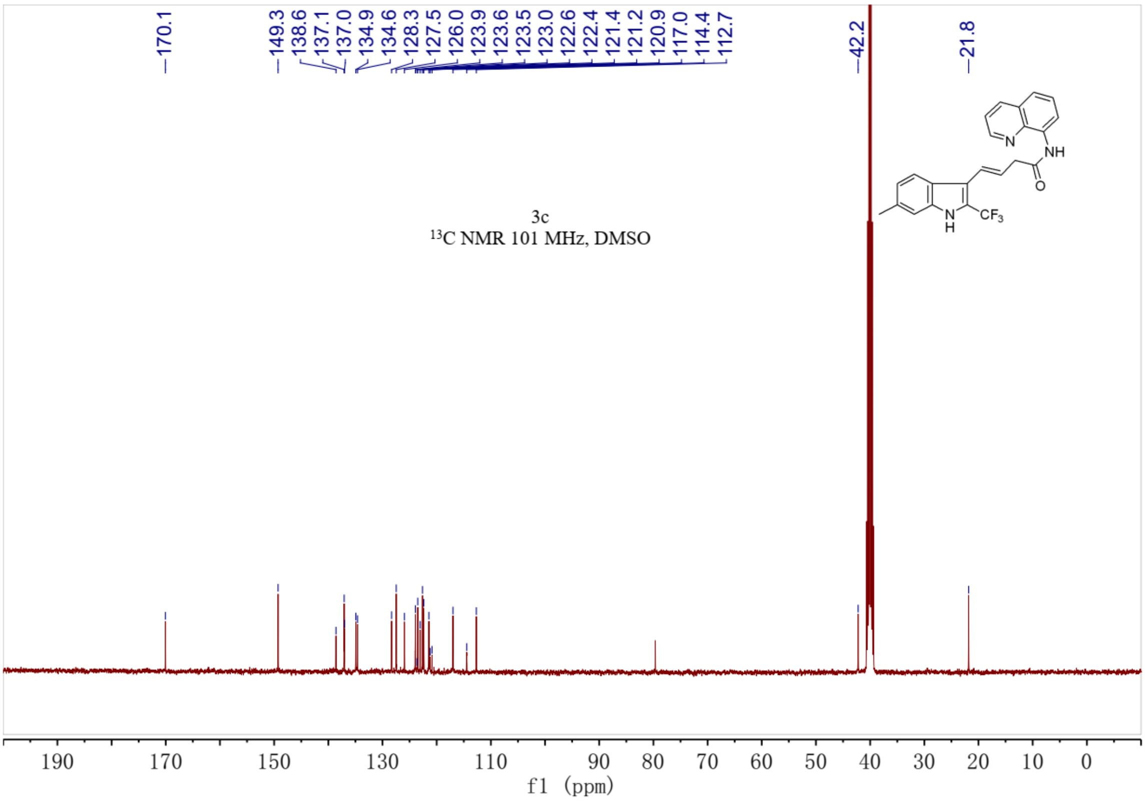
3c
^{13}C NMR 101 MHz, DMSO
f1 (ppm)

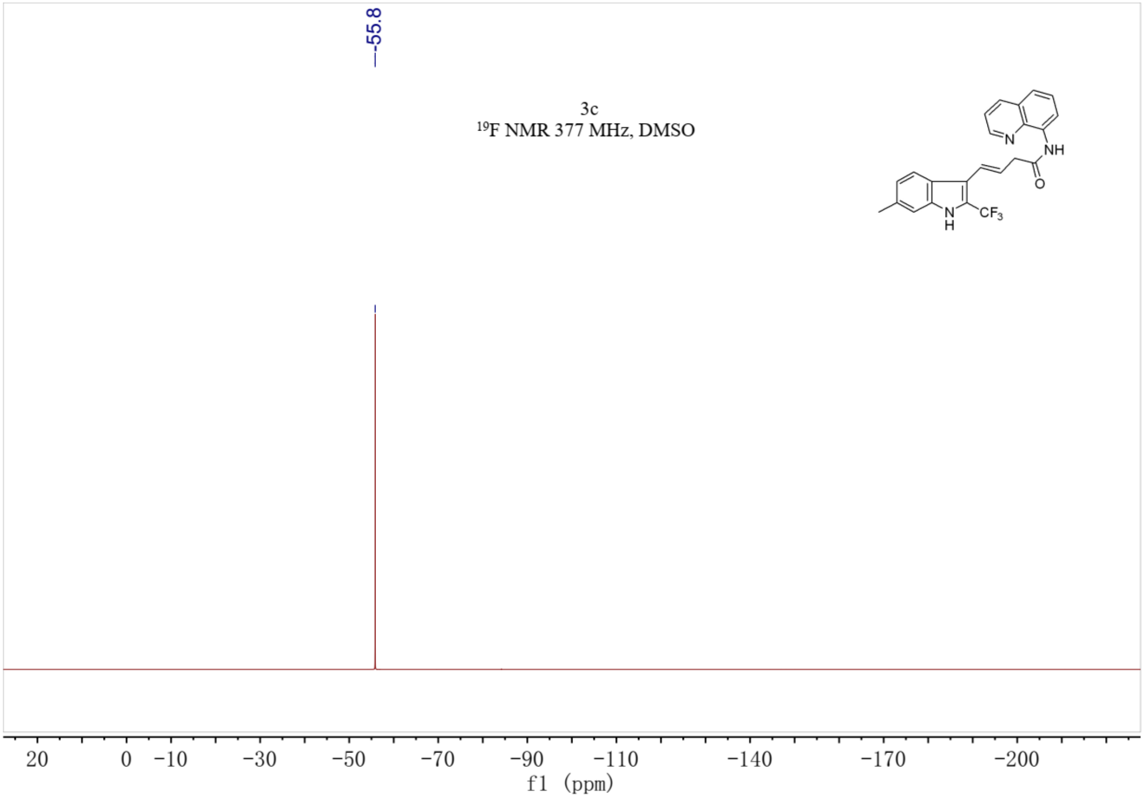

-55.8
3c
19F NMR 377 MHz, DMSO
NH
O
N
H
CF3
20
0
-10
-30
-50
-70
-90
-110
-140
-170
-200
f1 (ppm)

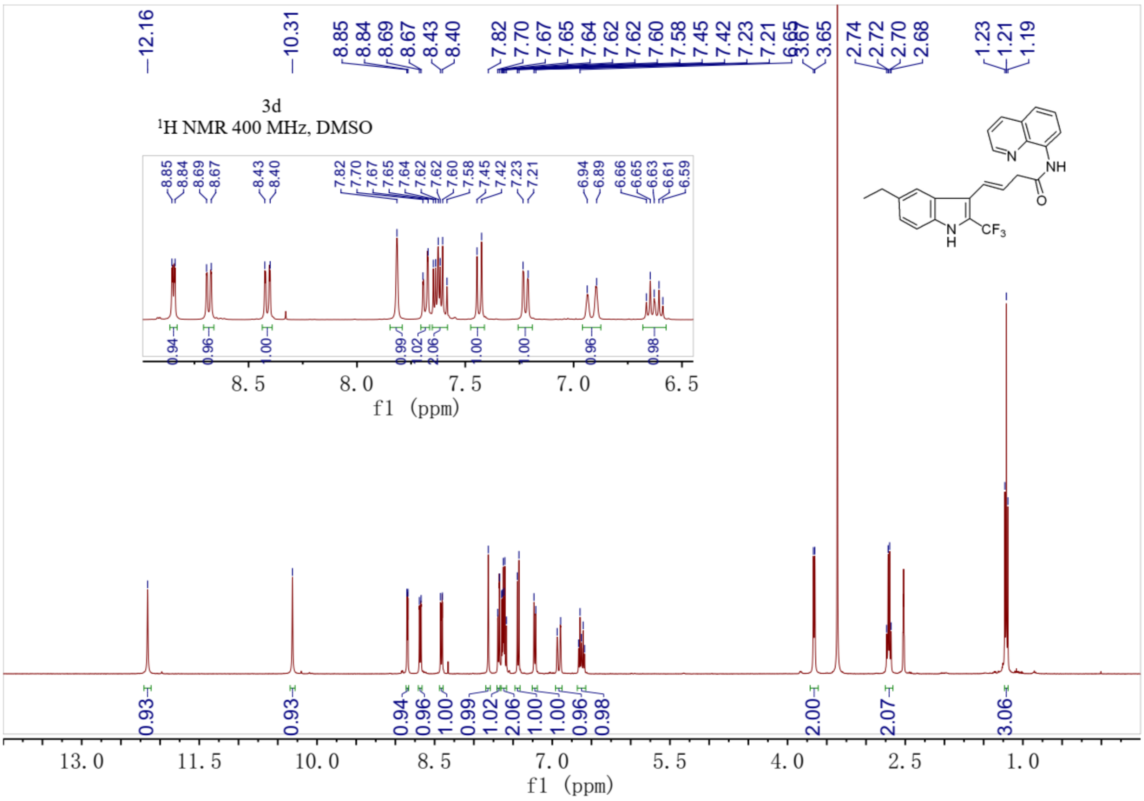
3d
^{1}H NMR 400 MHz, DMSO
f1 (ppm)

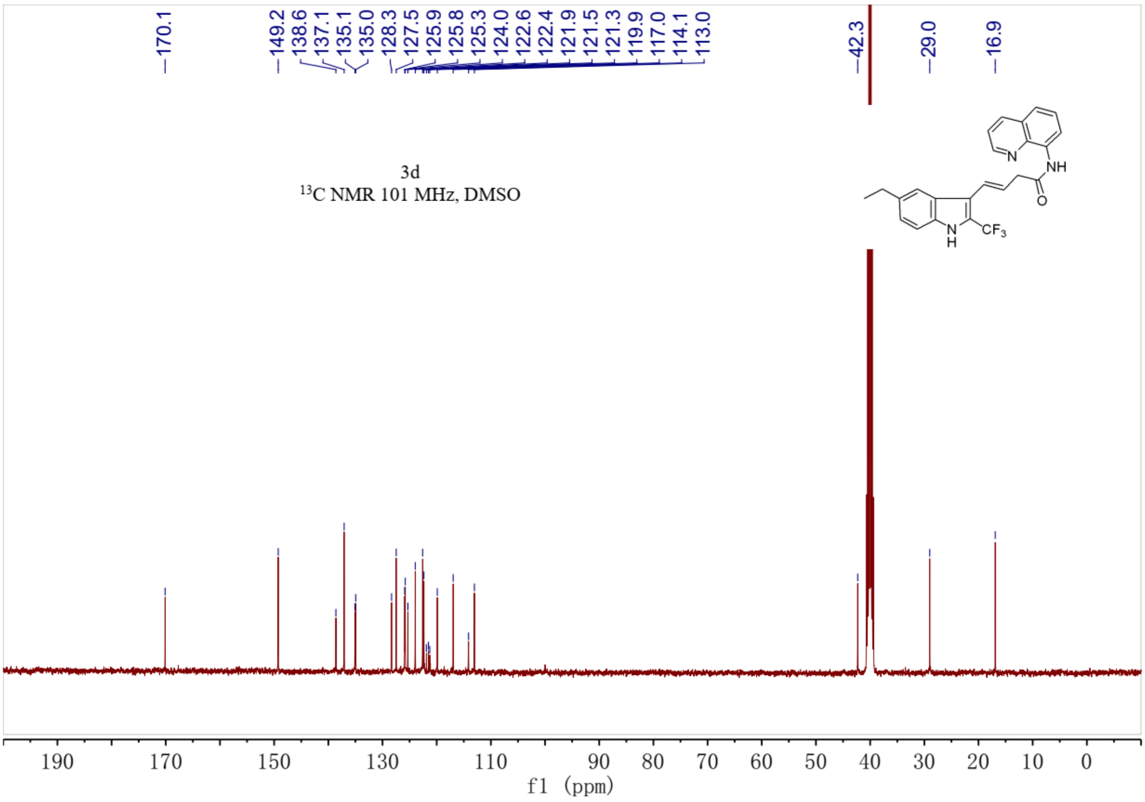
3d
^{13}C NMR 101 MHz, DMSO
f1 (ppm)

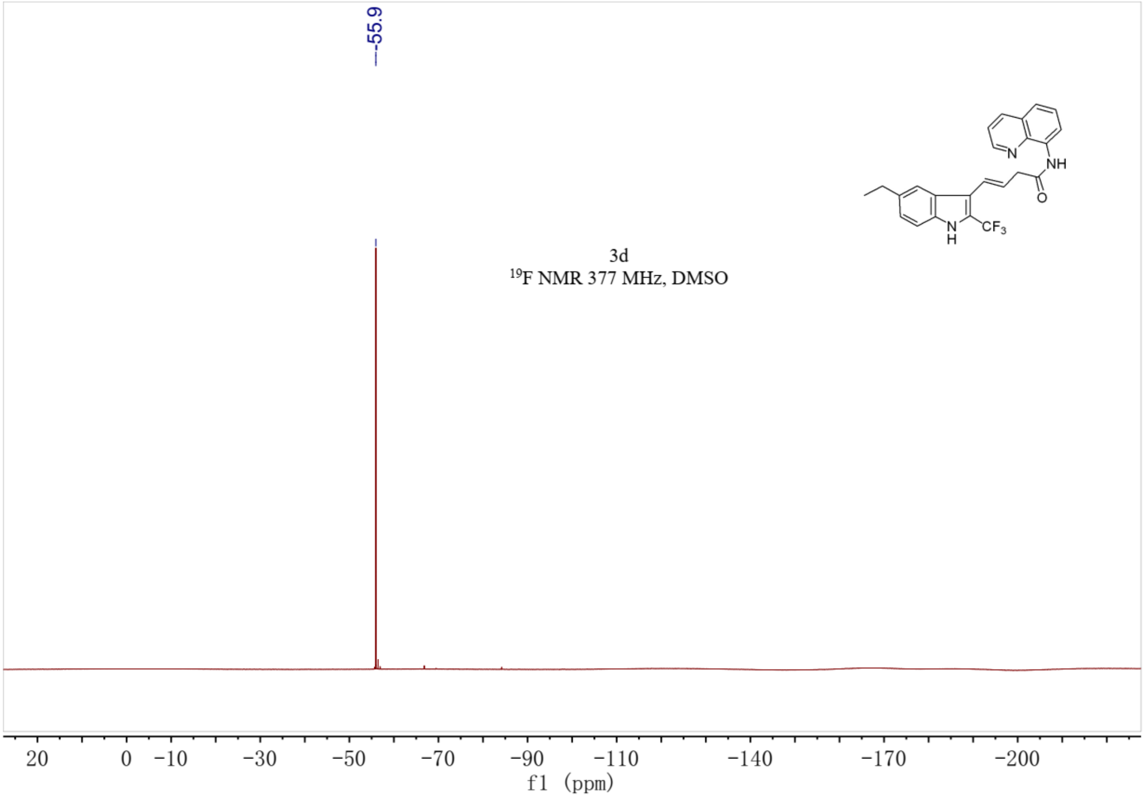

-55.9
3d
19F NMR 377 MHz, DMSO
NH
N
O
H
CF3
20
0
−10
−30
−50
−70
−90
−110
−140
−170
−200
f1 (ppm)

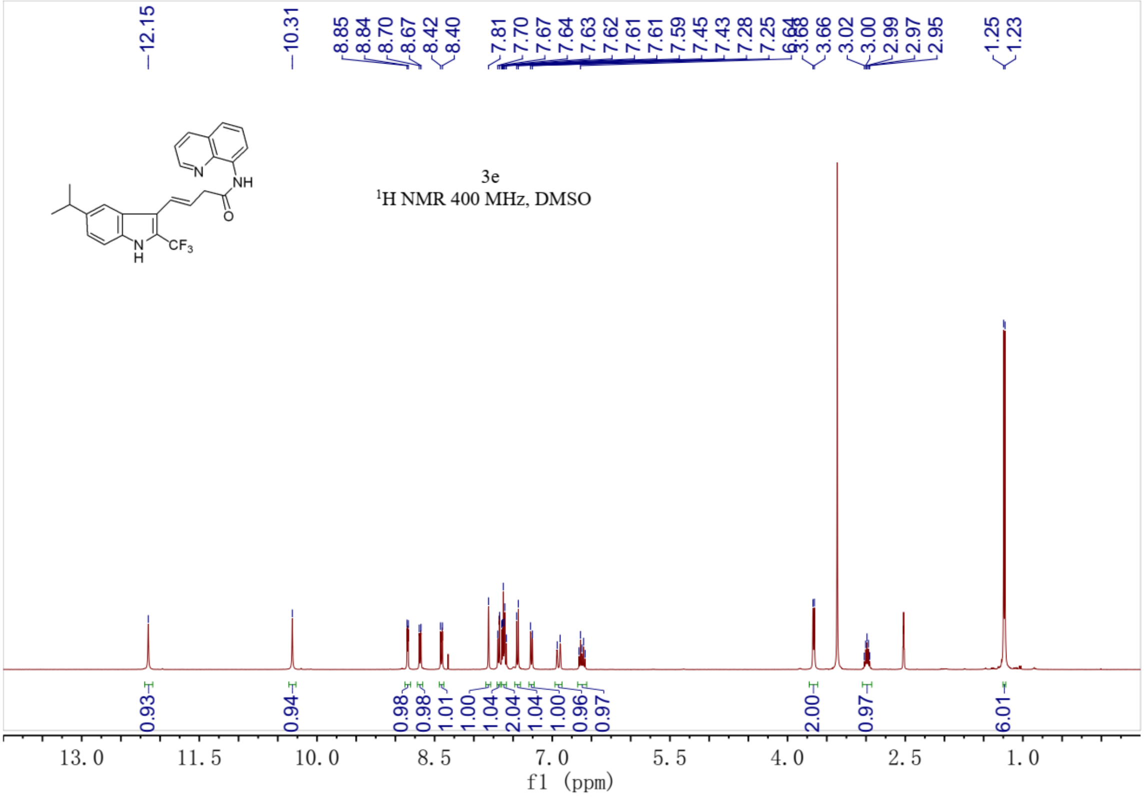
12.15
10.31
8.85
8.84
8.70
8.67
8.42
8.40
7.81
7.70
7.67
7.64
7.63
7.62
7.61
7.61
7.59
7.45
7.43
7.28
7.25
6.64
3.68
3.66
3.02
3.00
2.99
2.97
2.95
1.25
1.23
NH
N
O
N
H
CF3
3e
1H NMR 400 MHz, DMSO
0.93
0.94
0.98
0.98
1.01
1.00
1.04
2.04
1.04
1.00
0.96
0.97
2.00
0.97
6.01
13.0
11.5
10.0
8.5
7.0
5.5
4.0
2.5
1.0
f1 (ppm)

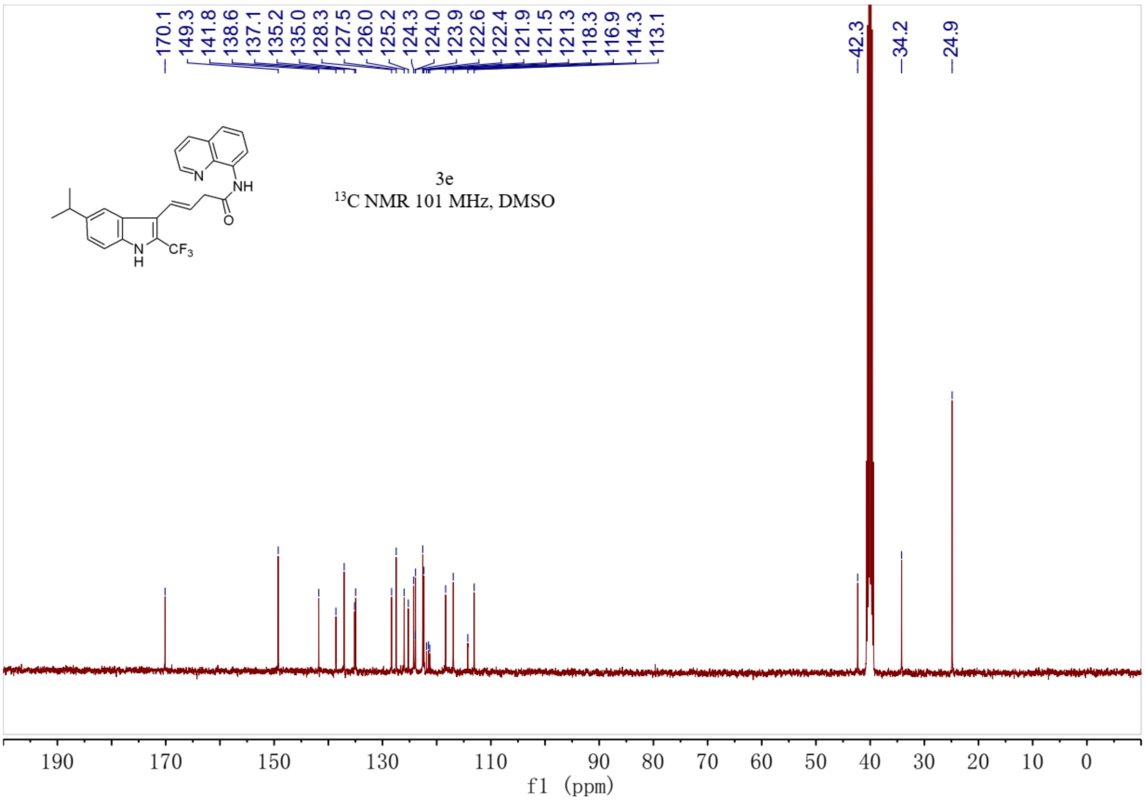
170.1
149.3
141.8
138.6
137.1
135.2
135.0
128.3
127.5
126.0
125.2
124.3
124.0
123.9
122.6
122.4
121.9
121.5
121.3
118.3
116.9
114.3
113.1
42.3
34.2
24.9
NH
N
O
N
H
CF3
3e
13C NMR 101 MHz, DMSO
190
170
150
130
110
90
80
70
60
50
40
30
20
10
0
f1 (ppm)

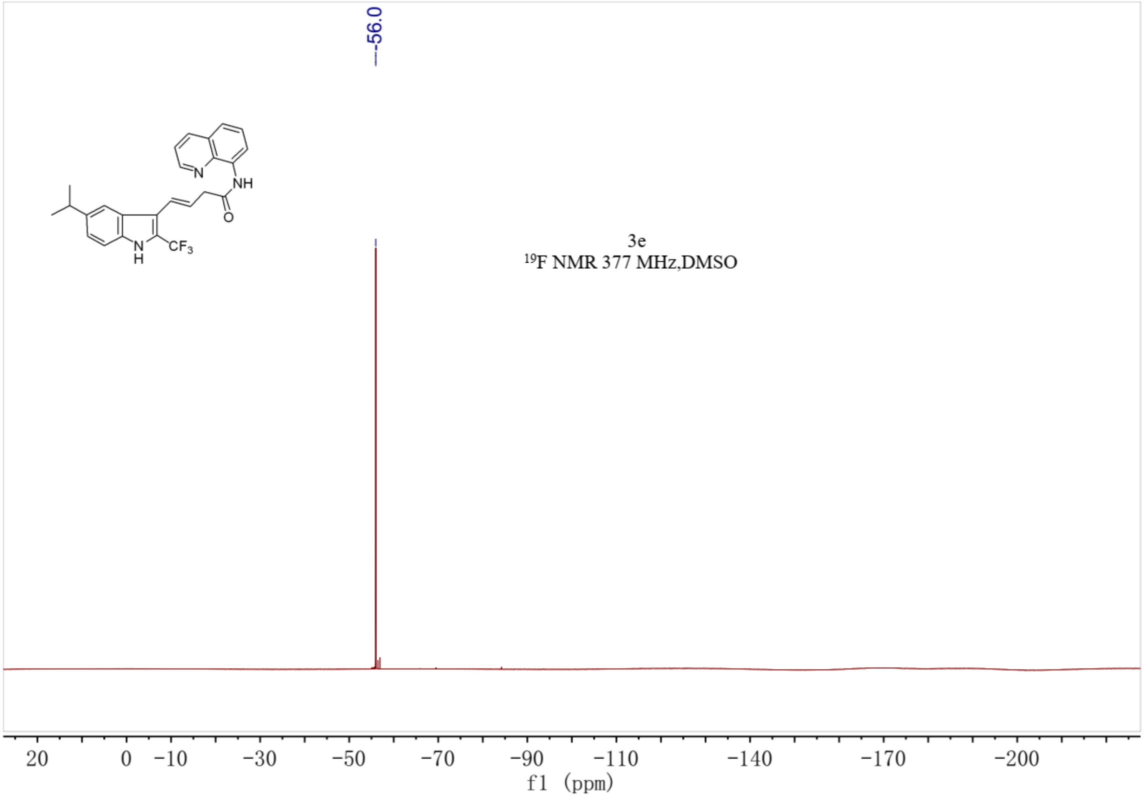
-56.0
NH
N
O
N
H
CF3
3e
19F NMR 377 MHz,DMSO
20
0
−10
−30
−50
−70
−90
−110
−140
−170
−200
f1 (ppm)

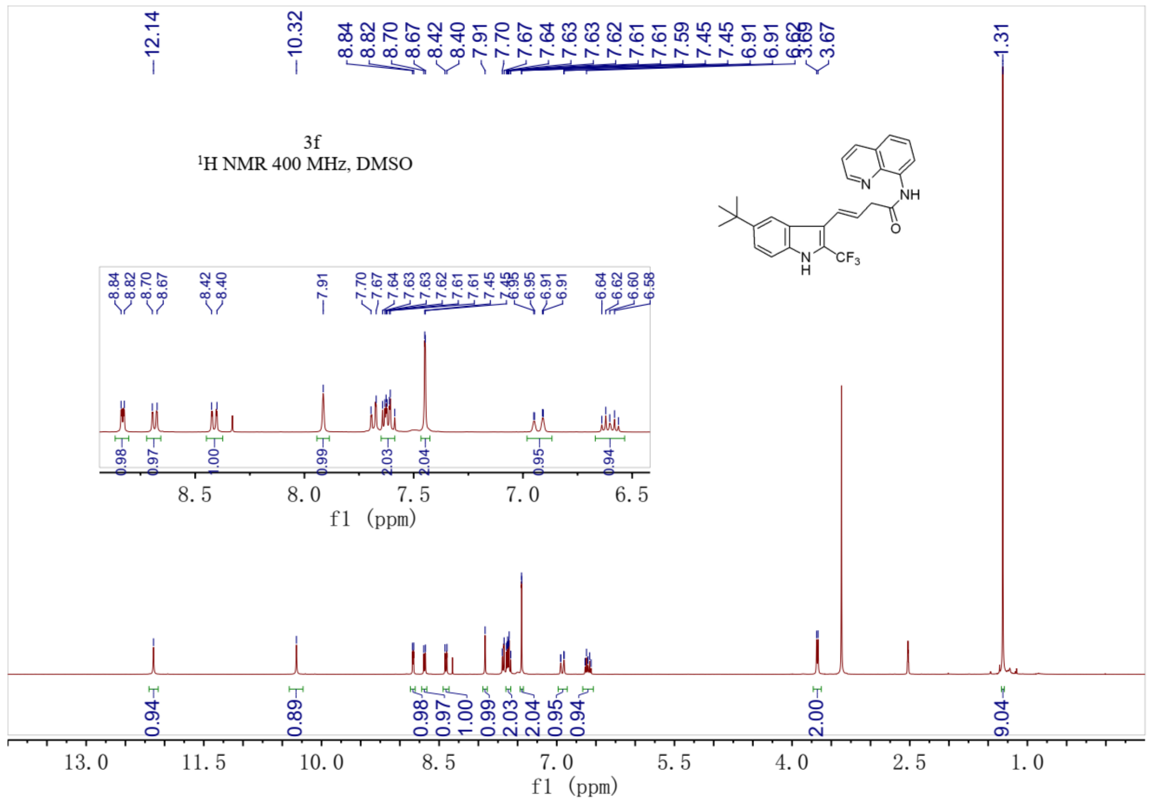
3f
1H NMR 400 MHz, DMSO
12.14
10.32
8.84 8.82 8.70 8.67 8.42 8.40 7.91 7.70 7.67 7.64 7.63 7.63 7.62 7.61 7.61 7.59 7.45 7.45 6.91 6.91 6.62 3.69 3.67
1.31
NH
O
N
H
CF3
0.94
0.89
0.98 0.97 1.00 0.99 2.03 2.04 0.95 0.94
2.00
9.04
13.0 11.5 10.0 8.5 7.0 5.5 4.0 2.5 1.0
f1 (ppm)
8.5 8.0 7.5 7.0 6.5

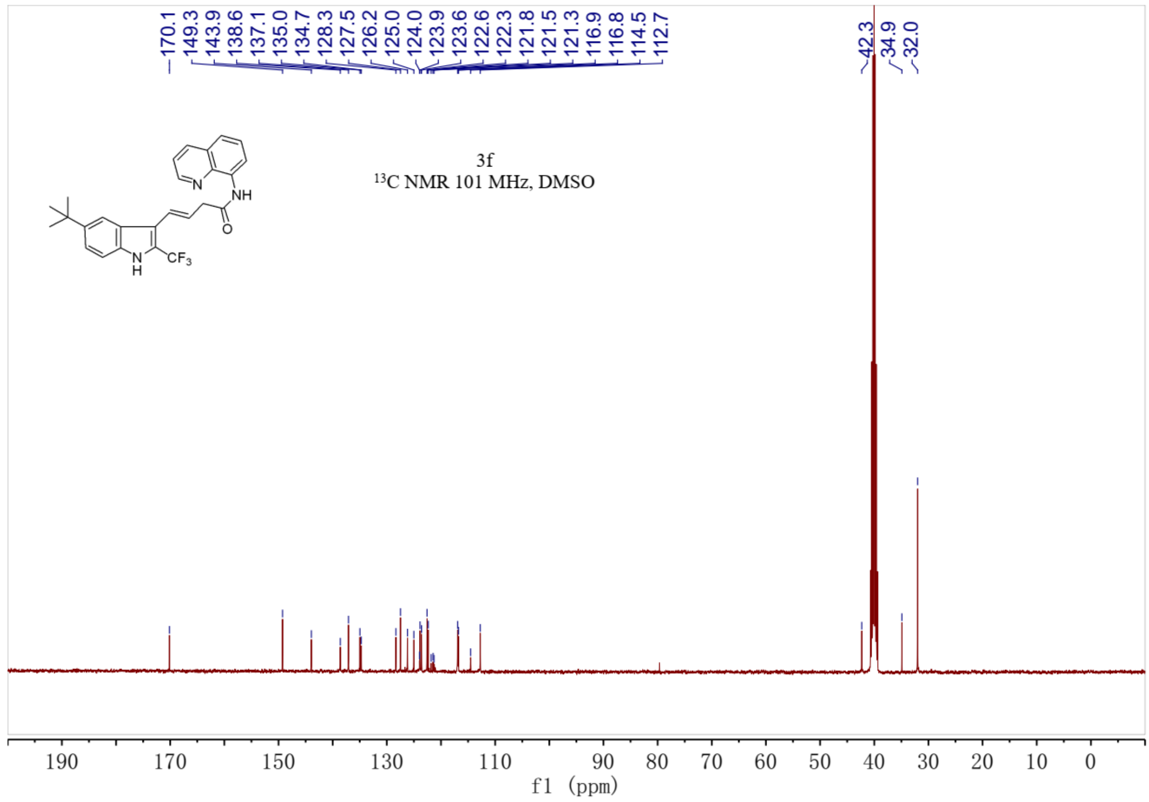
3f
13C NMR 101 MHz, DMSO
170.1
149.3 143.9 138.6 137.1 135.0 134.7 128.3 127.5 126.2 125.0 124.0 123.9 123.6 122.6 122.3 121.8 121.5 121.3 116.9 116.8 114.5 112.7
42.3
34.9
32.0
NH
O
N
H
CF3
190 170 150 130 110 90 80 70 60 50 40 30 20 10 0
f1 (ppm)

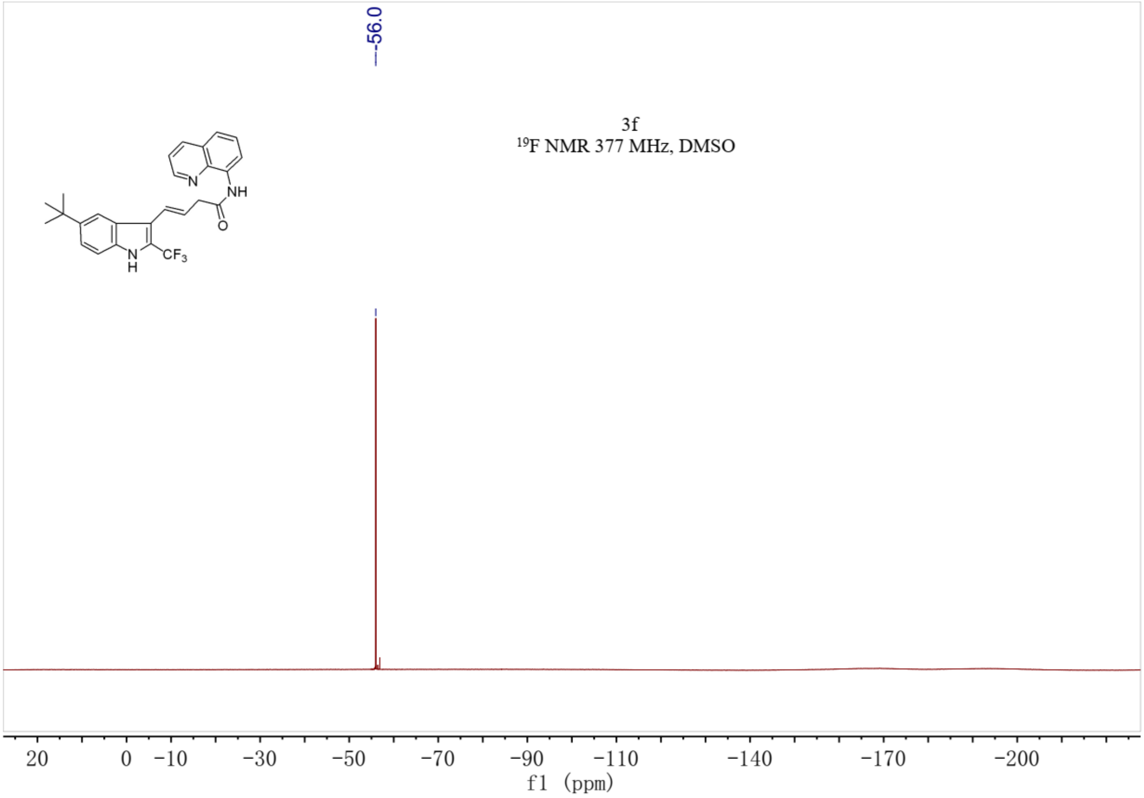

-56.0
3f
19F NMR 377 MHz, DMSO
NH
N
O
N
H
CF3
20
0
−10
−30
−50
−70
−90
−110
−140
−170
−200
f1 (ppm)

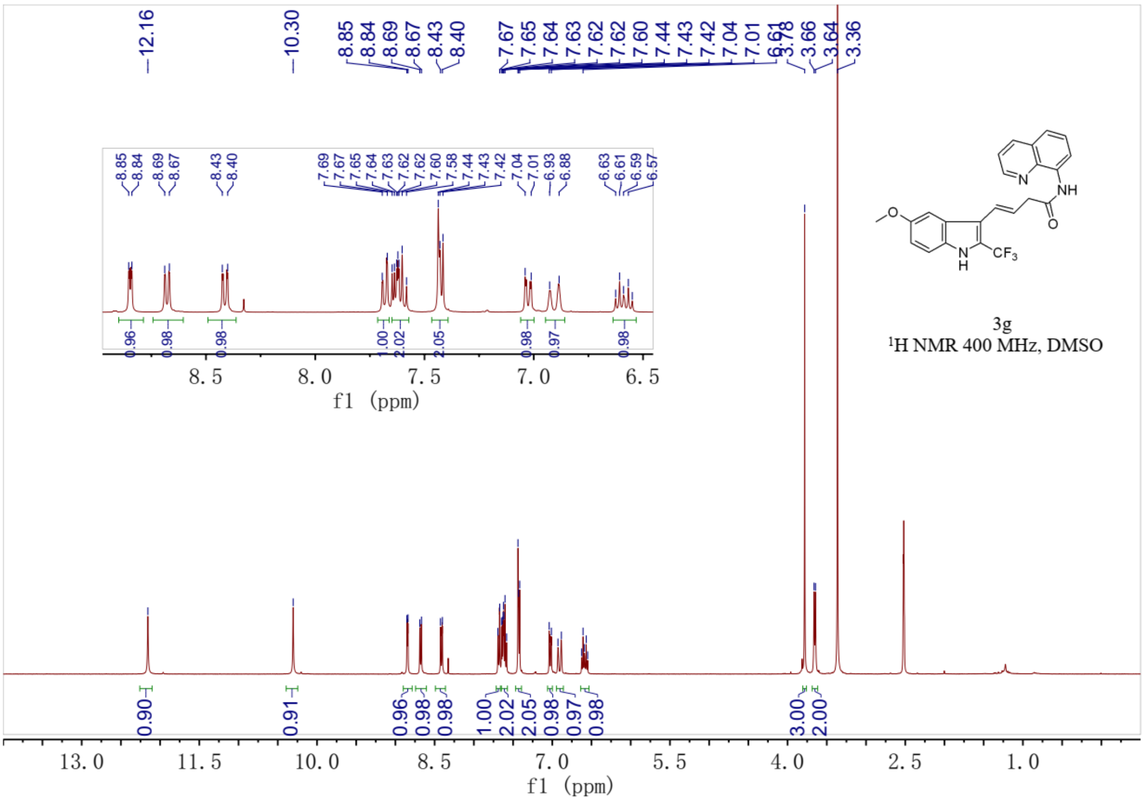
3g
^{1}H NMR 400 MHz, DMSO
f1 (ppm)

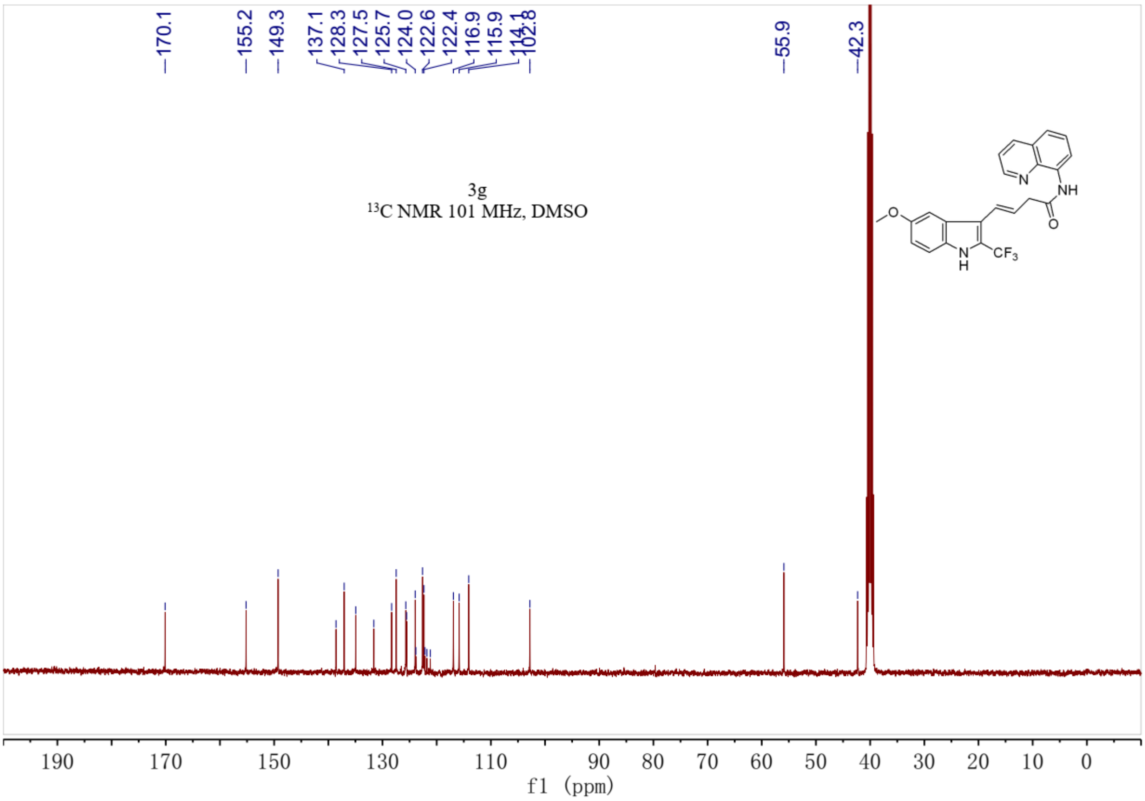
3g
^{13}C NMR 101 MHz, DMSO
f1 (ppm)

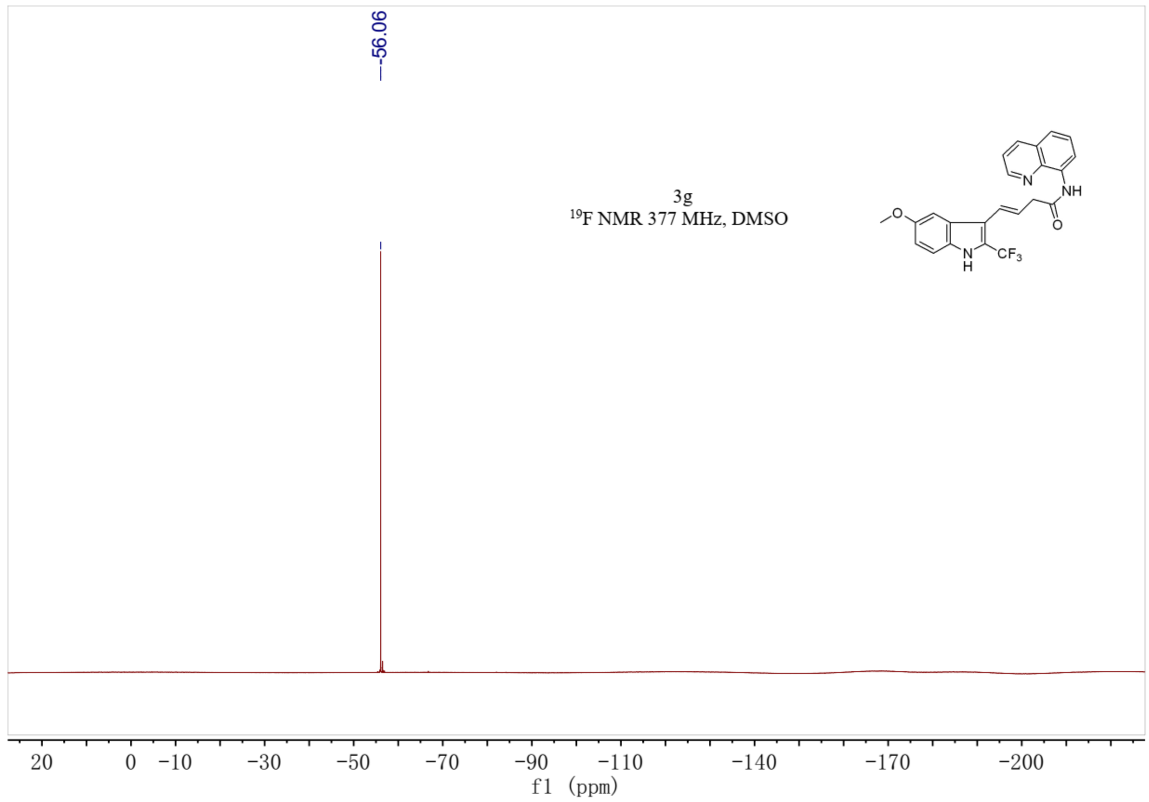
-56.06
3g
19F NMR 377 MHz, DMSO
NH
N
O
O
N
H
CF3
20
0
-10
-30
-50
-70
-90
-110
-140
-170
-200
f1 (ppm)

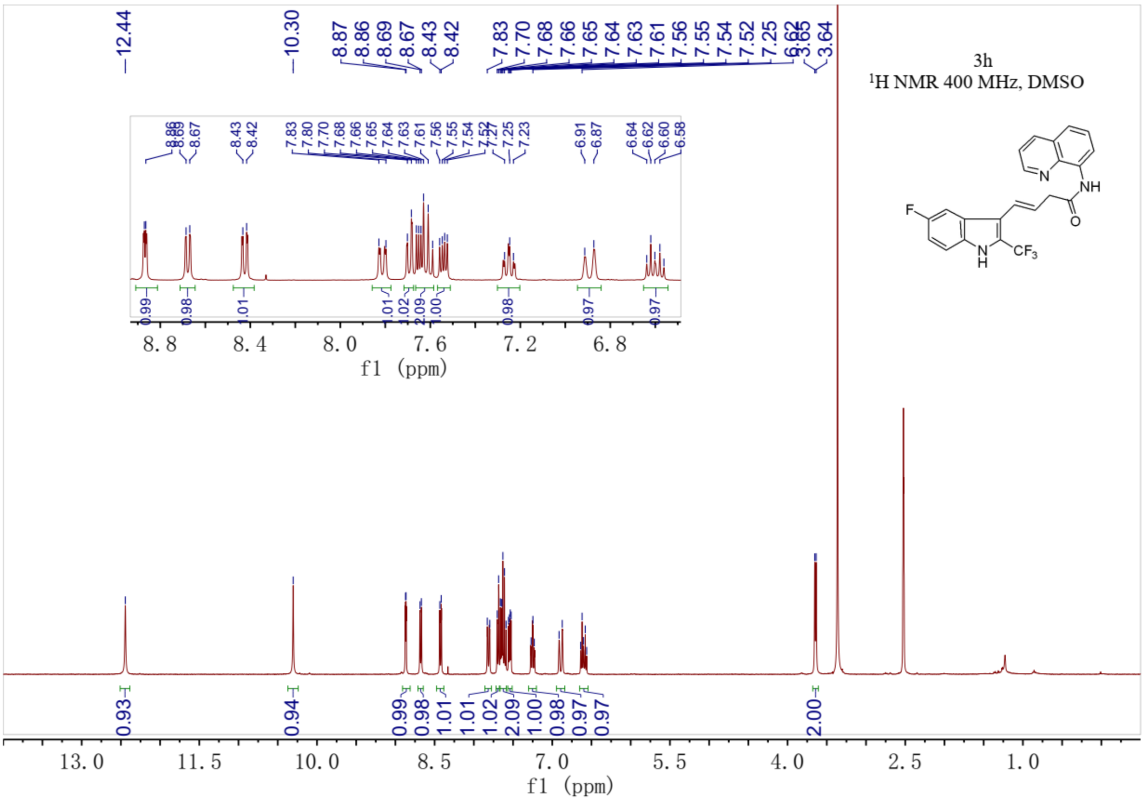
3h
¹H NMR 400 MHz, DMSO
f1 (ppm)

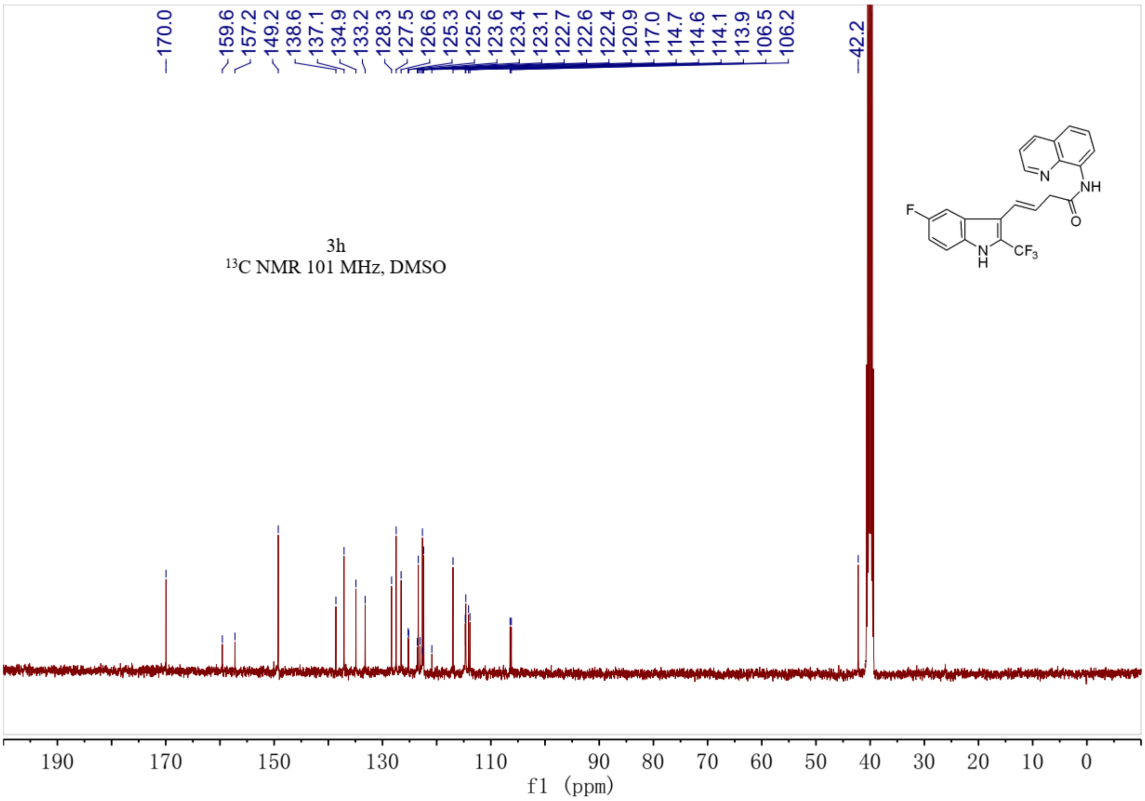
3h
¹³C NMR 101 MHz, DMSO
f1 (ppm)

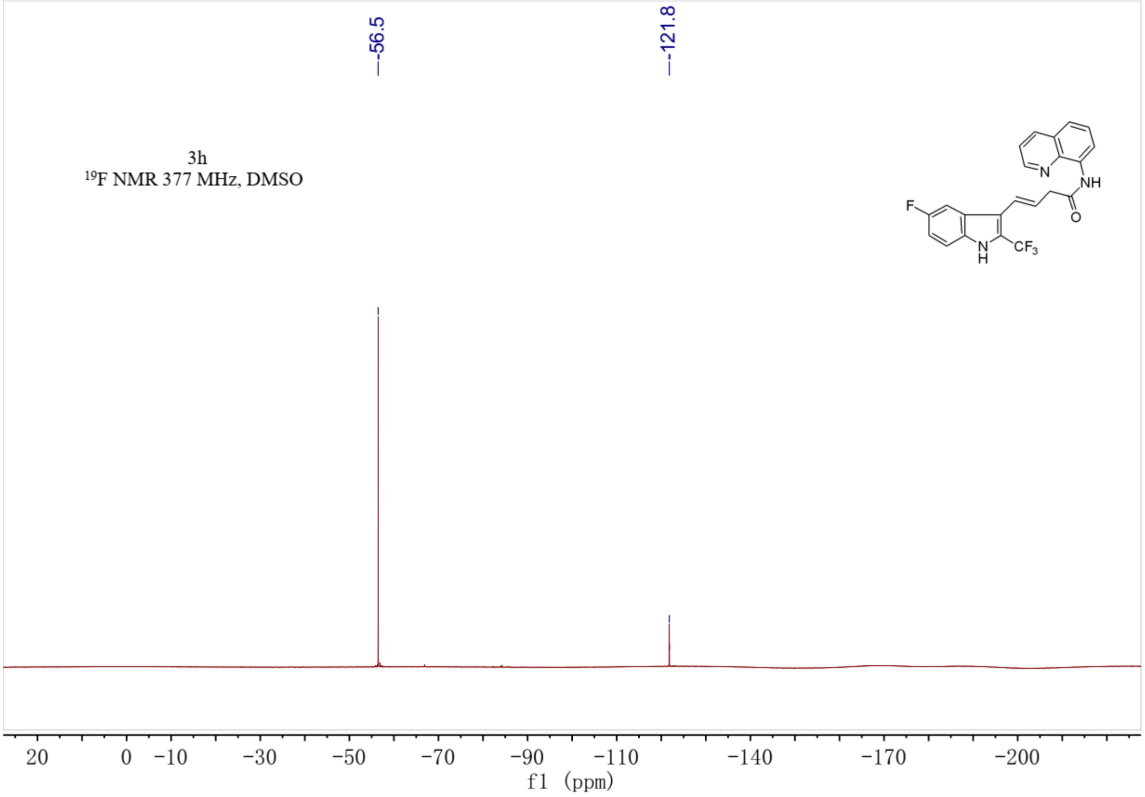
-56.5
-121.8
3h
19F NMR 377 MHz, DMSO
F
NH
N
O
N
H
CF3
20
0
−10
−30
−50
−70
−90
−110
−140
−170
−200
f1 (ppm)

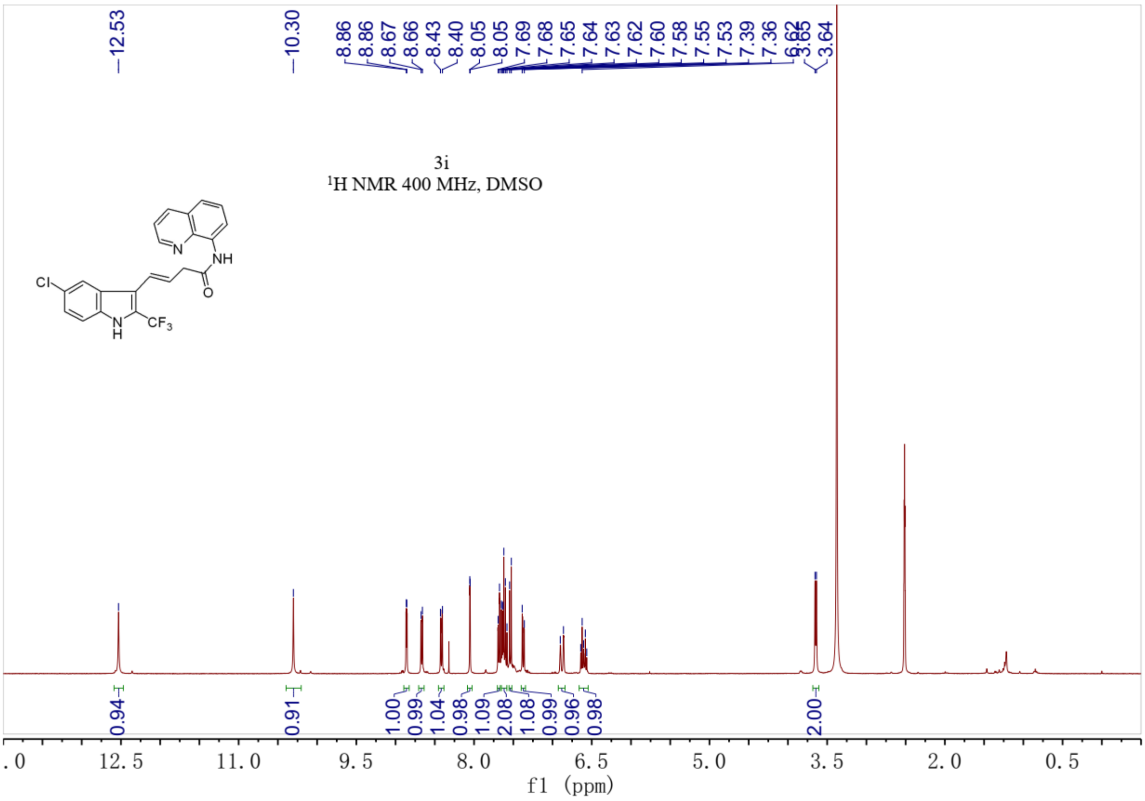
3i
^{1}H NMR 400 MHz, DMSO
12.53
10.30
8.86
8.86
8.67
8.66
8.43
8.40
8.05
8.05
7.69
7.68
7.65
7.64
7.63
7.62
7.60
7.58
7.55
7.53
7.39
7.36
3.65
3.64
0.94
0.91
1.00
0.99
1.04
0.98
1.09
2.08
1.08
0.99
0.96
0.98
2.00
Cl
NH
N
O
N
H
CF_3
f1 (ppm)

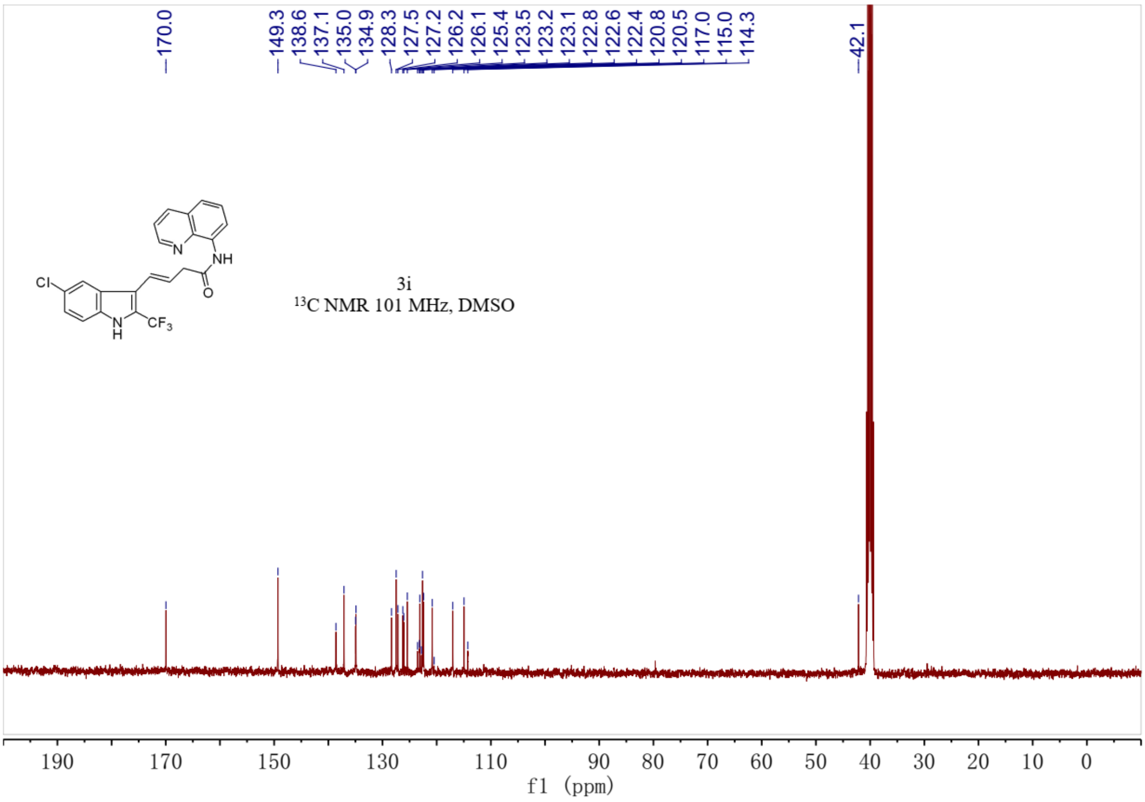
3i
^{13}C NMR 101 MHz, DMSO
170.0
149.3
138.6
137.1
135.0
134.9
128.3
127.5
127.2
126.2
126.1
125.4
123.5
123.2
123.1
122.8
122.6
122.4
120.8
120.5
117.0
115.0
114.3
42.1
Cl
NH
N
O
N
H
CF_3
f1 (ppm)

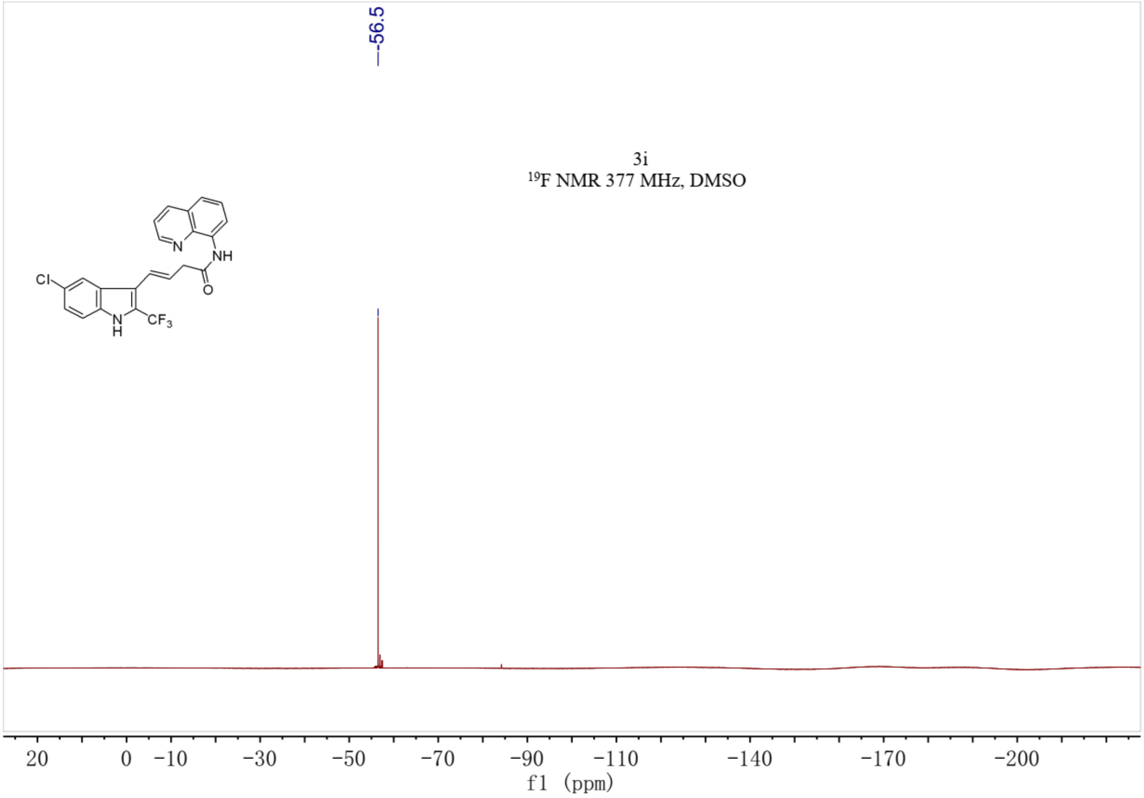

-56.5
3i
19F NMR 377 MHz, DMSO
Cl
NH
N
O
N
H
CF3
20
0
−10
−30
−50
−70
−90
−110
−140
−170
−200
f1 (ppm)

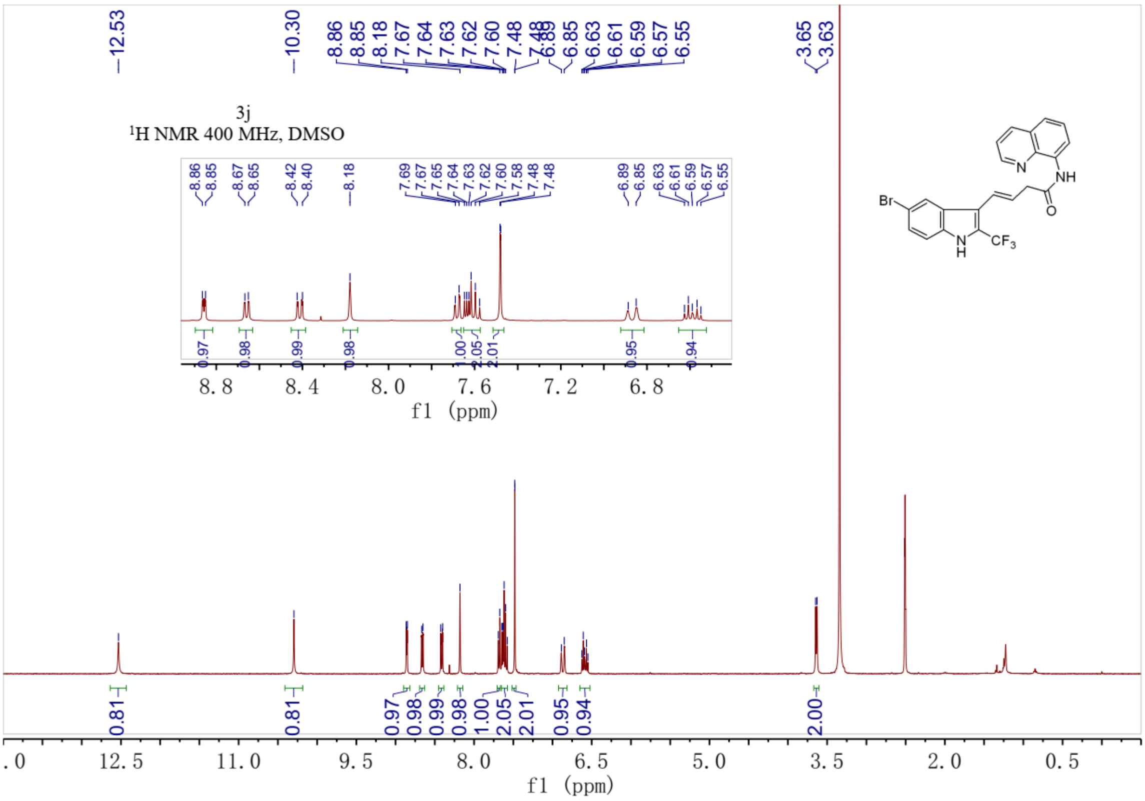
3j
¹H NMR 400 MHz, DMSO
f1 (ppm)

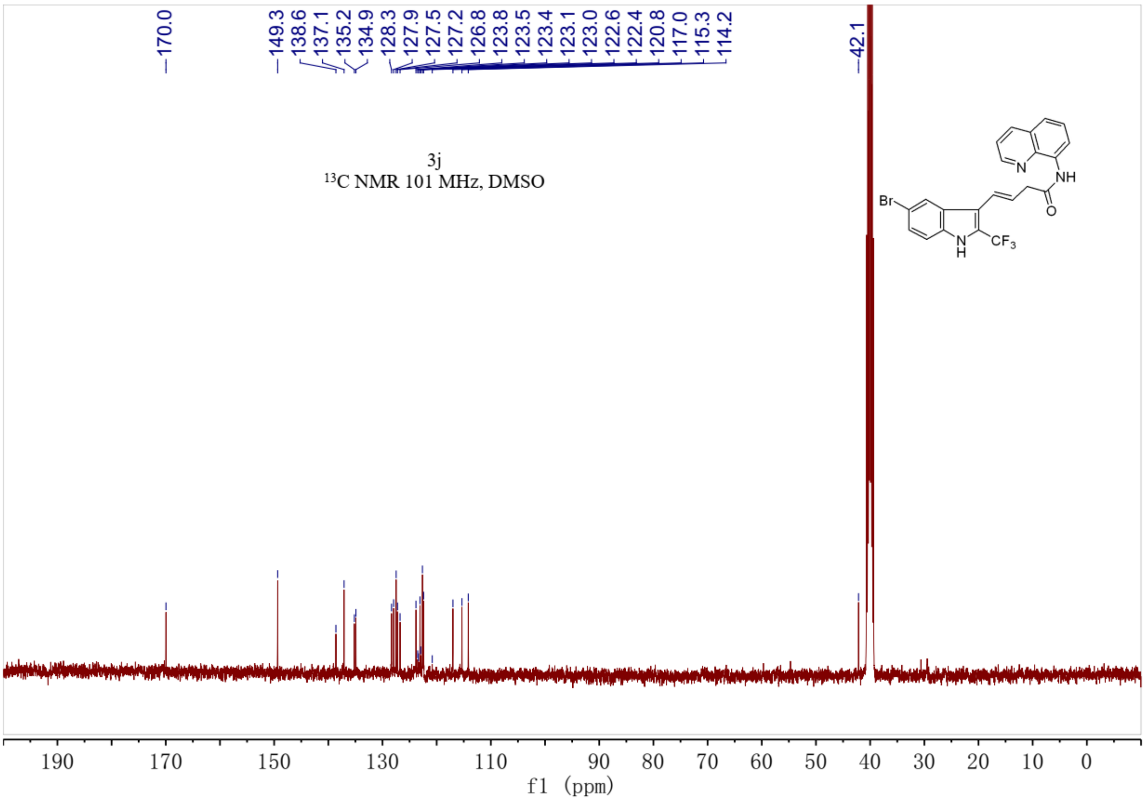
3j
¹³C NMR 101 MHz, DMSO
f1 (ppm)

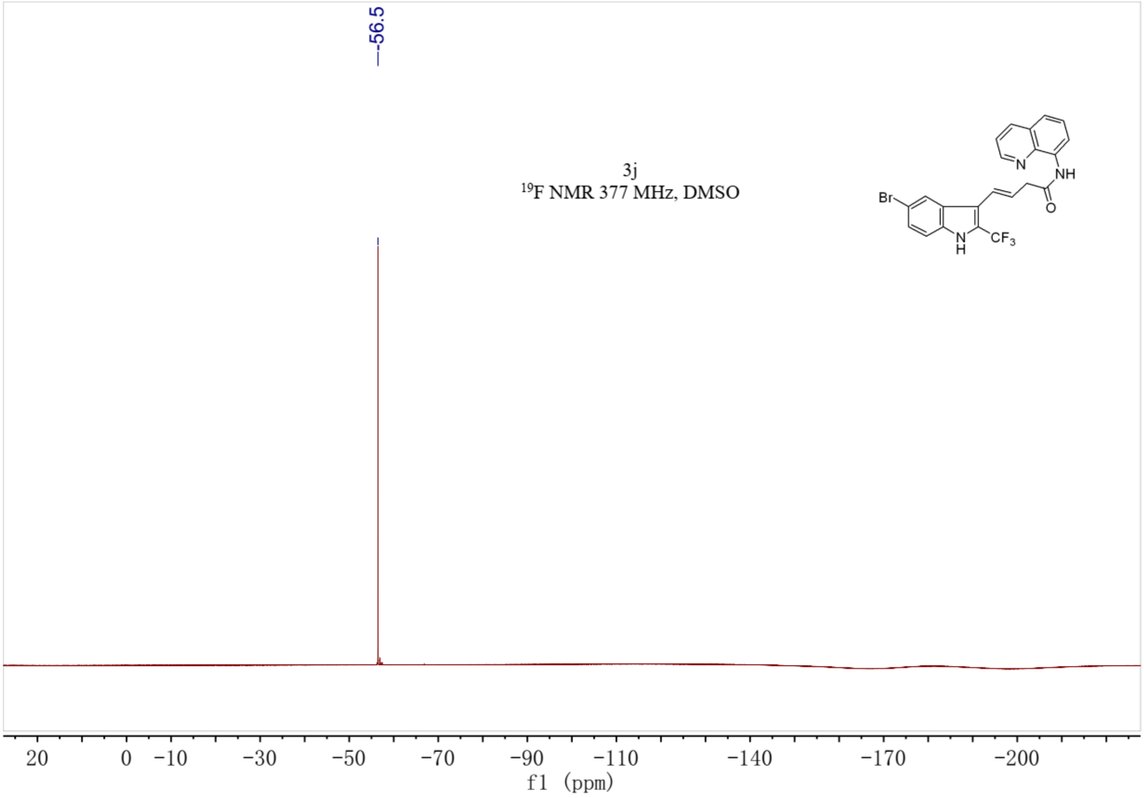

-56.5
3j
19F NMR 377 MHz, DMSO
Br
N
NH
O
N
H
CF3
20
0
−10
−30
−50
−70
−90
−110
−140
−170
−200
f1 (ppm)

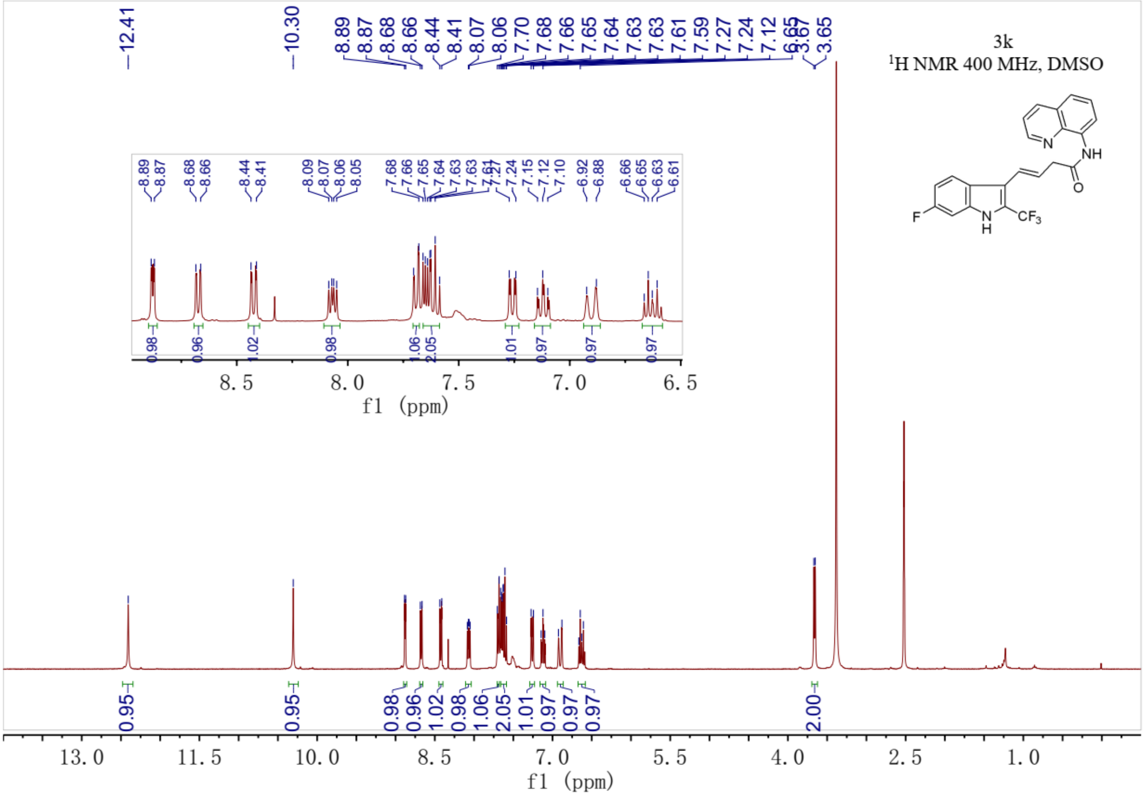
3k
^{1}H NMR 400 MHz, DMSO
f1 (ppm)

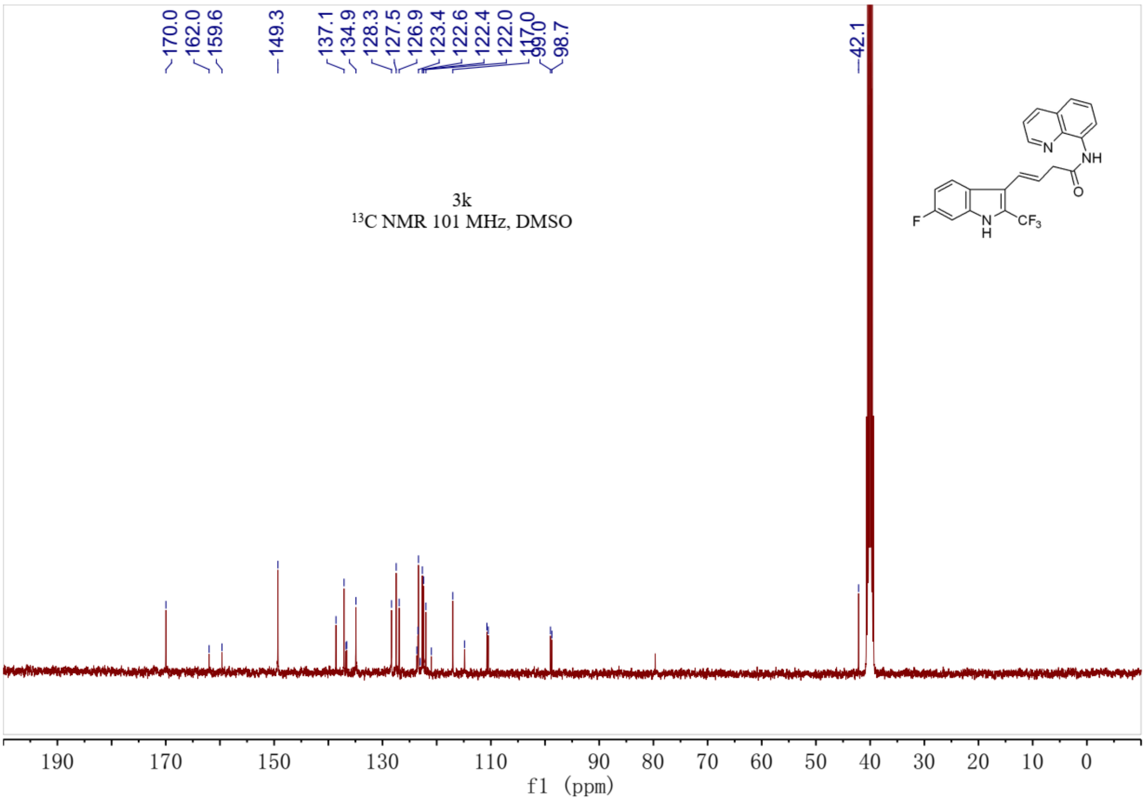
3k
^{13}C NMR 101 MHz, DMSO
f1 (ppm)

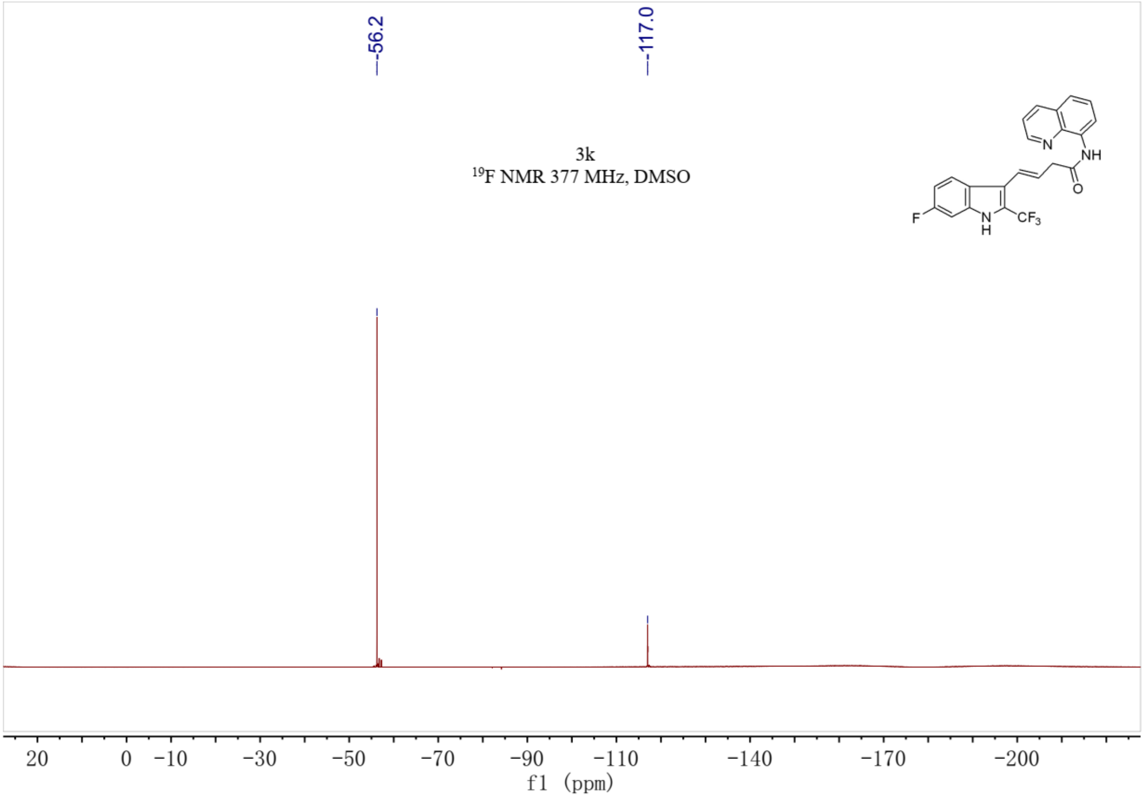

-56.2
-117.0
3k
19F NMR 377 MHz, DMSO
F
N
H
CF3
NH
O
N
20
0
−10
−30
−50
−70
−90
−110
−140
−170
−200
f1 (ppm)

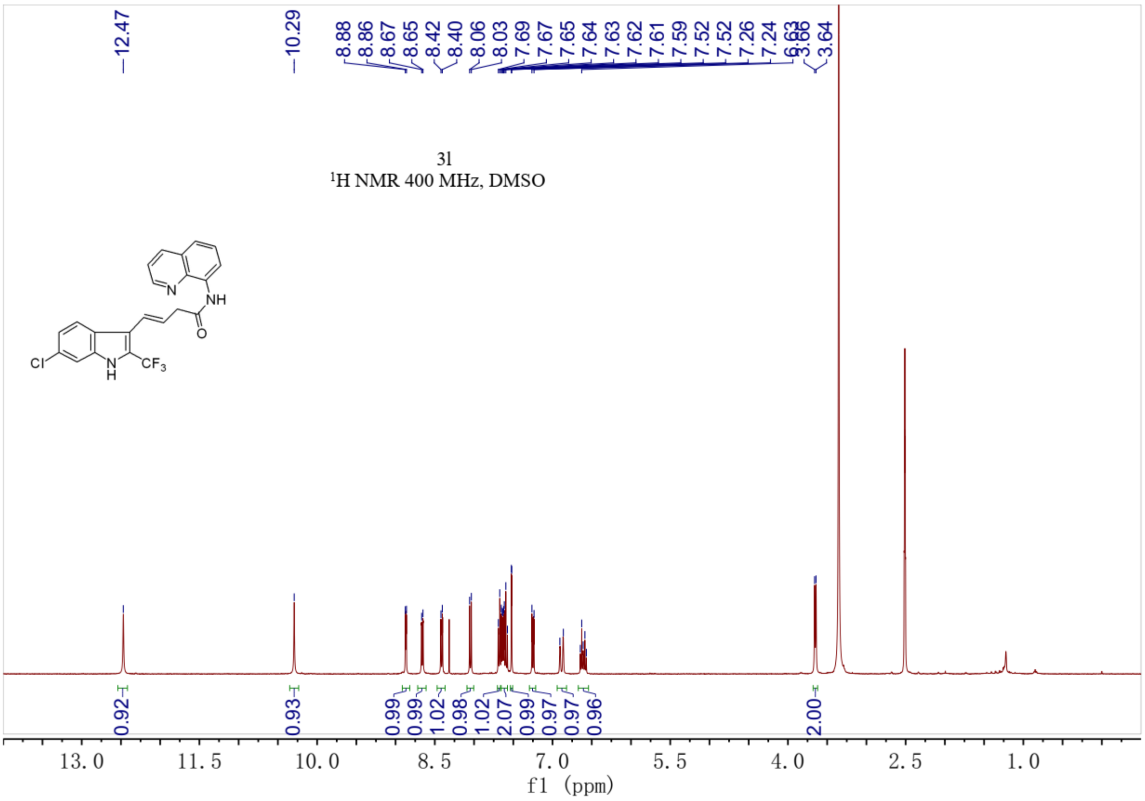
12.47
10.29
8.88
8.86
8.67
8.65
8.42
8.40
8.06
8.03
7.69
7.67
7.65
7.64
7.63
7.62
7.61
7.59
7.52
7.52
7.26
7.24
6.63
3.66
3.64
31
^{1}H NMR 400 MHz, DMSO
NH
N
O
Cl
N
H
CF_3
0.92
0.93
0.99
0.99
1.02
0.98
1.02
2.07
0.99
0.97
0.97
0.96
2.00
13.0
11.5
10.0
8.5
7.0
5.5
4.0
2.5
1.0
f1 (ppm)

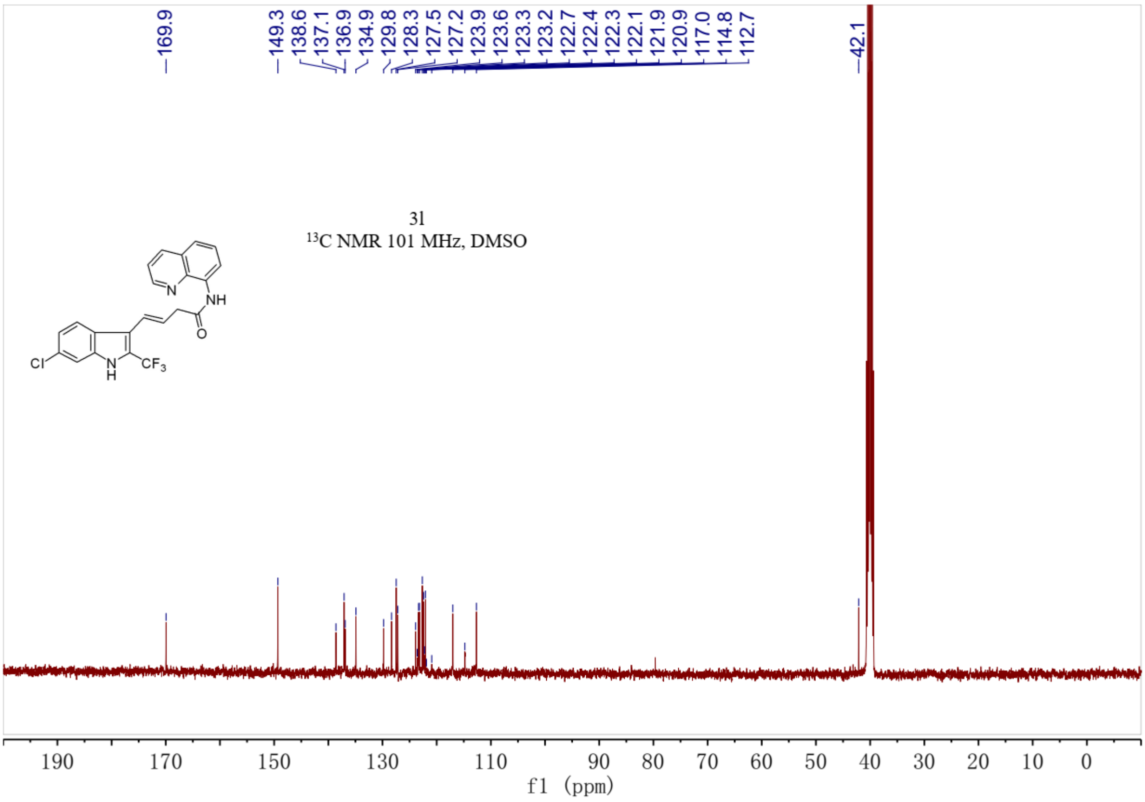
169.9
149.3
138.6
137.1
136.9
134.9
129.8
128.3
127.5
127.2
123.9
123.6
123.3
123.2
122.7
122.4
122.3
122.1
121.9
120.9
117.0
114.8
112.7
42.1
31
^{13}C NMR 101 MHz, DMSO
NH
N
O
Cl
N
H
CF_3
190
170
150
130
110
90
80
70
60
50
40
30
20
10
0
f1 (ppm)

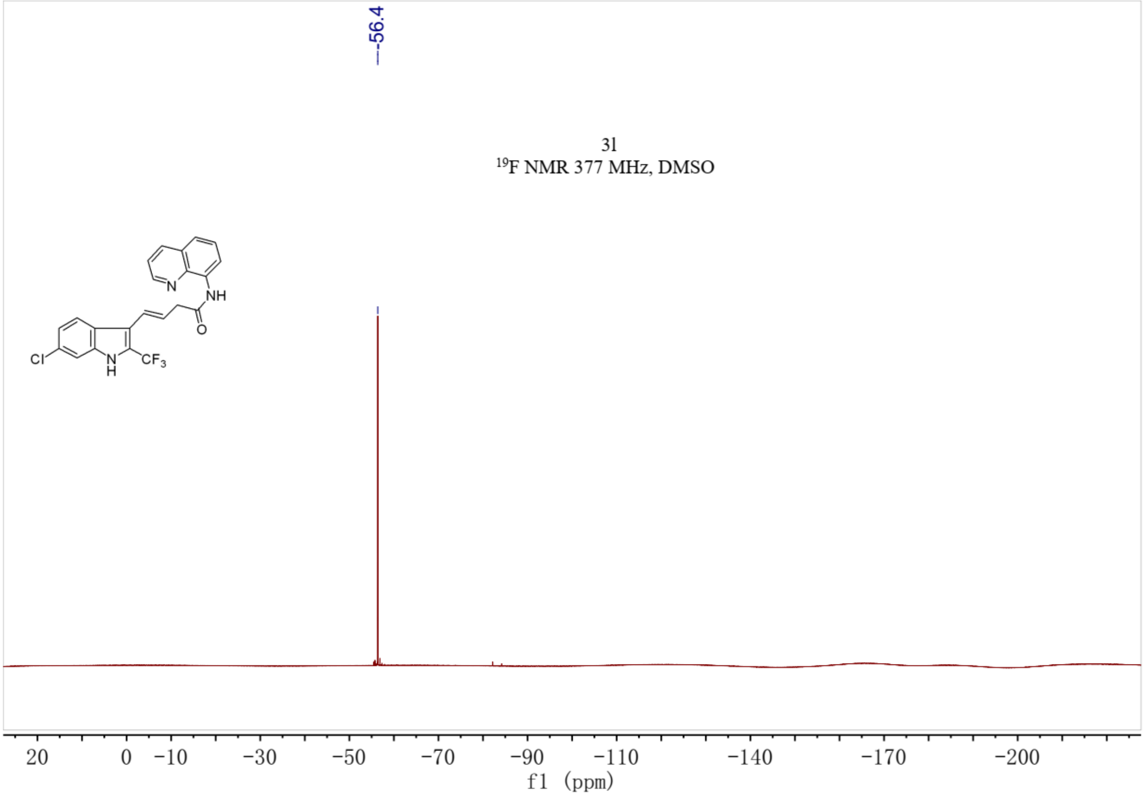

-56.4
31
19F NMR 377 MHz, DMSO
NH
N
O
Cl
N
H
CF3
20
0
−10
−30
−50
−70
−90
−110
−140
−170
−200
f1 (ppm)

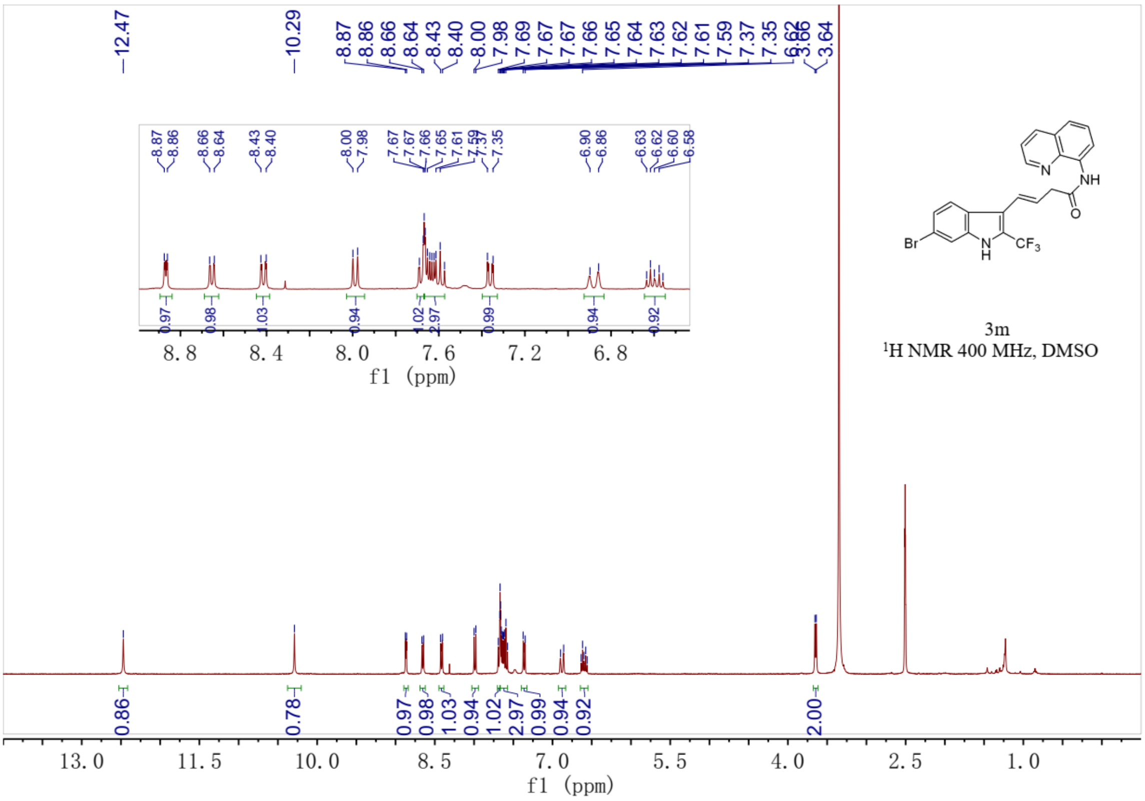
12.47
10.29
8.87 8.86 8.66 8.64 8.43 8.40 8.00 7.98 7.69 7.67 7.67 7.66 7.65 7.64 7.63 7.62 7.61 7.59 7.37 7.35 6.62 3.66 3.64
3m
¹H NMR 400 MHz, DMSO
0.86 0.78 0.97 0.98 1.03 0.94 1.02 2.97 0.99 0.94 0.92 2.00
f1 (ppm)

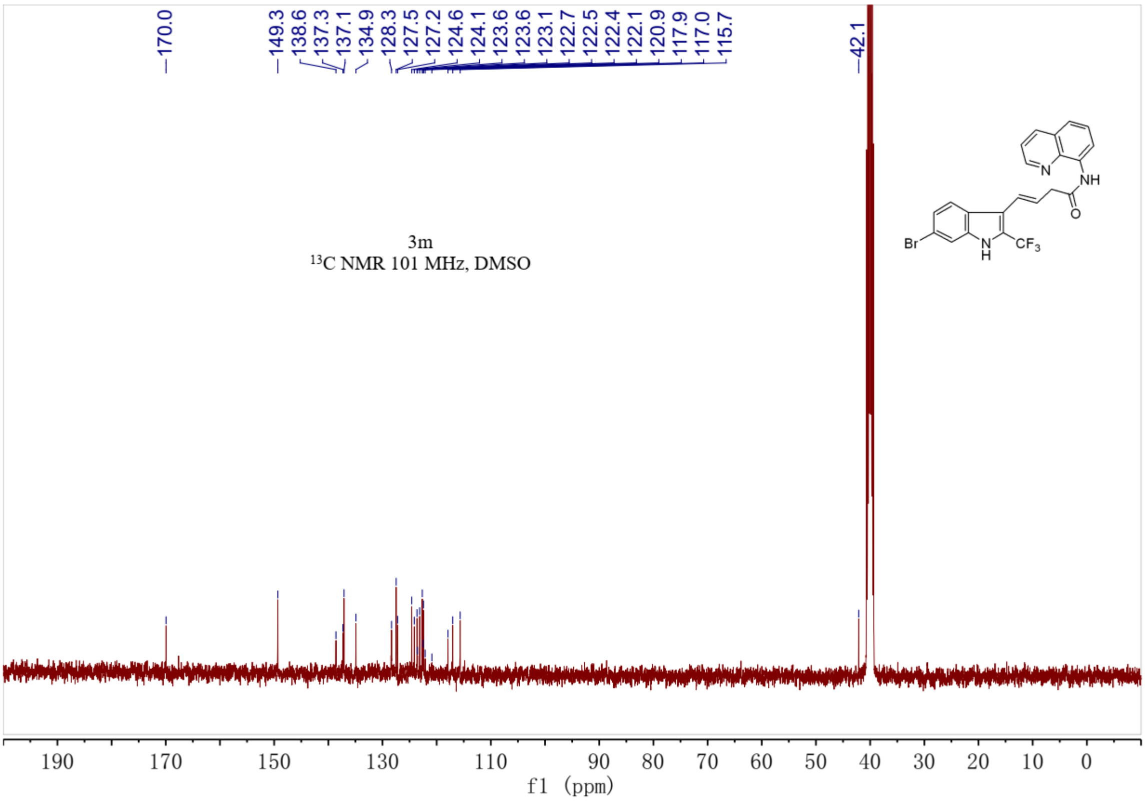
170.0
149.3 138.6 137.3 137.1 134.9 128.3 127.5 127.2 124.6 124.1 123.6 123.6 123.1 122.7 122.5 122.4 122.1 120.9 117.9 117.0 115.7
42.1
3m
¹³C NMR 101 MHz, DMSO
f1 (ppm)

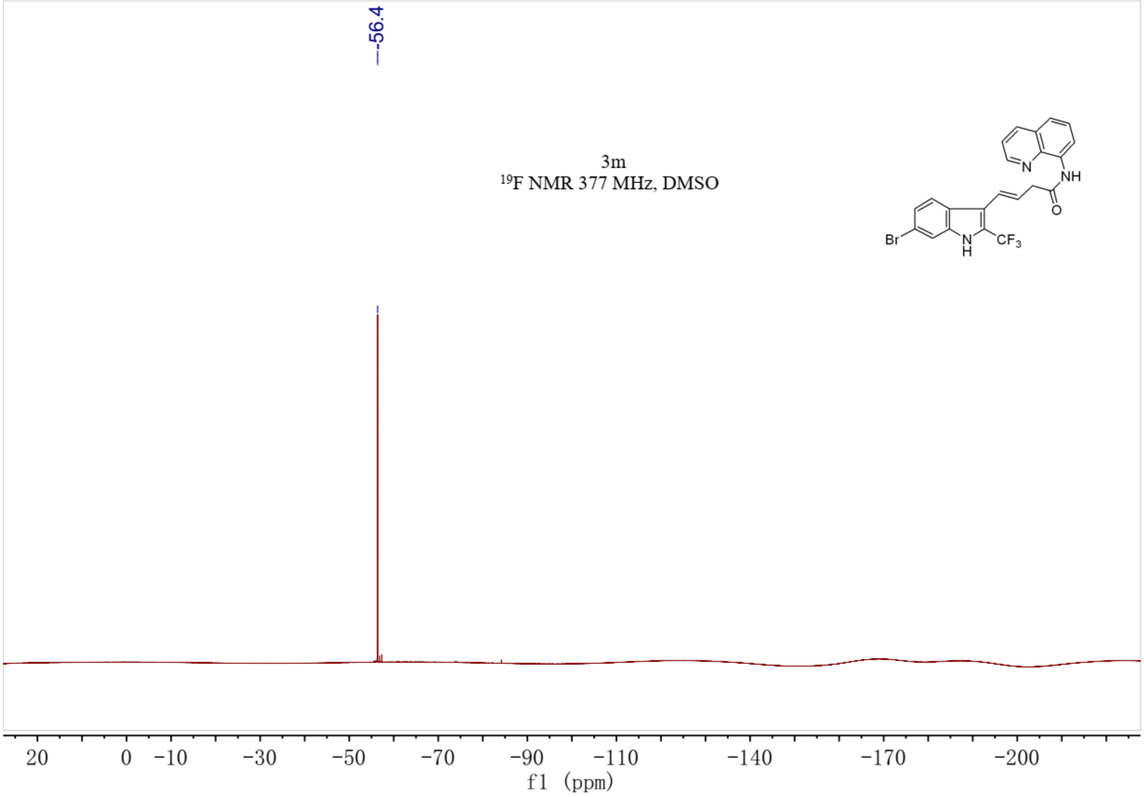
-56.4
3m
19F NMR 377 MHz, DMSO
NH
N
O
Br
N
H
CF3
20
0
−10
−30
−50
−70
−90
−110
−140
−170
−200
f1 (ppm)

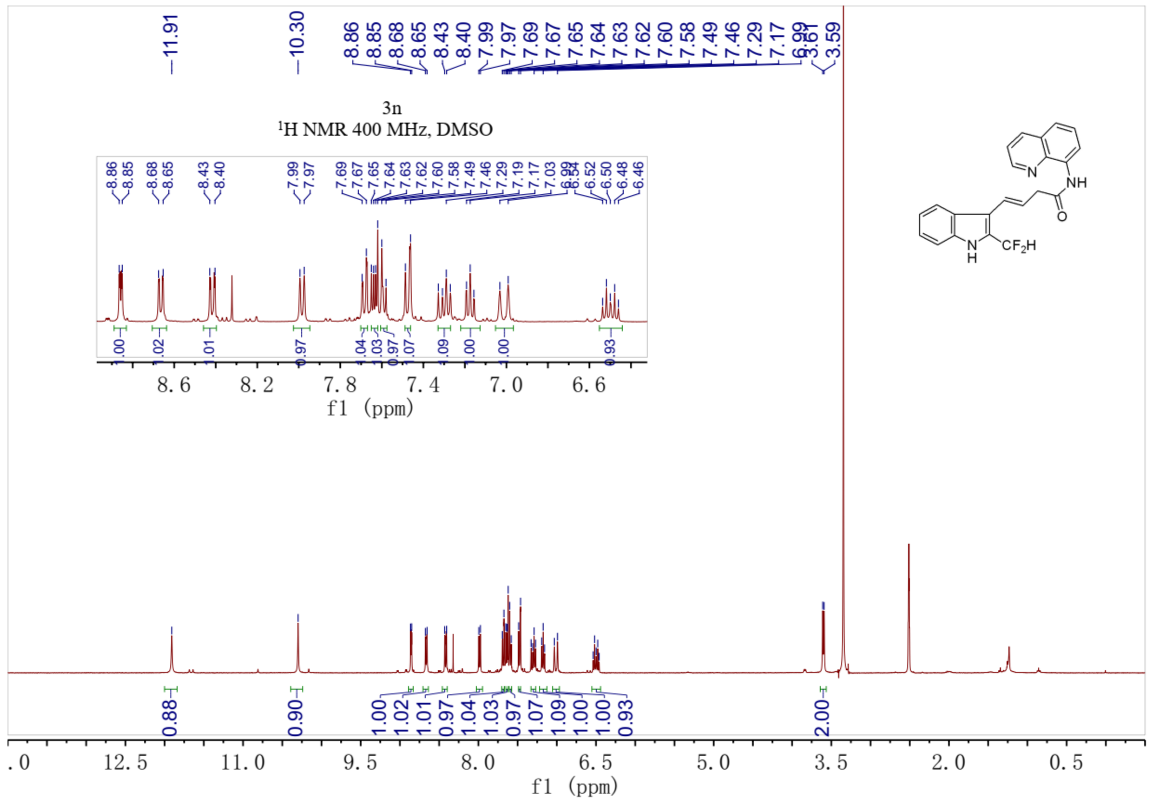
3n
1H NMR 400 MHz, DMSO
f1 (ppm)

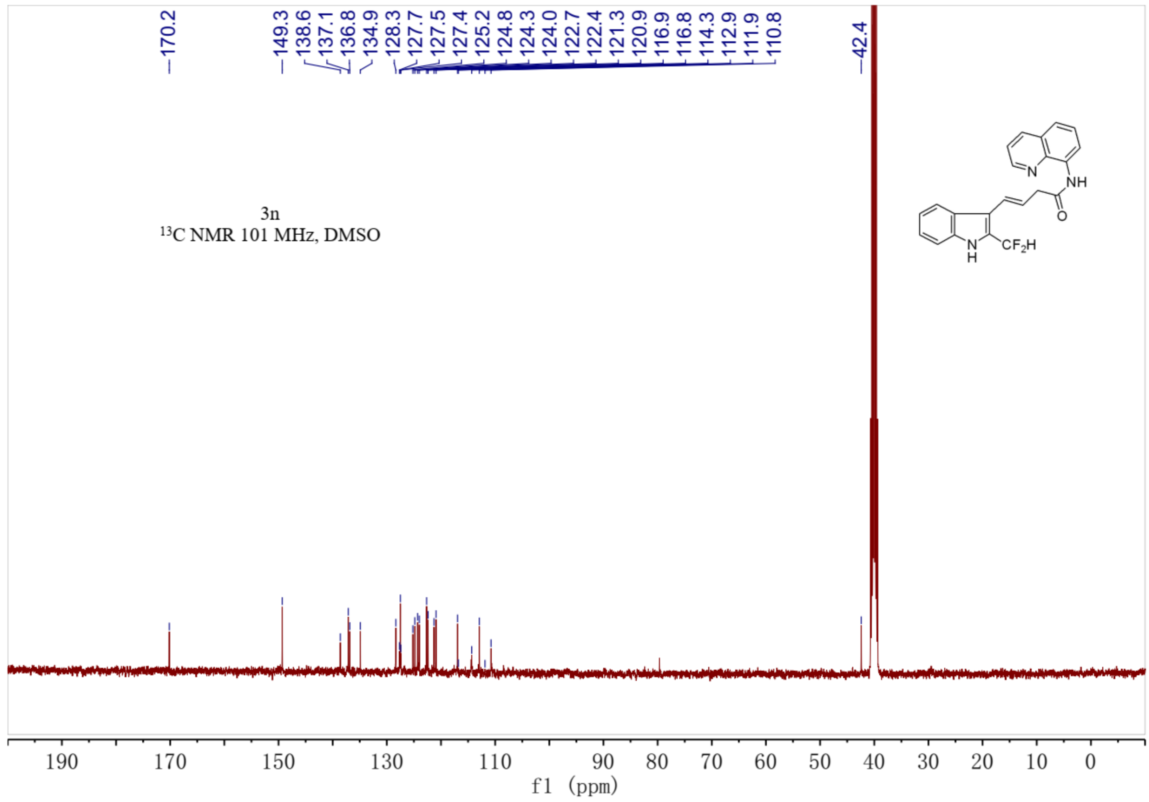
3n
13C NMR 101 MHz, DMSO
f1 (ppm)

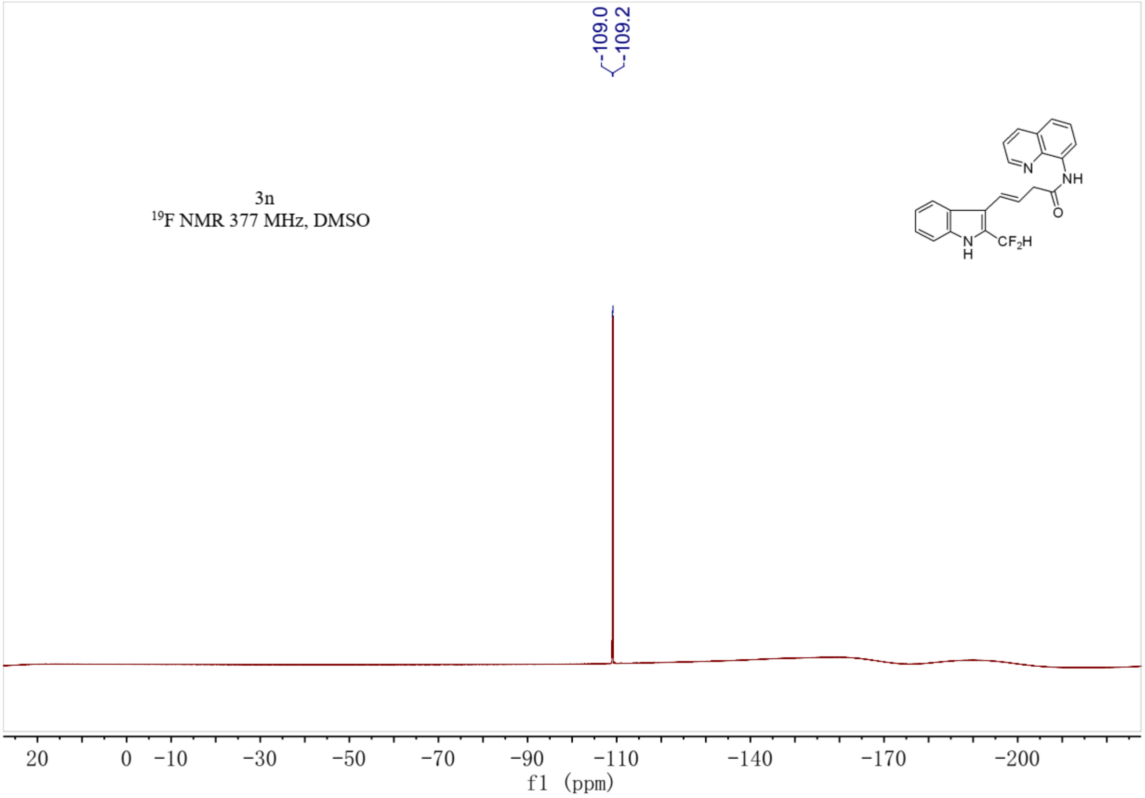
-109.0
-109.2
3n
19F NMR 377 MHz, DMSO
NH
N
O
N
H
CF2H
20
0
−10
−30
−50
−70
−90
−110
−140
−170
−200
f1 (ppm)

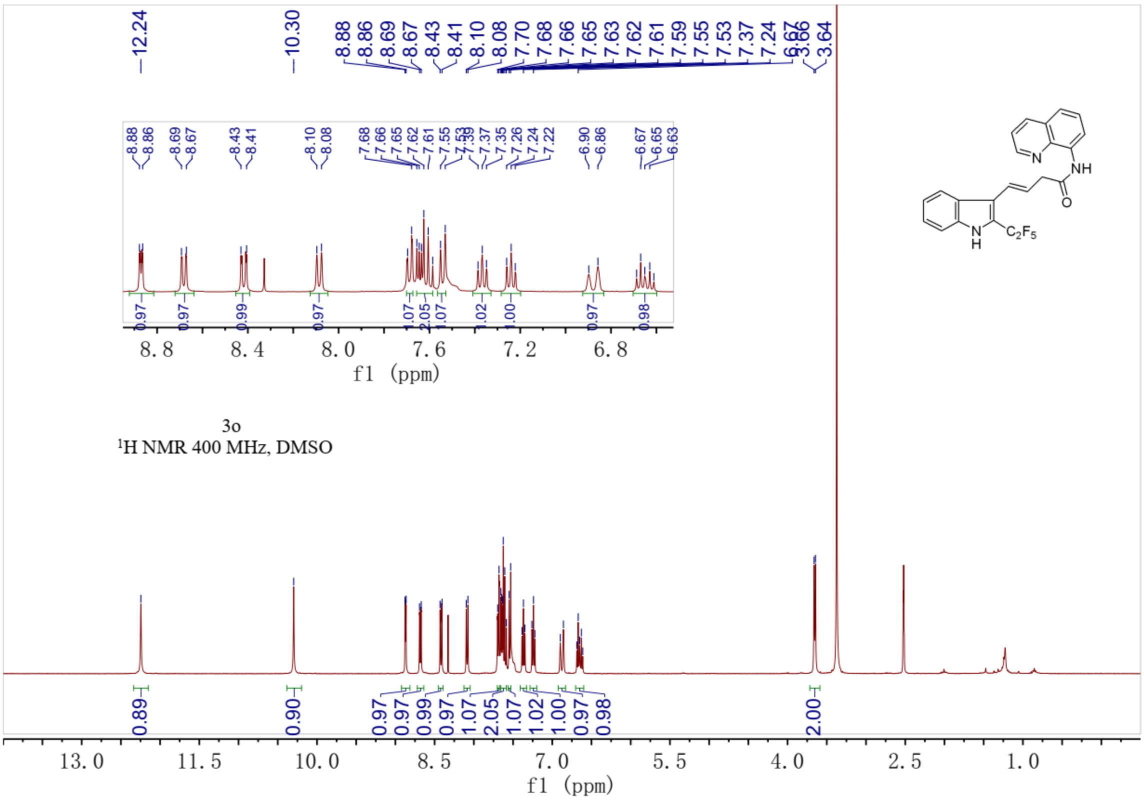
3o
1H NMR 400 MHz, DMSO
f1 (ppm)

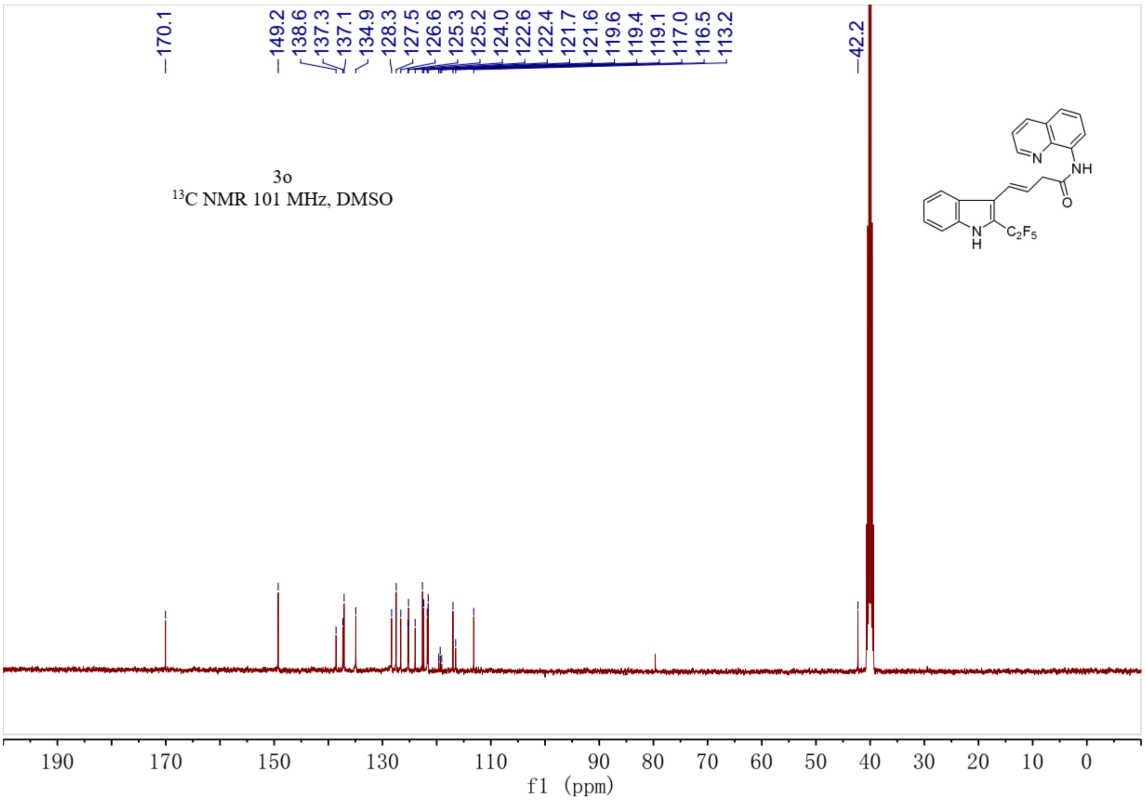
3o
13C NMR 101 MHz, DMSO
f1 (ppm)

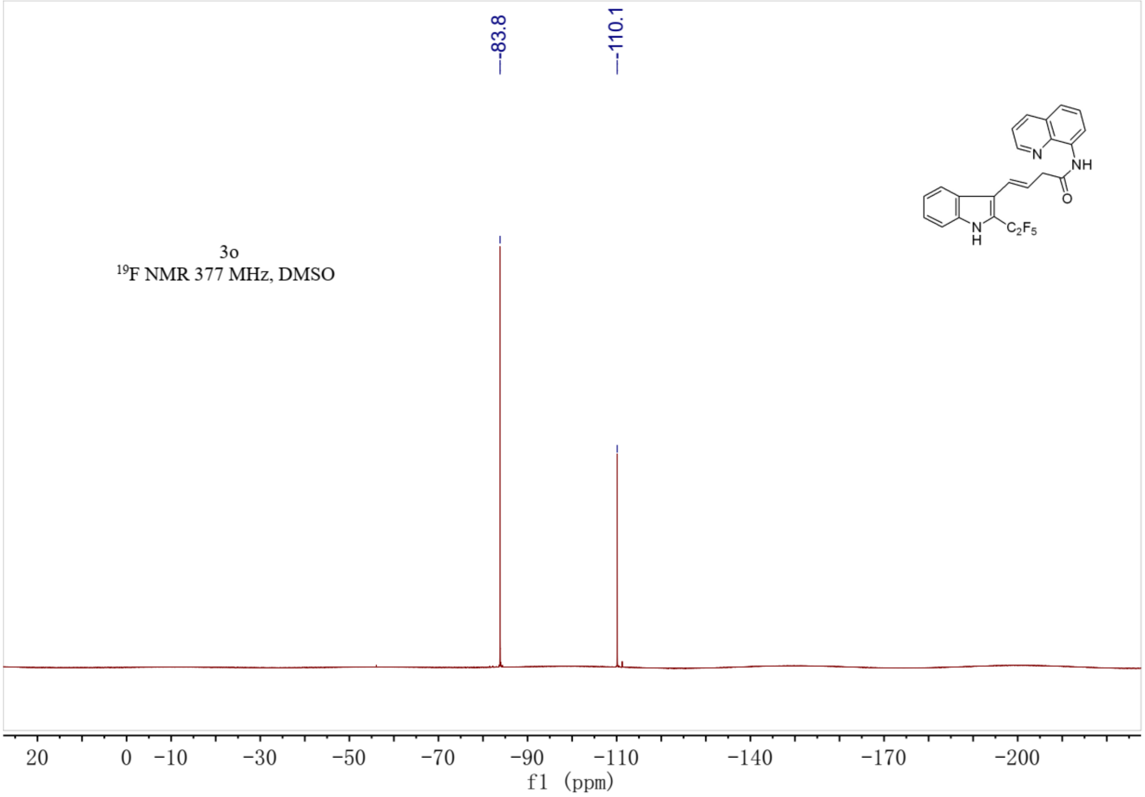
-83.8
-110.1
3o
19F NMR 377 MHz, DMSO
NH
N
O
N
H
C2F5
20
0
−10
−30
−50
−70
−90
−110
−140
−170
−200
f1 (ppm)

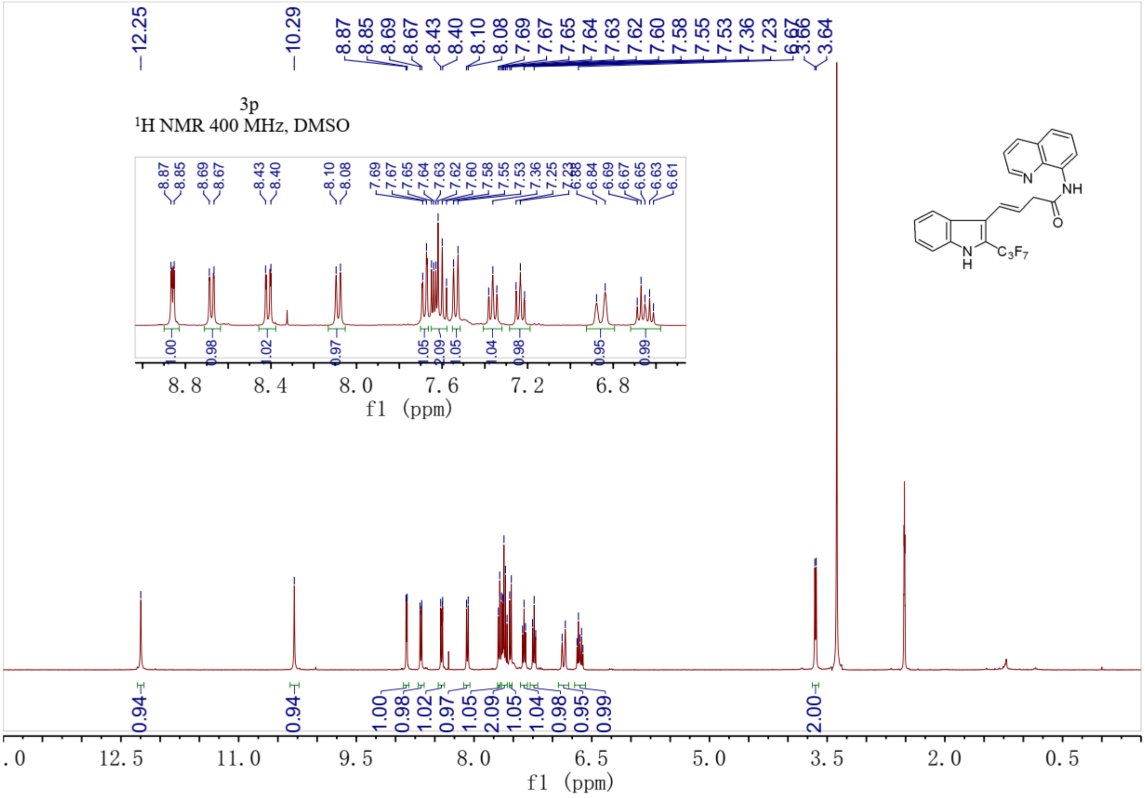
3p
^{1}H NMR 400 MHz, DMSO

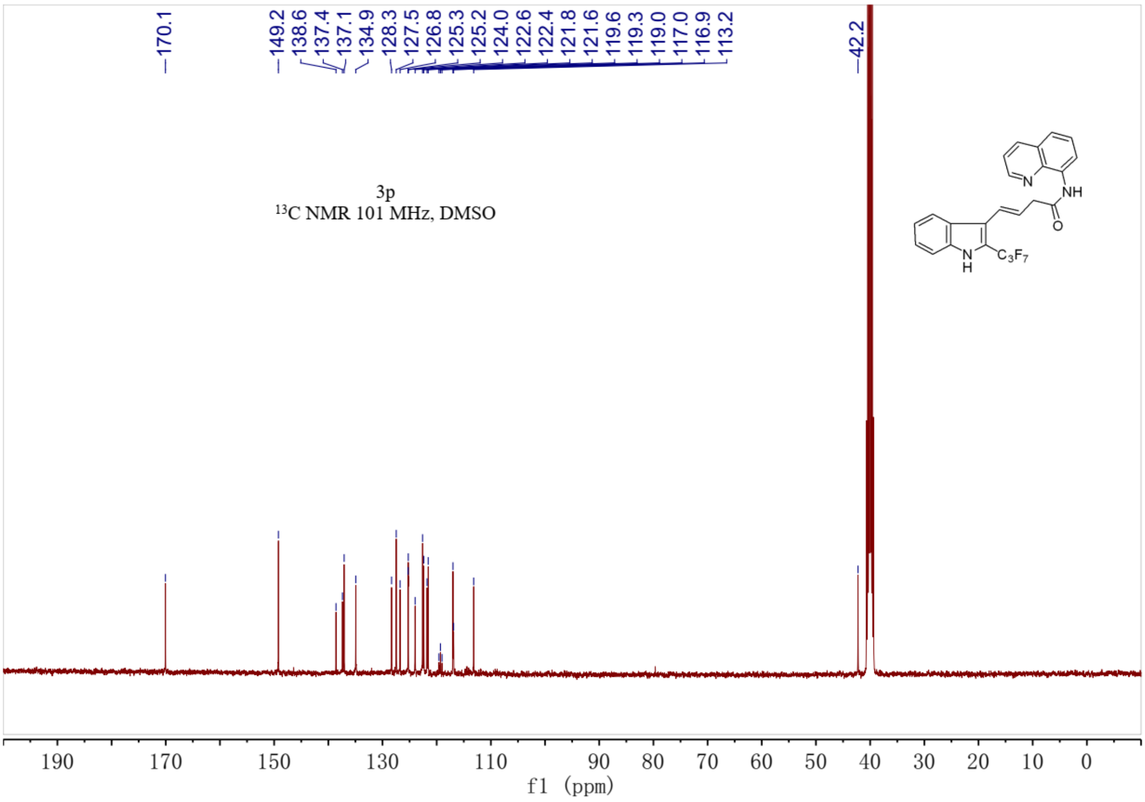
3p
^{13}C NMR 101 MHz, DMSO

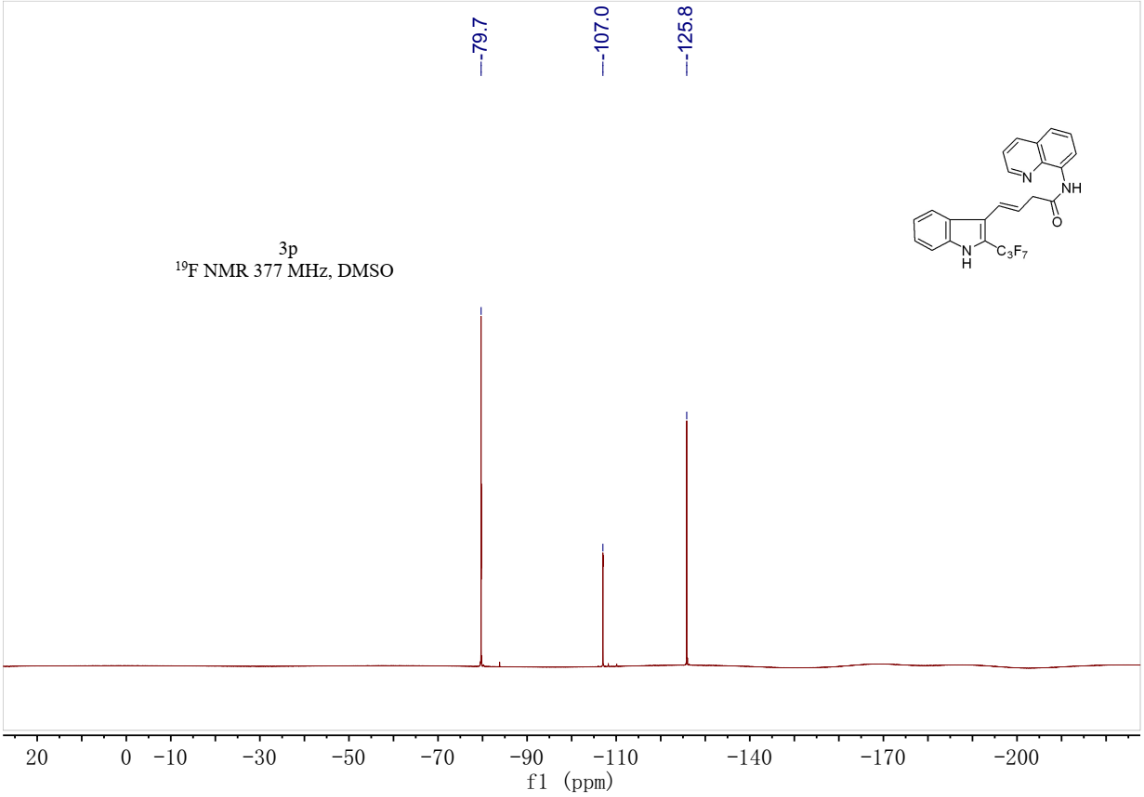

-79.7
-107.0
-125.8
3p
19F NMR 377 MHz, DMSO
NH
N
O
N
H
C3F7
20
0
-10
-30
-50
-70
-90
-110
-140
-170
-200
f1 (ppm)

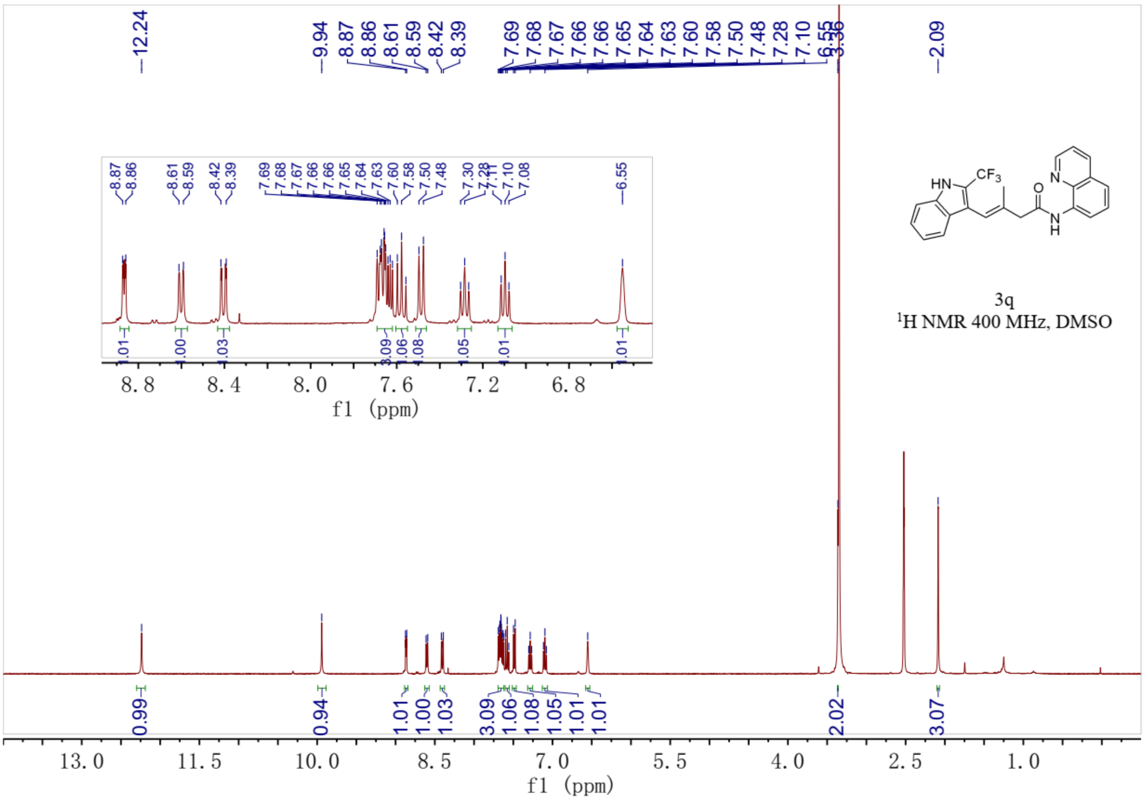
12.24
9.94
8.87
8.86
8.61
8.59
8.42
8.39
7.69
7.68
7.67
7.66
7.66
7.65
7.64
7.63
7.60
7.58
7.50
7.48
7.28
7.10
6.55
2.09
3q
¹H NMR 400 MHz, DMSO
f1 (ppm)

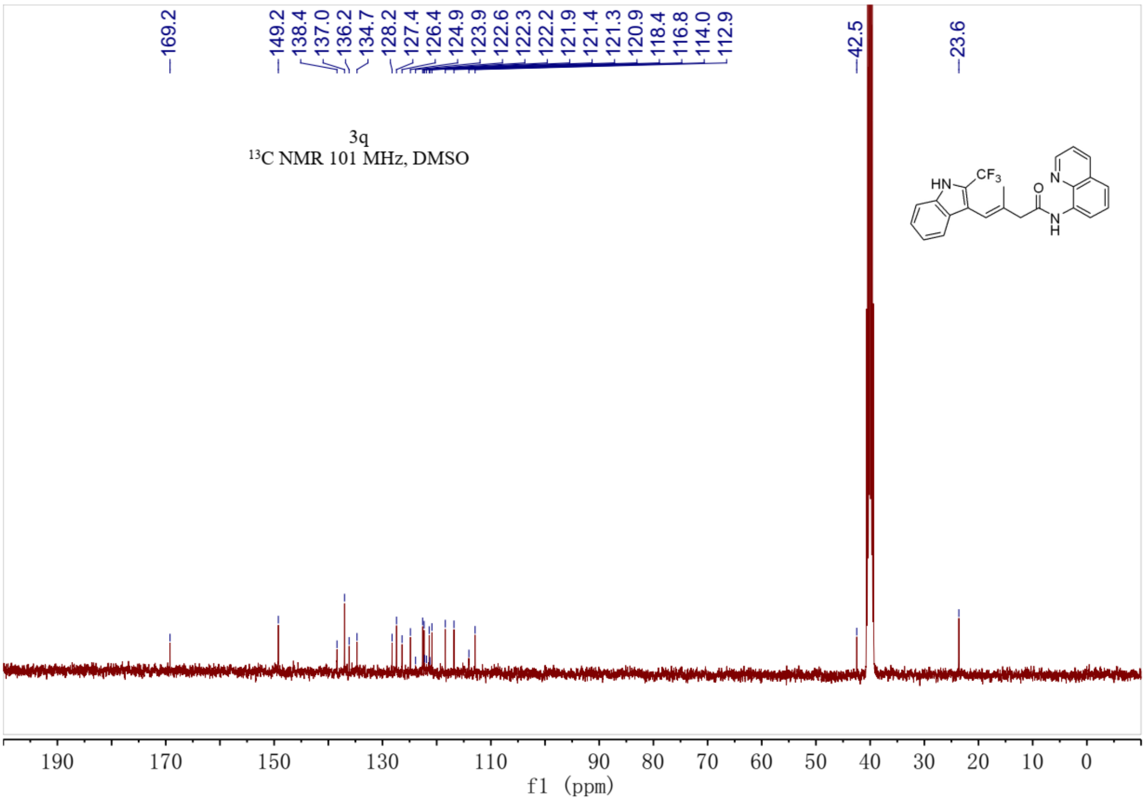
169.2
149.2
138.4
137.0
136.2
134.7
128.2
127.4
126.4
124.9
123.9
122.6
122.3
122.2
121.9
121.4
121.3
120.9
118.4
116.8
114.0
112.9
42.5
23.6
3q
¹³C NMR 101 MHz, DMSO
f1 (ppm)

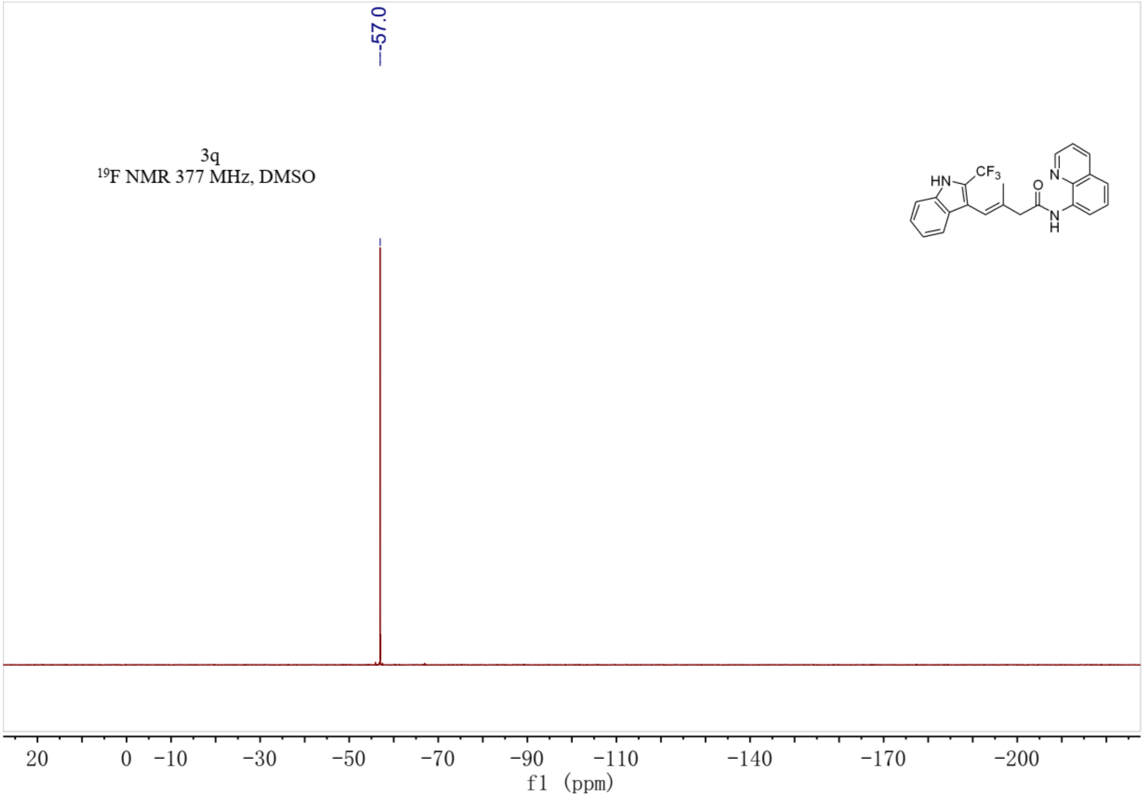
-57.0
3q
19F NMR 377 MHz, DMSO
HN
CF3
O
N
N
H
20
0
−10
−30
−50
−70
−90
−110
−140
−170
−200
f1 (ppm)

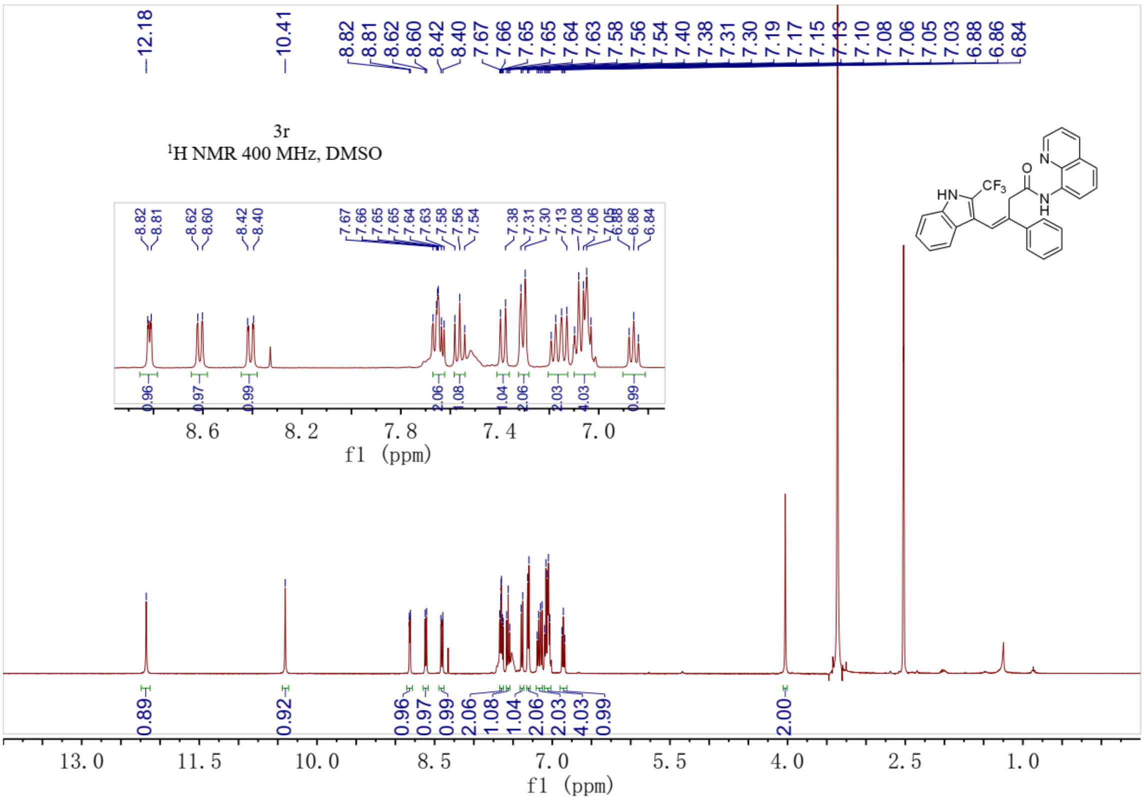
3r
¹H NMR 400 MHz, DMSO
f1 (ppm)

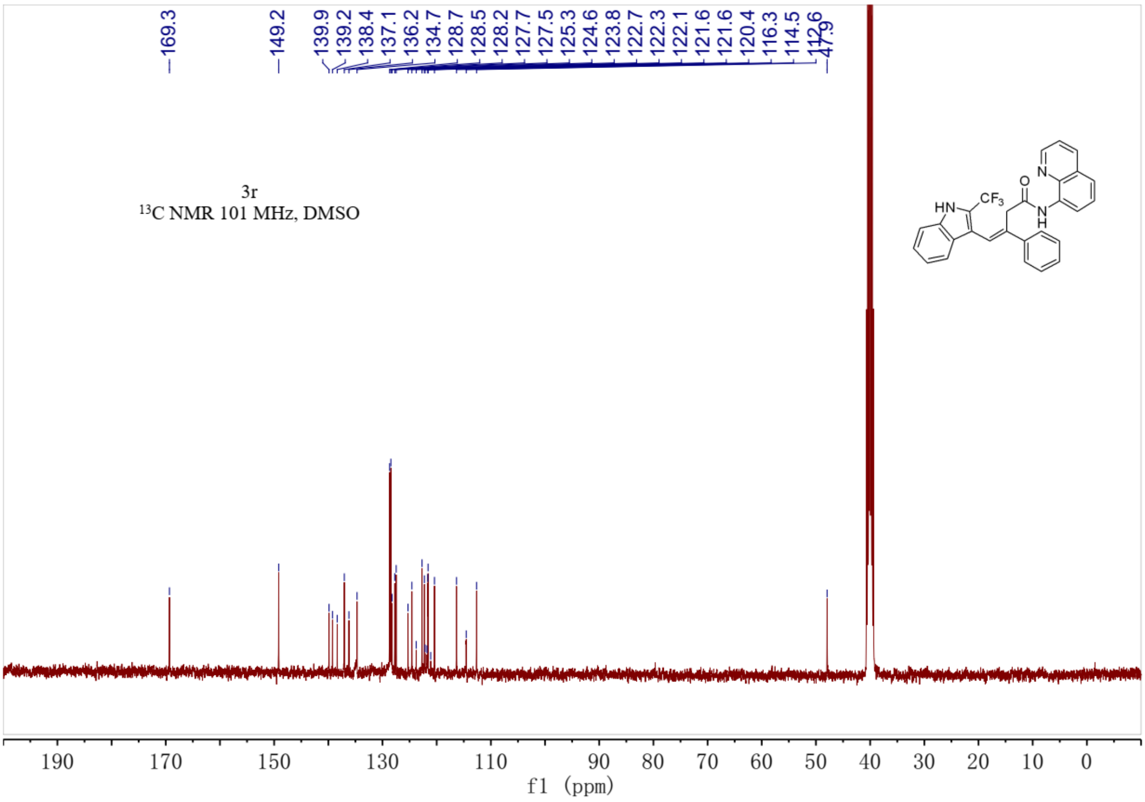
3r
¹³C NMR 101 MHz, DMSO
f1 (ppm)

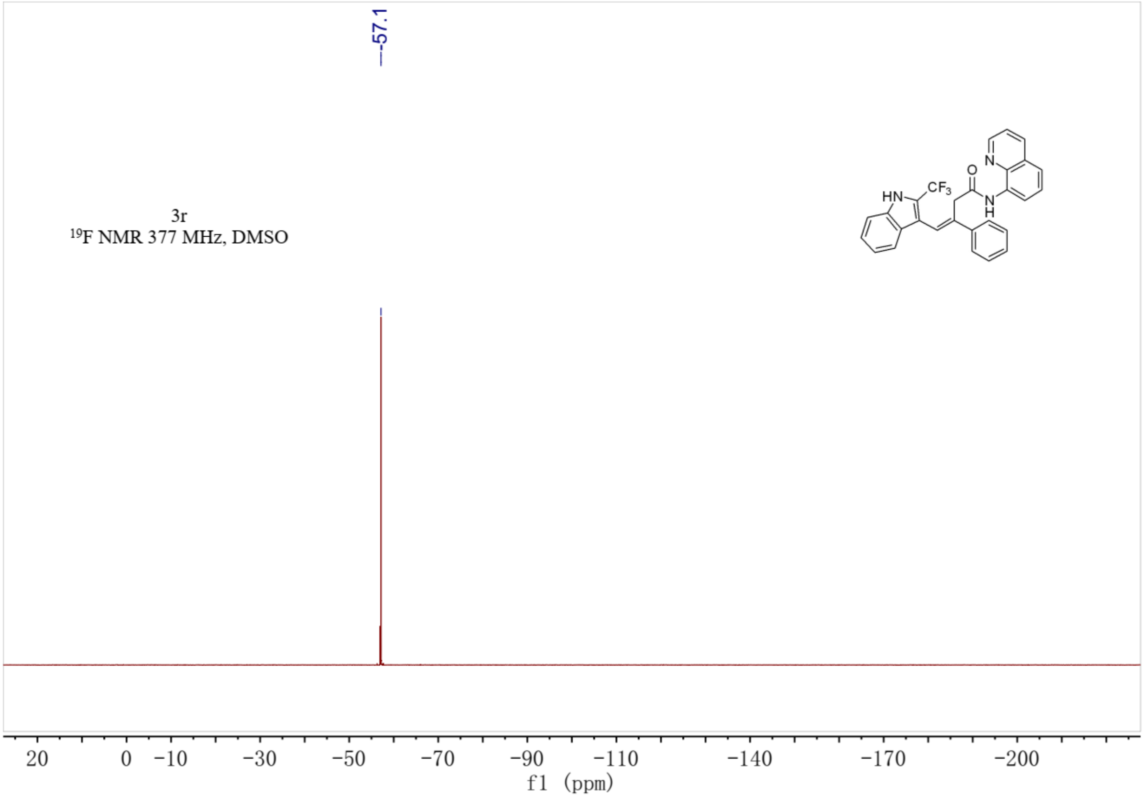
-57.1
3r
19F NMR 377 MHz, DMSO
HN
CF3
O
N
H
20
0
−10
−30
−50
−70
−90
−110
−140
−170
−200
f1 (ppm)

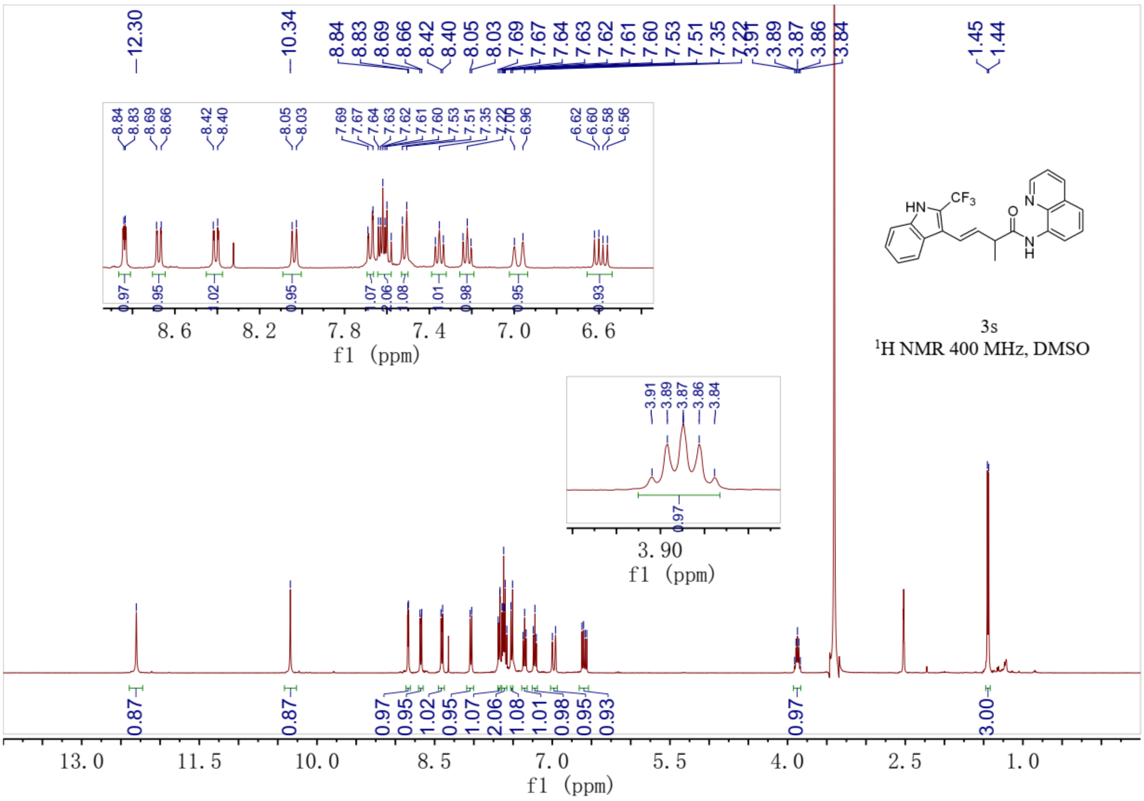
3s
¹H NMR 400 MHz, DMSO
f1 (ppm)

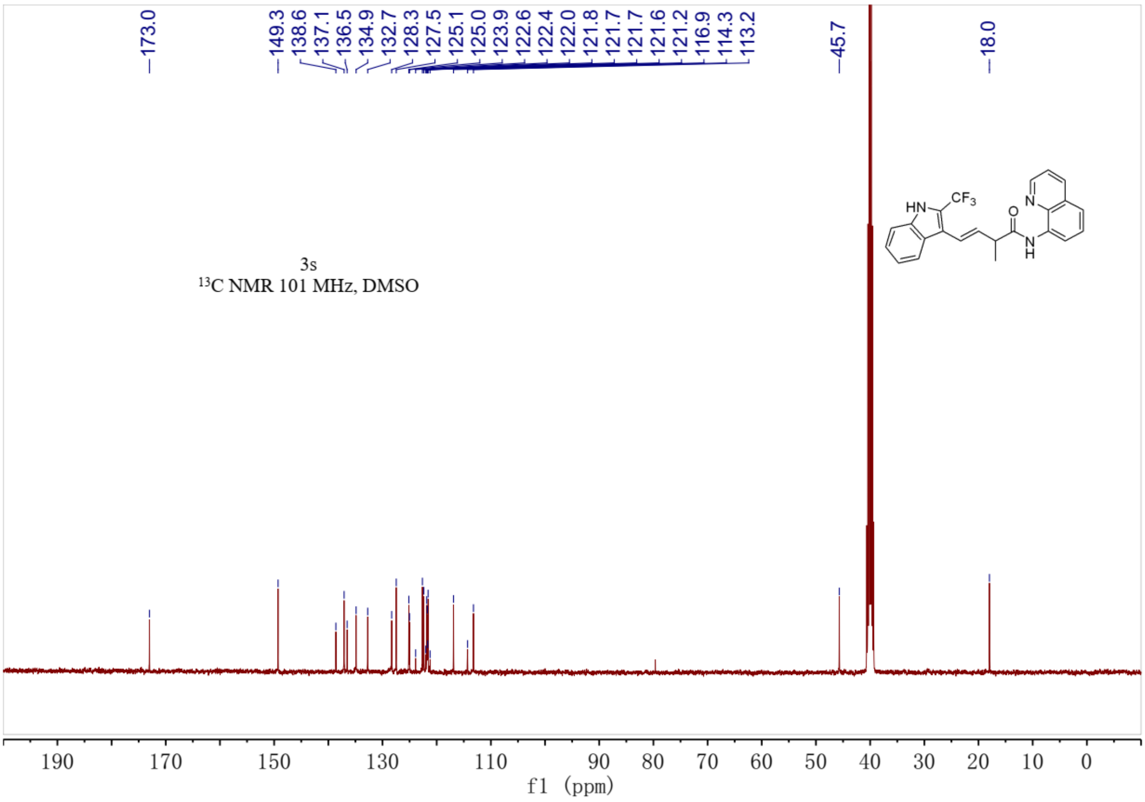
3s
¹³C NMR 101 MHz, DMSO
f1 (ppm)

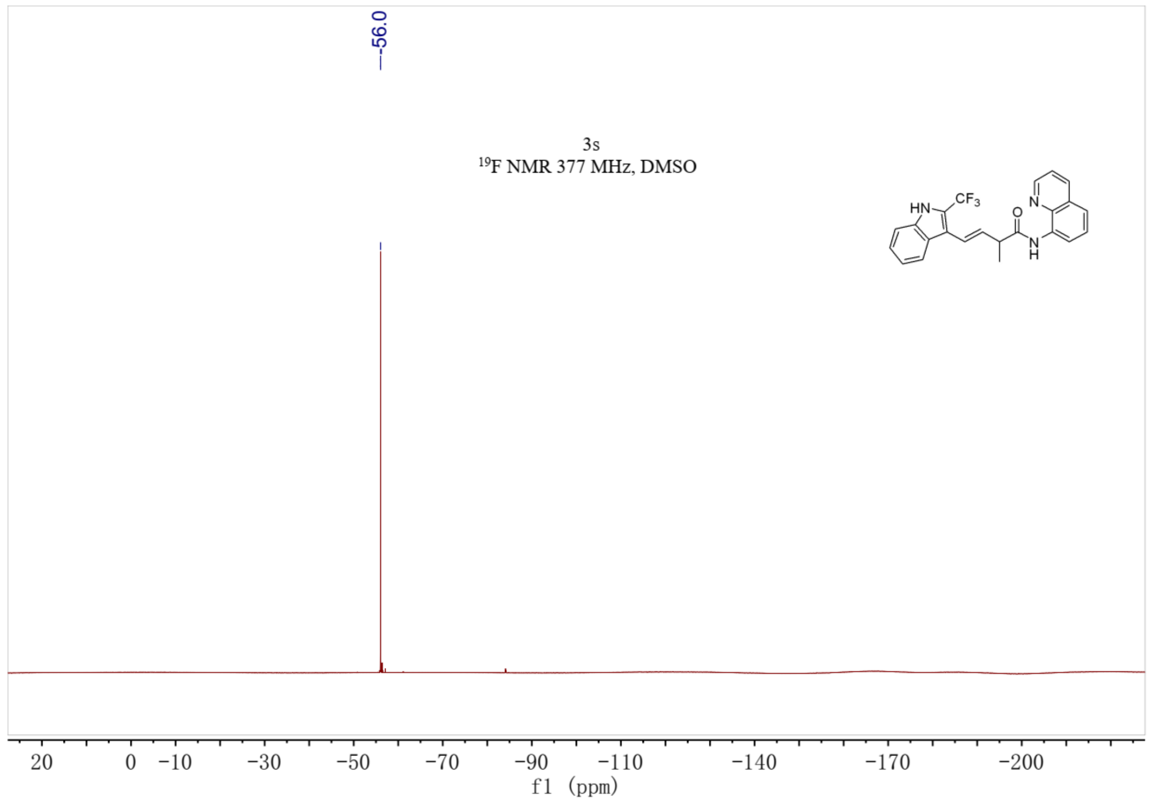
-56.0
3s
19F NMR 377 MHz, DMSO
HN
CF3
O
N
N
H
20
0
-10
-30
-50
-70
-90
-110
-140
-170
-200
f1 (ppm)

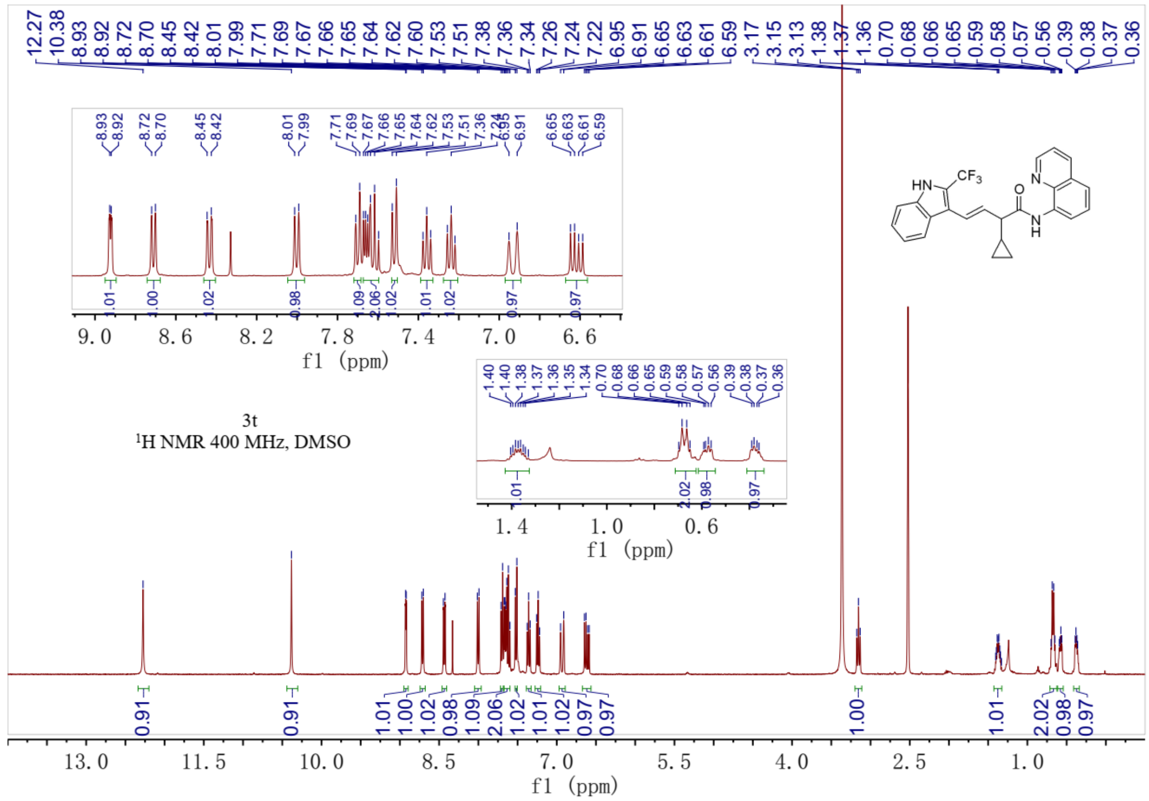
3t
1H NMR 400 MHz, DMSO
f1 (ppm)

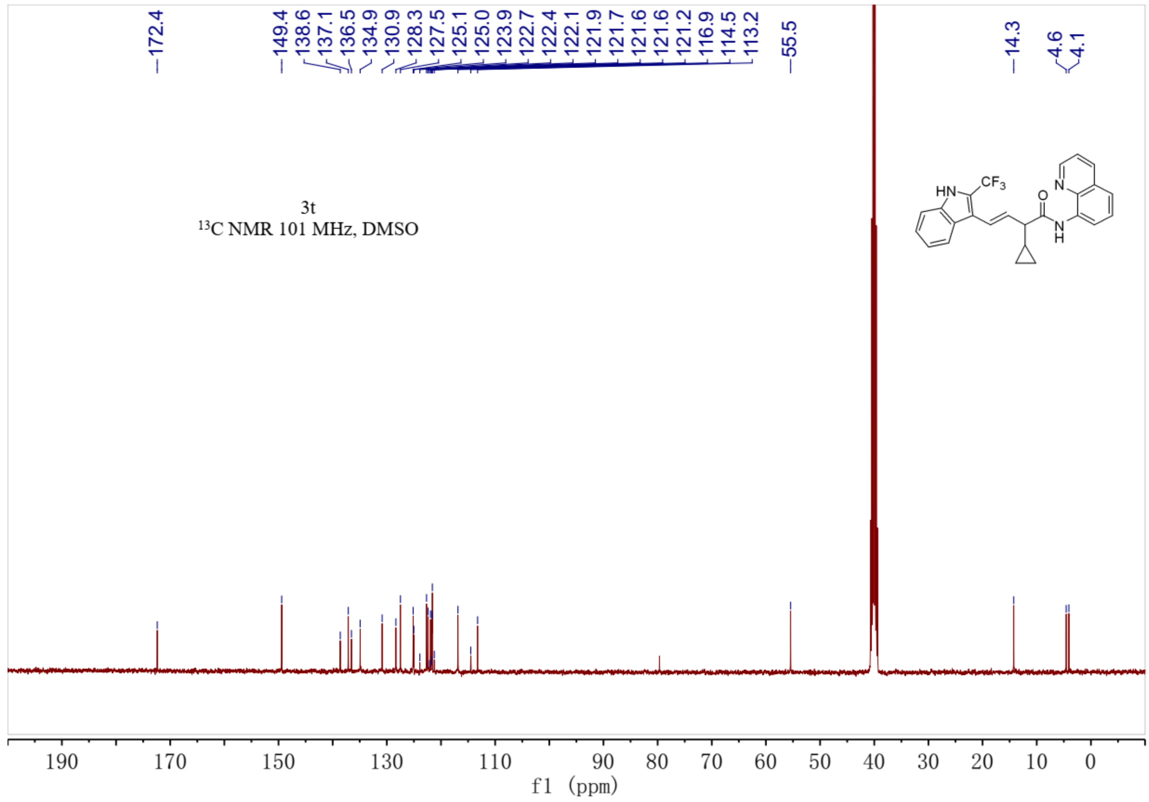
3t
13C NMR 101 MHz, DMSO
f1 (ppm)

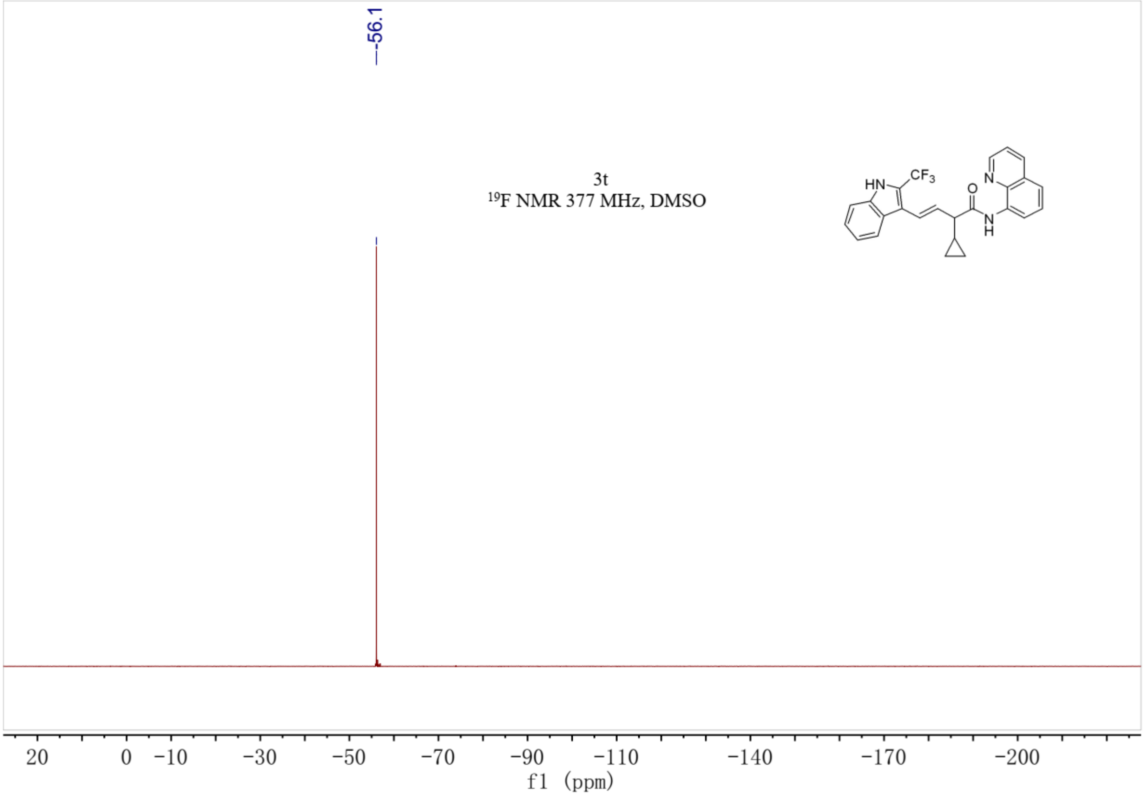
-56.1
3t
19F NMR 377 MHz, DMSO
HN
CF3
O
N
N
H
20
0
−10
−30
−50
−70
−90
−110
−140
−170
−200
f1 (ppm)

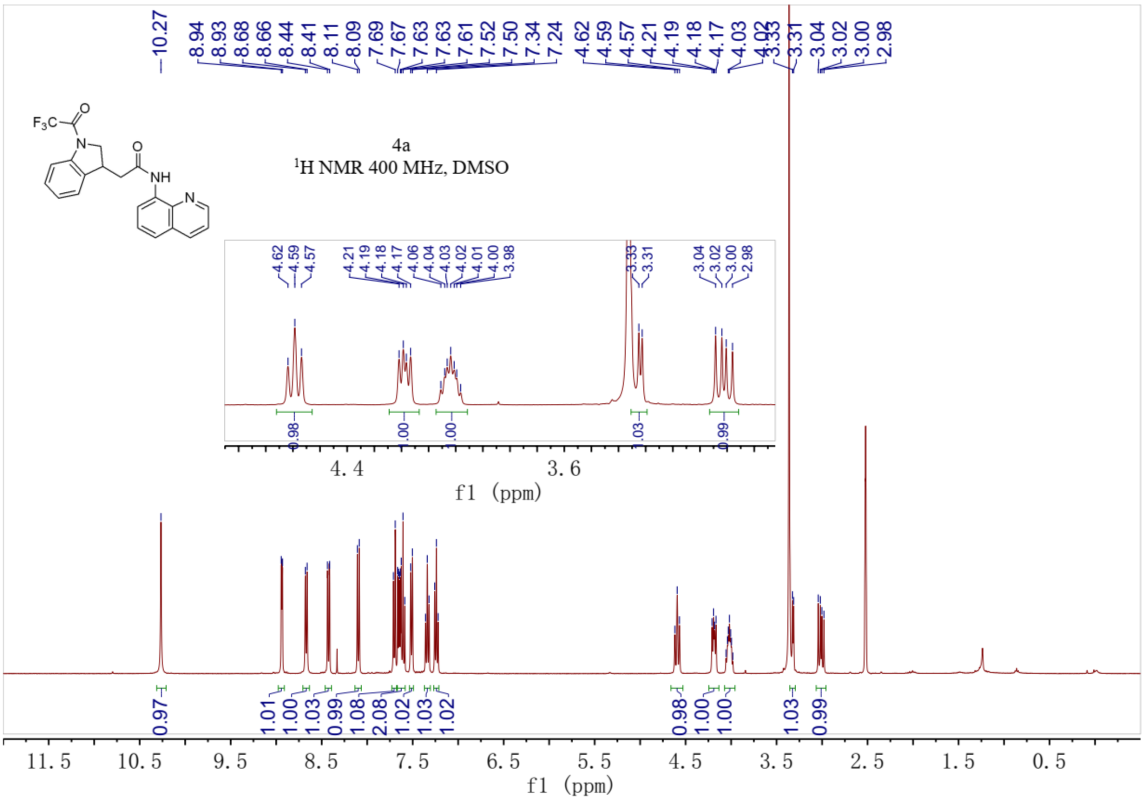

4a
^{1}H NMR 400 MHz, DMSO
f1 (ppm)

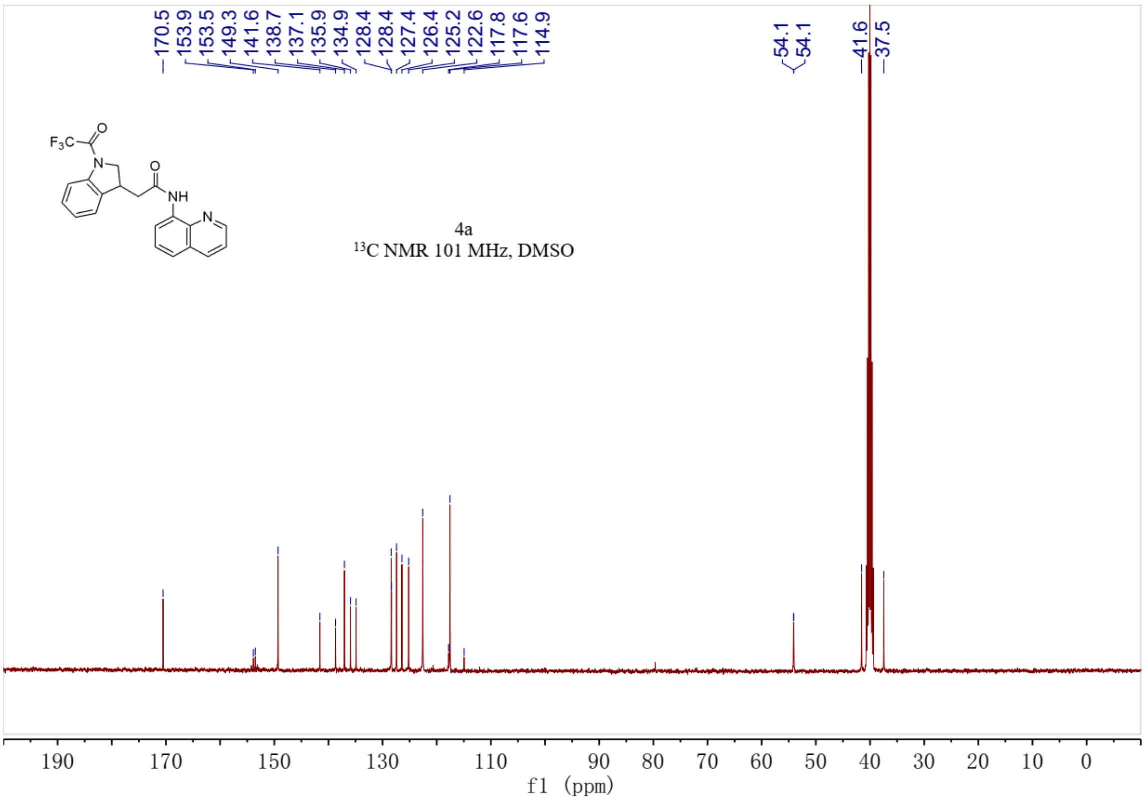

4a
^{13}C NMR 101 MHz, DMSO
f1 (ppm)

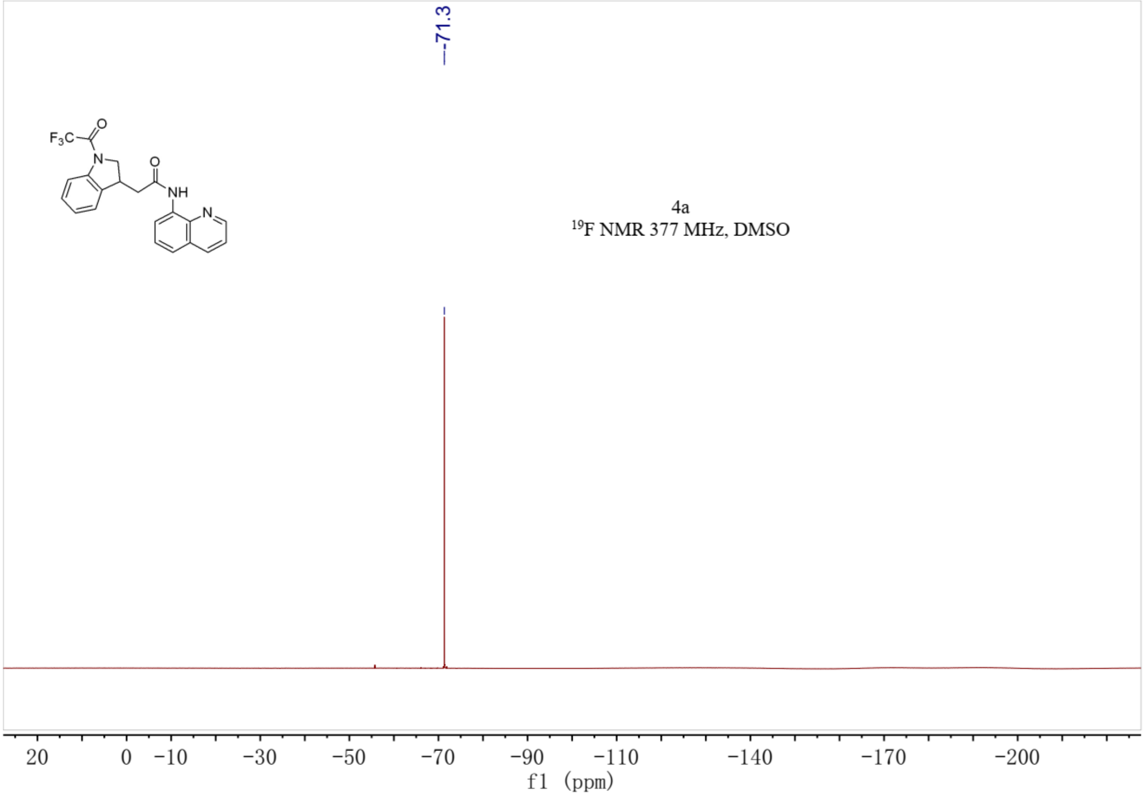

-71.3
F_3C
O
N
O
NH
N
4a
^{19}F NMR 377 MHz, DMSO
20
0
−10
−30
−50
−70
−90
−110
−140
−170
−200
f1 (ppm)

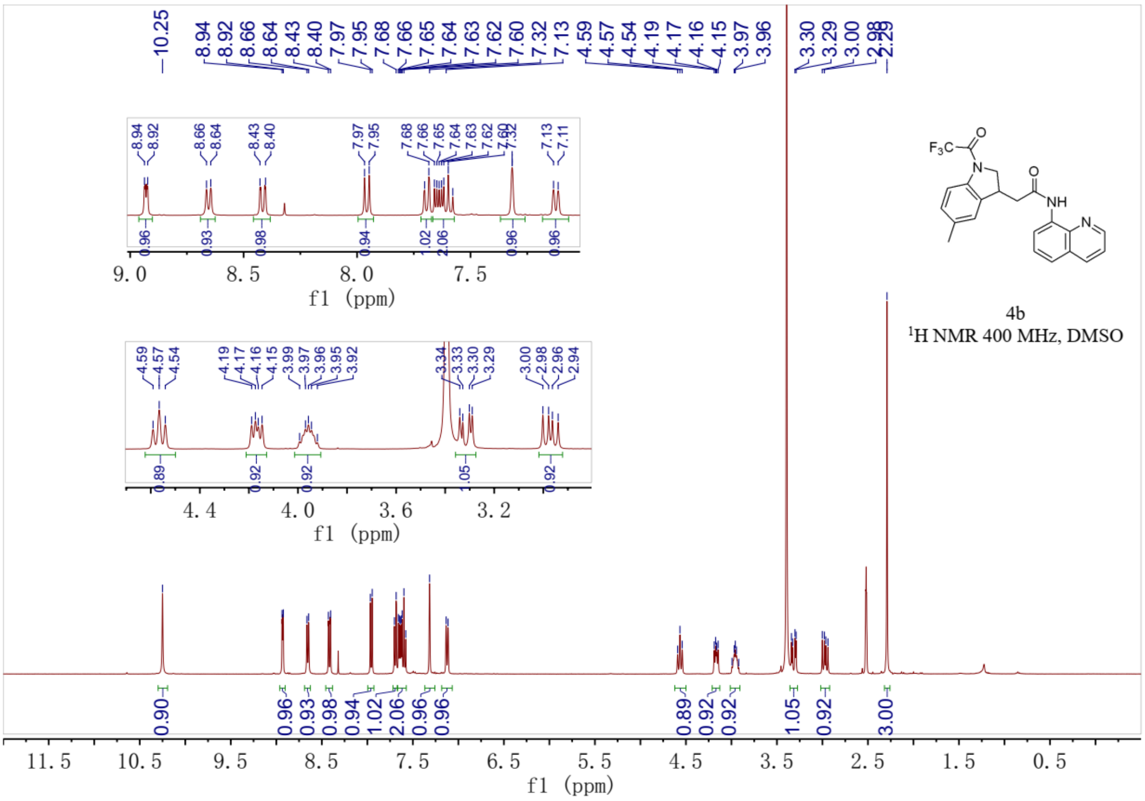
4b
¹H NMR 400 MHz, DMSO
f1 (ppm)

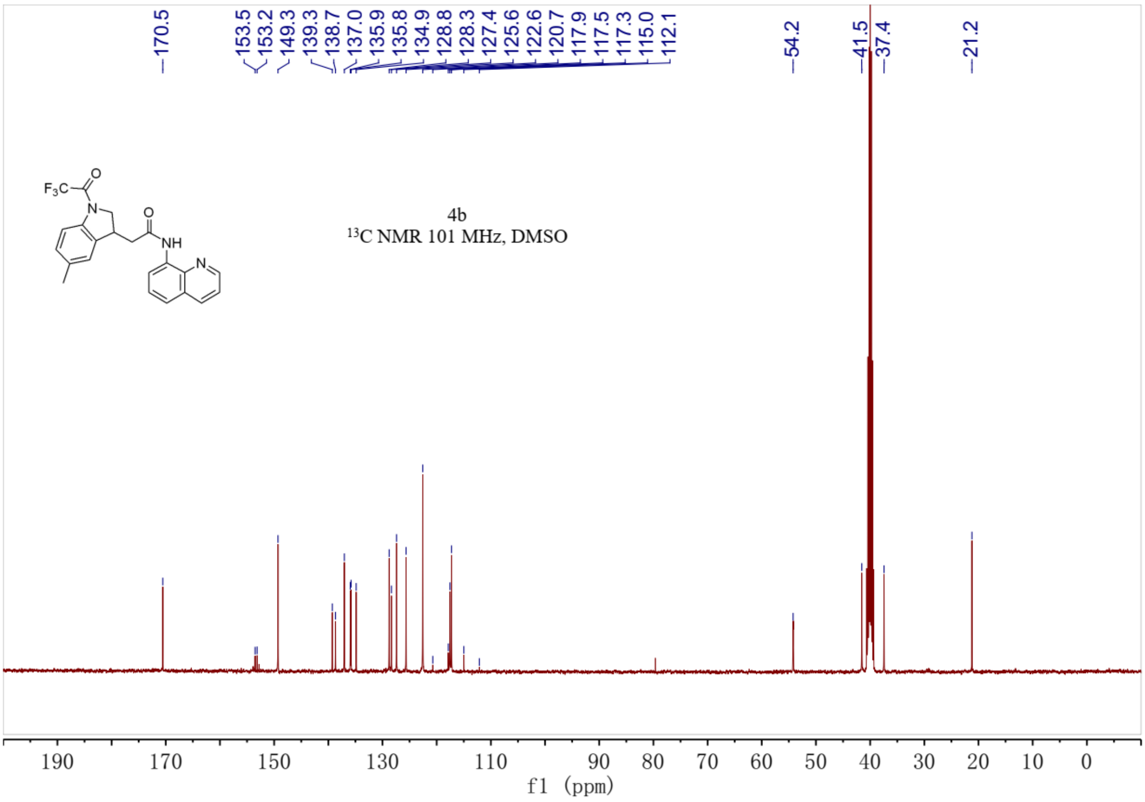
4b
¹³C NMR 101 MHz, DMSO
f1 (ppm)

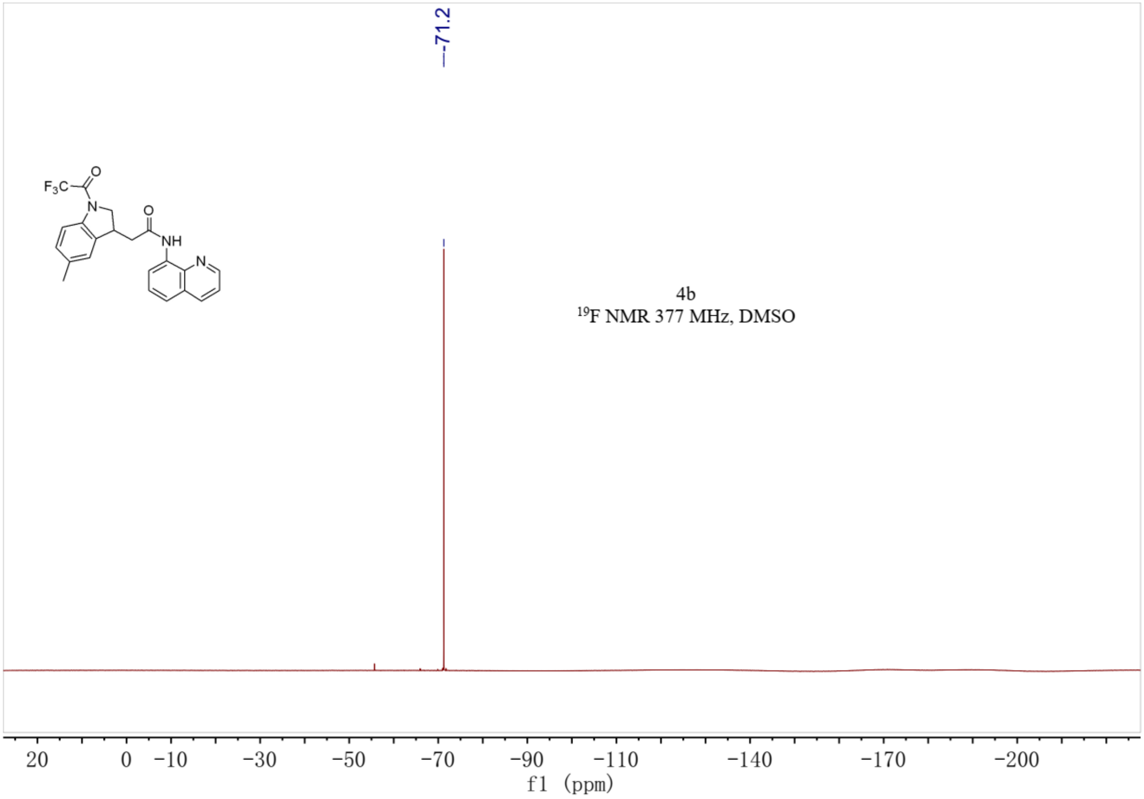
-71.2
4b
19F NMR 377 MHz, DMSO
20
0
-10
-30
-50
-70
-90
-110
-140
-170
-200
f1 (ppm)

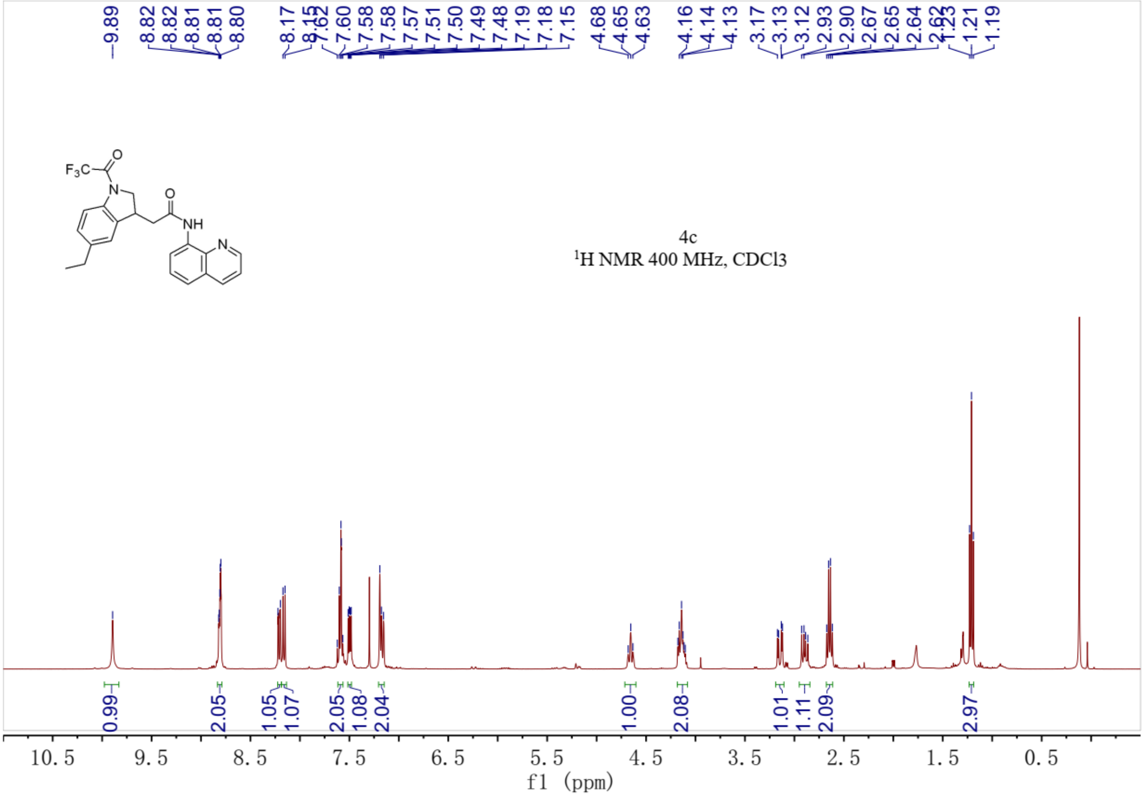
9.89
8.82
8.82
8.81
8.81
8.80
8.17
8.15
7.62
7.60
7.58
7.58
7.57
7.51
7.50
7.49
7.48
7.19
7.18
7.15
4.68
4.65
4.63
4.16
4.14
4.13
3.17
3.13
3.12
2.93
2.90
2.67
2.65
2.64
2.62
1.23
1.21
1.19
F3C
O
N
O
NH
N
4c
1H NMR 400 MHz, CDCl3
0.99
2.05
1.05
1.07
2.05
1.08
2.04
1.00
2.08
1.01
1.11
2.09
2.97
10.5
9.5
8.5
7.5
6.5
5.5
4.5
3.5
2.5
1.5
0.5
f1 (ppm)

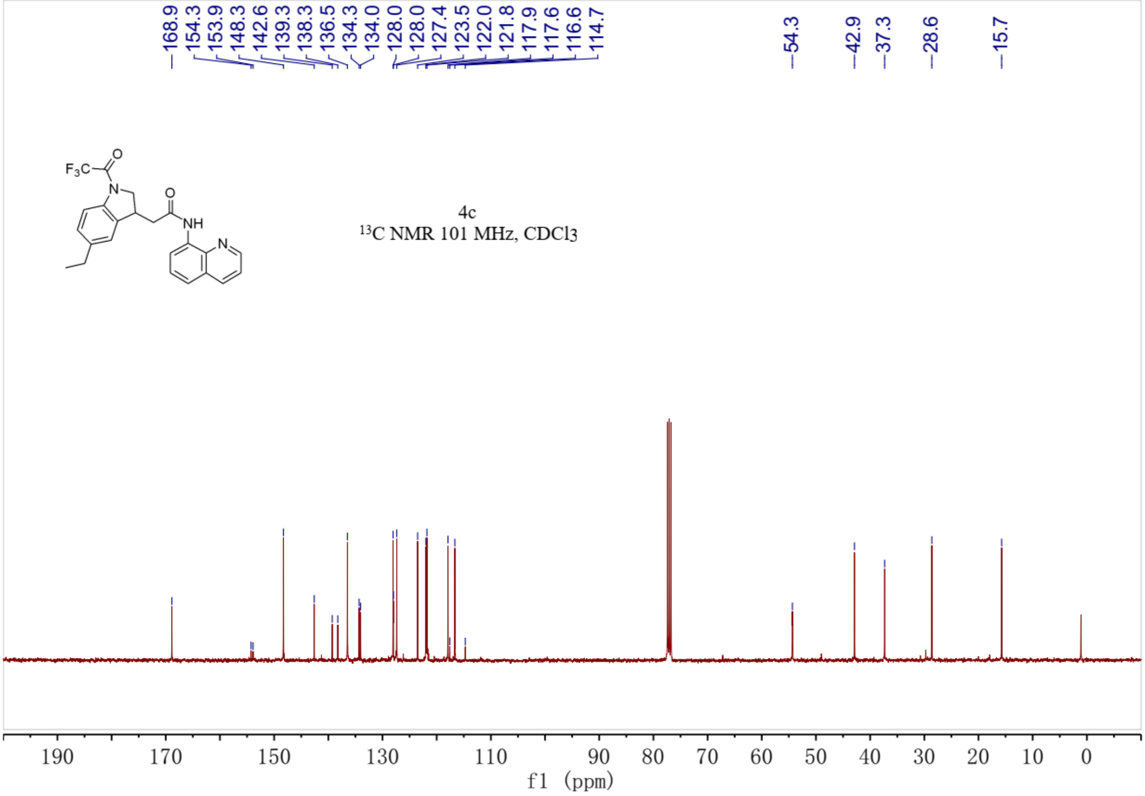
168.9
154.3
153.9
148.3
142.6
139.3
138.3
136.5
134.3
134.0
128.0
128.0
127.4
123.5
122.0
121.8
117.9
117.6
116.6
114.7
54.3
42.9
37.3
28.6
15.7
F3C
O
N
O
NH
N
4c
13C NMR 101 MHz, CDCl3
190
170
150
130
110
90
80
70
60
50
40
30
20
10
0
f1 (ppm)

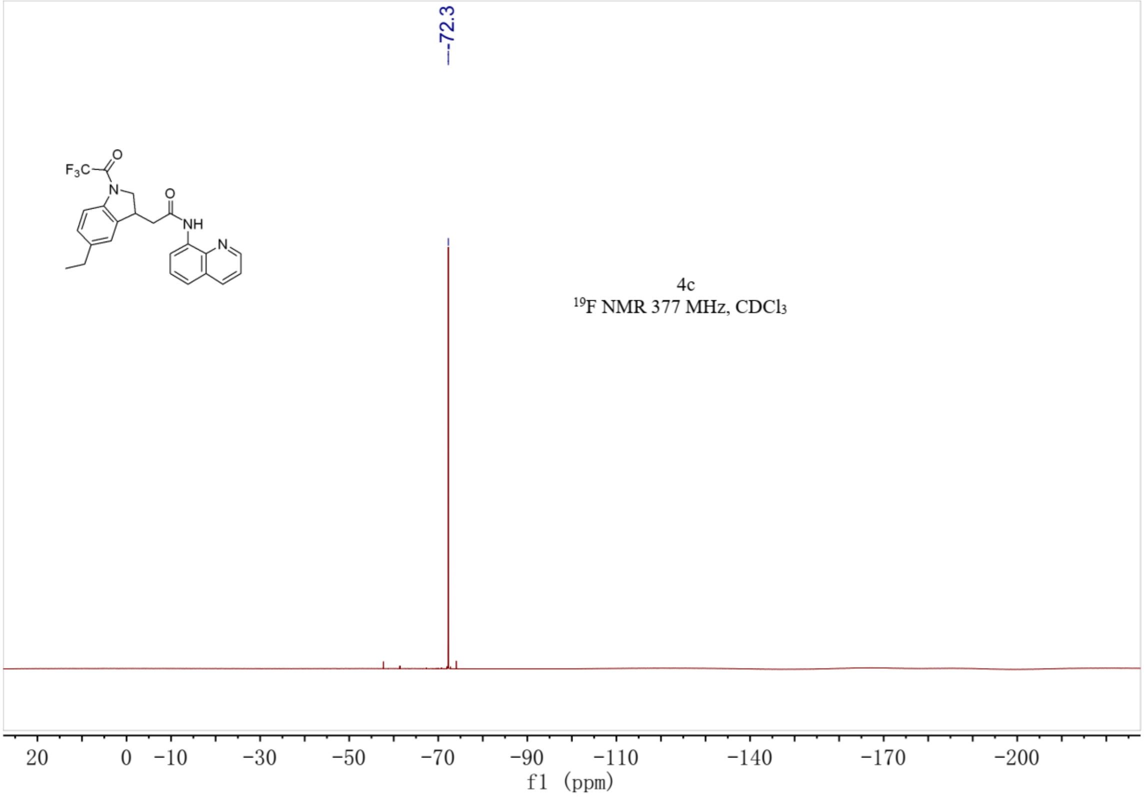

--72.3
F3C
O
N
O
NH
N
4c
19F NMR 377 MHz, CDCl3
20
0
−10
−30
−50
−70
−90
−110
−140
−170
−200
f1 (ppm)

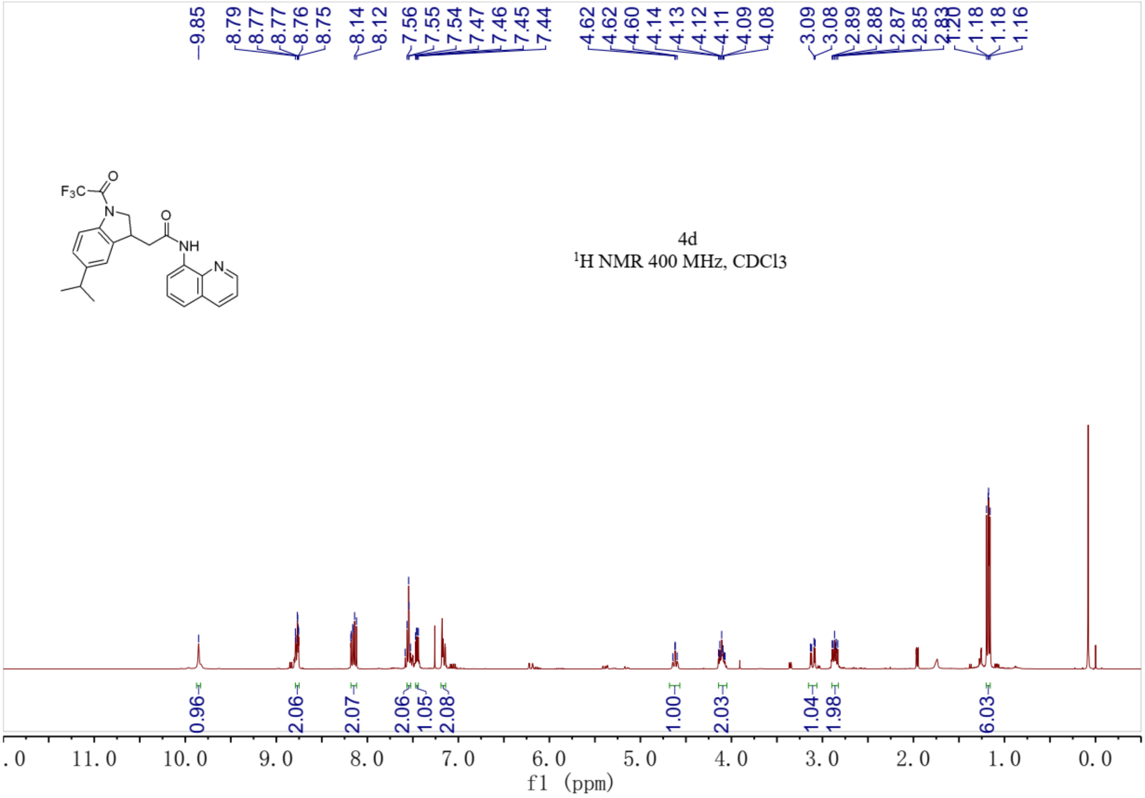
4d
1H NMR 400 MHz, CDCl3
9.85
8.79
8.77
8.77
8.76
8.75
8.14
8.12
7.56
7.55
7.54
7.47
7.46
7.45
7.44
4.62
4.62
4.60
4.14
4.13
4.12
4.11
4.09
4.08
3.09
3.08
2.89
2.88
2.87
2.85
2.83
1.20
1.18
1.18
1.16
0.96
2.06
2.07
2.06
1.05
2.08
1.00
2.03
1.04
1.98
6.03
f1 (ppm)

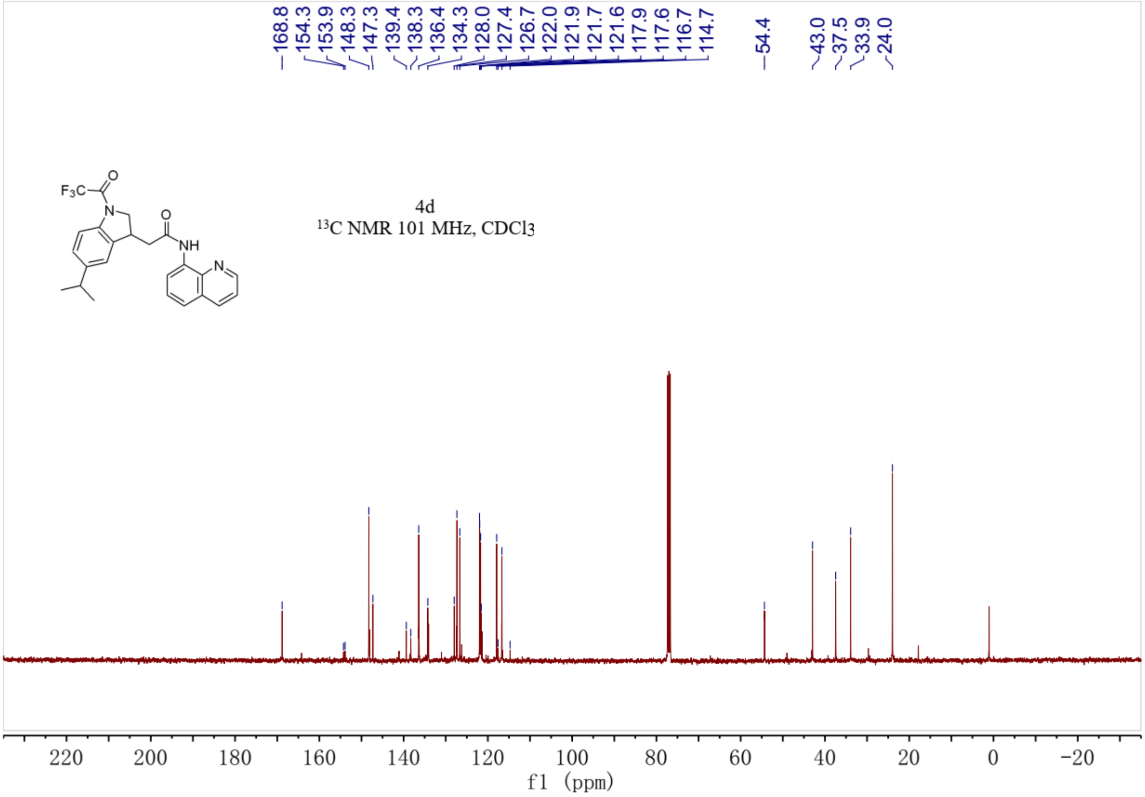
4d
13C NMR 101 MHz, CDCl3
168.8
154.3
153.9
148.3
147.3
139.4
138.3
136.4
134.3
128.0
127.4
126.7
122.0
121.9
121.7
121.6
117.9
117.6
116.7
114.7
54.4
43.0
37.5
33.9
24.0
f1 (ppm)

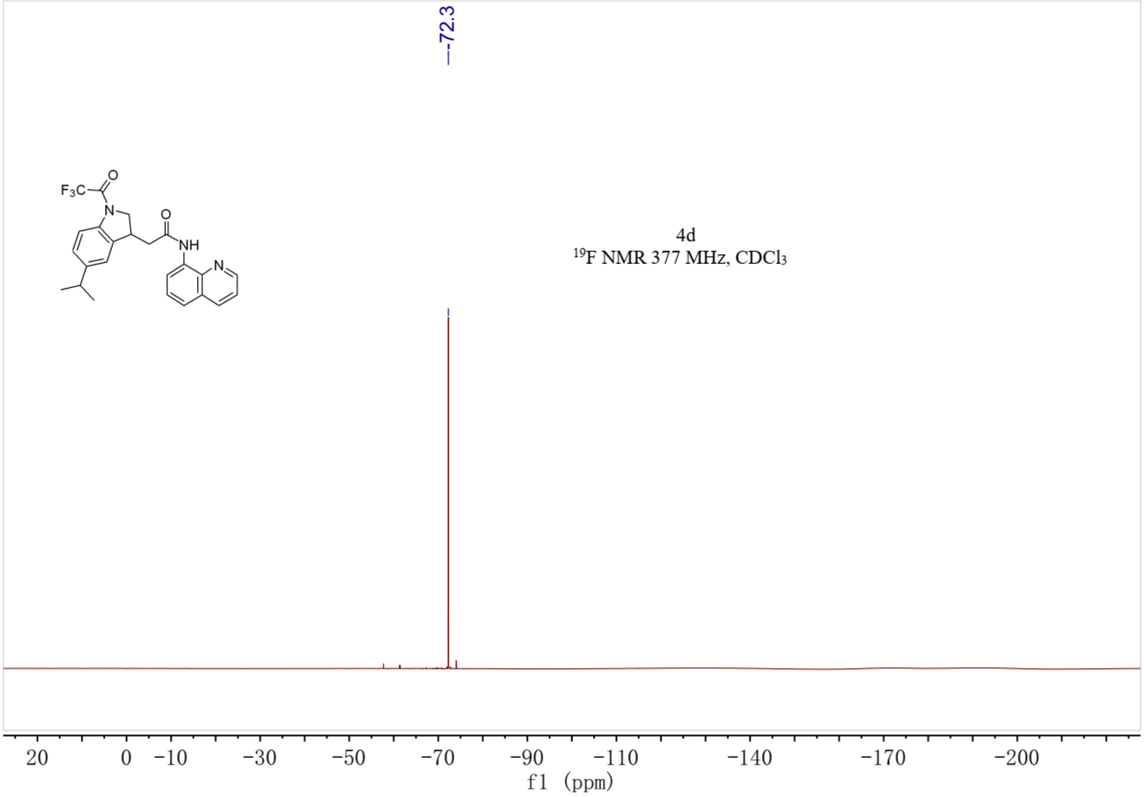
-72.3
F_3C
O
N
O
NH
N
4d
^{19}F NMR 377 MHz, $CDCl_3$
20
0
−10
−30
−50
−70
−90
−110
−140
−170
−200
f1 (ppm)

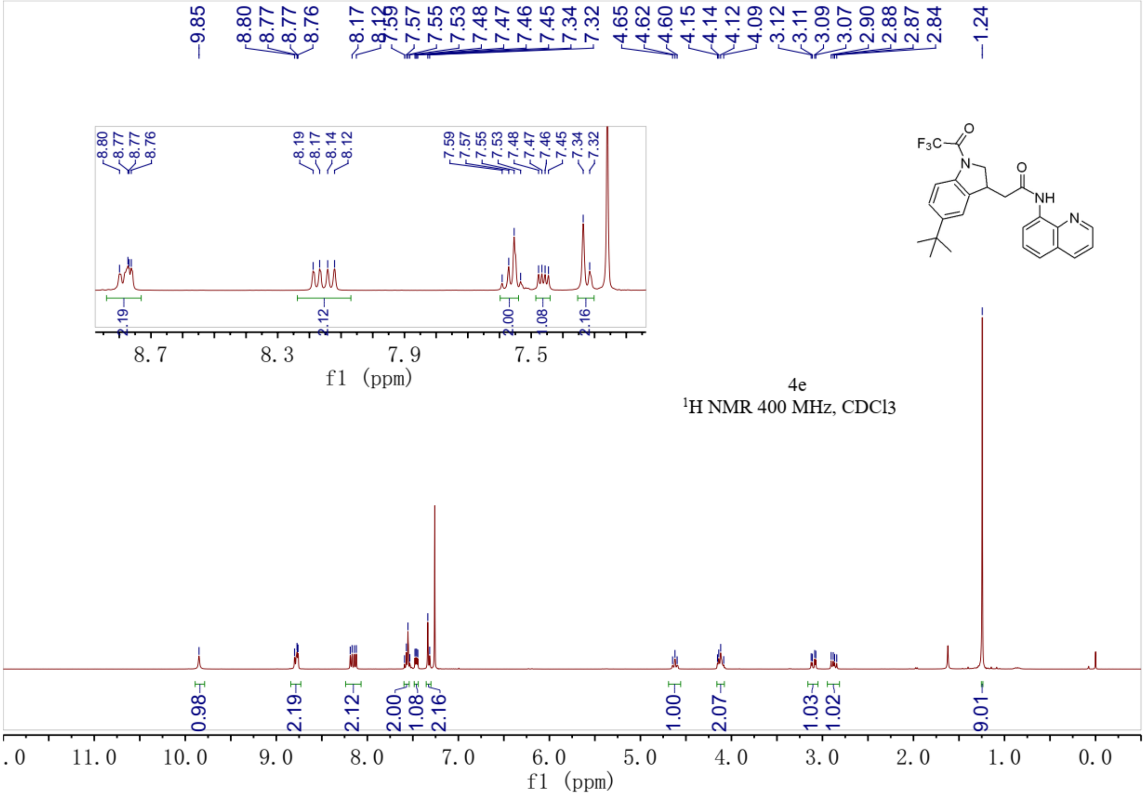
4e
^{1}H NMR 400 MHz, CDCl3
f1 (ppm)

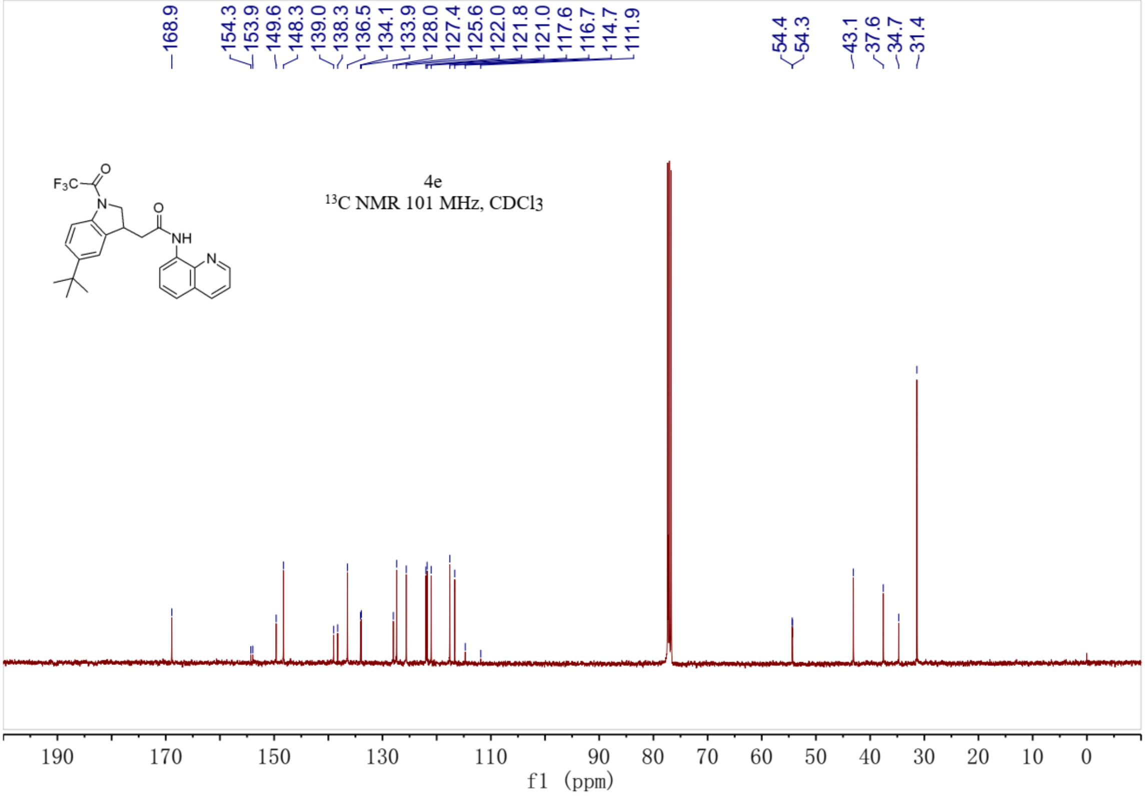
4e
^{13}C NMR 101 MHz, CDCl3
f1 (ppm)

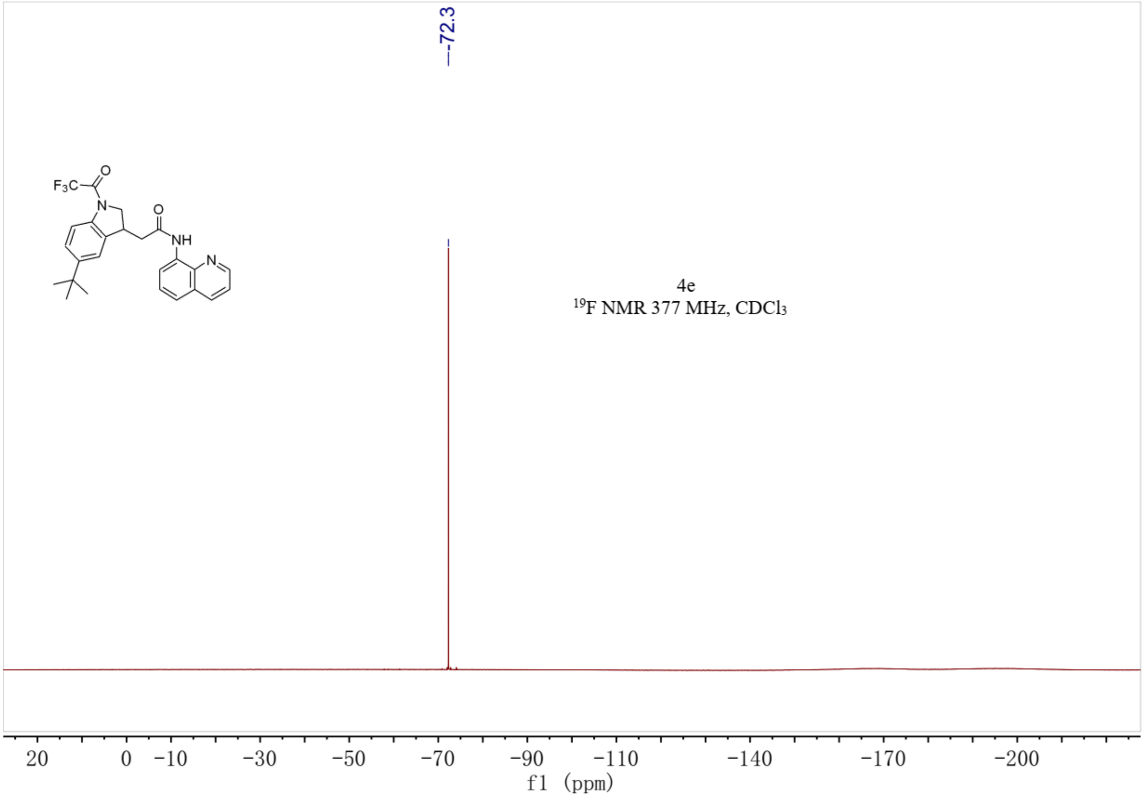

-72.3
4e
19F NMR 377 MHz, CDCl3
20
0
−10
−30
−50
−70
−90
−110
−140
−170
−200
f1 (ppm)

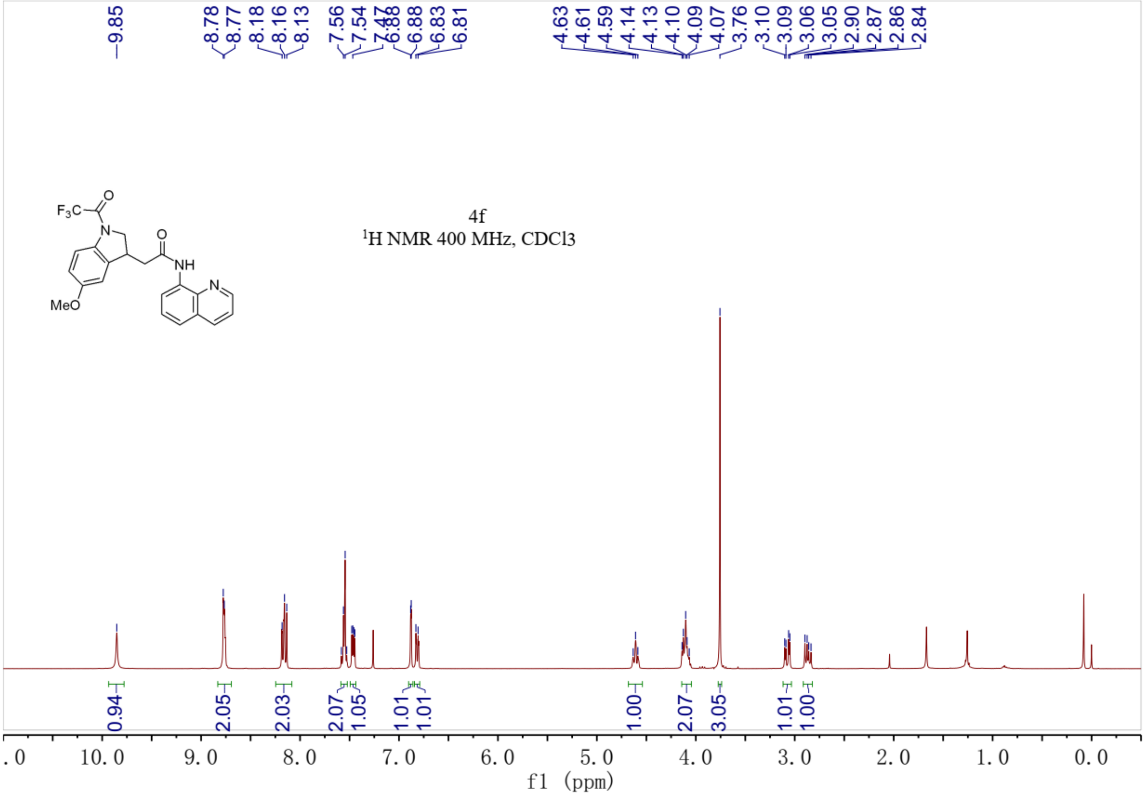
9.85
8.78
8.77
8.18
8.16
8.13
7.56
7.54
7.47
6.88
6.83
6.81
4.63
4.61
4.59
4.14
4.13
4.10
4.09
4.07
3.76
3.10
3.09
3.06
3.05
2.90
2.87
2.86
2.84
4f
^{1}H NMR 400 MHz, CDCl3
0.94
2.05
2.03
2.07
1.05
1.01
1.01
1.00
2.07
3.05
1.01
1.00
f1 (ppm)

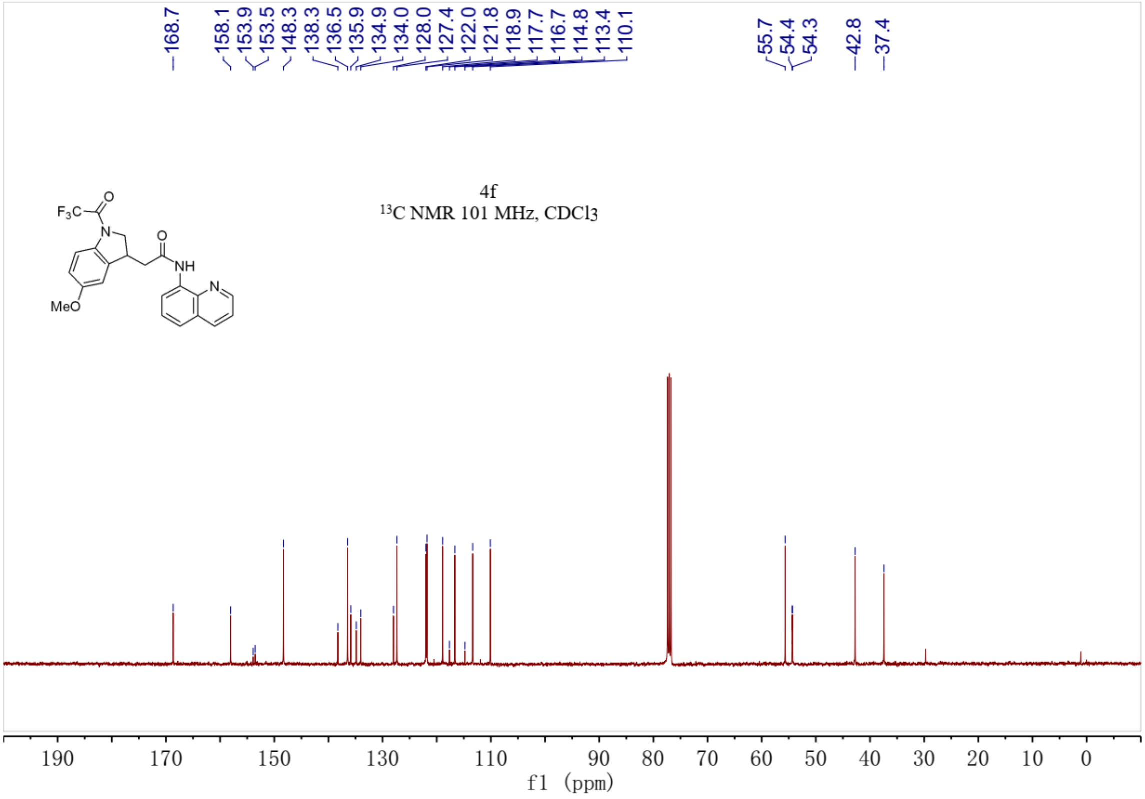
168.7
158.1
153.9
153.5
148.3
138.3
136.5
135.9
134.9
134.0
128.0
127.4
122.0
121.8
118.9
117.7
116.7
114.8
113.4
110.1
55.7
54.4
54.3
42.8
37.4
4f
^{13}C NMR 101 MHz, CDCl3
f1 (ppm)

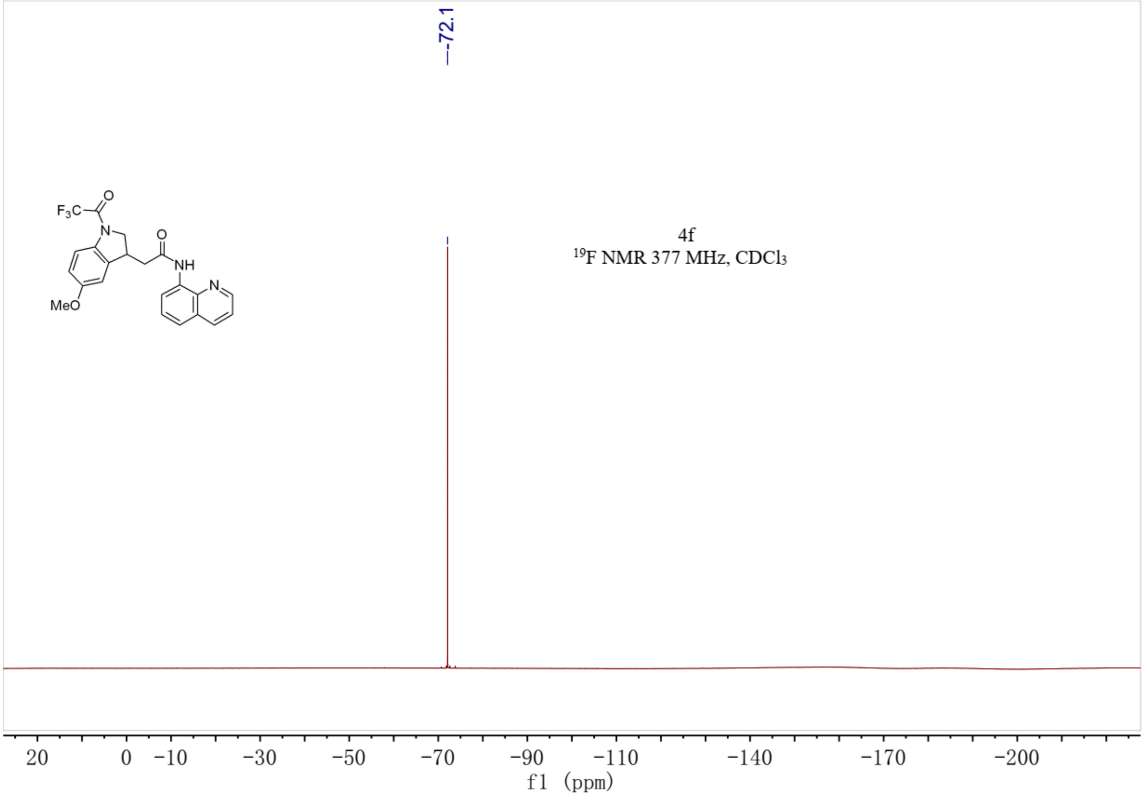

-72.1
F3C
O
N
O
NH
N
MeO
4f
19F NMR 377 MHz, CDCl3
20
0
−10
−30
−50
−70
−90
−110
−140
−170
−200
f1 (ppm)

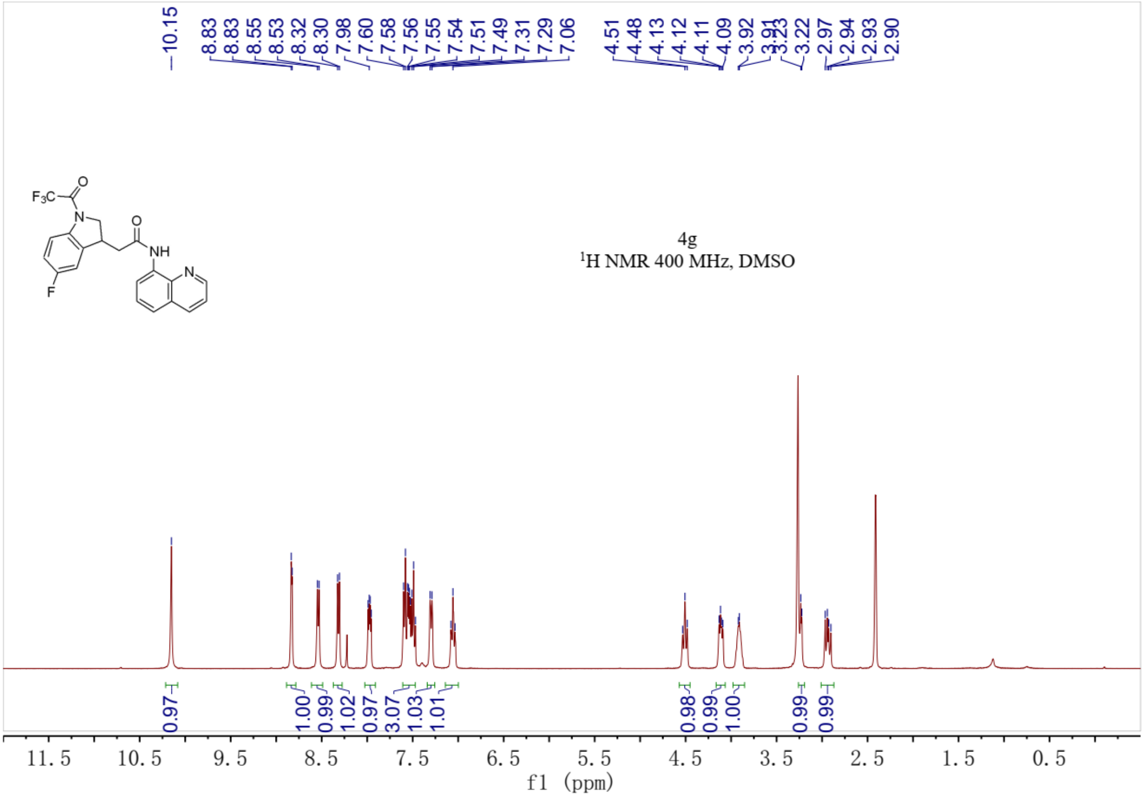
4g
1H NMR 400 MHz, DMSO
f1 (ppm)

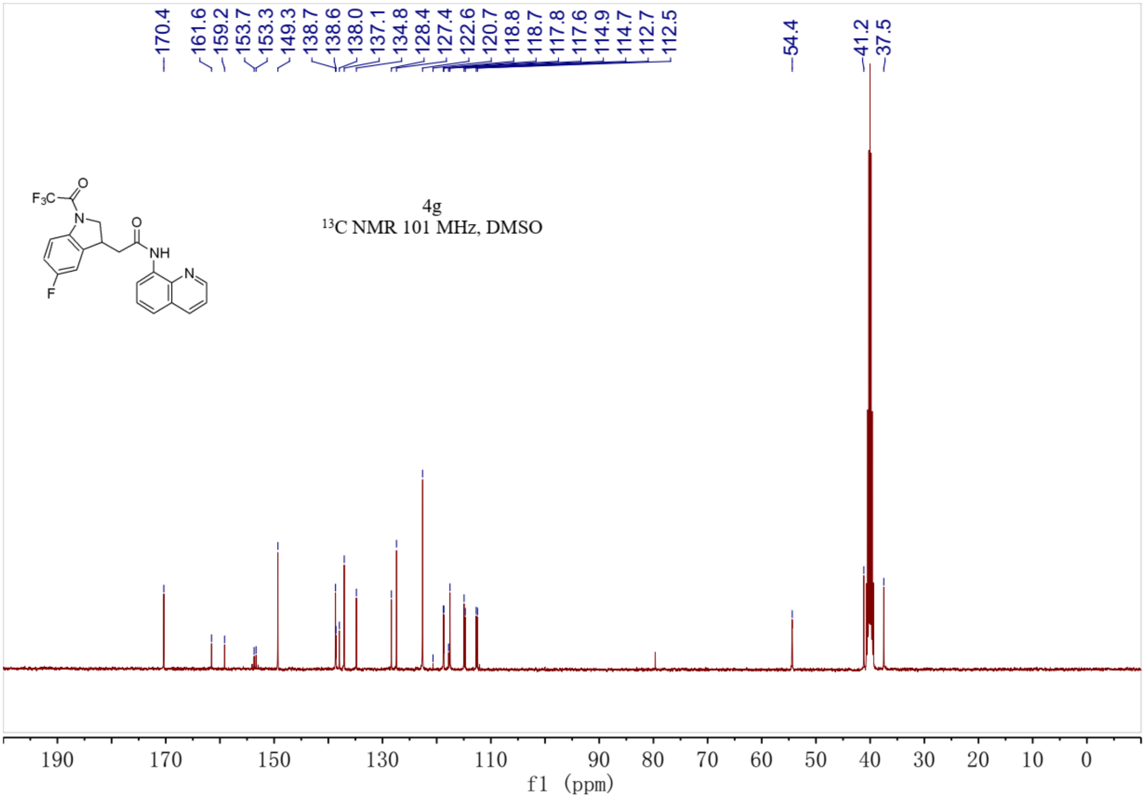
4g
13C NMR 101 MHz, DMSO
f1 (ppm)

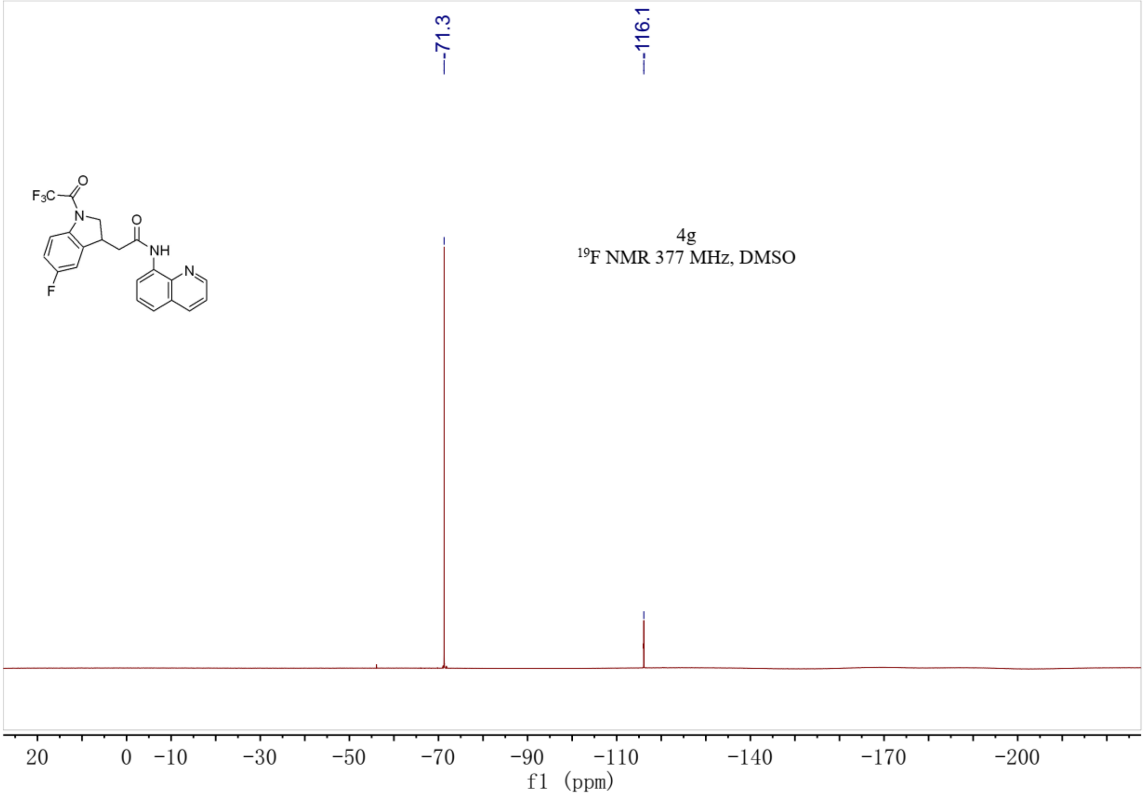

-71.3
-116.1
4g
19F NMR 377 MHz, DMSO
20
0
−10
−30
−50
−70
−90
−110
−140
−170
−200
f1 (ppm)

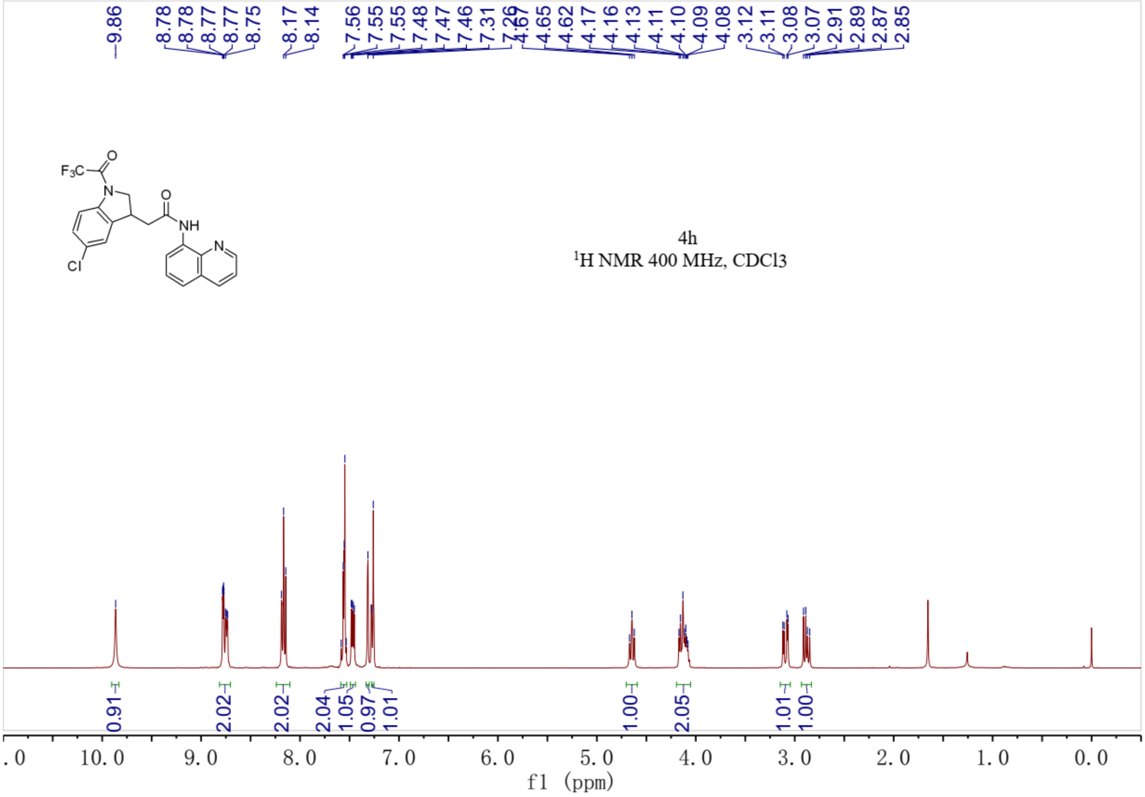

9.86
8.78
8.78
8.77
8.77
8.75
8.17
8.14
7.56
7.55
7.55
7.48
7.47
7.46
7.31
7.26
4.67
4.65
4.62
4.17
4.16
4.13
4.11
4.10
4.09
4.08
3.12
3.11
3.08
3.07
2.91
2.89
2.87
2.85
F3C
O
N
O
NH
N
Cl
4h
1H NMR 400 MHz, CDCl3
0.91
2.02
2.02
2.04
1.05
0.97
1.01
1.00
2.05
1.01
1.00
10.0
9.0
8.0
7.0
6.0
5.0
4.0
3.0
2.0
1.0
0.0
f1 (ppm)

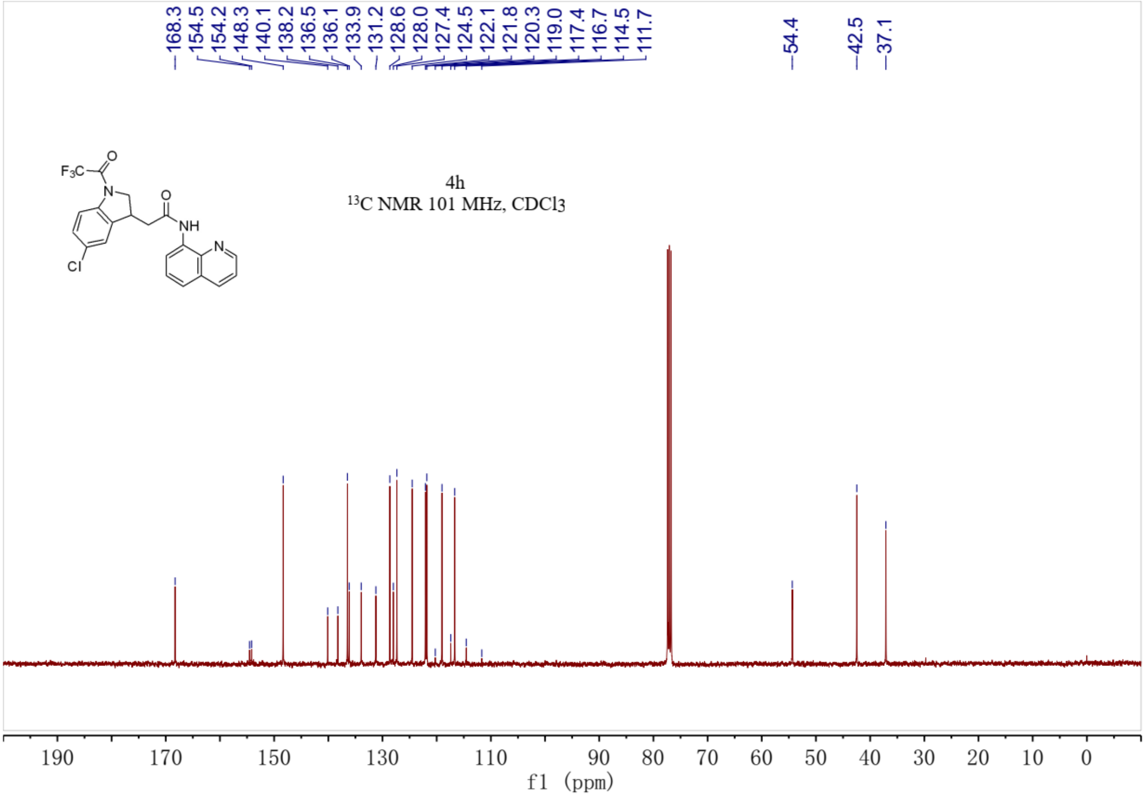

168.3
154.5
154.2
148.3
140.1
138.2
136.5
136.1
133.9
131.2
128.6
128.0
127.4
124.5
122.1
121.8
120.3
119.0
117.4
116.7
114.5
111.7
54.4
42.5
37.1
F3C
O
N
O
NH
N
Cl
4h
13C NMR 101 MHz, CDCl3
190
170
150
130
110
90
80
70
60
50
40
30
20
10
0
f1 (ppm)

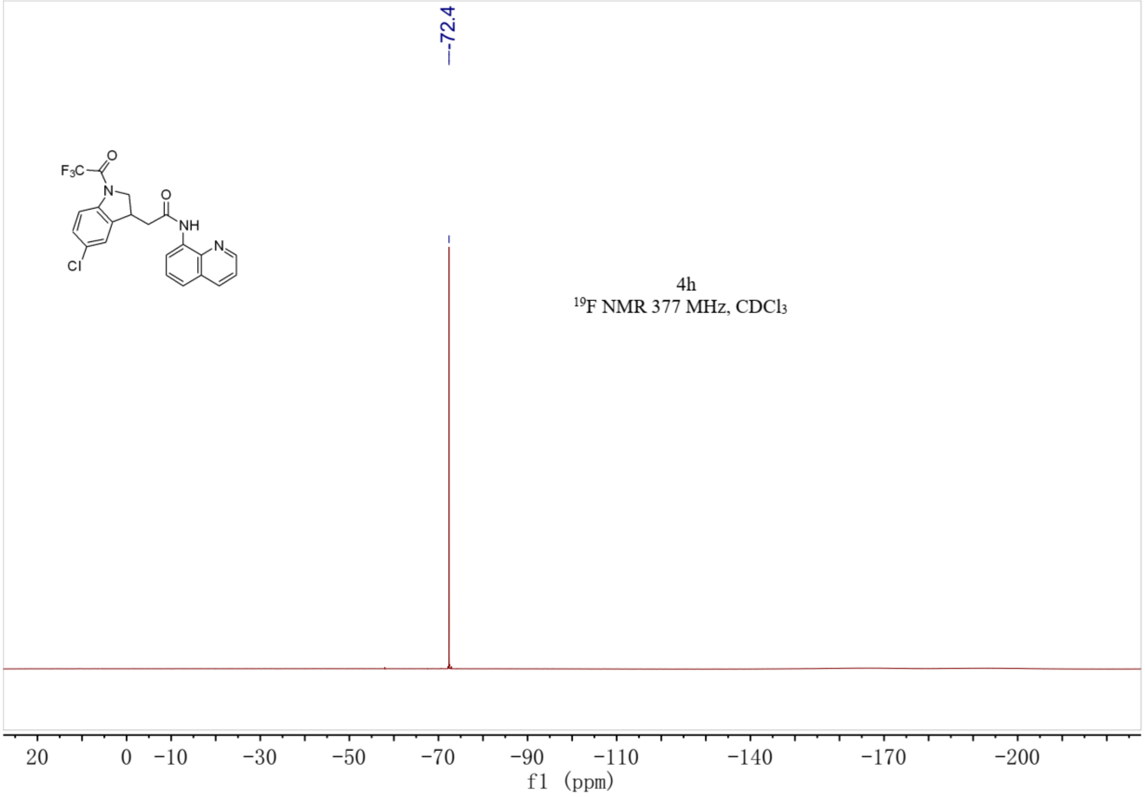

-72.4
4h
19F NMR 377 MHz, CDCl3
20
0
−10
−30
−50
−70
−90
−110
−140
−170
−200
f1 (ppm)

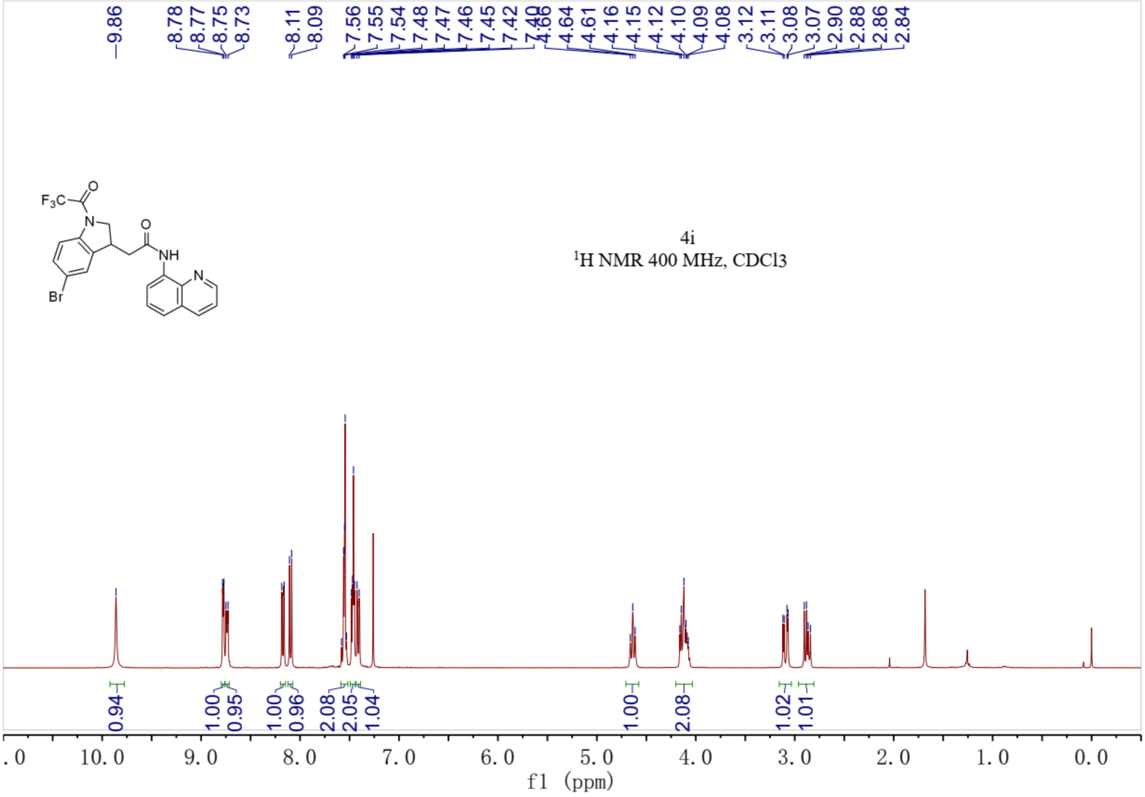
4i
^{1}H NMR 400 MHz, CDCl3
9.86
8.78
8.77
8.75
8.73
8.11
8.09
7.56
7.55
7.54
7.48
7.47
7.46
7.45
7.42
7.40
4.66
4.64
4.61
4.16
4.15
4.12
4.10
4.09
4.08
3.12
3.11
3.08
3.07
2.90
2.88
2.86
2.84
0.94
1.00
0.95
1.00
0.96
2.08
2.05
1.04
1.00
2.08
1.02
1.01
f1 (ppm)

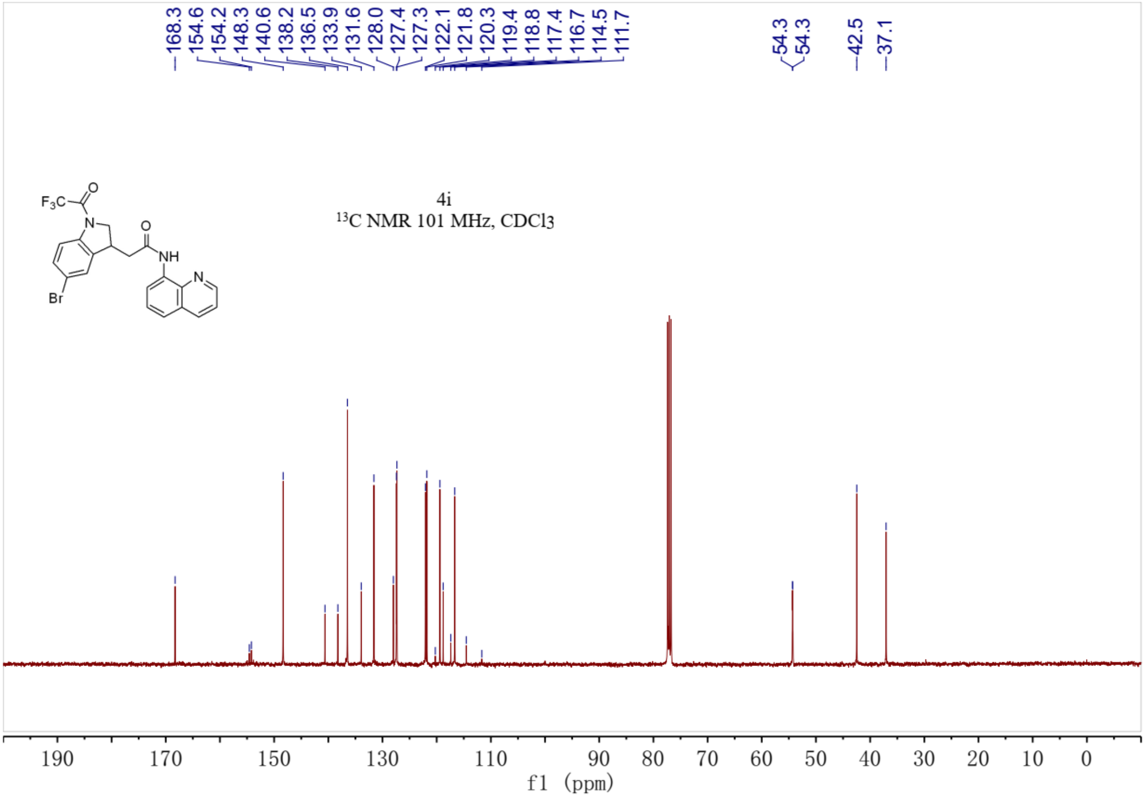
4i
^{13}C NMR 101 MHz, CDCl3
168.3
154.6
154.2
148.3
140.6
138.2
136.5
133.9
131.6
128.0
127.4
127.3
122.1
121.8
120.3
119.4
118.8
117.4
116.7
114.5
111.7
54.3
54.3
42.5
37.1
f1 (ppm)

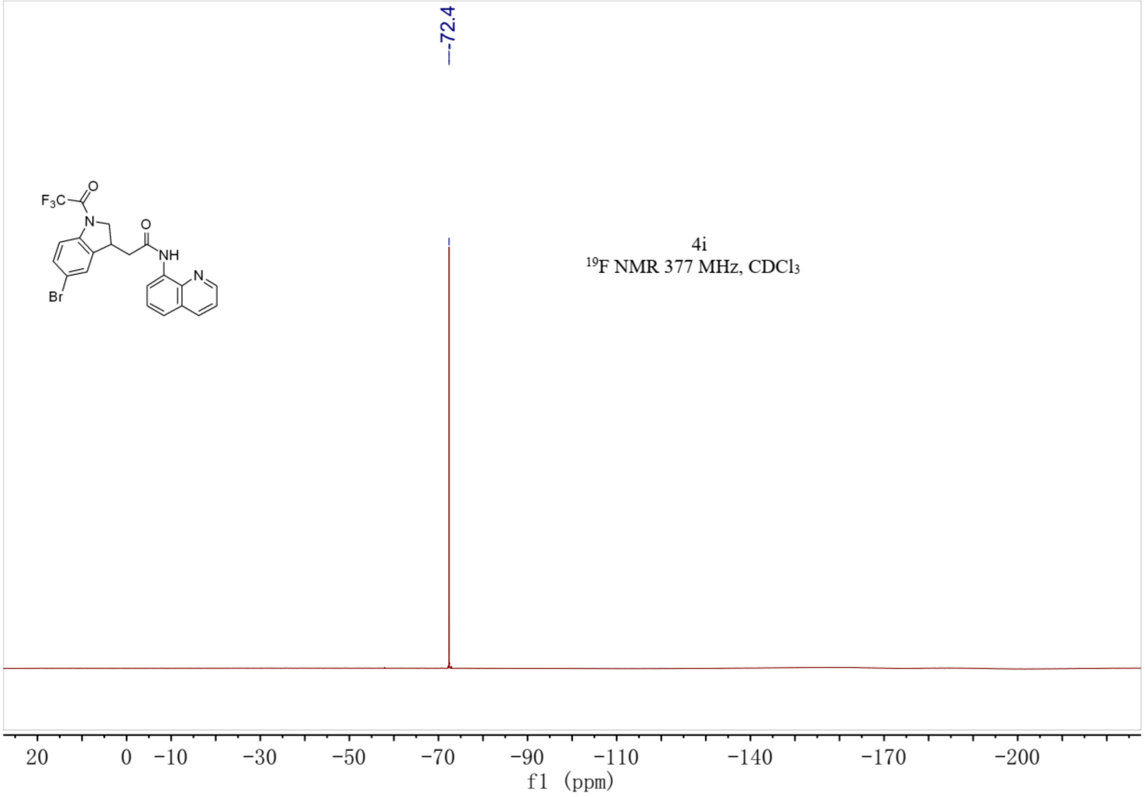
-72.4
4i
19F NMR 377 MHz, CDCl3
F3C
O
N
O
NH
N
Br
20
0
−10
−30
−50
−70
−90
−110
−140
−170
−200
f1 (ppm)

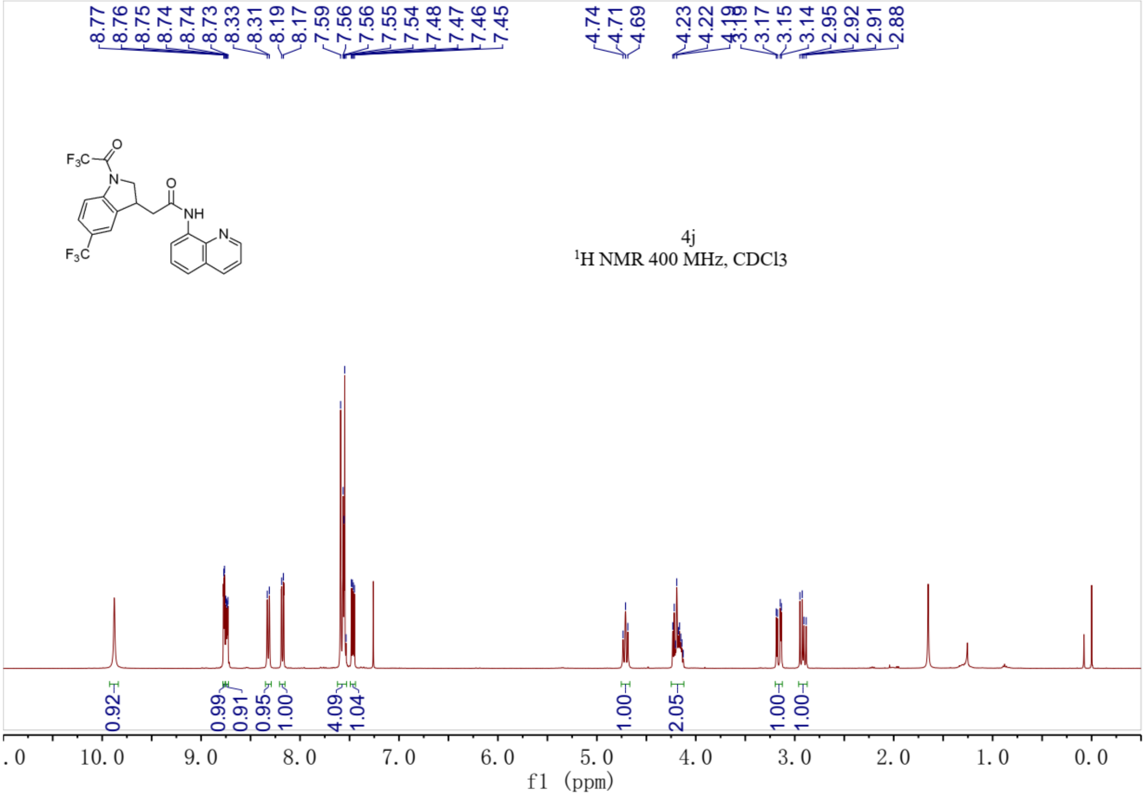
8.77
8.76
8.75
8.74
8.74
8.73
8.33
8.31
8.19
8.17
7.59
7.56
7.56
7.55
7.54
7.48
7.47
7.46
7.45
4.74
4.71
4.69
4.23
4.22
4.19
3.17
3.15
3.14
2.95
2.92
2.91
2.88
4j
¹H NMR 400 MHz, CDCl3
0.92
0.99
0.91
0.95
1.00
4.09
1.04
1.00
2.05
1.00
1.00
f1 (ppm)

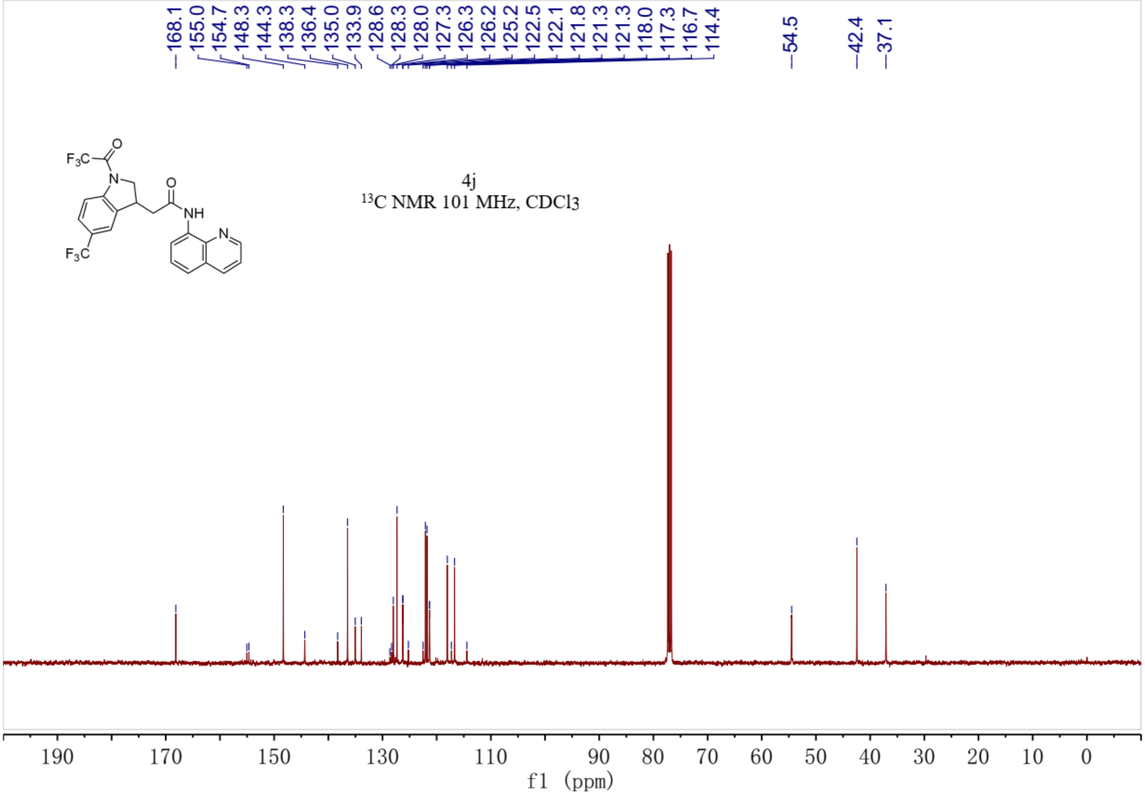
168.1
155.0
154.7
148.3
144.3
138.3
136.4
135.0
133.9
128.6
128.3
128.0
127.3
126.3
126.2
125.2
122.5
122.1
121.8
121.3
121.3
118.0
117.3
116.7
114.4
54.5
42.4
37.1
4j
¹³C NMR 101 MHz, CDCl3
f1 (ppm)

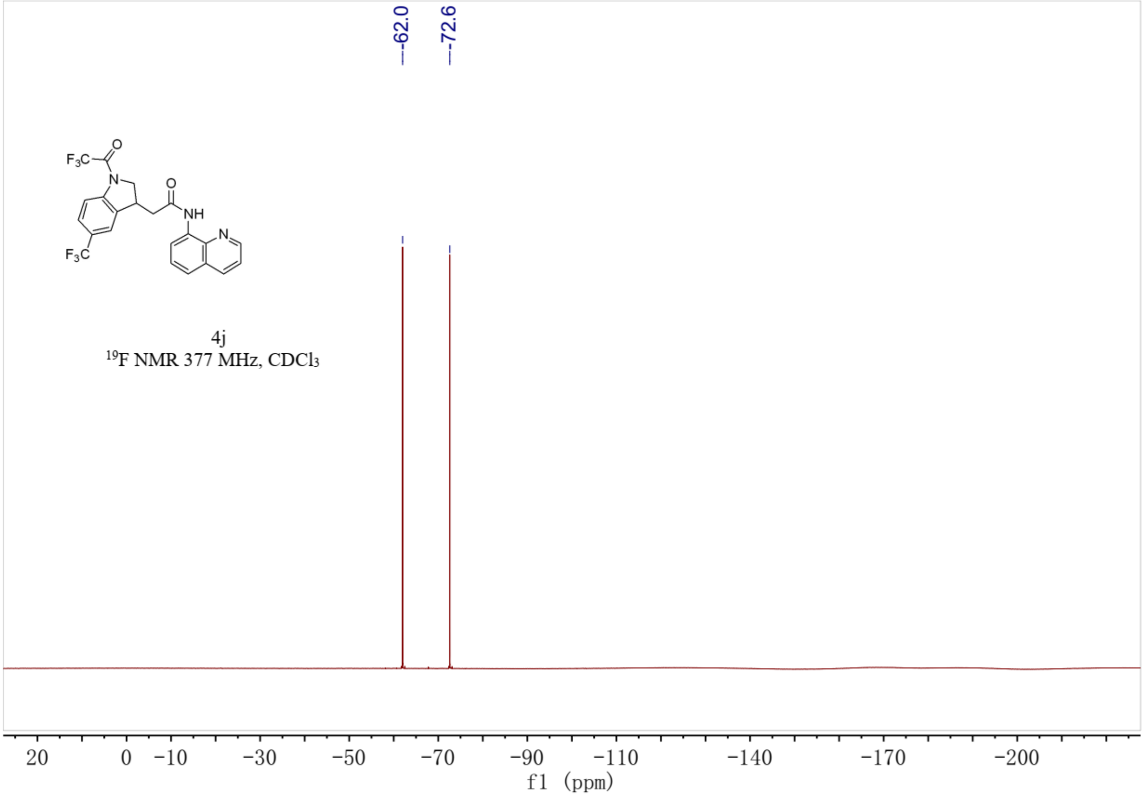

4j
^{19}F NMR 377 MHz, $CDCl_3$

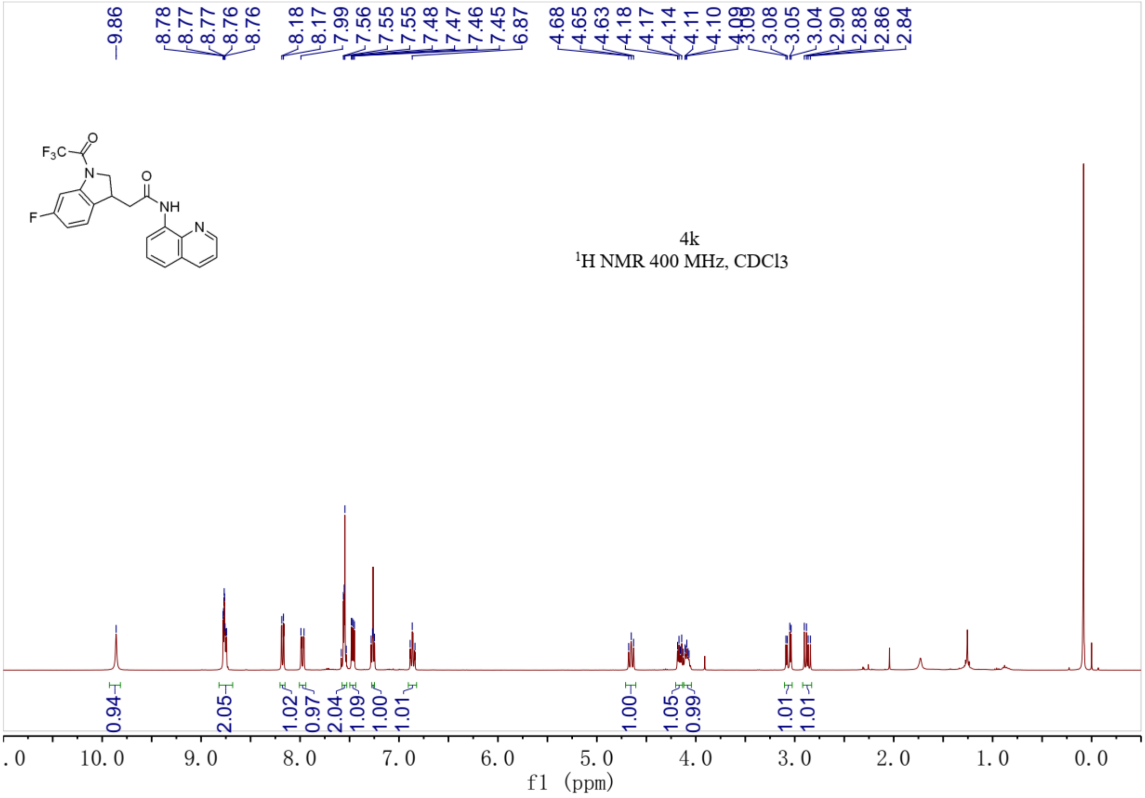
4k
1H NMR 400 MHz, CDCl3
f1 (ppm)

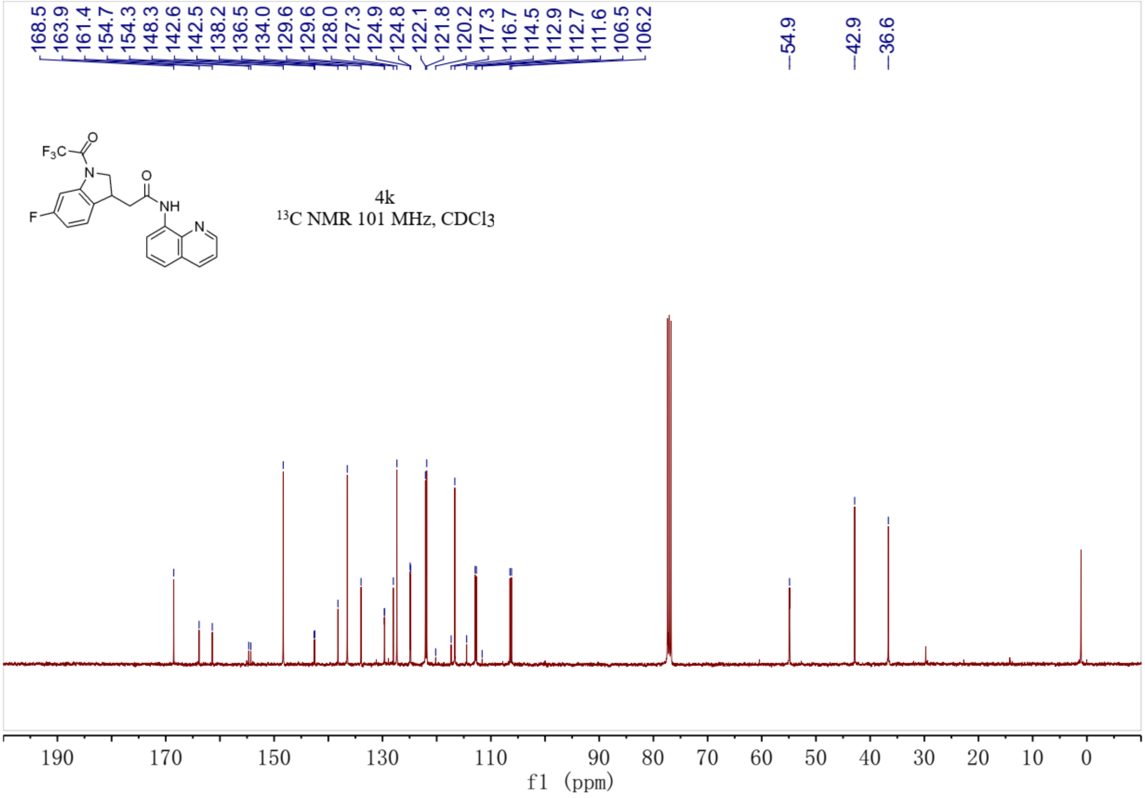
4k
13C NMR 101 MHz, CDCl3
f1 (ppm)

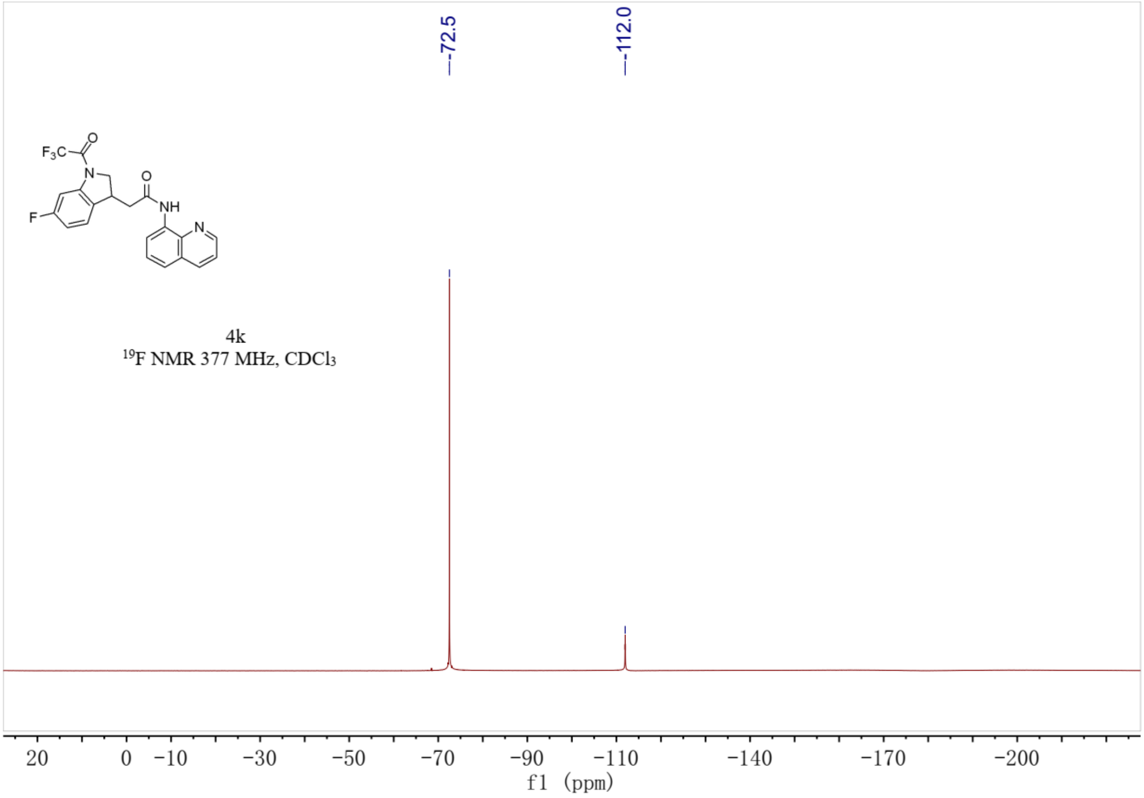
-72.5
-112.0
F3C
O
N
O
NH
F
N
4k
19F NMR 377 MHz, CDCl3
20
0
−10
−30
−50
−70
−90
−110
−140
−170
−200
f1 (ppm)

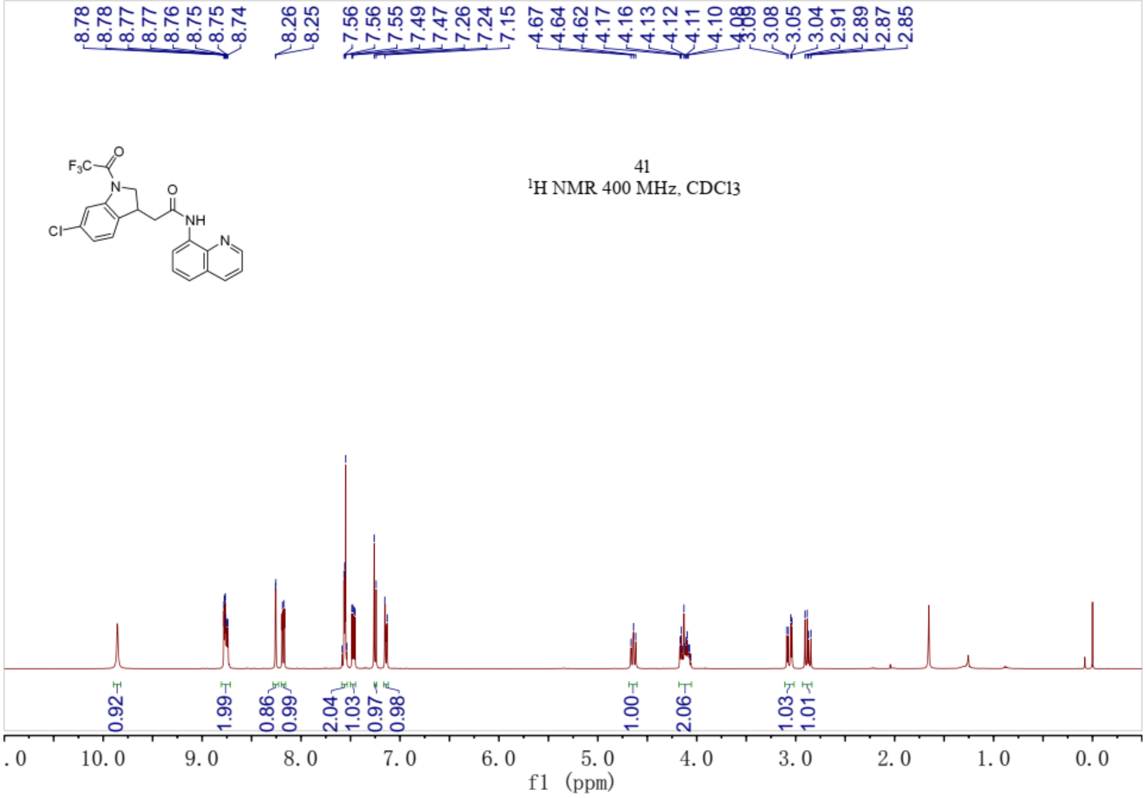
8.78
8.78
8.77
8.77
8.76
8.75
8.75
8.74
8.26
8.25
7.56
7.56
7.55
7.49
7.47
7.26
7.24
7.15
4.67
4.64
4.62
4.17
4.16
4.13
4.12
4.11
4.10
3.09
3.08
3.05
3.04
2.91
2.89
2.87
2.85
41
^{1}H NMR 400 MHz, CDCl3
0.92
1.99
0.86
0.99
2.04
1.03
0.97
0.98
1.00
2.06
1.03
1.01
10.0
9.0
8.0
7.0
6.0
5.0
4.0
3.0
2.0
1.0
0.0
f1 (ppm)

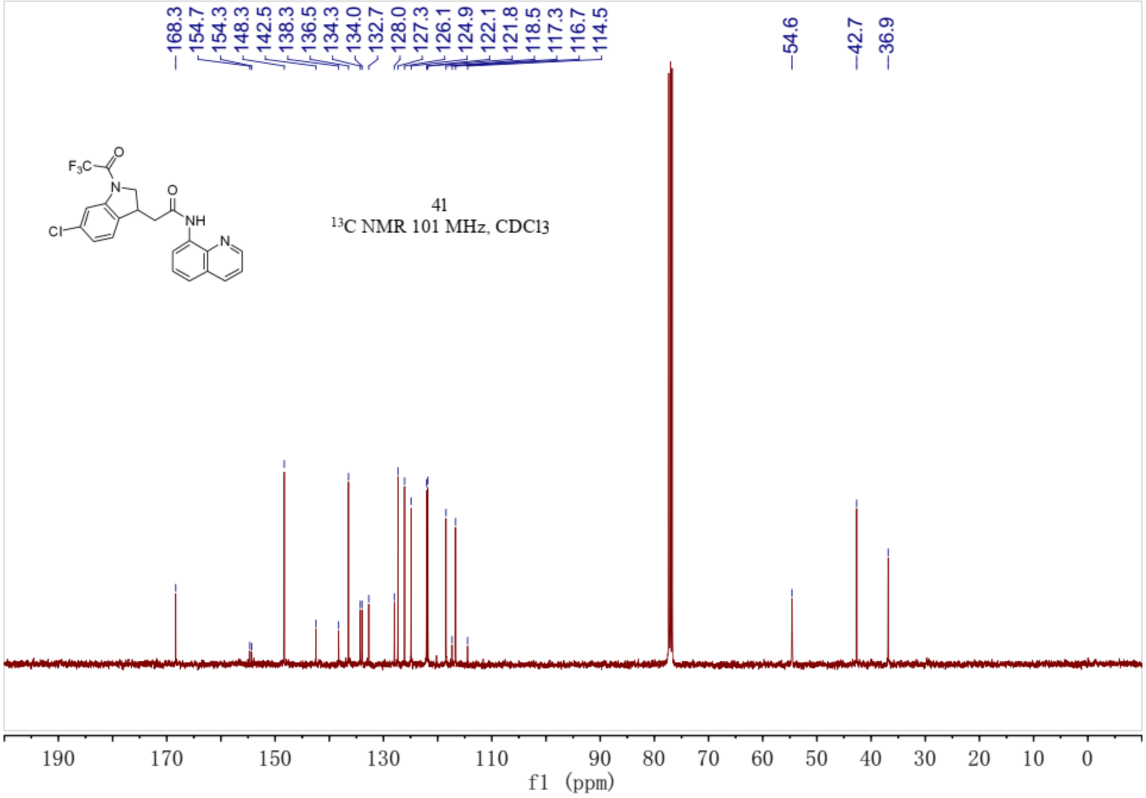
168.3
154.7
154.3
148.3
142.5
138.3
136.5
134.3
134.0
132.7
128.0
127.3
126.1
124.9
122.1
121.8
118.5
117.3
116.7
114.5
54.6
42.7
36.9
41
^{13}C NMR 101 MHz, CDCl3
190
170
150
130
110
90
80
70
60
50
40
30
20
10
0
f1 (ppm)

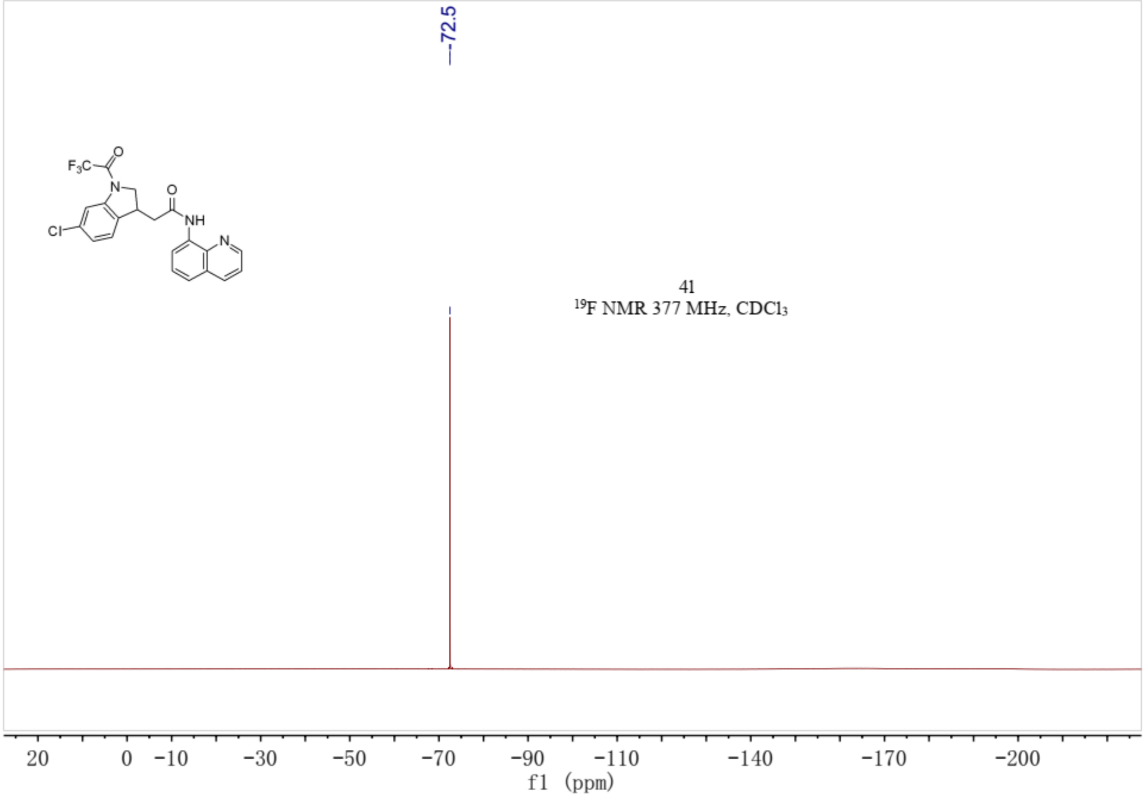
-72.5
F3C
O
N
O
NH
N
Cl
41
19F NMR 377 MHz, CDCl3
20
0
−10
−30
−50
−70
−90
−110
−140
−170
−200
f1 (ppm)

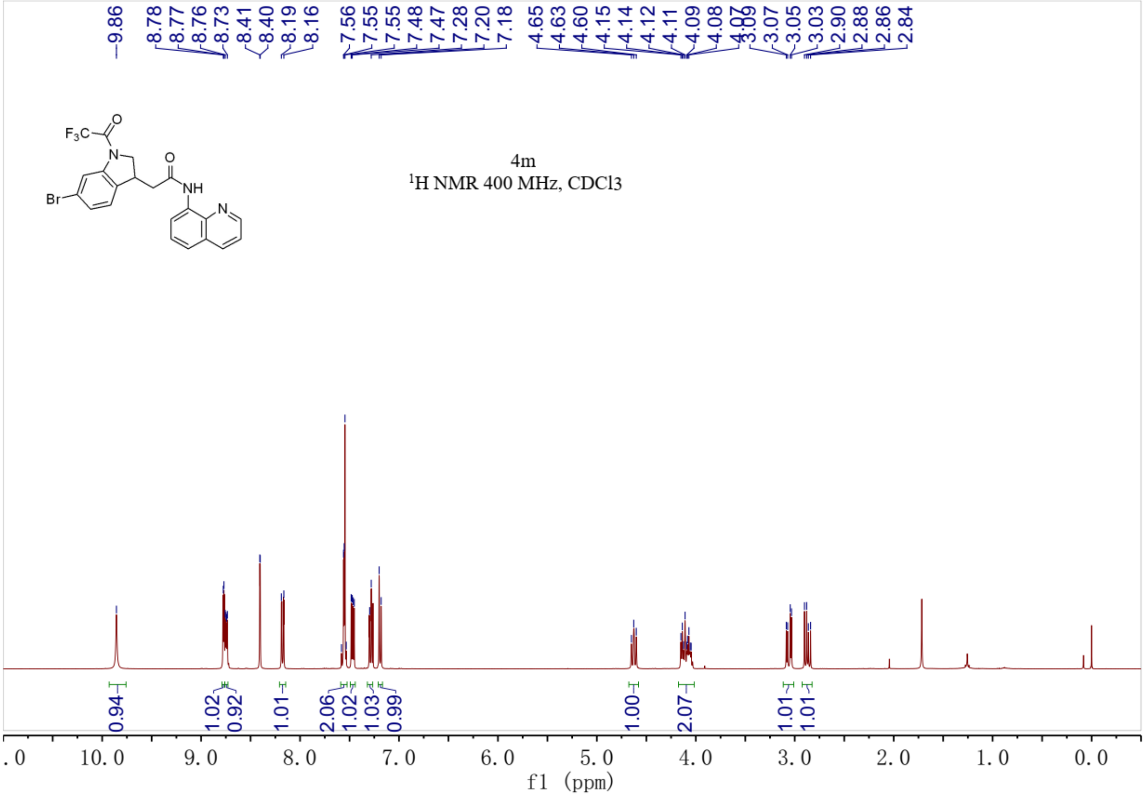
9.86
8.78
8.77
8.76
8.73
8.41
8.40
8.19
8.16
7.56
7.55
7.55
7.48
7.47
7.28
7.20
7.18
4.65
4.63
4.60
4.15
4.14
4.12
4.11
4.09
4.08
4.07
3.09
3.07
3.05
3.03
2.90
2.88
2.86
2.84
F3C
O
N
O
NH
Br
N
4m
1H NMR 400 MHz, CDCl3
0.94
1.02
0.92
1.01
2.06
1.02
1.03
0.99
1.00
2.07
1.01
1.01
10.0
9.0
8.0
7.0
6.0
5.0
4.0
3.0
2.0
1.0
0.0
f1 (ppm)

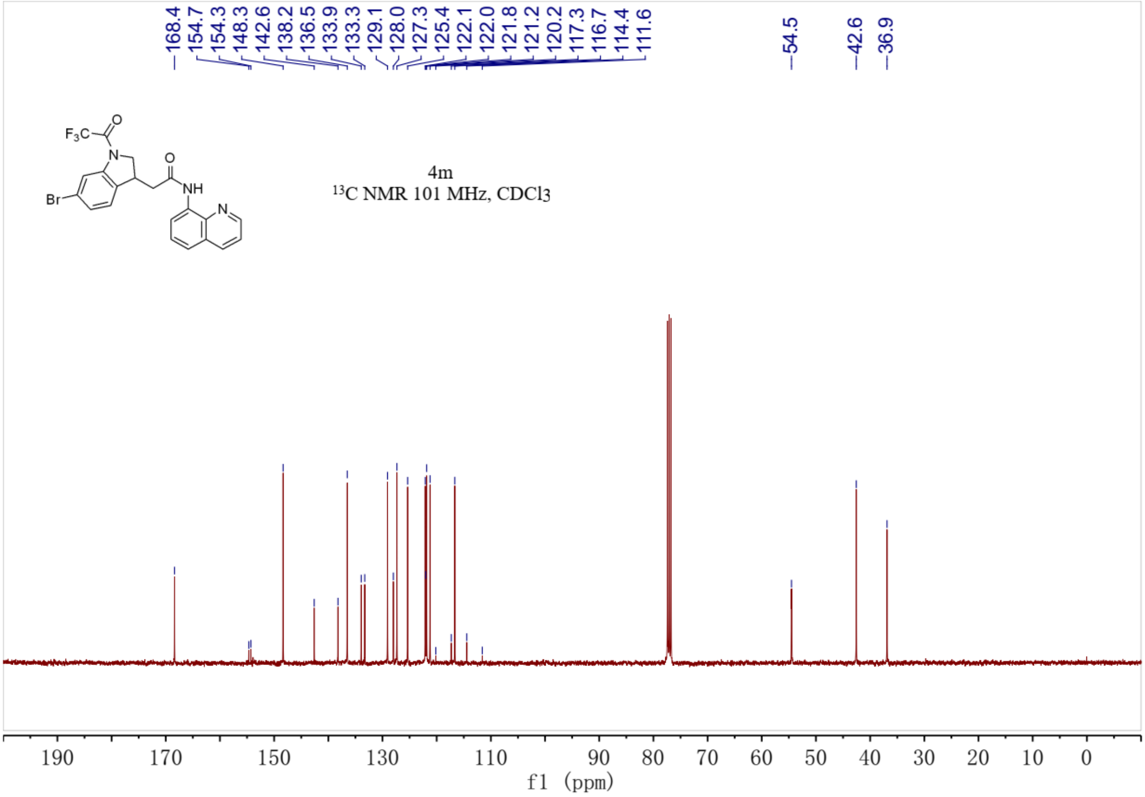
168.4
154.7
154.3
148.3
142.6
138.2
136.5
133.9
133.3
129.1
128.0
127.3
125.4
122.1
122.0
121.8
121.2
120.2
117.3
116.7
114.4
111.6
54.5
42.6
36.9
F3C
O
N
O
NH
Br
N
4m
13C NMR 101 MHz, CDCl3
190
170
150
130
110
90
80
70
60
50
40
30
20
10
0
f1 (ppm)

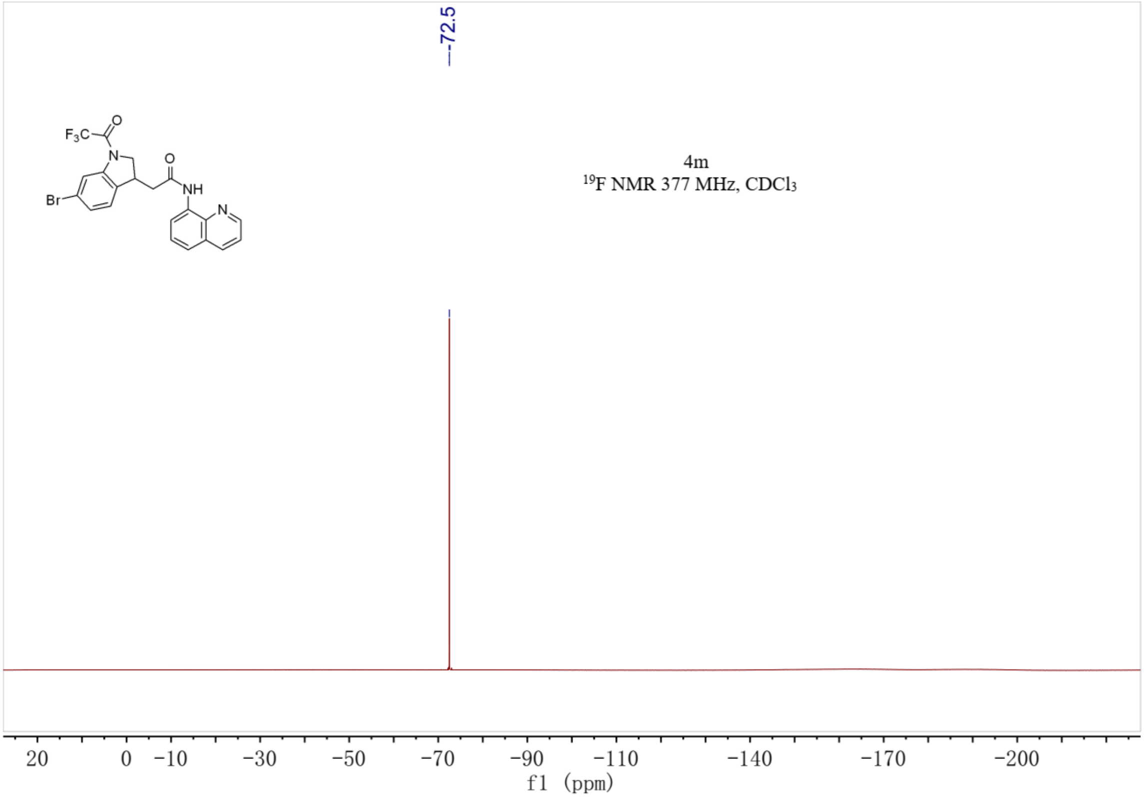

-72.5
F3C
O
N
O
NH
Br
N
4m
19F NMR 377 MHz, CDCl3
20
0
−10
−30
−50
−70
−90
−110
−140
−170
−200
f1 (ppm)

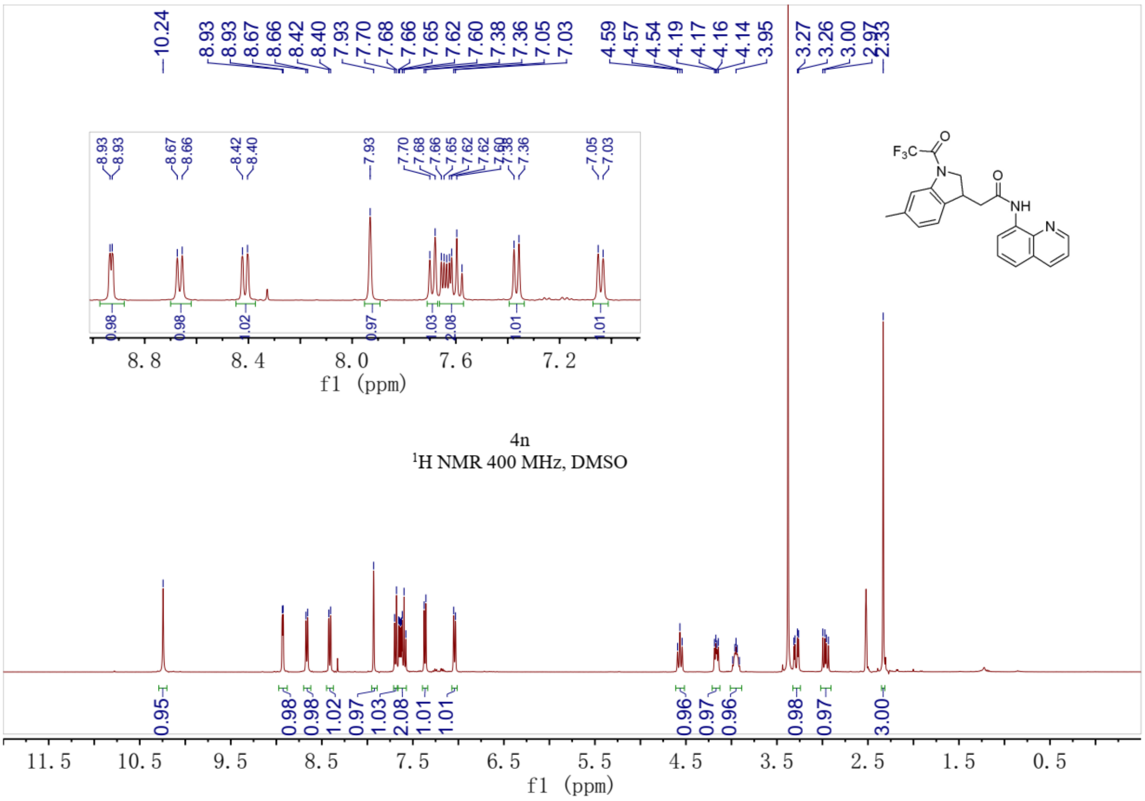
4n
^{1}H NMR 400 MHz, DMSO
f1 (ppm)

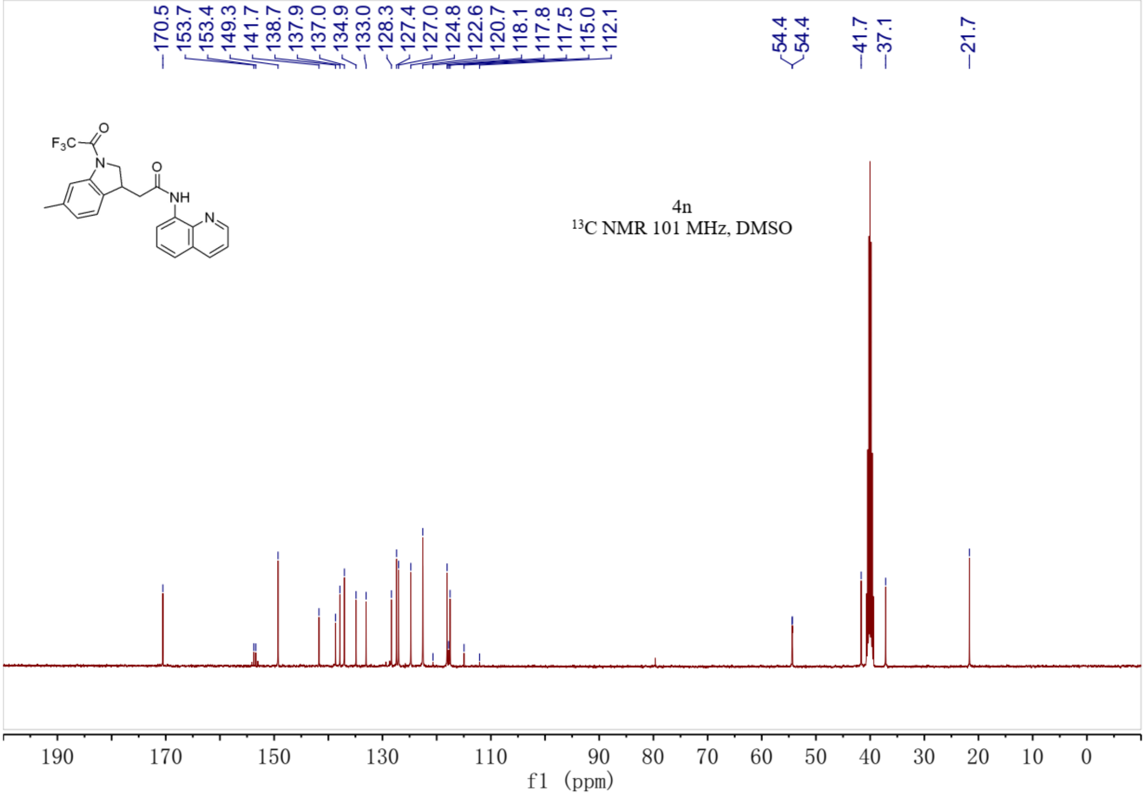
4n
^{13}C NMR 101 MHz, DMSO
f1 (ppm)

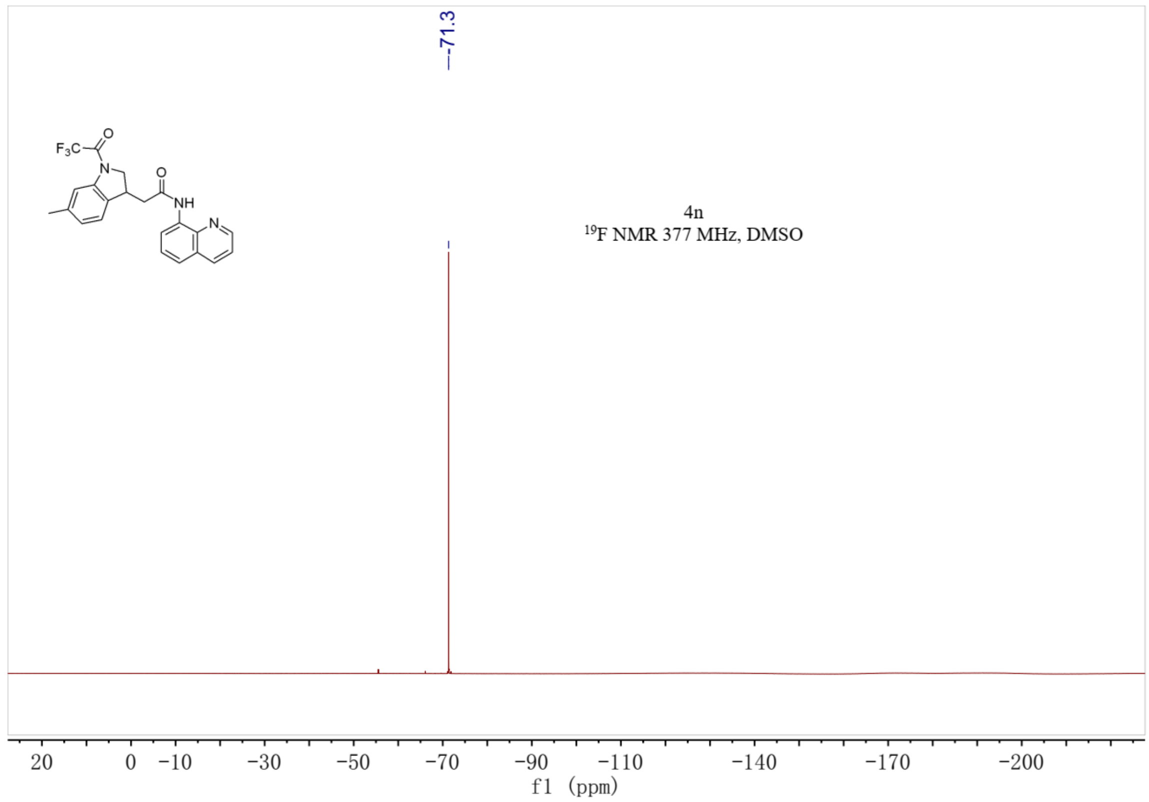

-71.3
F_3C
O
N
O
NH
N
4n
19F NMR 377 MHz, DMSO
20
0
−10
−30
−50
−70
−90
−110
−140
−170
−200
f1 (ppm)

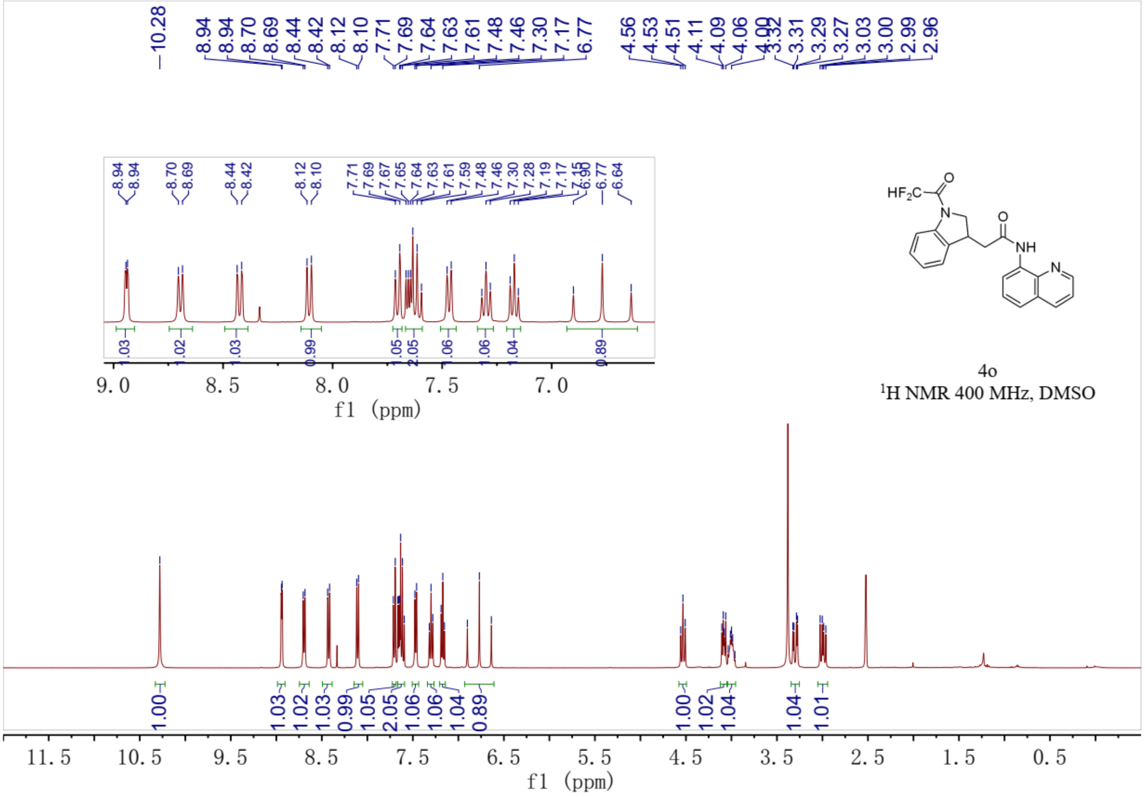
10.28
8.94
8.94
8.70
8.69
8.44
8.42
8.12
8.10
7.71
7.69
7.64
7.63
7.61
7.48
7.46
7.30
7.17
6.77
4.56
4.53
4.51
4.11
4.09
4.06
3.31
3.29
3.27
3.03
3.00
2.99
2.96
HF2C
O
N
O
NH
N
4o
1H NMR 400 MHz, DMSO
f1 (ppm)

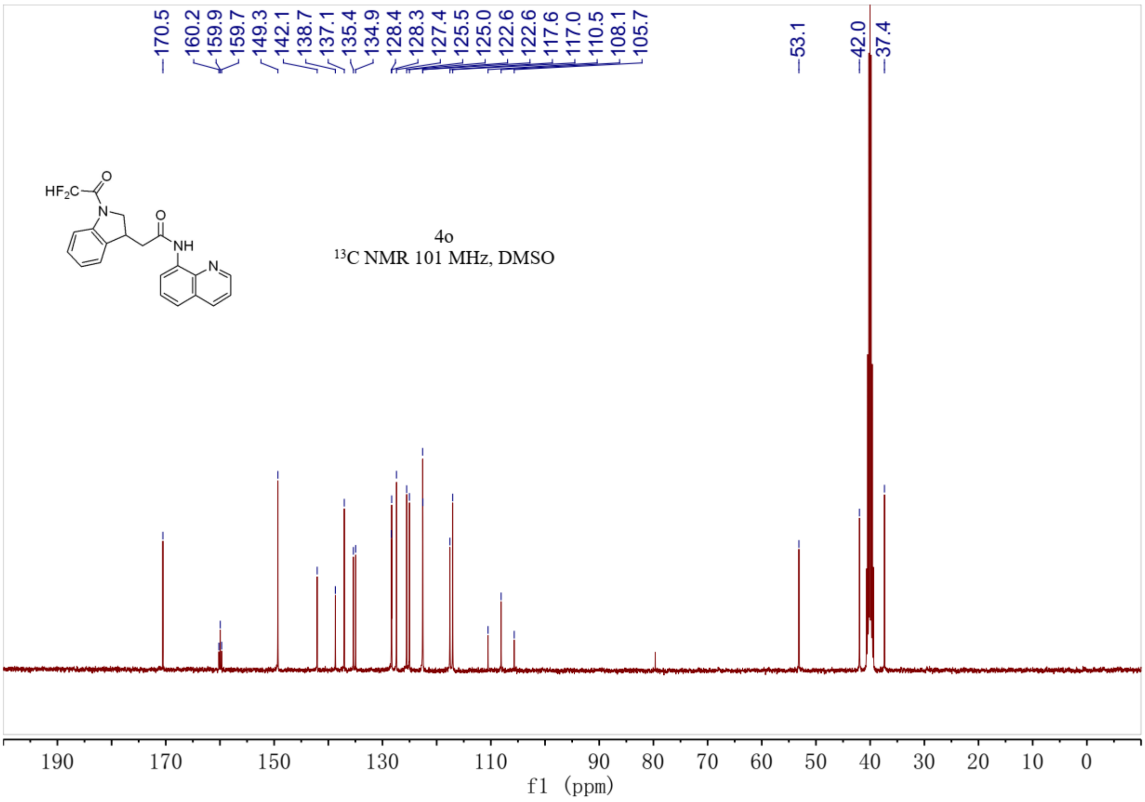
170.5
160.2
159.9
159.7
149.3
142.1
138.7
137.1
135.4
134.9
128.4
128.3
127.4
125.5
125.0
122.6
122.6
117.6
117.0
110.5
108.1
105.7
53.1
42.0
37.4
HF2C
O
N
O
NH
N
4o
13C NMR 101 MHz, DMSO
f1 (ppm)

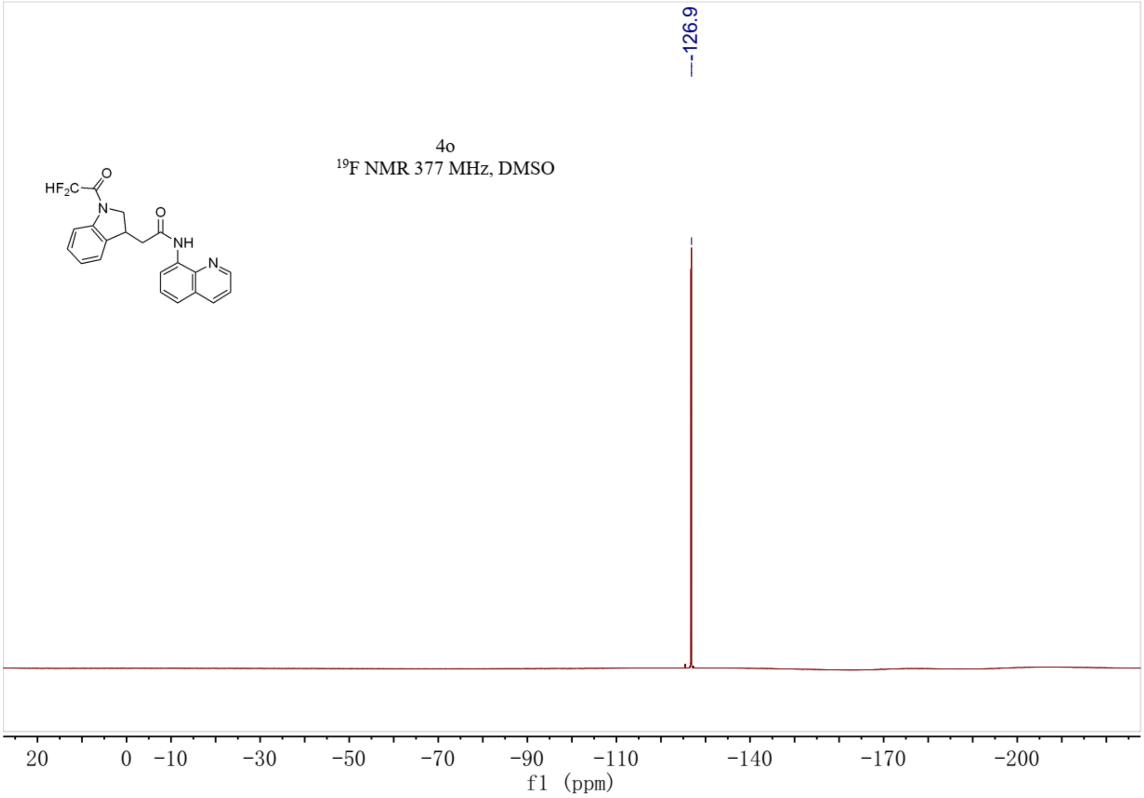
-126.9
4o
19F NMR 377 MHz, DMSO
HF2C
O
N
O
NH
N
20
0
−10
−30
−50
−70
−90
−110
−140
−170
−200
f1 (ppm)

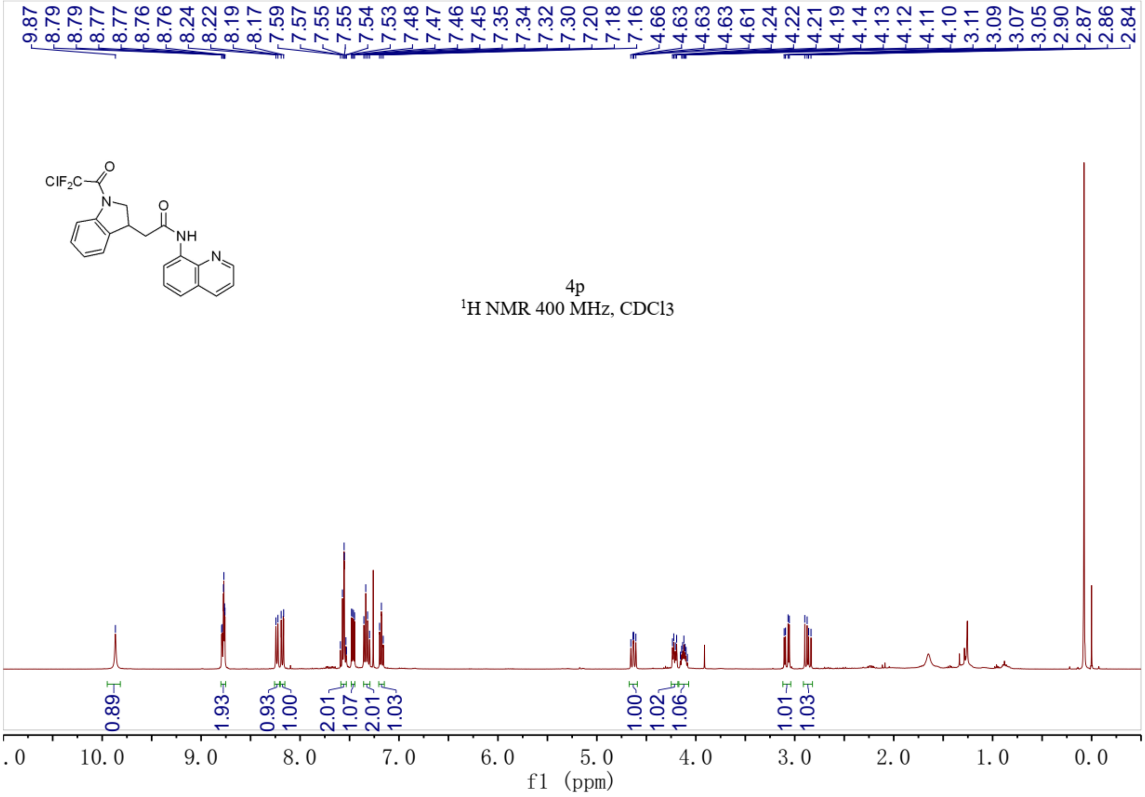
9.87
8.79
8.79
8.77
8.77
8.76
8.76
8.24
8.22
8.19
8.17
7.59
7.57
7.55
7.55
7.54
7.53
7.48
7.47
7.46
7.45
7.35
7.34
7.32
7.30
7.20
7.18
7.16
4.66
4.63
4.63
4.61
4.24
4.22
4.21
4.19
4.14
4.13
4.12
4.11
4.10
3.11
3.09
3.07
3.05
2.90
2.87
2.86
2.84
ClF2C
O
N
O
NH
N
4p
1H NMR 400 MHz, CDCl3
0.89
1.93
0.93
1.00
2.01
1.07
2.01
1.03
1.00
1.02
1.06
1.01
1.03
10.0
9.0
8.0
7.0
6.0
5.0
4.0
3.0
2.0
1.0
0.0
f1 (ppm)

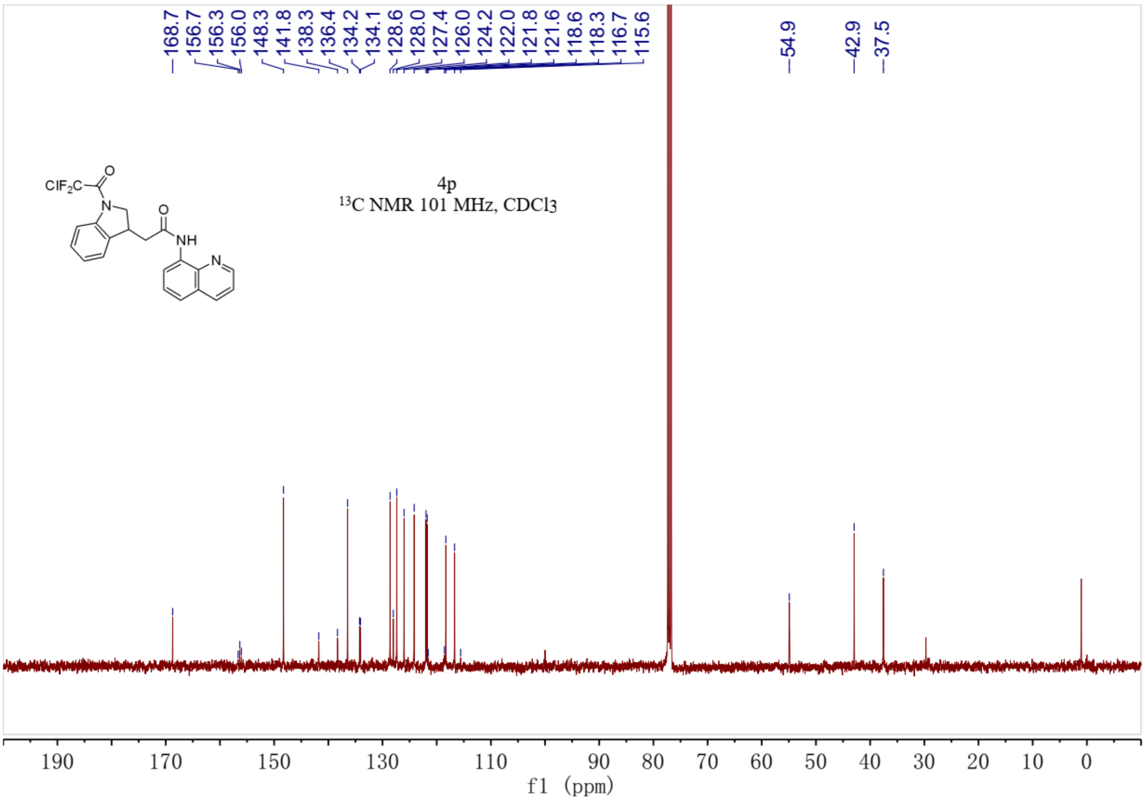
168.7
156.7
156.3
156.0
148.3
141.8
138.3
136.4
134.2
134.1
128.6
128.0
127.4
126.0
124.2
122.0
121.8
121.6
118.6
118.3
116.7
115.6
54.9
42.9
37.5
ClF2C
O
N
O
NH
N
4p
13C NMR 101 MHz, CDCl3
190
170
150
130
110
90
80
70
60
50
40
30
20
10
0
f1 (ppm)

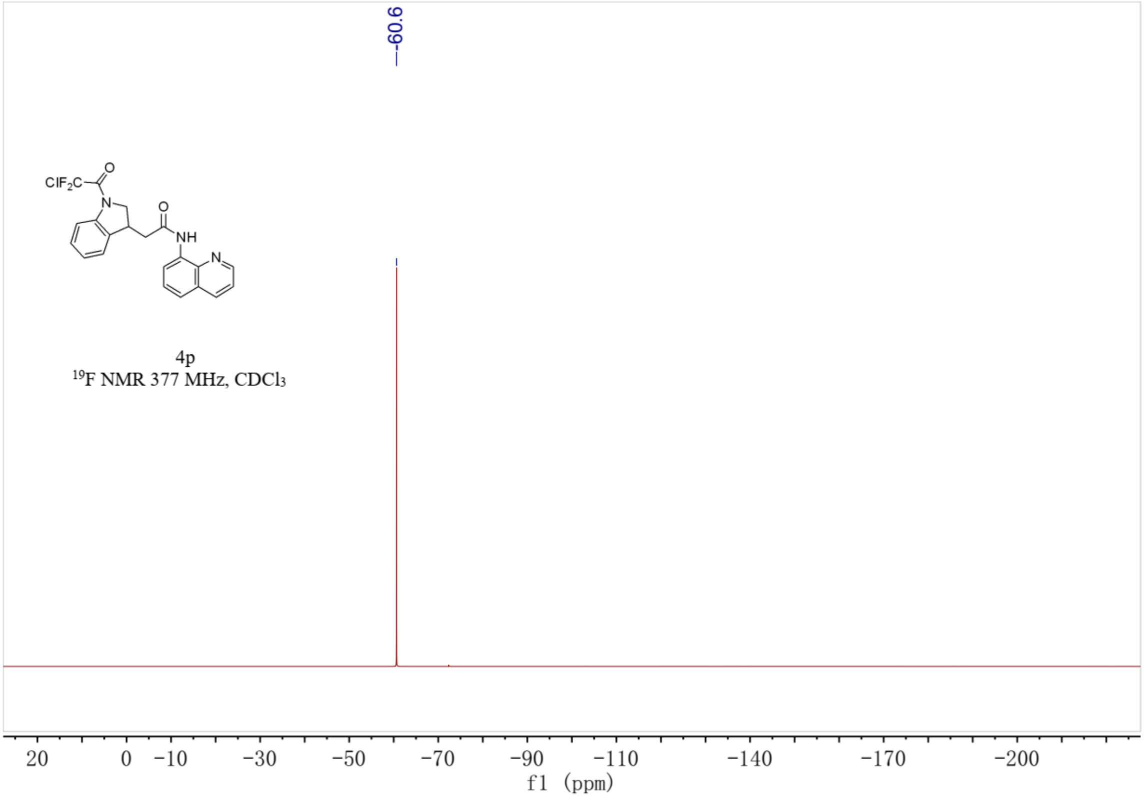

-60.6
ClF2C
O
N
O
NH
N
4p
19F NMR 377 MHz, CDCl3
20
0
−10
−30
−50
−70
−90
−110
−140
−170
−200
f1 (ppm)

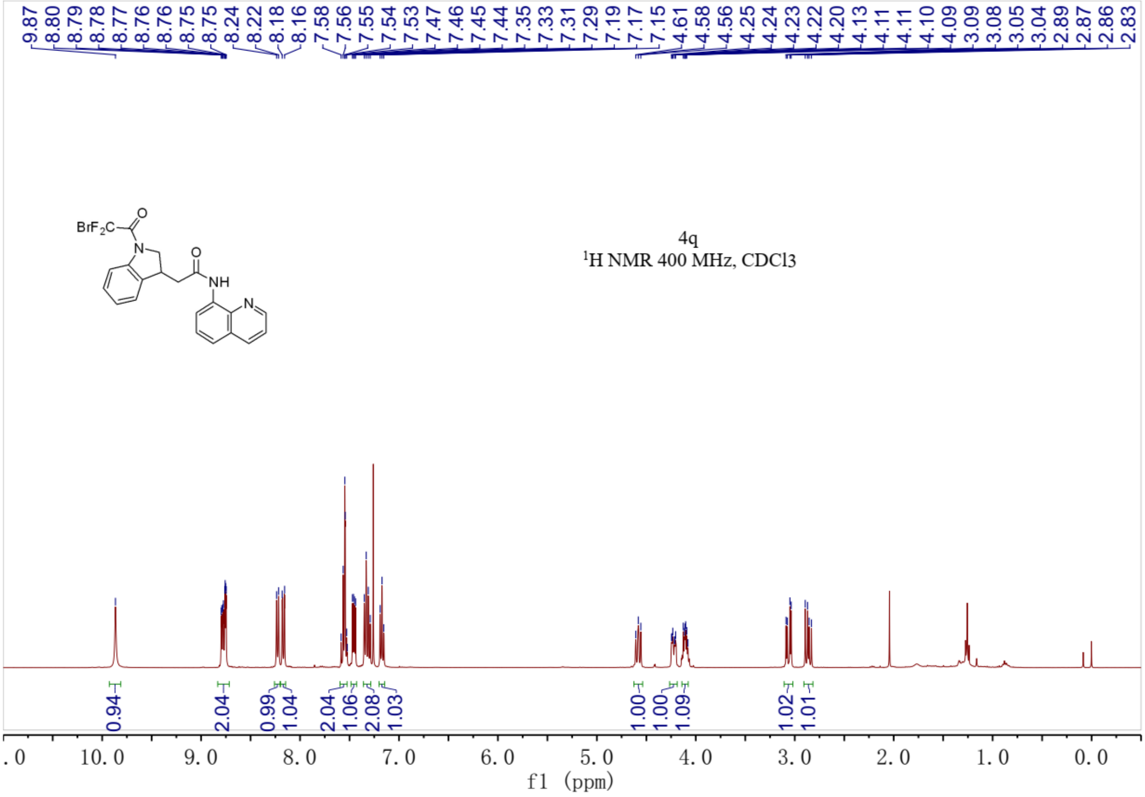
9.87 8.80 8.79 8.78 8.77 8.76 8.76 8.75 8.75 8.24 8.22 8.18 8.16 7.58 7.56 7.55 7.54 7.53 7.47 7.46 7.45 7.44 7.35 7.33 7.31 7.29 7.19 7.17 7.15 4.61 4.58 4.56 4.25 4.24 4.23 4.22 4.20 4.13 4.11 4.11 4.10 4.09 3.09 3.08 3.05 3.04 2.89 2.87 2.86 2.83
BrF2C
O
N
O
NH
N
4q
1H NMR 400 MHz, CDCl3
0.94 2.04 0.99 1.04 2.04 1.06 2.08 1.03 1.00 1.00 1.09 1.02 1.01
.0 10.0 9.0 8.0 7.0 6.0 5.0 4.0 3.0 2.0 1.0 0.0
f1 (ppm)

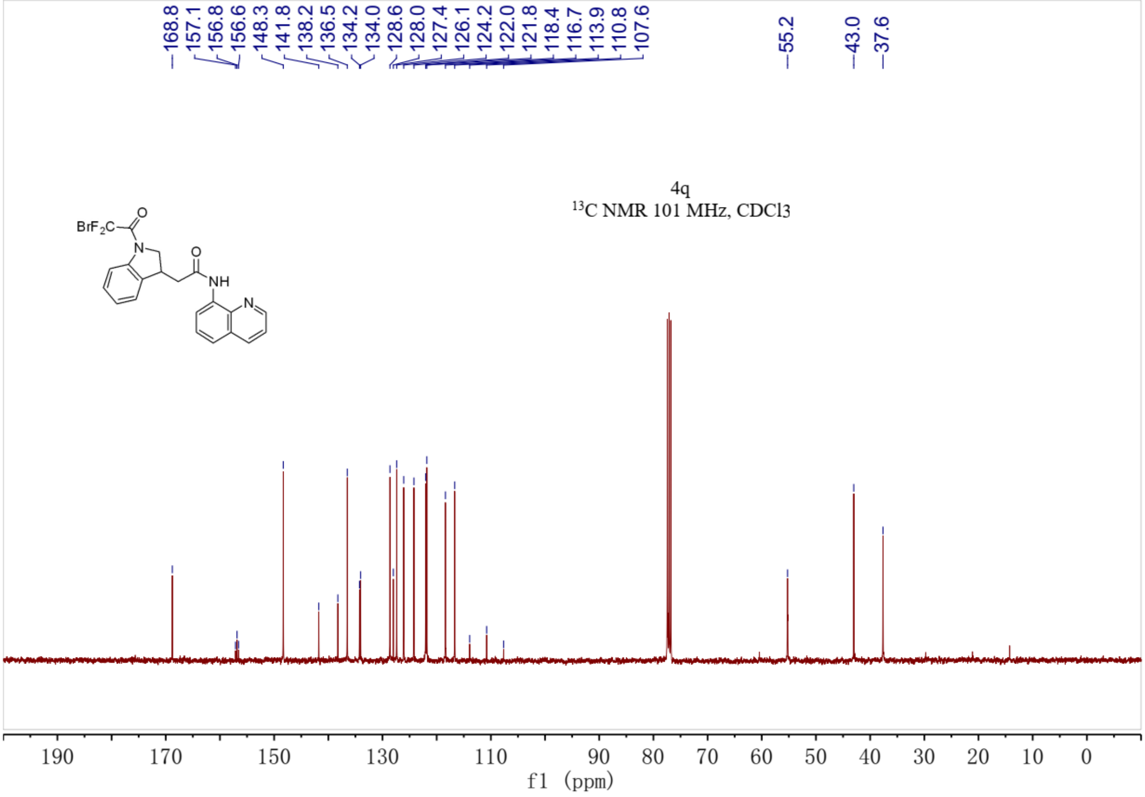
168.8 157.1 156.8 156.6 148.3 141.8 138.2 136.5 134.2 134.0 128.6 128.0 127.4 126.1 124.2 122.0 121.8 118.4 116.7 113.9 110.8 107.6 55.2 43.0 37.6
BrF2C
O
N
O
NH
N
4q
13C NMR 101 MHz, CDCl3
190 170 150 130 110 90 80 70 60 50 40 30 20 10 0
f1 (ppm)

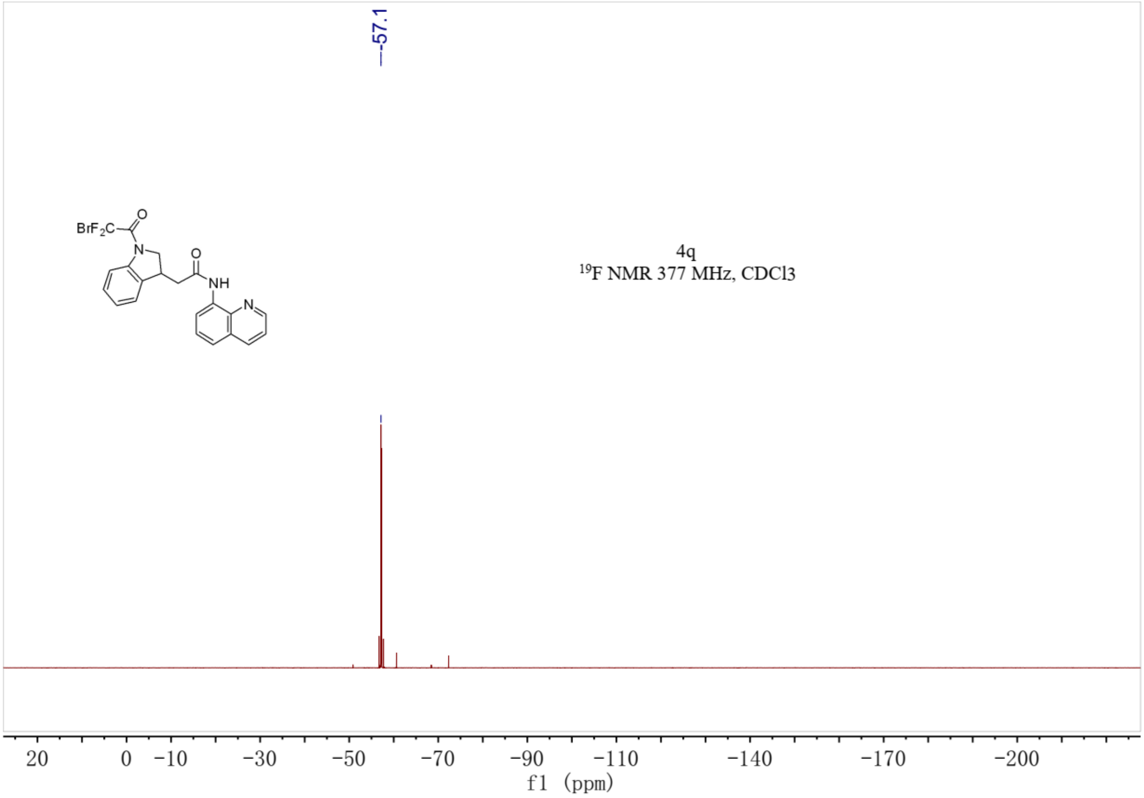

–57.1
BrF2C
O
N
O
NH
N
4q
19F NMR 377 MHz, CDCl3
20
0
−10
−30
−50
−70
−90
−110
−140
−170
−200
f1 (ppm)

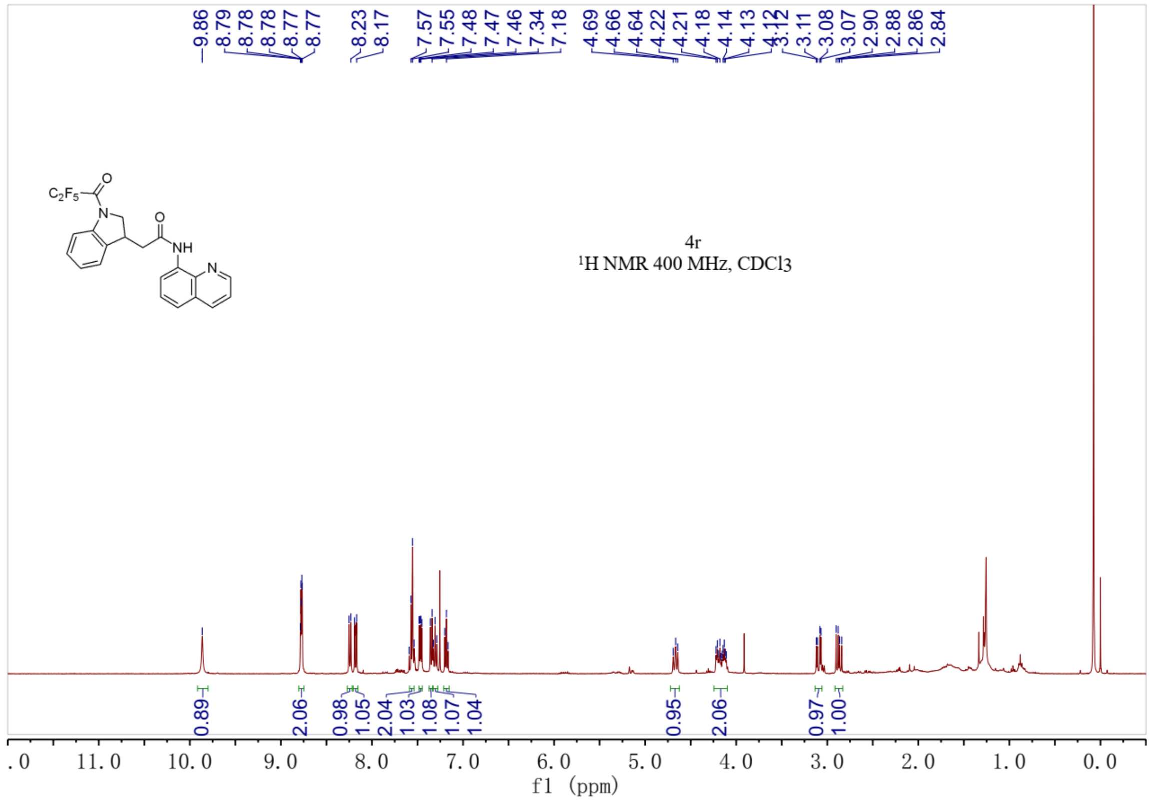

9.86 8.79 8.78 8.78 8.77 8.77 8.23 8.17 7.57 7.55 7.48 7.47 7.46 7.34 7.18 4.69 4.66 4.64 4.22 4.21 4.18 4.14 4.13 4.12 3.12 3.11 3.08 3.07 2.90 2.88 2.86 2.84
4r
¹H NMR 400 MHz, CDCl3
0.89 2.06 0.98 1.05 2.04 1.03 1.08 1.07 1.04 0.95 2.06 0.97 1.00
f1 (ppm)

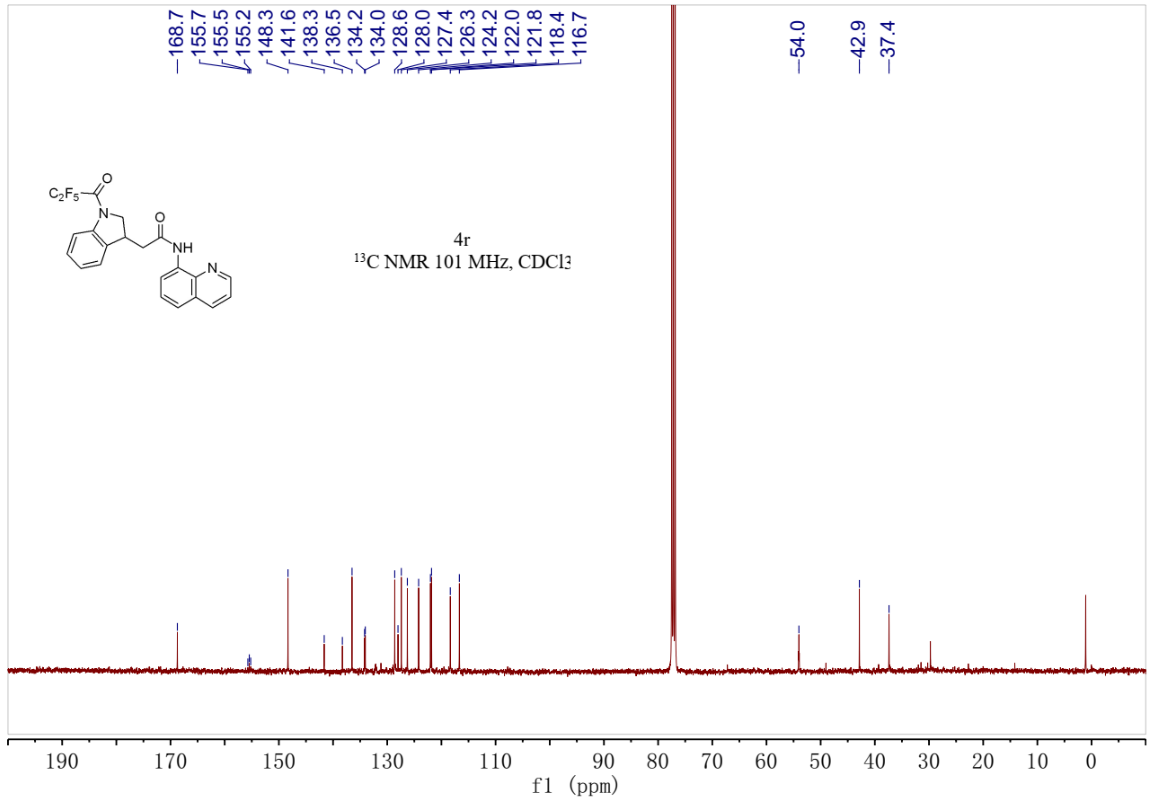

168.7 155.7 155.5 155.2 148.3 141.6 138.3 136.5 134.2 134.0 128.6 128.0 127.4 126.3 124.2 122.0 121.8 118.4 116.7 54.0 42.9 37.4
4r
¹³C NMR 101 MHz, CDCl3
f1 (ppm)

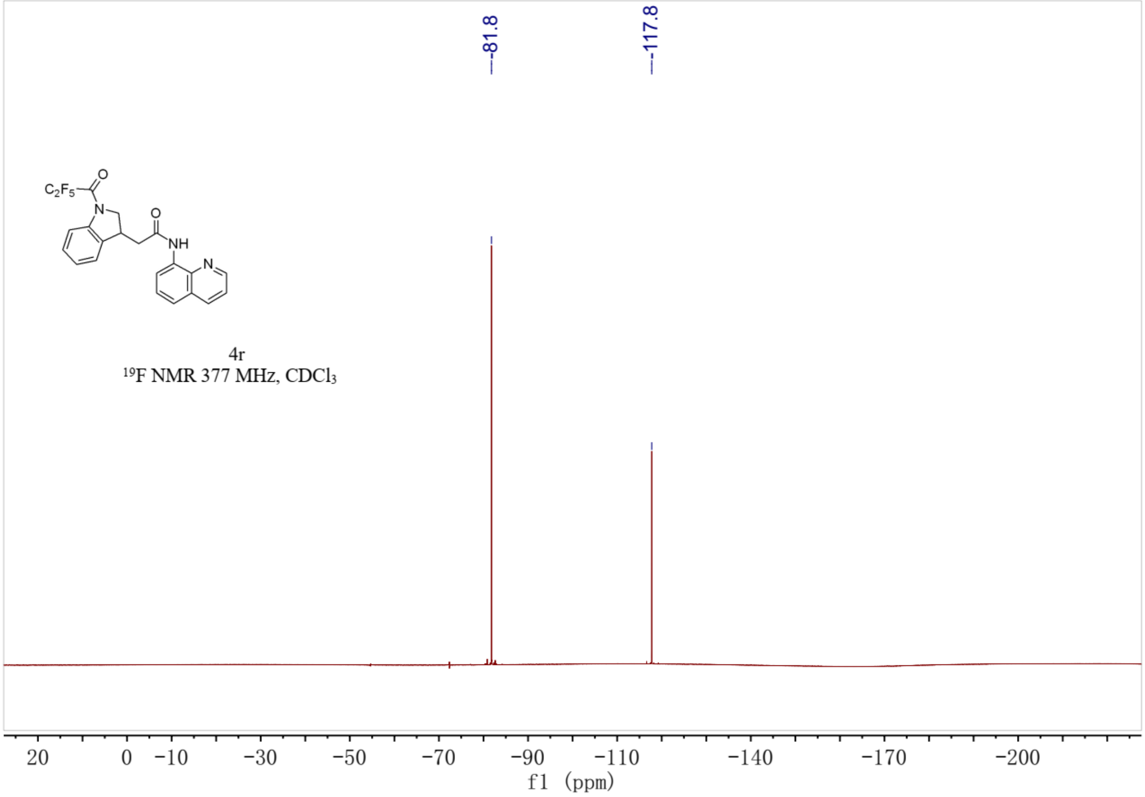
-81.8
-117.8
C2F5
O
N
O
NH
N
4r
19F NMR 377 MHz, CDCl3
20
0
−10
−30
−50
−70
−90
−110
−140
−170
−200
f1 (ppm)

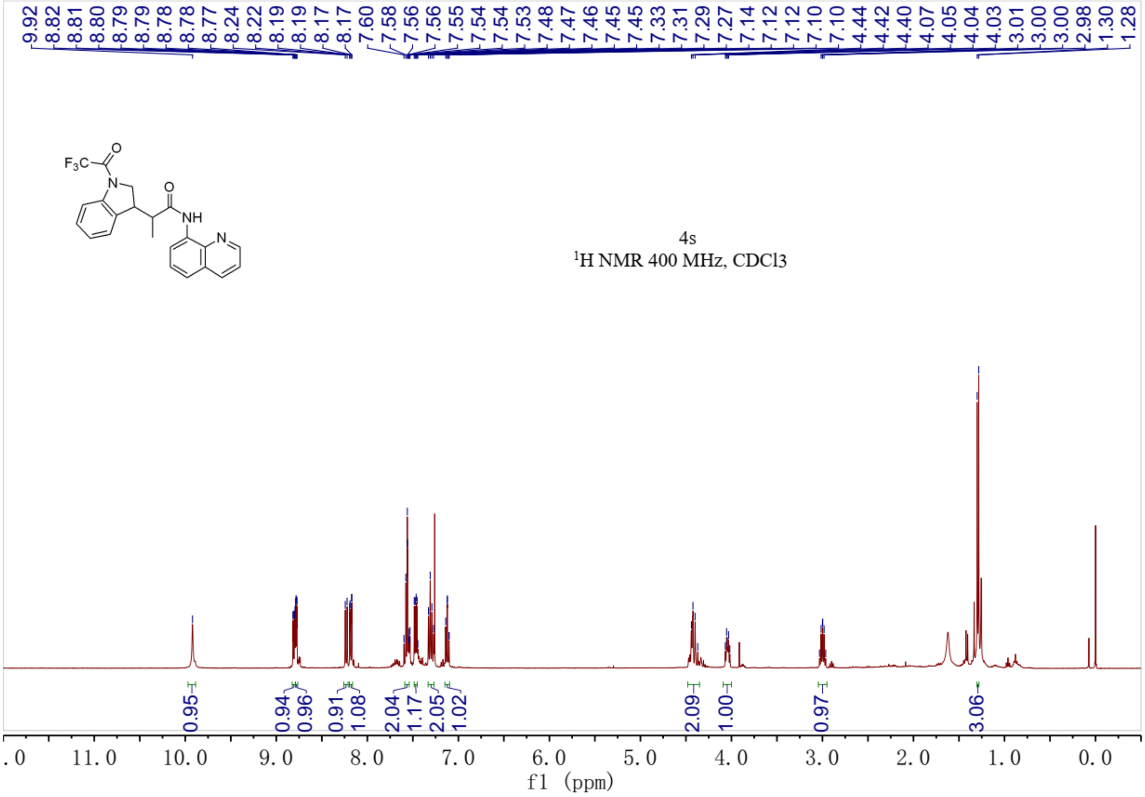

9.92
8.82
8.81
8.80
8.79
8.79
8.78
8.78
8.77
8.24
8.22
8.19
8.19
8.17
8.17
7.60
7.58
7.56
7.55
7.54
7.54
7.53
7.48
7.47
7.46
7.45
7.45
7.33
7.31
7.29
7.27
7.14
7.12
7.12
7.10
7.10
4.44
4.42
4.40
4.07
4.05
4.04
4.03
3.01
3.00
3.00
2.98
1.30
1.28
F_3C
O
N
O
NH
N
4s
^{1}H NMR 400 MHz, CDCl3
0.95
0.94
0.96
0.91
1.08
2.04
1.17
2.05
1.02
2.09
1.00
0.97
3.06
11.0
10.0
9.0
8.0
7.0
6.0
5.0
4.0
3.0
2.0
1.0
0.0
f1 (ppm)

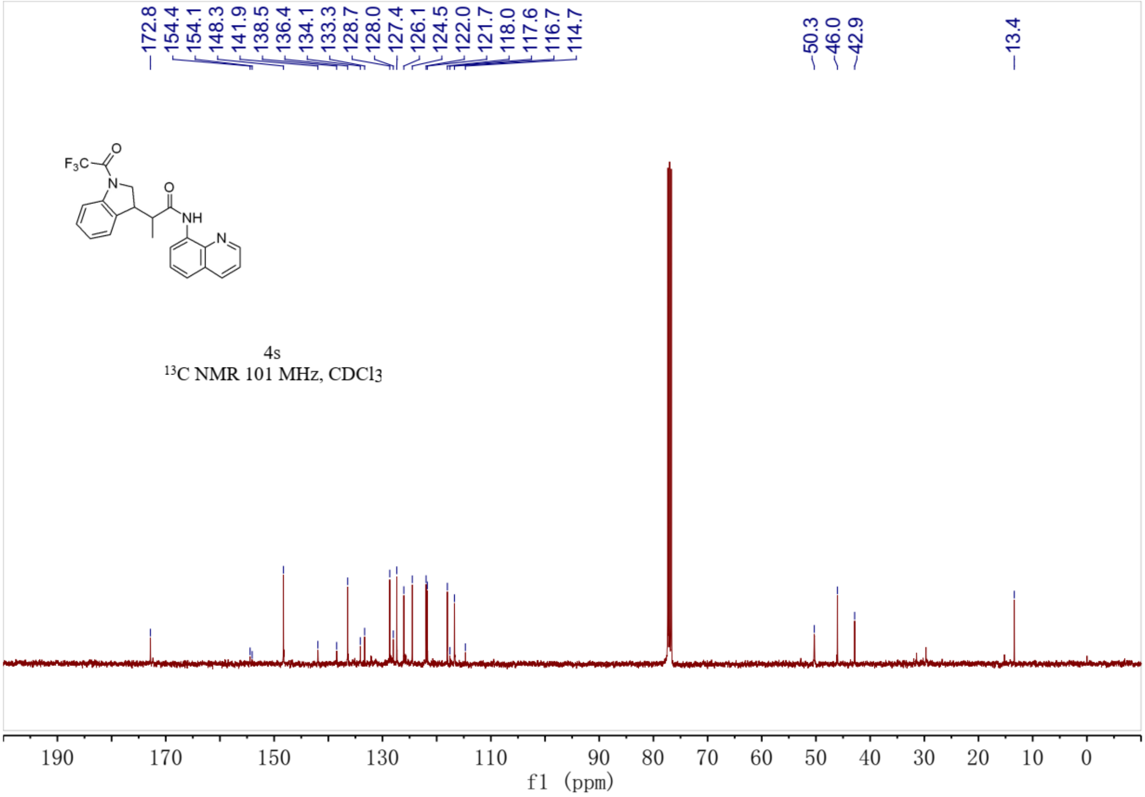

172.8
154.4
154.1
148.3
141.9
138.5
136.4
134.1
133.3
128.7
128.0
127.4
126.1
124.5
122.0
121.7
118.0
117.6
116.7
114.7
50.3
46.0
42.9
13.4
F_3C
O
N
O
NH
N
4s
^{13}C NMR 101 MHz, CDCl3
190
170
150
130
110
90
80
70
60
50
40
30
20
10
0
f1 (ppm)

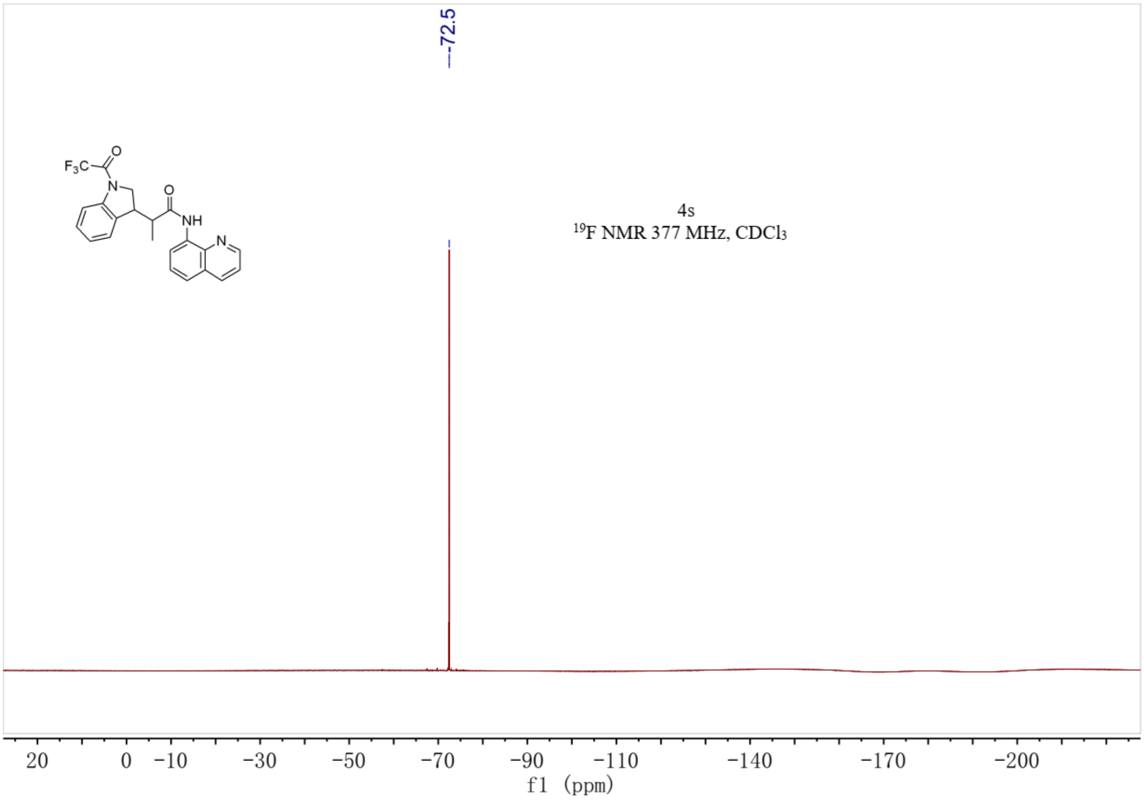
-72.5
4s
19F NMR 377 MHz, CDCl3
F3C
O
N
O
NH
N
20
0
−10
−30
−50
−70
−90
−110
−140
−170
−200
f1 (ppm)

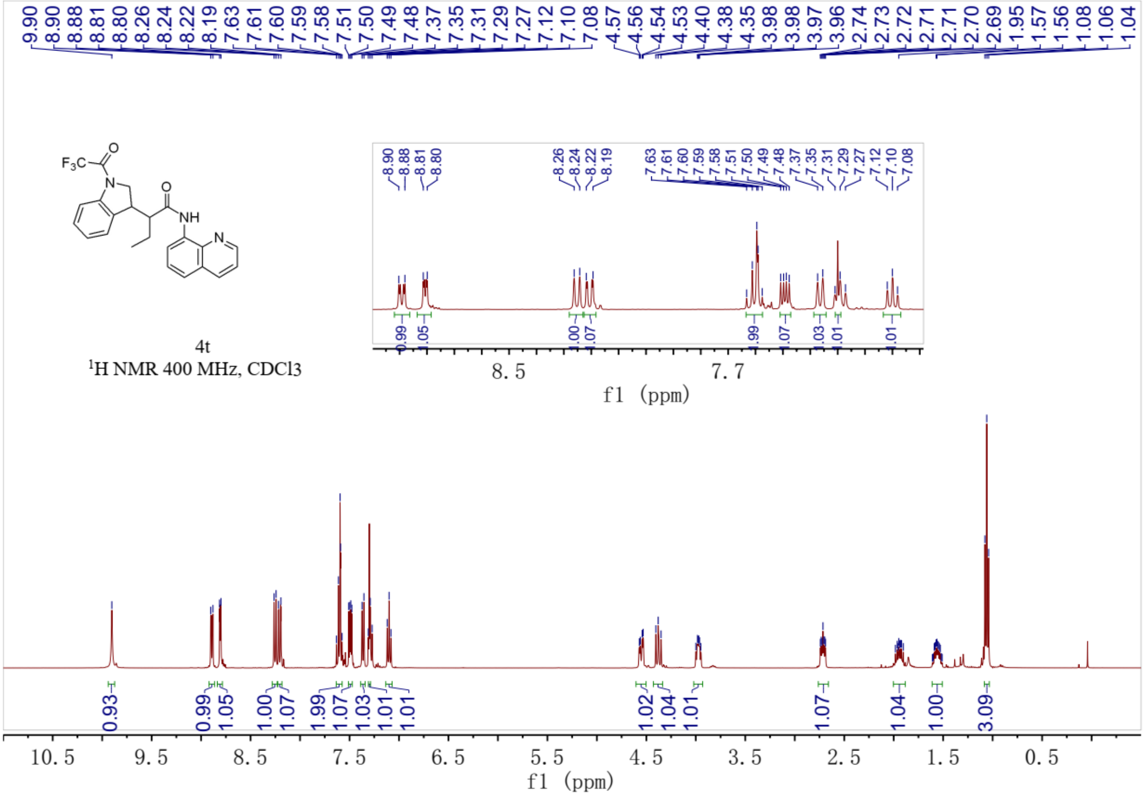
9.90 8.90 8.88 8.81 8.80 8.26 8.24 8.22 8.19 7.63 7.61 7.60 7.59 7.58 7.51 7.50 7.49 7.48 7.37 7.35 7.31 7.29 7.27 7.12 7.10 7.08 4.57 4.56 4.54 4.53 4.40 4.38 4.35 3.98 3.98 3.97 3.96 2.74 2.73 2.72 2.71 2.71 2.70 2.69 1.95 1.57 1.56 1.08 1.06 1.04
F_3C
O
N
NH
4t
^{1}H NMR 400 MHz, CDCl3
8.5
7.7
f1 (ppm)
0.93 0.99 1.05 1.00 1.07 1.99 1.07 1.03 1.01 1.01 1.02 1.04 1.01 1.07 1.04 1.00 3.09
10.5 9.5 8.5 7.5 6.5 5.5 4.5 3.5 2.5 1.5 0.5
f1 (ppm)

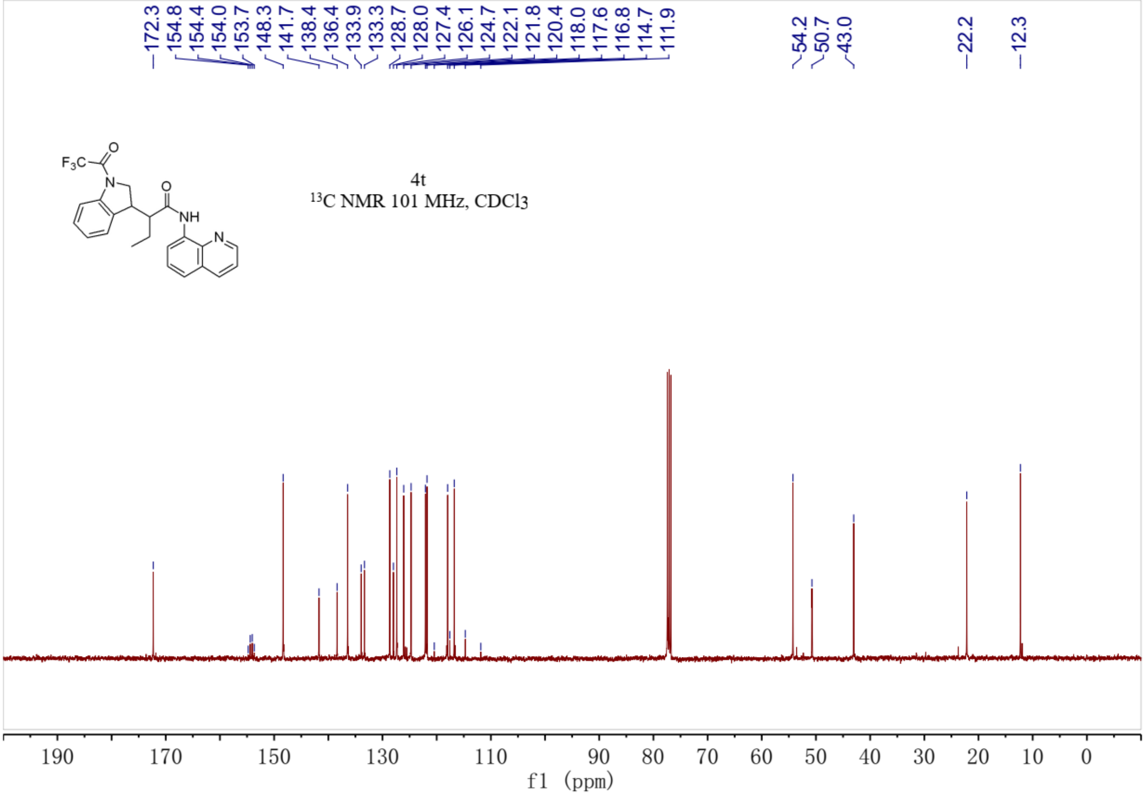
172.3 154.8 154.4 154.0 153.7 148.3 141.7 138.4 136.4 133.9 133.3 128.7 128.0 127.4 126.1 124.7 122.1 121.8 120.4 118.0 117.6 116.8 114.7 111.9
54.2 50.7 43.0
22.2
12.3
F_3C
O
N
NH
4t
^{13}C NMR 101 MHz, CDCl3
190 170 150 130 110 90 80 70 60 50 40 30 20 10 0
f1 (ppm)

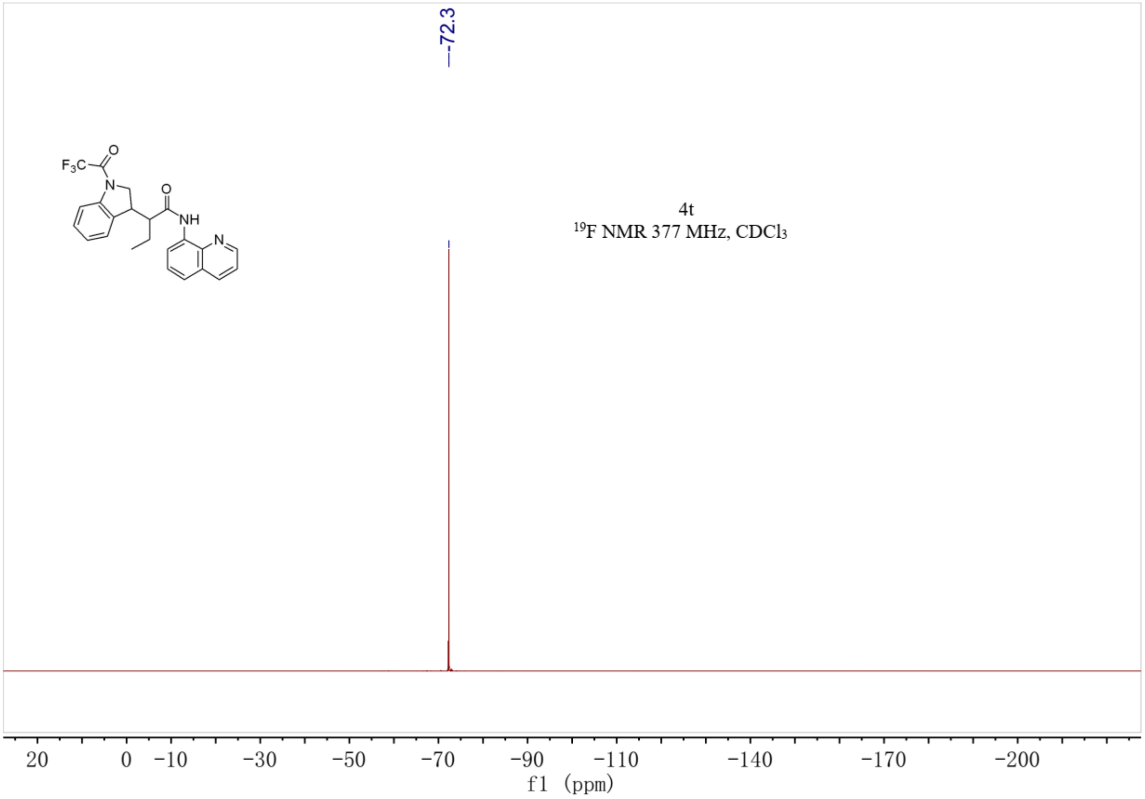
-72.3
4t
19F NMR 377 MHz, CDCl3
20
0
-10
-30
-50
-70
-90
-110
-140
-170
-200
f1 (ppm)

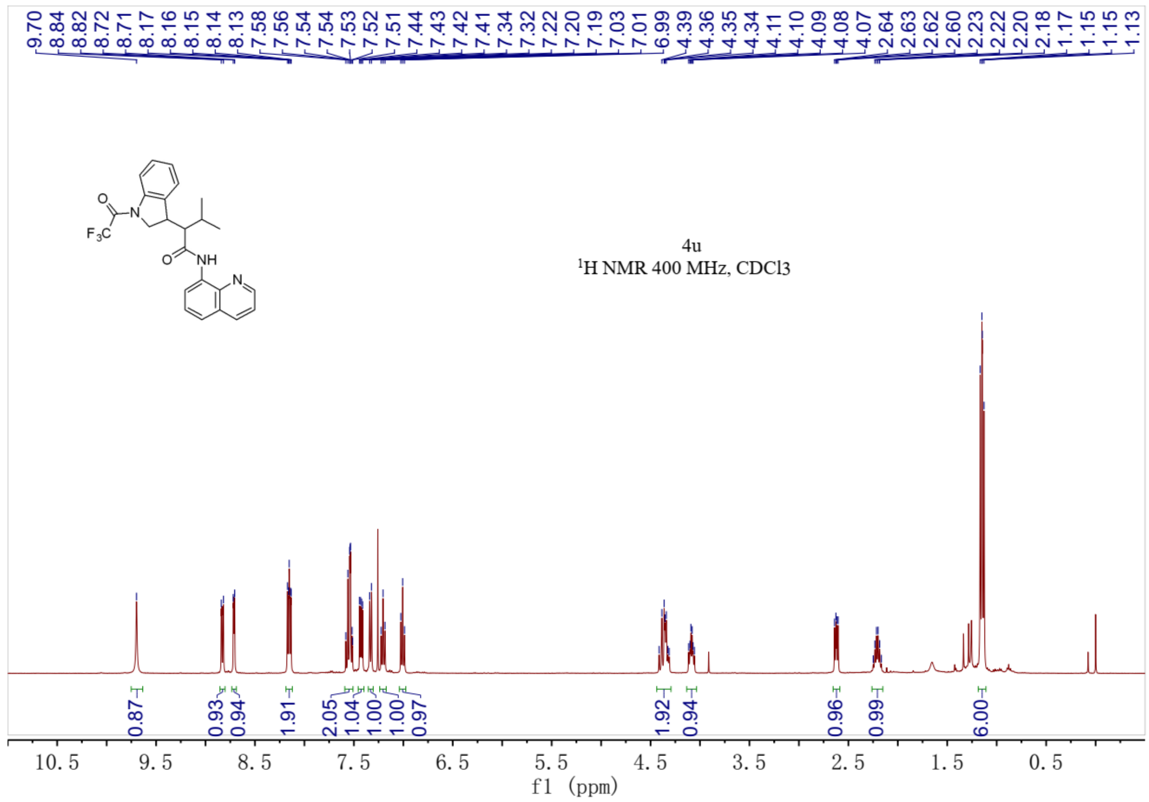
4u
^{1}H NMR 400 MHz, CDCl3
f1 (ppm)

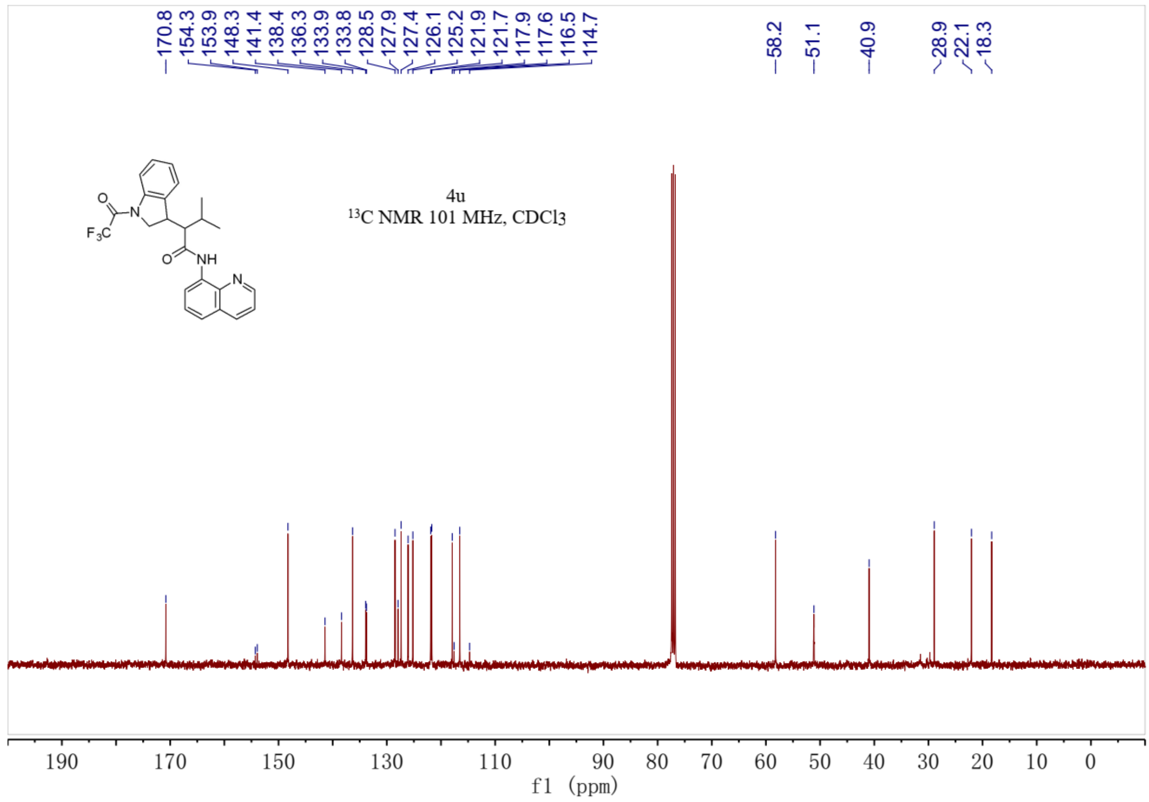
4u
^{13}C NMR 101 MHz, CDCl3
f1 (ppm)

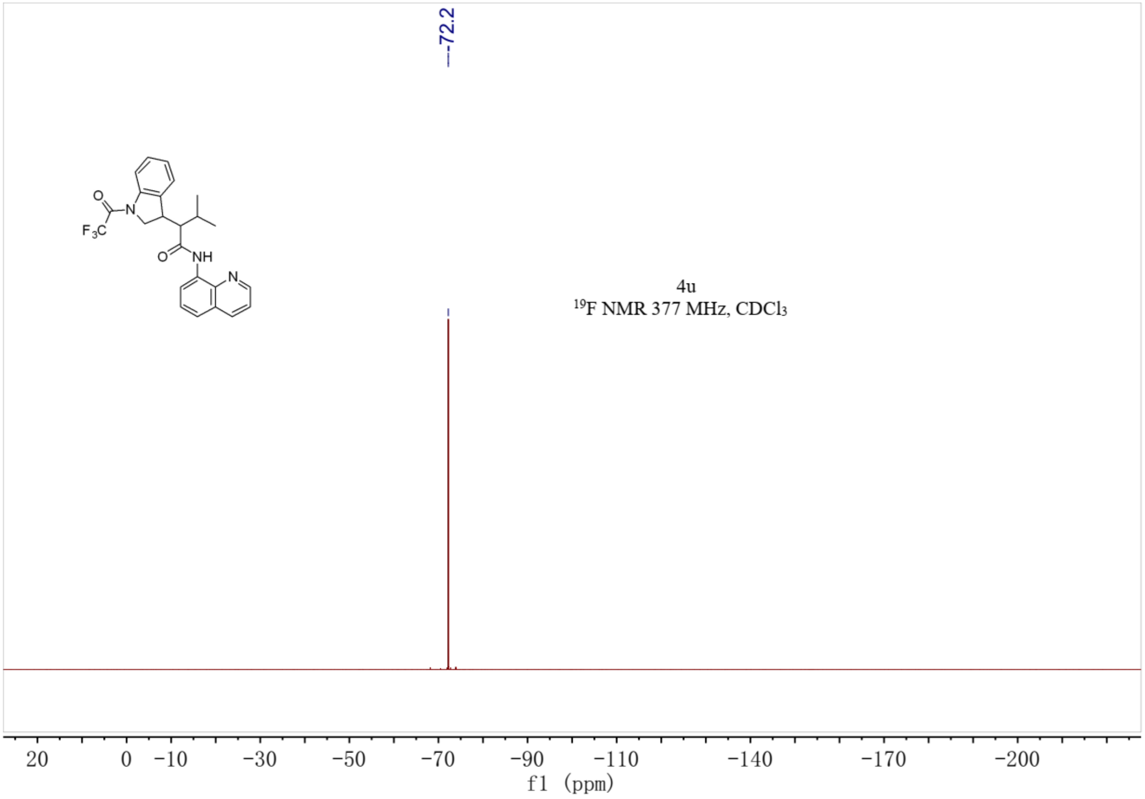

-72.2
F3C
O
N
O
NH
N
4u
19F NMR 377 MHz, CDCl3
20
0
−10
−30
−50
−70
−90
−110
−140
−170
−200
f1 (ppm)

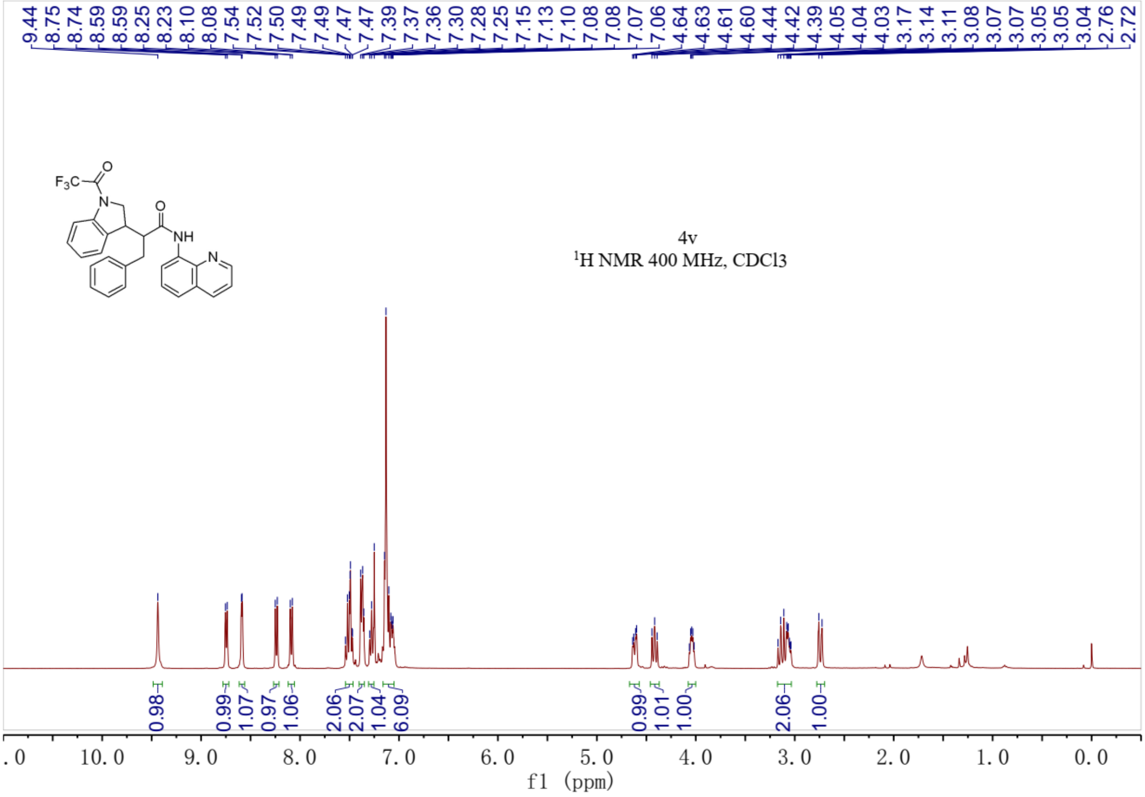
9.44
8.75
8.74
8.59
8.59
8.25
8.23
8.10
8.08
7.54
7.52
7.50
7.49
7.47
7.47
7.39
7.37
7.36
7.30
7.28
7.25
7.15
7.13
7.10
7.08
7.08
7.07
7.06
4.64
4.63
4.61
4.60
4.44
4.42
4.39
4.05
4.04
4.03
3.17
3.14
3.11
3.08
3.07
3.07
3.05
3.05
3.04
2.76
2.72
F3C
O
N
O
NH
N
4v
1H NMR 400 MHz, CDCl3
0.98
0.99
1.07
0.97
1.06
2.06
2.07
1.04
6.09
0.99
1.01
1.00
2.06
1.00
10.0
9.0
8.0
7.0
6.0
5.0
4.0
3.0
2.0
1.0
0.0
f1 (ppm)

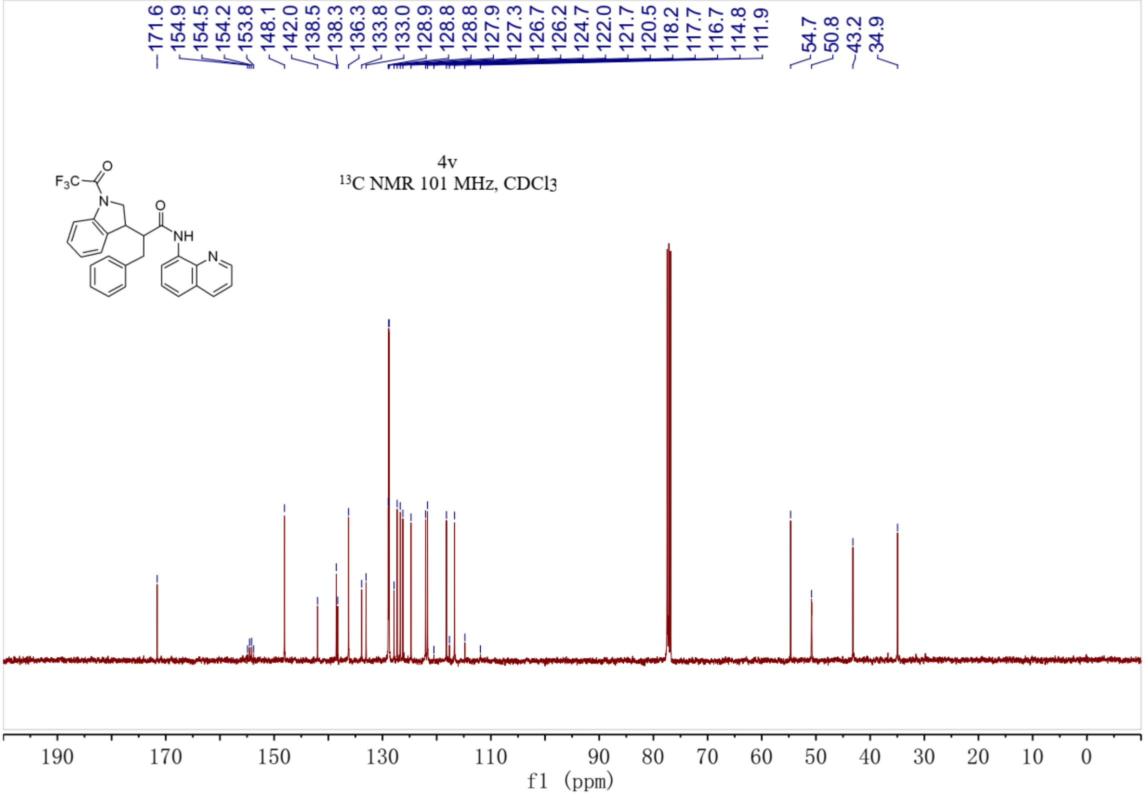
171.6
154.9
154.5
154.2
153.8
148.1
142.0
138.5
138.3
136.3
133.8
133.0
128.9
128.8
128.8
127.9
127.3
126.7
126.2
124.7
122.0
121.7
120.5
118.2
117.7
116.7
114.8
111.9
54.7
50.8
43.2
34.9
F3C
O
N
O
NH
N
4v
13C NMR 101 MHz, CDCl3
190
170
150
130
110
90
80
70
60
50
40
30
20
10
0
f1 (ppm)

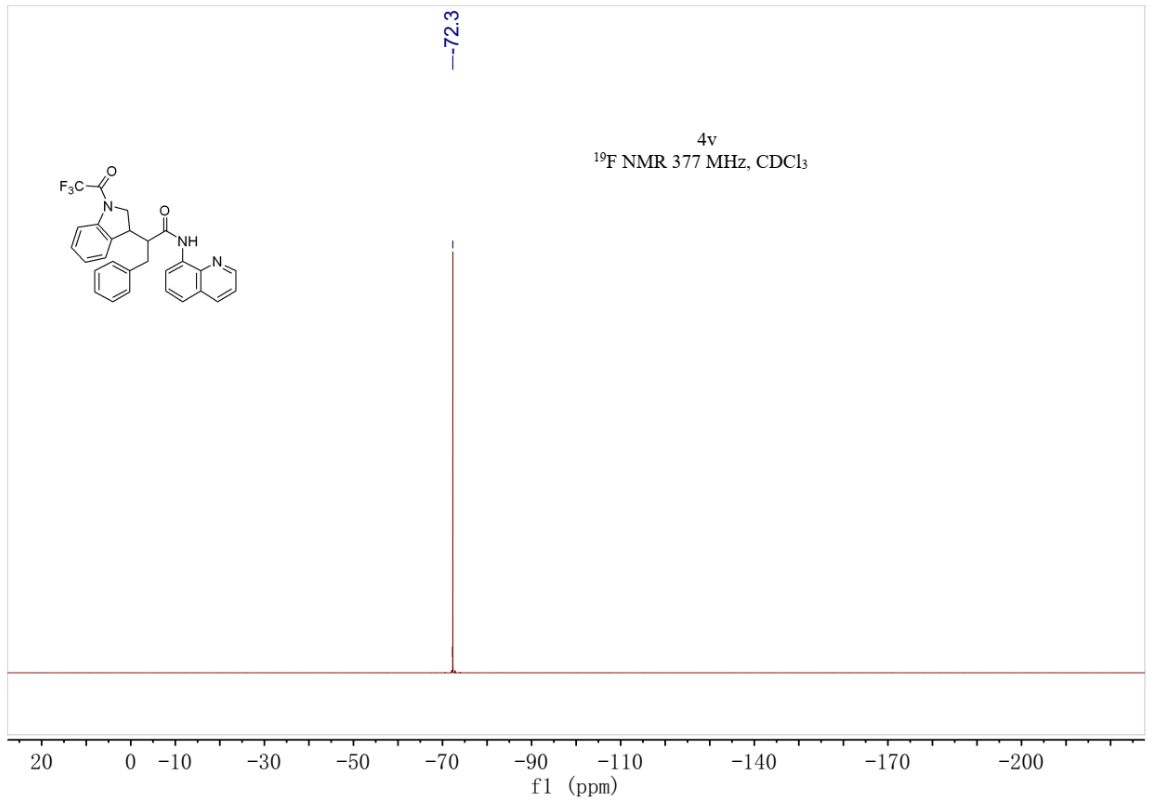

-72.3
4v
19F NMR 377 MHz, CDCl3
F3C
O
N
O
NH
N
20
0
-10
-30
-50
-70
-90
-110
-140
-170
-200
f1 (ppm)

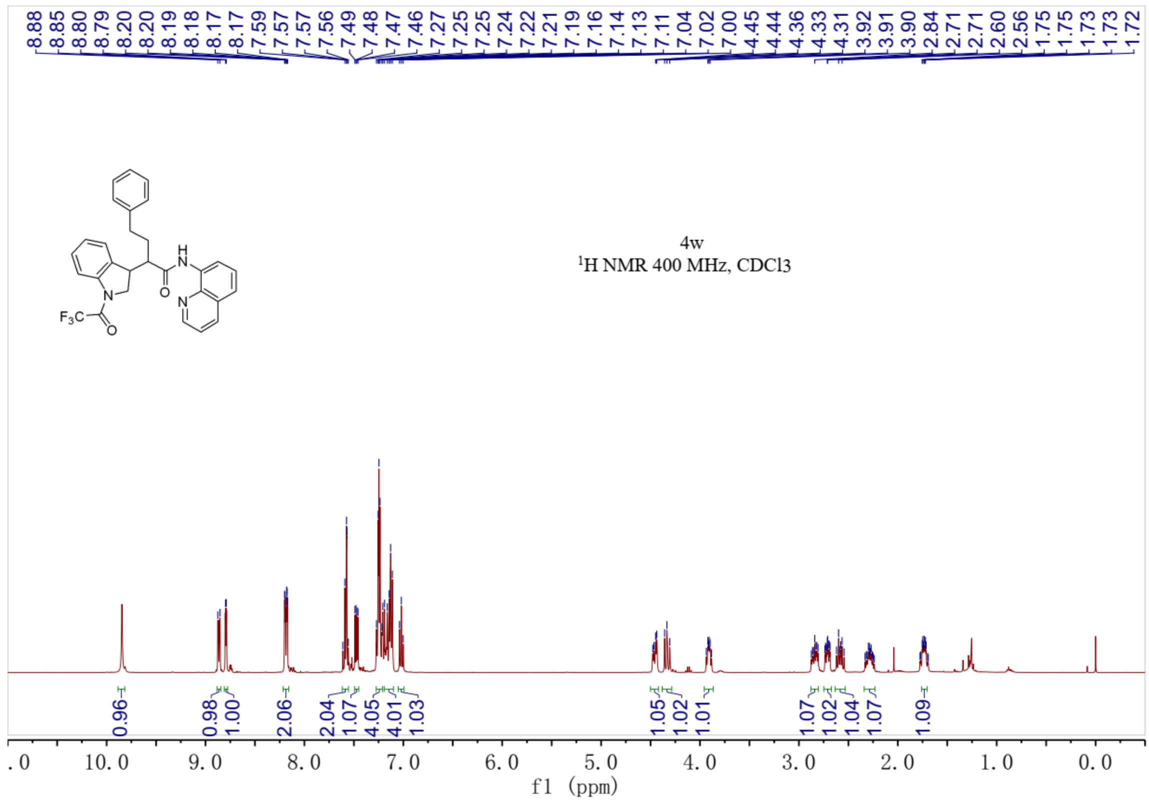
4w
1H NMR 400 MHz, CDCl3
f1 (ppm)

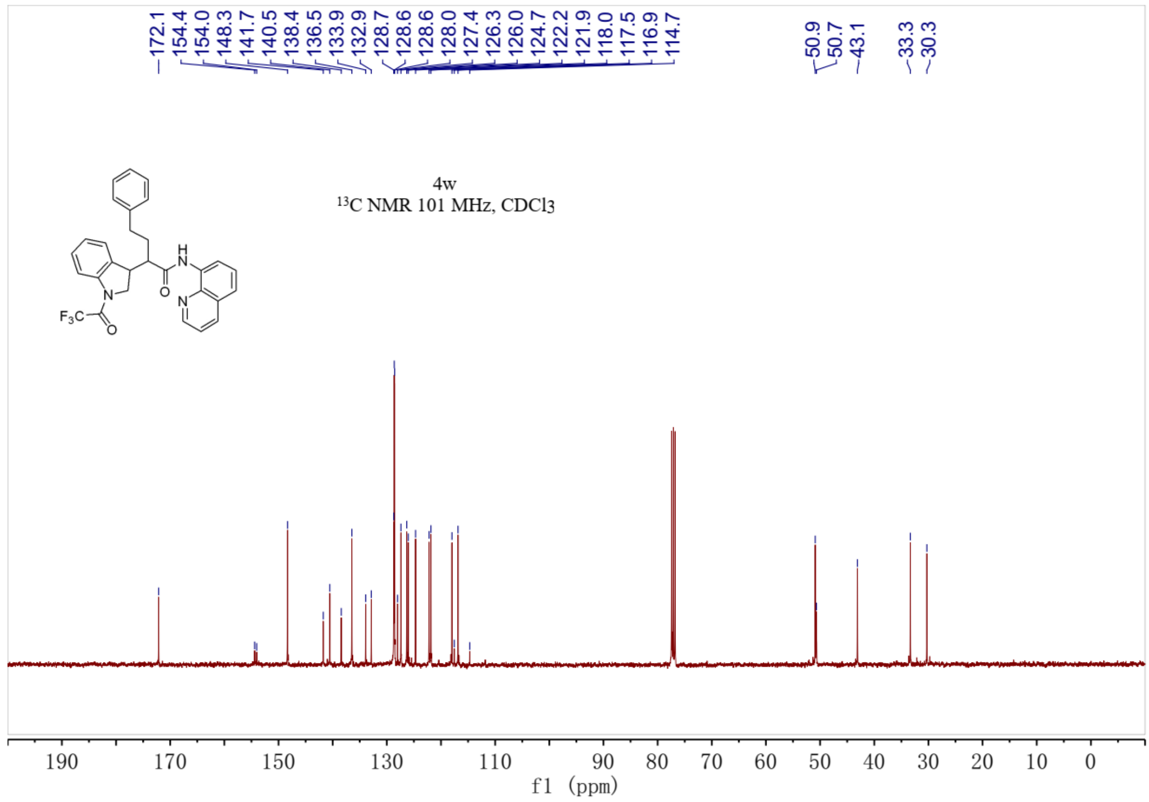
4w
13C NMR 101 MHz, CDCl3
f1 (ppm)

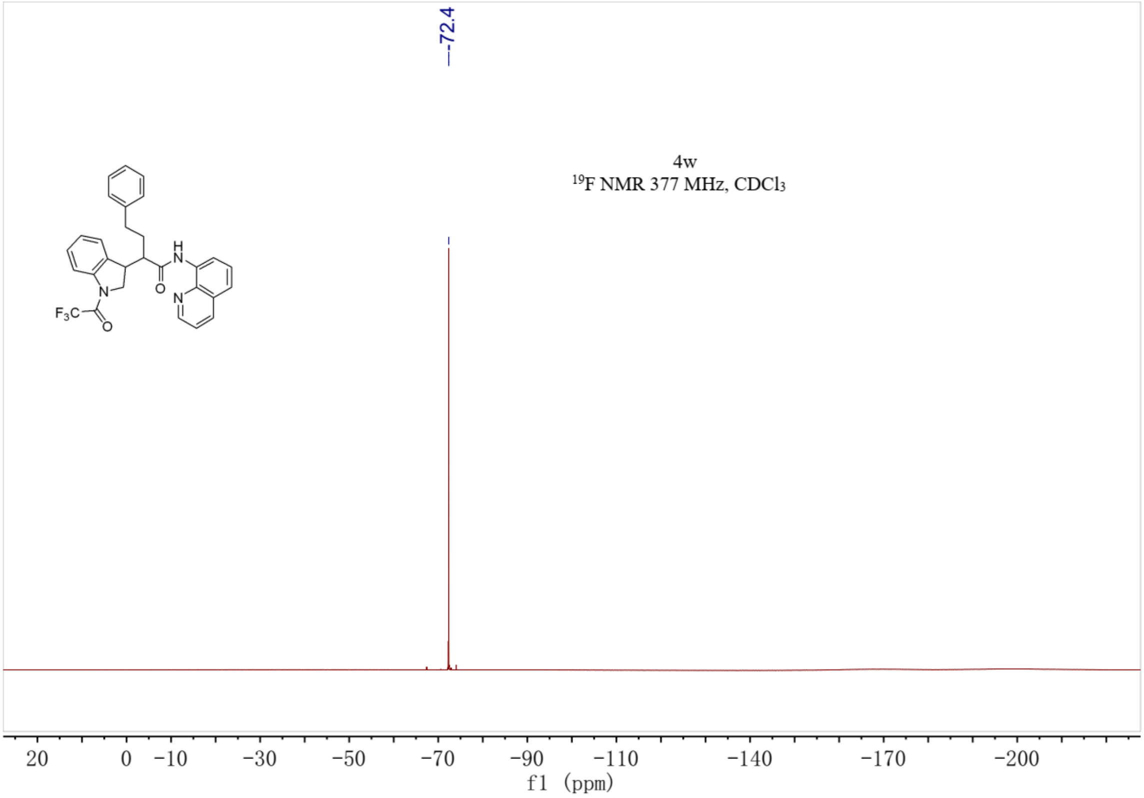
-72.4
4w
19F NMR 377 MHz, CDCl3
F3C
N
O
H
N
N
O
20
0
−10
−30
−50
−70
−90
−110
−140
−170
−200
f1 (ppm)

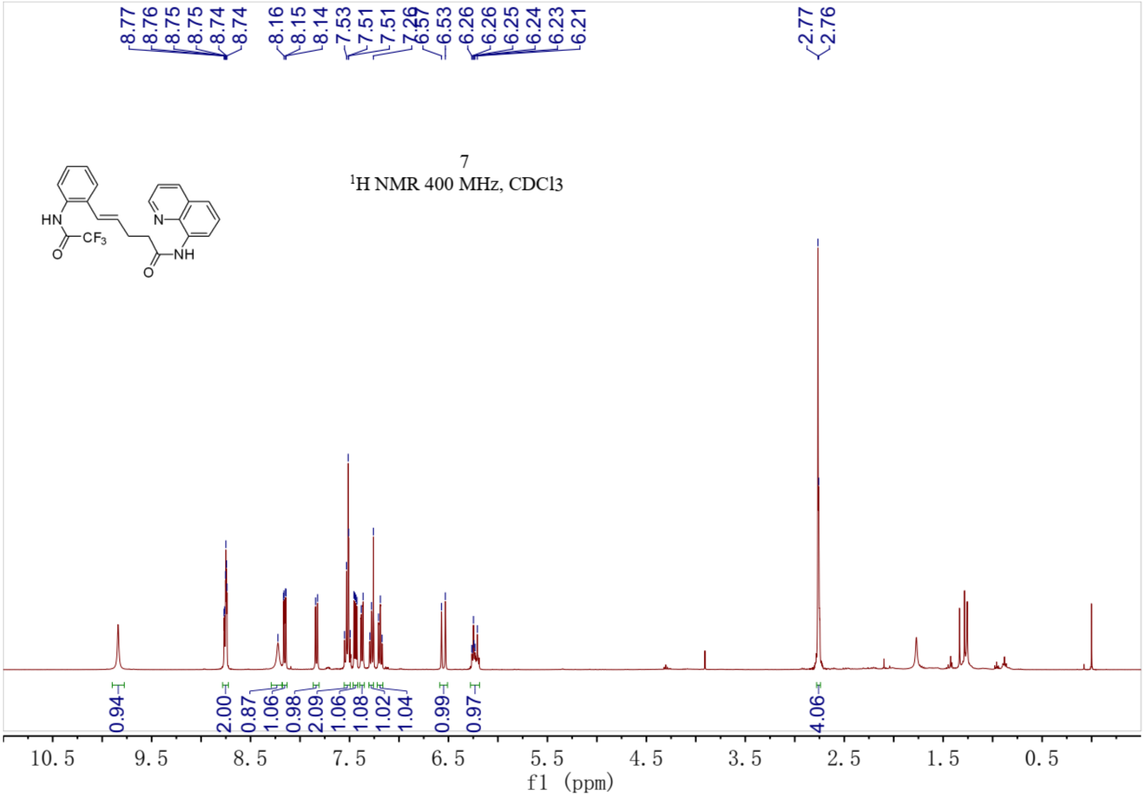
7
^{1}H NMR 400 MHz, CDCl3
f1 (ppm)

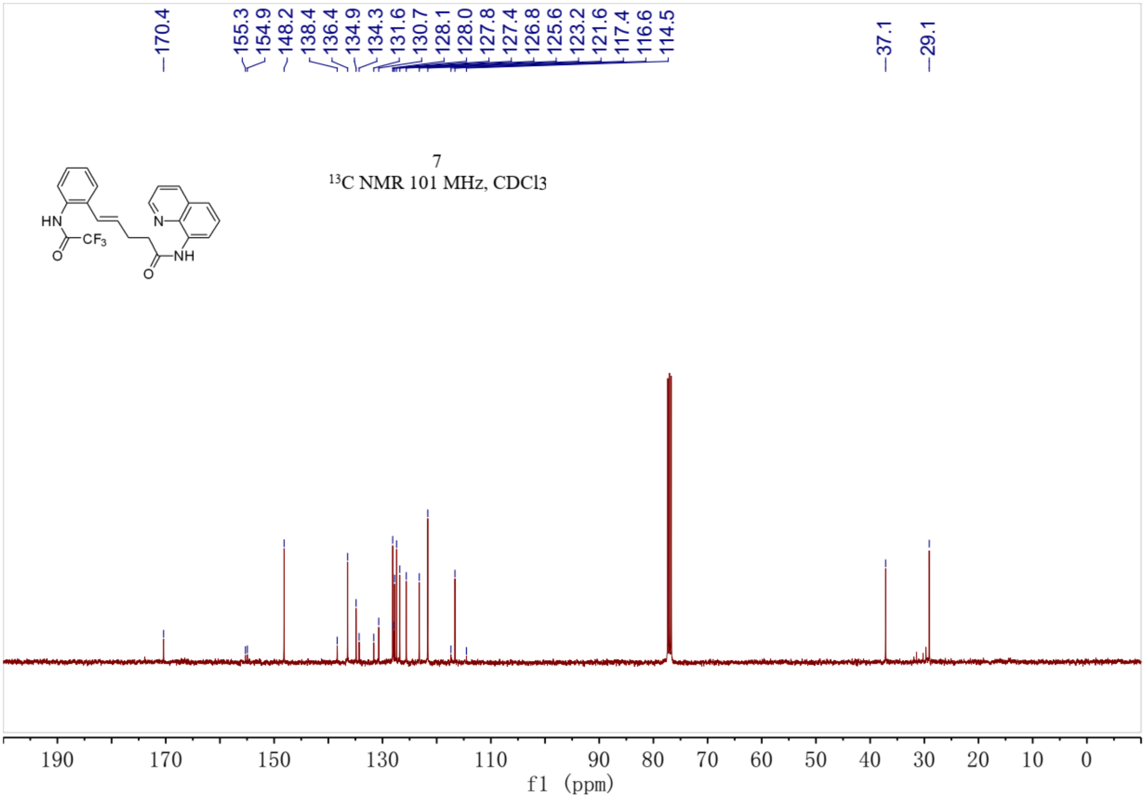
7
^{13}C NMR 101 MHz, CDCl3
f1 (ppm)

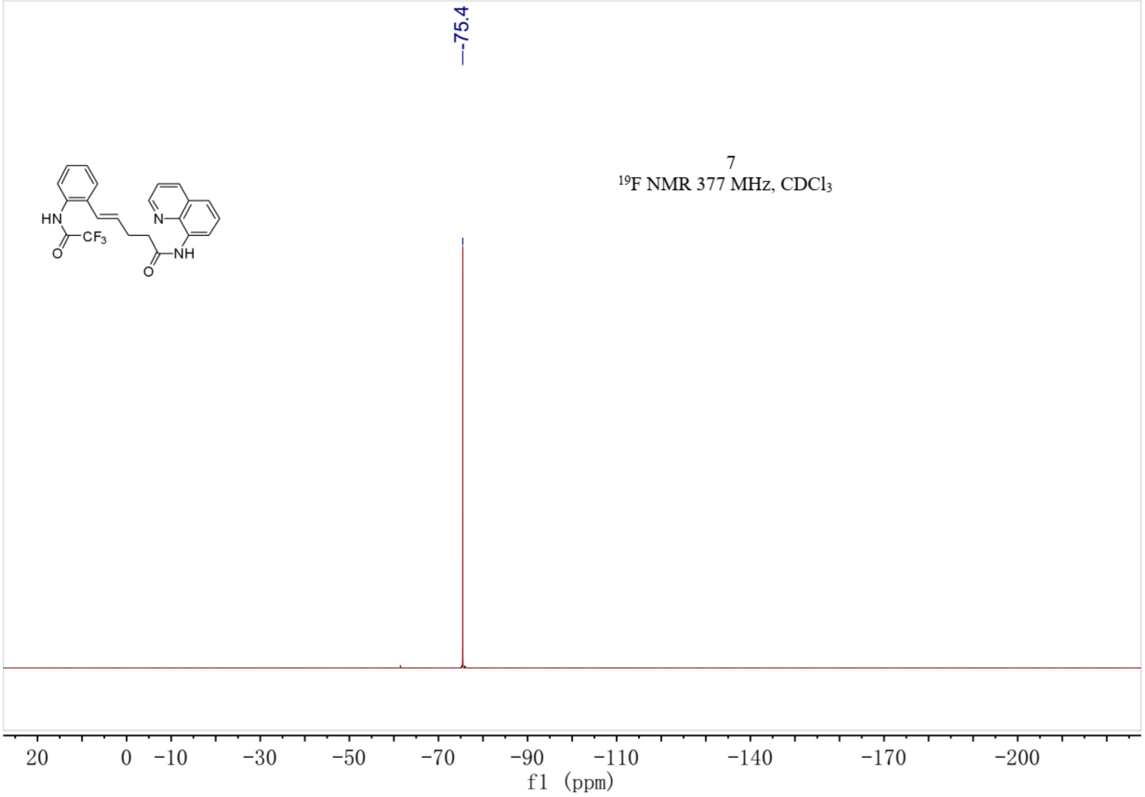
-75.4
7
19F NMR 377 MHz, CDCl3
HN
CF3
O
N
NH
O
20
0
−10
−30
−50
−70
−90
−110
−140
−170
−200
f1 (ppm)

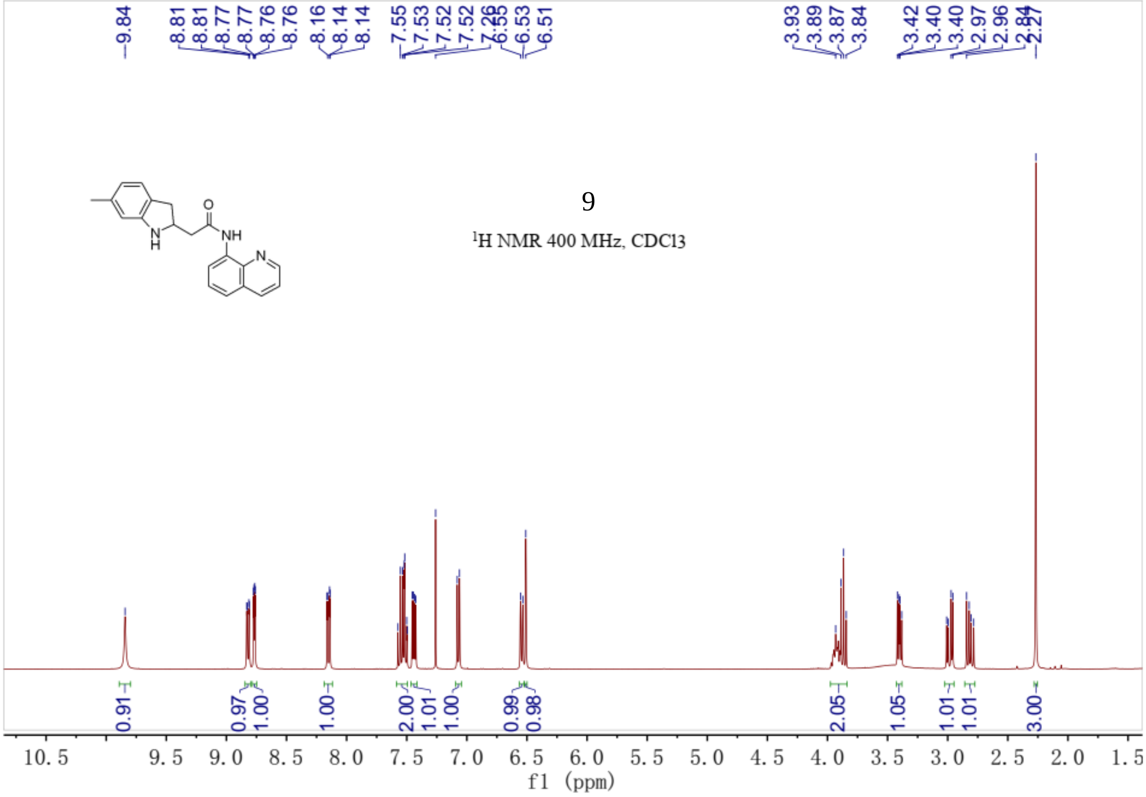
9
1H NMR 400 MHz, CDCl3
9.84
8.81 8.81 8.77 8.77 8.76 8.76
8.16 8.14 8.14
7.55 7.53 7.52 7.52
6.53 6.51
3.93 3.89 3.87 3.84
3.42 3.40 3.40 2.97 2.96
2.27
0.91 0.97 1.00 1.00 2.00 1.01 1.00 0.99 0.98 2.05 1.05 1.01 1.01 3.00
f1 (ppm)

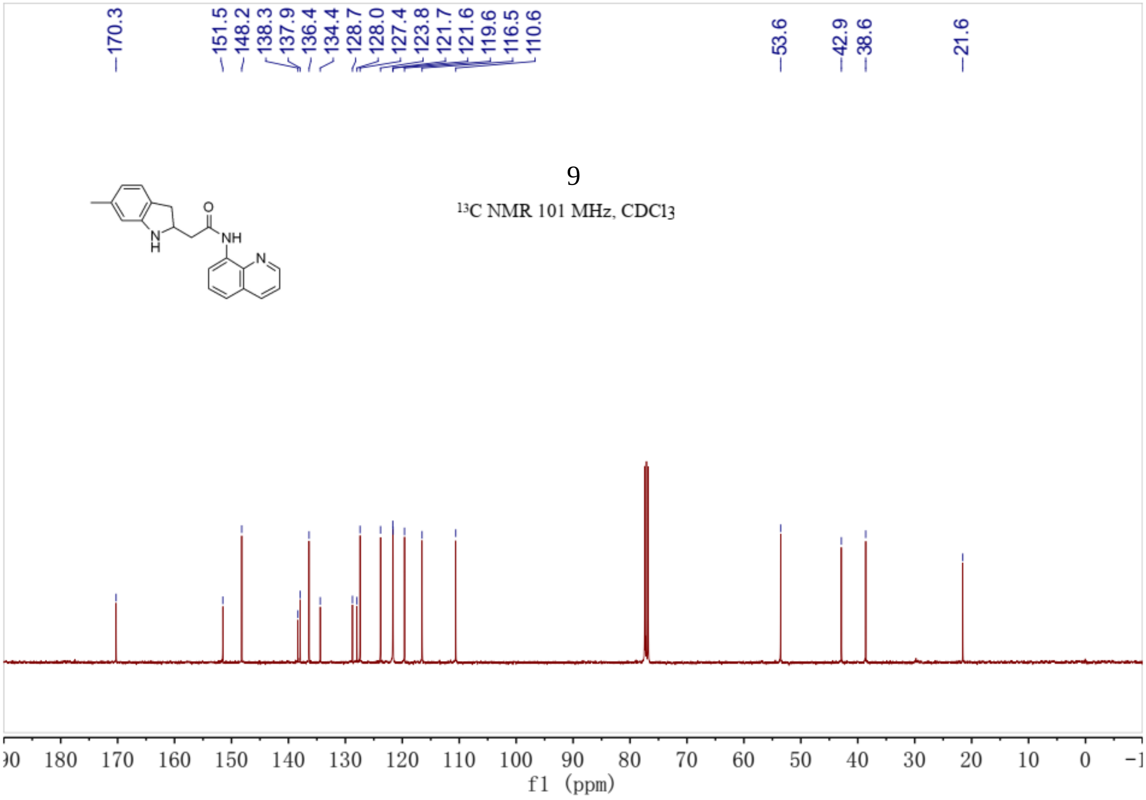
9
13C NMR 101 MHz, CDCl3
170.3
151.5 148.2 138.3 137.9 136.4 134.4 128.7 128.0 127.4 123.8 121.7 121.6 119.6 116.5 110.6
53.6
42.9 38.6
21.6
f1 (ppm)

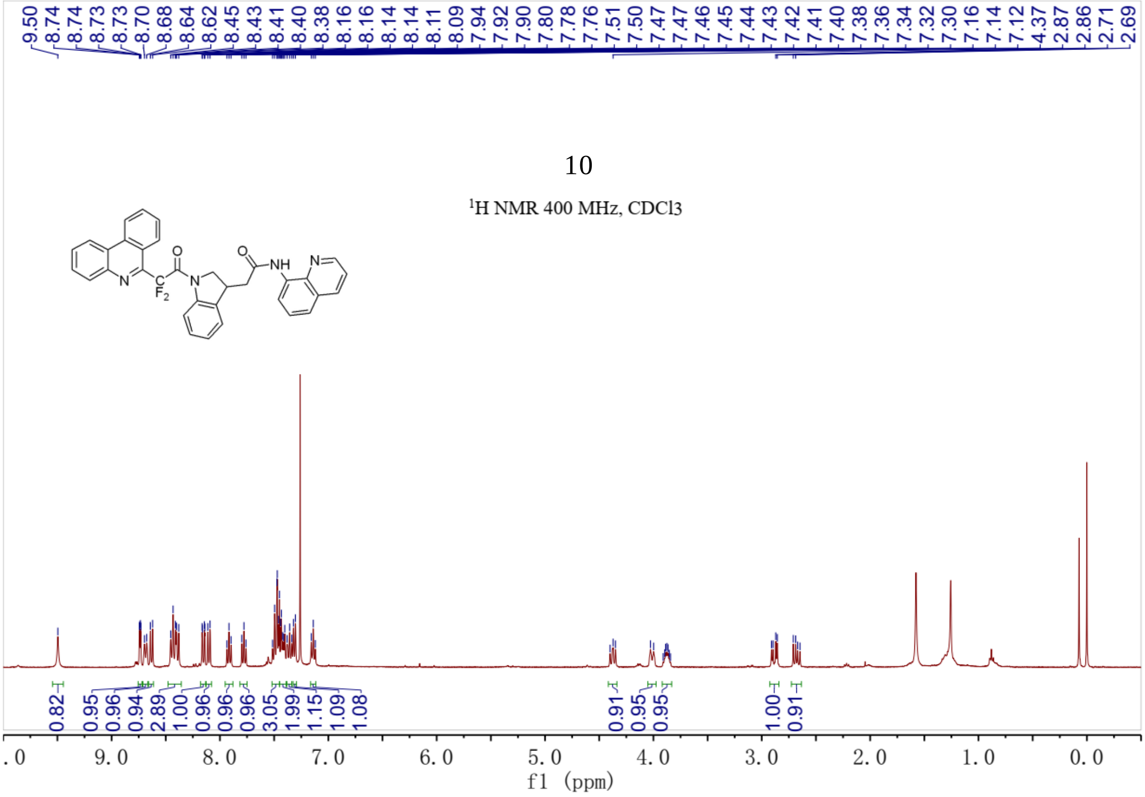
10
1H NMR 400 MHz, CDCl3
f1 (ppm)

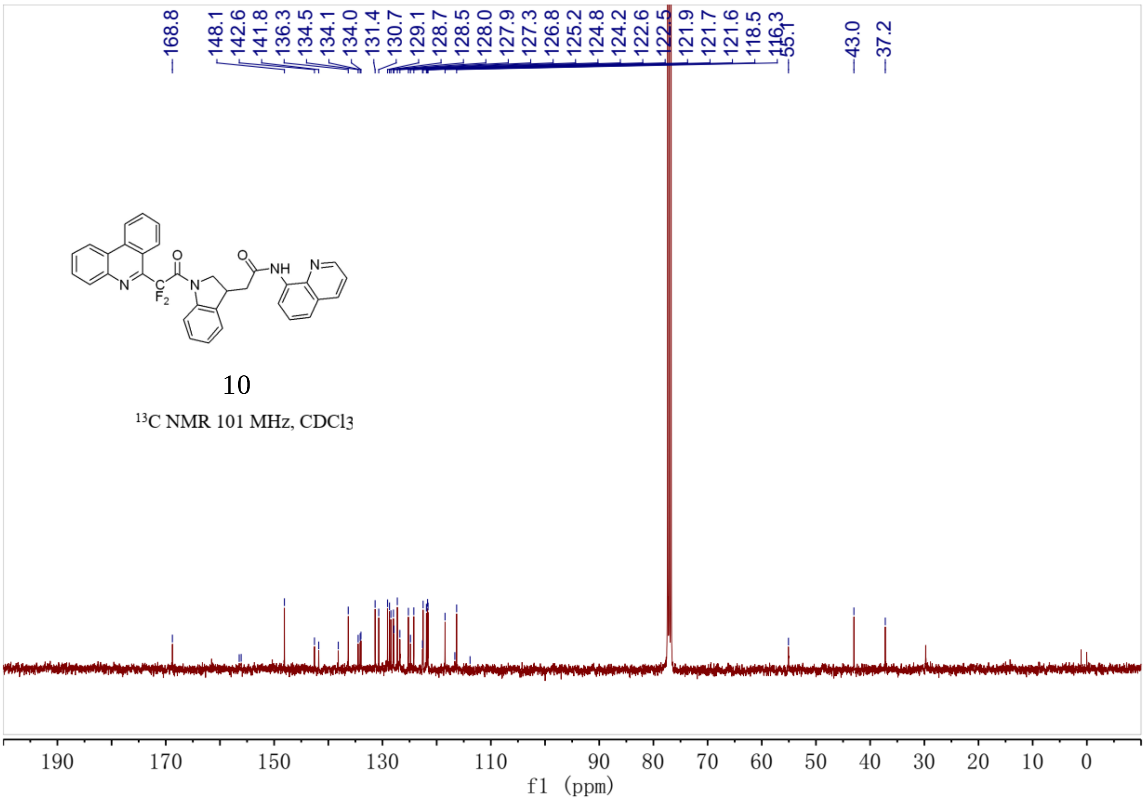
10
13C NMR 101 MHz, CDCl3
f1 (ppm)
